# Plasma-activated media inhibits epithelial-mesenchymal transition and ameliorates intestinal fibrosis through the PPARγ/TGF-β1/SMAD3 pathway

**DOI:** 10.1371/journal.pone.0335225

**Published:** 2025-10-22

**Authors:** Yi You, Yaping Shen, Yan Yang, Xiaoyang Wei, Yuheng Zhou, Foxing Tan, Longcheng Deng, Haolin Du, Sen Wang, Cheng Wang, Yan Huang

**Affiliations:** 1 Nanjing University of Chinese Medicine, Nanjing, China; 2 Nanjing Tech University, Nanjing, China; 3 Department of Ultrasound, Nanjing Hospital of Chinese Medicine Affiliated to Nanjing University of Chinese Medicine, Nanjing, China; Pennsylvania State University Hershey Medical Center, UNITED STATES OF AMERICA

## Abstract

Inflammatory bowel disease often complicates intestinal lumen stenosis, and intestinal fibrosis is the core pathological process leading to its development. Currently, there are no effective drug treatments available to prevent or improve intestinal fibrosis. Previous studies have shown that PAM (plasma-activated media) inhibits epithelial-mesenchymal transition (EMT) and improves skin fibrosis by regulating the PPARγ/TGF-β1 axis. However, it is unclear whether PAM can improve intestinal fibrosis. We used a gradient concentration of PAM to intervene in the dextran sulfate sodium (DSS)-induced mouse intestinal fibrosis model to evaluate its effects onalleviating fibrosis and explore the specific molecular mechanisms. In addition, we used PAM to intervene in the TGF-β1-induced rat intestinal crypt epithelial cell (IEC-6) EMT and fibrosis in an in vitro model to further explore the molecular mechanisms by which PAM improves intestinal fibrosis. We found that PAM can improve intestinal fibrosis by inhibiting epithelial-mesenchymal transition through the PPARγ/TGF-β1/SMAD signaling pathway.

## 1. Introduction

Inflammatory bowel disease (IBD) is a chronic, nonspecific intestinal inflammatory disease induced by multiple factors [1]. IBD is often complicated by intestinal lumen narrowing, and intestinal fibrosis is the core pathological process leading to its development: repeated intestinal inflammatory damage triggers inflammatory cell infiltration, abnormal activation of myofibroblasts, and excessive extracellular matrix deposition, eventually leading to fibrous scarring through abnormal repair mechanisms [2]. As the disease progresses, fibrosis can lead to tissue hardening and intestinal lumen narrowing, severely affecting the patient’s quality of life and potentially causing obstructive complications that threaten life [3]. Although significant progress has been made in understanding the molecular mechanisms of intestinal fibrosis [4,5], treatment for fibrotic stenosis still primarily relies on surgical intervention [6,7]. Aminosalicylic acid preparations are the key drugs for preventing the recurrence of intestinal fibrosis after surgery. The main ones include sulfasalazine (SASP) and different types of 5-aminosalicylic acid (5-ASA) preparations. Due to the fact that SASP is prone to adverse reactions caused by sulfadiazine, 5-ASA is the one commonly used in clinical practice. However, its role in improving intestinal fibrosis during the long-term progression of the disease remains unclear. In clinical practice, attempts have been made to use biologics targeting tumor necrosis factor-α (TNF-α) to improve the fibrosis process [8,9], but long-term efficacy still requires large-scale clinical validation. Meanwhile, although new anti-fibrosis candidate drugs, including traditional Chinese medicines, continue to emerge in basic research [10,11], no drug has yet been approved for clinical prevention or reversal of intestinal fibrosis [12]. This “active basic research-clinical translation gap” highlights the urgent need for the development of effective anti-fibrosis drugs.

Abnormally activated myofibroblasts are a key component in the pathogenesis of intestinal fibrosis, leading to excessive deposition of extracellular matrix (ECM). Epithelial-mesenchymal transition (EMT) [3,13] plays a critical role in this process: epithelial cells undergo EMT to transform into mesenchymal phenotype cells (the main source of abnormally activated myofibroblasts), gaining enhanced migration ability and accumulating at the injury site, with their excessive ECM secretion characteristics promoting pathological repair while exacerbating the fibrosis process. Studies have shown that targeting the inhibition of abnormally activated myofibroblasts can effectively alleviate the progression of intestinal fibrosis [10,11,14].On the molecular mechanism level, the transforming growth factor-β1 (TGF-β1)/SMAD signaling pathway serves as a central hub for fibrosis regulation [15] and presents a dual effect in EMT: On one hand, it downregulates epithelial markers (such as E-cadherin) and upregulates mesenchymal markers (such as N-cadherin, Vimentin), driving cell phenotype transformation; on the other hand, it enhances cell proliferation and migration abilities, ultimately leading to abnormal ECM accumulation [16]. Additionally, peroxisome proliferator-activated receptor-γ (PPAR-γ), a key transcription factor responsible for fatty acid uptake and oxidation in lipid metabolism, has been confirmed to possess anti-fibrotic properties [5]. Its mechanism involves antagonizing the TGF-β1/SMAD pathway to inhibit the EMT process, thereby improving intestinal fibrosis [17,18].

Plasma is the fourth state of matter, distinct from solid, liquid, and gas, composed of reactive radicals, high-energy electrons, and ions generated by ionized gas [19]. Depending on the temperature difference of ionized particles, plasma can be divided into high-temperature and low-temperature types. Low-temperature plasma, with a gas temperature close to room temperature and higher biological safety, has become an important research subject in the biomedical field. The application of plasma in medical research mainly uses two modes: direct plasma irradiation or generation of plasma-activated media (PAM) by treating solution media (such as phosphate-buffered saline, PBS) [20,21]. Studies have shown that plasma technology demonstrates potential applications in biomedical fields such as wound healing, skin disease treatment, and tumor intervention [22–24].In particular, PAM can inhibit the proliferation and migration of fibroblasts in rheumatoid arthritis, alleviate synovial hyperplasia [25], and suppress tumor cell metastasis by blocking EMT [26–28], indicating its inhibitory effect on pathologically activated cells. Further molecular mechanism studies have revealed that PAM inhibits the EMT process and improves wound fibrosis by upregulating the expression of bone morphogenetic protein 7 (BMP7) and PPARγ, while downregulating TGF-β1 levels [29]. A similar mechanism is also observed in the skin scar model, where PAM alleviates fibrosis by inhibiting TGF-β1-mediated collagen deposition [30].

In basic research on intestinal fibrosis, it has been found that regulating the PPARγ/TGF-β1/SMAD signaling pathway to inhibit the EMT process can improve intestinal fibrosis [18].In plasma-based research, it has been found that PAM regulates the PPARγ/TGF-β1 axis to inhibit EMT, improve skin fibrosis [29], and suppress the proliferation and migration of abnormally activated cells. Based on this, the present study proposes the hypothesis that PAM may improve intestinal fibrosis by inhibiting EMT through the PPARγ/TGF-β1/SMAD3 pathway. To verify the scientific hypothesis, we used gradient concentrations of PAM to intervene in a dextran sulfate sodium (DSS)-induced mouse intestinal fibrosis model to evaluate its effects on alleviating fibrosis and explore the specific molecular mechanisms. In addition, we used PAM to intervene in the TGF-β1-induced rat intestinal crypt epithelial cell (IEC-6) EMT and fibrosis in vitro to further explore the molecular mechanisms by which PAM improves intestinal fibrosis. This study provides a theoretical basis for expanding the medical applications of PAM.

## 2. Materials and methods

### 2.1 Reagents and antibodies

The recombinant growth factor TGF-β1 (Lot: 0521209) was procured from PeproTech, Cranbury, NJ, USA. The PPARγ antagonist GW9662 (CAS number: 22978-25-2) was sourced from MCE, Monmouth Junction, NJ, USA. We used the specific antibodies from Abmart Company in Shanghai, China: E-cadherin, N-cadherin, and Vimentin. Additionally, we purchased the antibodies targeting α-SMA (14395–1-AP), PPARγ (16643–1-AP), TGF-β1 (26155–1-AP), and Tubulin (10094–1-AP) from Proteintech, Wuhan, China. We acquired the SMAD3 antibody (ab84177) and the phosphorylated SMAD3 antibody (ab52903) from Abcam, situated in Cambridge, United Kingdom. The SMAD3 antibody (ab84177) and the phosphorylated SMAD3 antibody (ab52903) were purchased from Abcam, Cambridge, UK. The GAPDH antibody (GB11002−100) was sourced from Servicebio, Wuhan, China. The β-actin antibody (ACD26) was acquired from ABclonal, Wuhan, China. Finally, the secondary antibody targeting rabbit (L3012) was obtained from SAB, Shanghai, China.

### 2.2 Preparation of PAM

Preparation conditions: The plasma device was supplied with argon gas flowing at 300 milliliters per minute, and the nanosecond pulse voltage was tuned to 3 kHz. The plasma source was positioned 3 mm away from the PBS solution, which was sterile and maintained at a pH of 7.0–7.2. A total volume of 30 ml of PBS was treated with a 7 kV pulse for 10 minutes, then kept at −80°C. We will use up the plasma solution within two days after its preparation. The concentrations of the long-acting components H_2_O_2_, NO_3_^-^, and NO_2_^-^ were measured using a UV spectrophotometer.

### 2.3 Animal experiments

C57BL/6 mice (6–8 weeks old, male, approximately 23g) were provided by Beijing Vital River Laboratory Animal Technology Co., Ltd (Production License: SCXK (Zhe) 2020–0002). Randomly assigned eight SPF-grade C57BL/6 mice to each group, resulting in a total of six groups. After 7 days of adaptive feeding, the control group was given deionized water, while the other five groups received 2.5% DSS (60316ES60, Yeasen Biotechnology Co., Ltd., Shanghai, China) for 5 days (with the solution refreshed every two days), followed by a 7-day period of DSS withdrawal and deionized water, repeated for three cycles [18]. On the second day of DSS administration, the treatment groups (L-PAM, M-PAM, H-PAM) were given corresponding concentrations of plasma-activated medium (Intraperitoneal injection of 100ul). Among them, the currently prepared PAM is used as the H-PAM group, the PAM diluted 1 times with PBS is used as the M-PAM group, and the PAM diluted 2 times with PBS is mixed together to form the L-PAM group. Meanwhile, the positive control group of mice was administered 5-aminosalicylic acid at a dosage of 200 mg/kg [11]. Throughout the experimental duration, daily observations were made on the mice regarding their body weight, stool properties, and the presence of occult blood. The Disease Activity Index (DAI) scores were calculated based on these three parameters: body weight, fecal shape, and occult blood (as shown in Supplementary Table 1). Culminating in anesthesia for blood sample collection by enucleation at the end of the period. The animals were then euthanized by cervical dislocation. The Animal Committee of the First People’s Hospital of Nanjing approved the animal experiment based on the institution’s animal ethics guidelines (Ethics Number: DWSY-24162620).

### 2.4 Cell culture

Originating from Zhong Qiao Xin Zhou Biotechnology Co., Ltd. (Shanghai, China; item code ZQ0783), IEC-6 cells, representing normal intestinal epithelial cells from rats, were utilized. These cells were propagated in DMEM medium fortified with high glucose, 1% penicillin-streptomycin solution, 0.01 mg/mL insulin, and 10% fetal bovine serum (FBS), all acquired from the aforementioned company. We cultured the cells in a cell incubator at 37°C with 5% CO2.

### 2.5 Cell treatment

TGF-β1, as a stimulating factor, can induce epithelial-mesenchymal transformation. Different concentrations of TGF-β1(0–12 ng/mL) are applied to IEC-6 cells, CCK8 detection is performed, and subsequent modeling is conducted to find the lowest concentration that can produce proliferative activity on IEC-6 cells. TGF-β1 was found to promote cell proliferation at a concentration of 10 ng/mL (Supplementary Fig. 1). Different concentrations of PAM (0%−10%, PAM diluted with complete medium) were applied to IEC-6 cells for CCK8 detection. Subsequent cell experiments were conducted to find PAM concentrations that had no significant effect on IEC-6 cell viability, and it was found that concentrations below 6% had no significant effect on cell viability. However, the cell viability was inhibited at 8% PAM concentration. Therefore, 2%, 4% and 6% PAM concentrations were selected for subsequent experiments (Supplementary Fig. 2). Subsequently, the interstitial transformation and fibrosis of IEC-6 cells were induced by TGF-β1, and CCK8 was detected by adding PAM to observe cell proliferation. At the same time, the cells were seeded in 6-well plates with 2 × 105 seeds per well and incubated in incubators. When the cell adhesion density reached more than 50%, the cells were starved with serum-free medium for 24 h and incubated with TGF-β1 and PAM for 48 h. Cell RNA and protein were extracted to detect the effects of PAM on TGF-β1-induced IEC-6 cell interstitial transformation and fibrosis. In another experiment, the cells were exposed to DMEM containing 10% fetal bovine serum, 10 ng/mL TGF-β1, 6% PAM, and a GW9662 inhibitor (10uM) for 48 hours. Cell morphology was assessed using an inverted microscope (Leica DMI3000B), and cells were then collected.

### 2.6 Hematoxylin-eosin and masson’s staining

Following euthanasia by cervical dislocation, tissues from the heart, liver, lungs, kidneys, and colon were harvested. All tissues, except the colon, were fixed in 4% paraformaldehyde. A segment of the colon was carefully dissected, with the intestinal contents removed, and the tissue was then fixed in paraformaldehyde. The remaining tissues underwent dehydration and were stored at −80°C after paraformaldehyde removal. After a 24-hour fixation period in paraformaldehyde, 5-micron-thick sections were continuously cut from each mouse’s tissue block for further analysis. Histological staining with hematoxylin and eosin (HE) was performed on the heart, liver, lungs, kidneys, and colon tissues, while Masson’s trichrome staining was applied specifically to the colon tissue. Microscopic images were captured at 20x magnification using an OLYMPUS DP74 microscope, and then analyzed based on the pathological injury score and Masson staining score. Among them, the pathological injury score is the sum of the scores for acute inflammatory cell infiltration (0–4 points), chronic inflammatory cell infiltration (0–3 points), and crypt damage (0–4 points). The score for crypt damage: 0 points, crypt intact; 1 point, one-third of the crypt lost; 2 points, two-thirds of the crypt lost; 3 points, complete loss of the crypt; 4 points, both the crypt and the epithelium lost. The Masson staining score is determined by using ImageJ software to analyze the proportion of collagen fibers in the area of colon tissue.

### 2.7 ELISA assay

Blood samples collected from the eyeballs of mice were left at ambient temperature for 2 hours, followed by centrifugation at 1000 g for 15 minutes to isolate the serum supernatant. For the quantitative assessment of TNF-α and IL-6 concentrations in the serum, ELISA kits manufactured by Elabscience Biotechnology Co., Ltd. in Wuhan, China, were utilized.

### 2.8 Western blot

Colon tissue (50 mg) from mice or cells after 48-hour incubationwere lysed in Radio-Immunoprecipitation Assay (RIPA) buffer (Epizyme Biotech, Shanghai, China; product code PC101), supplemented with protease and phosphatase inhibitors (GRF101 and GRF102, respectively, also from Epizyme Biotech) at a ratio of 100:1:1. Following homogenization, the lysate was centrifuged at 8000 g for a duration of 10 minutes, yielding a supernatant which was subsequently collected. The abundance of proteins within this supernatant was quantified using a kit manufactured by Beyotime Biotechnology, bearing the product code P0012. For protein sample preparation, water and loading buffer (LT101S, Epizyme Biotech) were added, followed by thorough mixing, heating at 100°C for 10 minutes, cooling on ice, centrifugation, and loading onto gels for electrophoresis. Post transfer and blocking, the membranes underwent incubation with specific primary antibodies at 4°C for a duration of 16 hours. This was followed by an incubation with a secondary antibody at ambient temperature for one hour, culminating in the development of the protein bands.

### 2.9 Real-time quantitative PCR

Colon tissue (30 mg) from mice or cells after 48-hour incubation was added with 500 microliters of RNA extraction reagent. The mixture was homogenized and centrifuged, and to extract RNA, use the RNA Extraction Kit (RC112) provided by Vazyme Biotech Co., Ltd, Nanjing, China. Elute the RNA using 40 microliters of deionized water in a spin column, and then determine its concentration. After extraction, the RNA samples were converted into cDNA utilizing the R223 kit supplied by Vazyme Biotech Co., Ltd., adhering strictly to the manufacturer’s protocols, and cDNA levels were detected using a Quantitative Fluorescence PCR Instrument (ABI 7500 model). The primer sequences employed for our research are detailed in Supplementary Table 2 and 3.

### 2.10 Cell proliferation assay

Cells in the logarithmic phase of growth were distributed into 96-well plates, with each well containing 5000 cells. When the cells attached successfully and their confluence exceeded 60%, they were subjected to 24 hours of starvation in DMEM culture medium with high sugar components. The medium was then added with plasma-activated medium (PAM) and TGF-β1 according to the experimental purpose, for an additional 48 hours. In a parallel experiment, cells were treated with DMEM including 10% FBS, 6% PAM, 10 ng/mL of TGF-β1, and the GW9662 inhibitor(10uM), also for 48 hours. After the medium was removed, each well was supplemented with 10 µL of CCK8 reagent (APExBIO Technology LLC, Shanghai, China, product code K1018). Subsequently, the plates were placed in an incubator under the prescribed cultural conditions for a period of 30 minutes, followed by the measurement of optical density (OD) values at a wavelength of 450 nm utilizing a BioTek SynergyHI MFD microplate reader.

### 2.11 Wound healing assay

Cells undergoing logarithmic growth were inoculated into 6-well culture plates, with each well containing a density of 2 x 10^5 cells. Once the cells adhered to the 6-well plate and achieved a confluence exceeding 50%, they were subjected to starvation conditions in high-glucose DMEM medium for a duration of 24 hours. Afterwards, the cells were exposed to a new medium comprising 10% fetal bovine serum (FBS), varying levels of plasma-activated medium (PAM) at 2%, 4%, or 6%, and 10 ng/mL of TGF-β1, for a period of 48 hours. In an alternative experiment, cells underwent a 48-hour treatment with DMEM supplemented with 10% FBS, 6% PAM, 10 ng/mL of TGF-β1, and the GW9662 inhibitor(10uM). Upon reaching full confluence, a cross-shaped wound was inflicted upon the monolayer using the pointed end of a 200-µL pipette. The cells were then washed three times with phosphate-buffered saline (PBS), and then supplemented with serum-free medium. The migration of cells was monitored at 0 and 24 hours with an inverted microscope, followed by quantitative analysis of the migration area using ImageJ software.

### 2.12 Statistical analysis

Version 9 of GraphPad Prism software was utilized to perform the statistical analyses. When comparing two groups, we employed the Student’s t-test, whereas for assessments involving several groups, a one-way ANOVA was applied. The data are reported as mean ± standard deviation (SD), with statistical significance determined at a p-value below 0.05.

## 3. Results

### 3.1 PAM improves symptoms and biosafety analysis in mice with intestinal fibrosis

**Fig 1:**
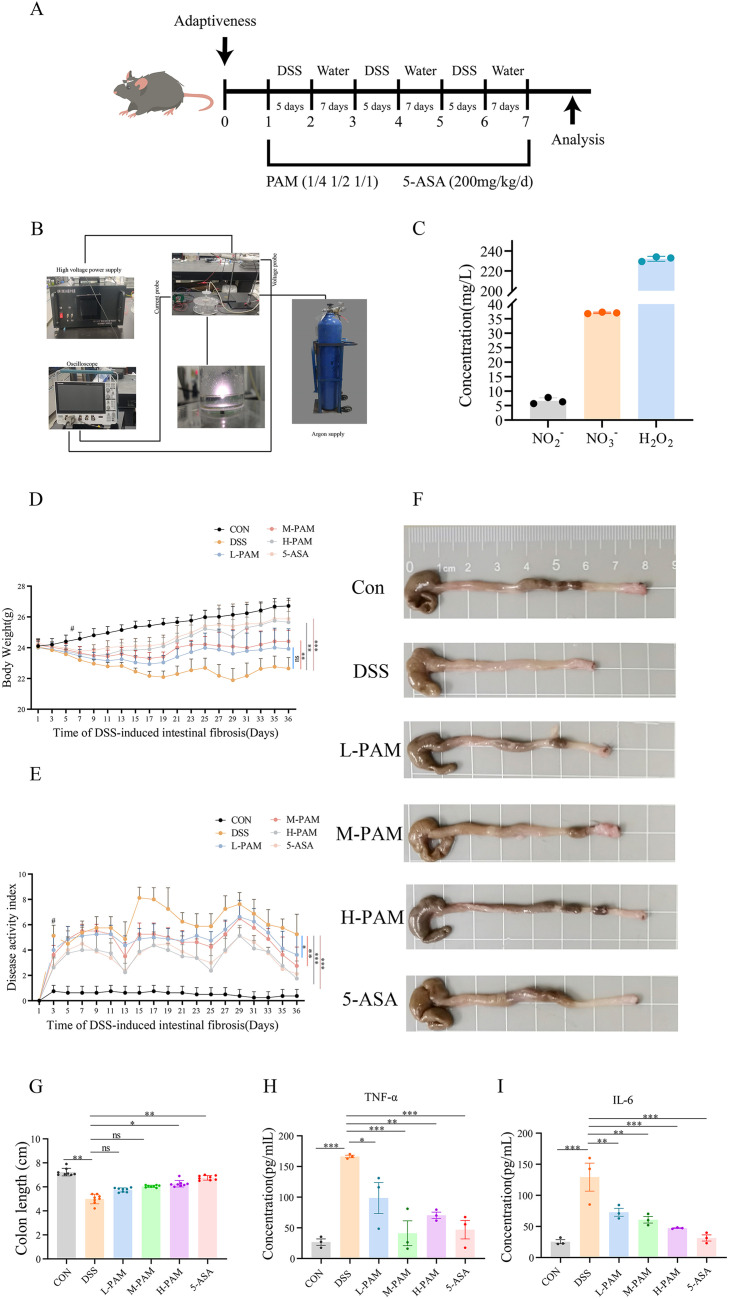
PAM can improve DSS-induced intestinal fibrosis in mice and reduce the production of inflammatory factors. **(A)** Model induction time, drug administration time, and dosage. **(B)** Apparatus for preparing PAM. **(C)** Active components of PAM. **(D)** Weight changes of mice in different groups during the model induction period. **(E)** DAI scores of mice in different groups during the model induction period. **(F)** Colon length of mice in different groups. **(G)** Bar chart of colon length changes in different groups of mice. **(H)** Changes in serum TNF-α levels in different groups of mice. **(I)** Changes in serum IL-6 levels in different groups of mice.

PBS was activated using a cold plasma device (Fig. 1B), generating reactive species such as nitrite, nitrate, and reactive oxygen species (ROS). Long-lasting active components were detected with a specialized device (Fig. 1C). Mice belonging to the intestinal fibrosis model group displayed a significant reduction in body weight. A statistically significant difference was evident on Day 6 in comparison to the control group. Upon completion of the modeling period, weight loss in the M-PAM, H-PAM, and 5-ASA treatment groups was markedly improved contrasted to the model group (Fig. 1D). As depicted in Fig 1E, on Day 3, the DAI scores of the model group showed a notable increase in comparison to the control group. By the final day, DAI scores in the treatment groups were reduced. Figs 1F and 1G present gross images and statistical results of colon length, indicating that both the plasma-activated medium and the positive drug group alleviated colon shortening in the intestinal fibrosis model. In addition, the expression levels of TNF-α and IL-6 increased in the model group, while the expression levels of TNF-α and IL-6 decreased after treatment with PAM or 5-ASA, indicating that PAM and 5-ASA can inhibit inflammatory factors and improve fibrosis (Figs 1H and 1I).

**Fig 2:**
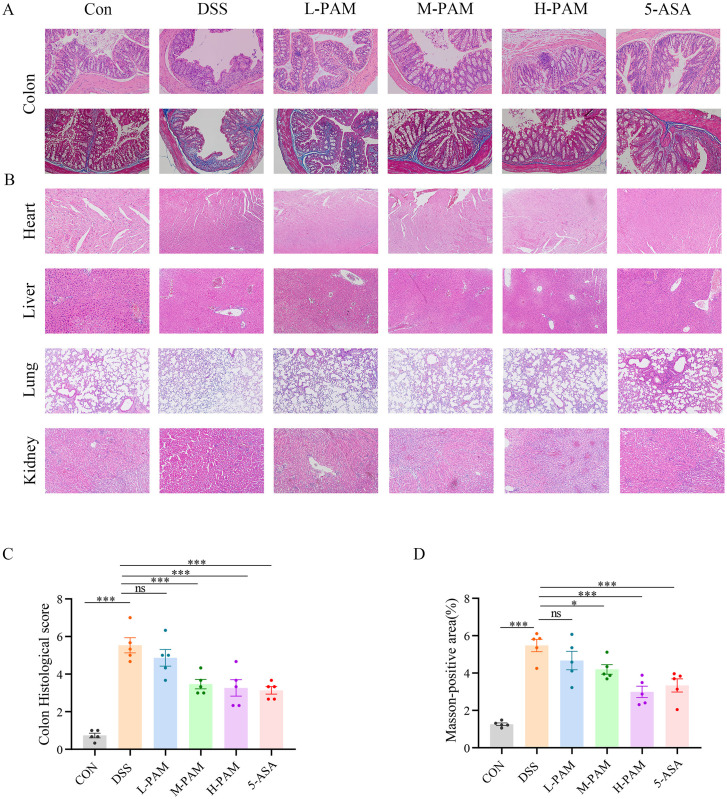
PAM improves the pathology of intestinal fibrosis in mice, and no obvious lesions were observed in the toxicity experiments on the heart, liver, lungs, and kidneys. **(A)** HE staining and Masson staining changes in the colon of mice in different groups(n = 6) (200x). **(B)** HE staining changes in the heart, liver, lungs, and kidneys of mice in different groups(n = 3) (200x). **(C)** Pathological damage score of the mouse colon. **(D)** Colon fibrosis severity score in mice.

As illustrated in Figs 2A and 2G, the histological assessment using hematoxylin and eosin (HE) staining demonstrated that the intestinal tissues from the treatment group exhibited notably lower pathological damage scores in comparison to those from the model group. Moreover, Figs 2B and 2H present evidence that the treatment group exhibited decreased collagen deposition, as demonstrated by Masson’s trichrome staining, in contrast to the model group. HE staining also demonstrated that plasma-activated medium did not induce toxicity in the heart, liver, lungs, or kidneys of the mice (Figs 2C-F).

### 3.2 PAM improves DSS-induced intestinal fibrosis in mice by suppressing EMT through the PPARγ/TGF-β1/SMAD3 pathway

**Fig 3:**
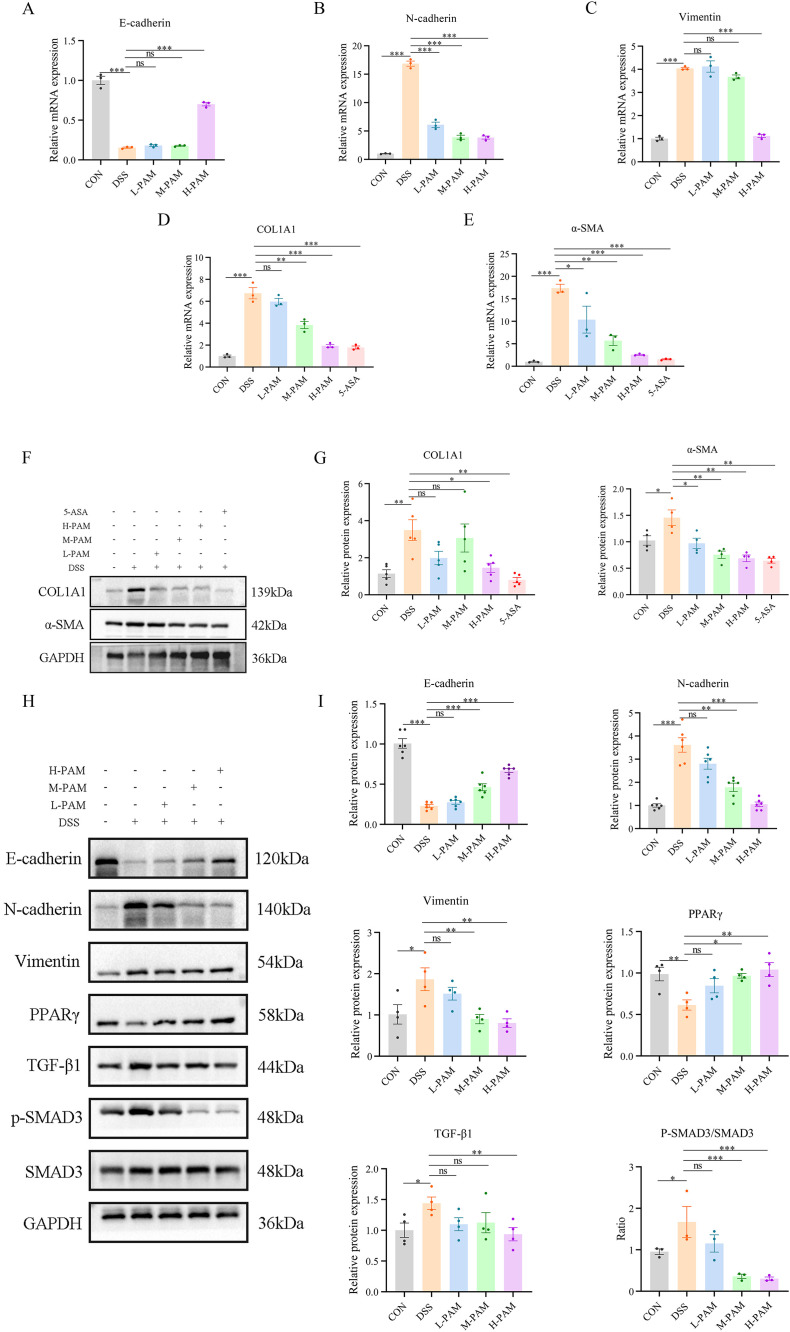
After the injection of PAM, the level of PPARγ increases, and the expression of TGFβ1/SMAD3 decreases, thereby inhibiting EMT and improving intestinal fibrosis. (A) Changes in the mRNA expression level of E-cadherin. (B) Changes in the mRNA expression level of N-cadherin. (C) Changes in the mRNA expression level of V-cadherin. (D) Changes in the mRNA expression level of COL1A1. (E) Changes in the mRNA expression level of α-SMA. (F, G) Changes in the protein expression level of COL1A1 and α-SMA. (H, I) Changes in the expression levels of EMT and pathway-related proteins.

In the context of IBD, epithelial cells undergo a transformation known as EMT, where they convert into mesenchymal cells. This process results in heightened production of the extracellular matrix, ultimately leading to intestinal fibrosis. Mice subjected to DSS-induced intestinal fibrosis exhibited significantly higher protein and mRNA expression of fibrosis markers, specifically COL1A1 and α-SMA. In an in vitro setting where TGF-β1 induced intestinal EMT, we observed an upregulation of fibrosis markers, notably COL1A1 and α-SMA, coupled with a downregulation of E-cadherin expression. Additionally, there was a notable increase in the levels of N-cadherin and Vimentin. Both protein and mRNA expression levels of COL1A1 and α-SMA were markedly reduced by treatment with plasma-activated medium (PAM), effectively suppressing the EMT process as a result (Figs 3A-G). This was achieved by enhancing E-cadherin expression and reducing N-cadherin and Vimentin expression. PPAR-γ, a key regulator in lipid metabolism, has been recognized as an antifibrotic agent due to its role in fatty acid uptake and oxidation. Additionally, the TGF-β/SMAD signal pathway is a central mechanism driving the pathogenesis of intestinal fibrosis [31]. Previous studies have shown that modulation of the PPARγ/TGF-β1/SMAD axis can mitigate DSS-induced intestinal fibrosis in the IBD model [18]. In the mouse model of DSS-induced intestinal fibrosis, as illustrated in Figs 3H-I, a reduction in PPARγ expression was observed, alongside an increase in TGF-β1 and SMAD3 expression. Remarkably, within the treatment groups, a dose-dependent augmentation in PPARγ expression was observed as the concentration of plasma-activated medium was elevated, accompanied by a corresponding decline in the abundance of both TGF-β1 and SMAD3.

### 3.3 PAM inhibits IEC-6 cell proliferation and migration

**Fig 4:**
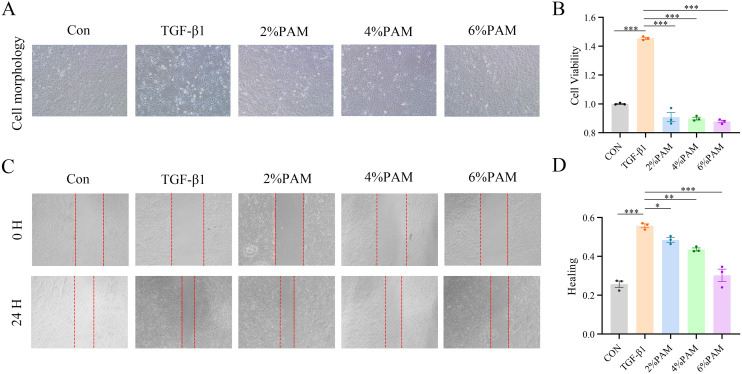
PAM inhibits TGF-β1-induced proliferation and migration of intestinal epithelial cells. (A) Changes in cell morphology (100x). (B) Changes in cell proliferation. (C) The cell scratch assay indicates cell migration (50x). (D) Changes in cell migration ability.

In the context of establishing an EMT model of intestinal fibrosis, TGF-β1 induces the transformation of IEC-6 intestinal epithelial cells into mesenchymal cells, leading to extracellular matrix production. To investigate how TGF-β1 impacts the survival of IEC-6 cells, a concentration gradient was applied. The findings indicated that a 10 ng/mL concentration of TGF-β1 stimulated cell growth. Concurrently, the expression of the mesenchymal markers N-cadherin and Vimentin increased, whereas the expression of the epithelial marker E-cadherin decreased. CCK8 assays were then used to assess the impact of different concentrations of plasma-activated medium (PAM) on cell viability, as well as PAM’s modulatory role in the presence of 10 ng/mL TGF-β1. The research revealed that PAM concentrations ranging from 2% to 6% had no impact on the viability of normal IEC-6 cells, but suppressed the viability of IEC-6 cells undergoing EMT (Fig 4B). PAM concentrations of 2%, 4%, and 6% were selected for further experiments. TGF-β1 stimulation caused the cells to elongate and enhanced their migratory ability, whereas PAM reduced these morphological changes and inhibited cell migration (Figs 4A and 4C).

### 3.4 PAM suppresses epithelial stromal transformation in IEC-6 cells via the PPAR γ/ TGF s/ β 1/ SMAD3 signaling pathway

**Fig 5:**
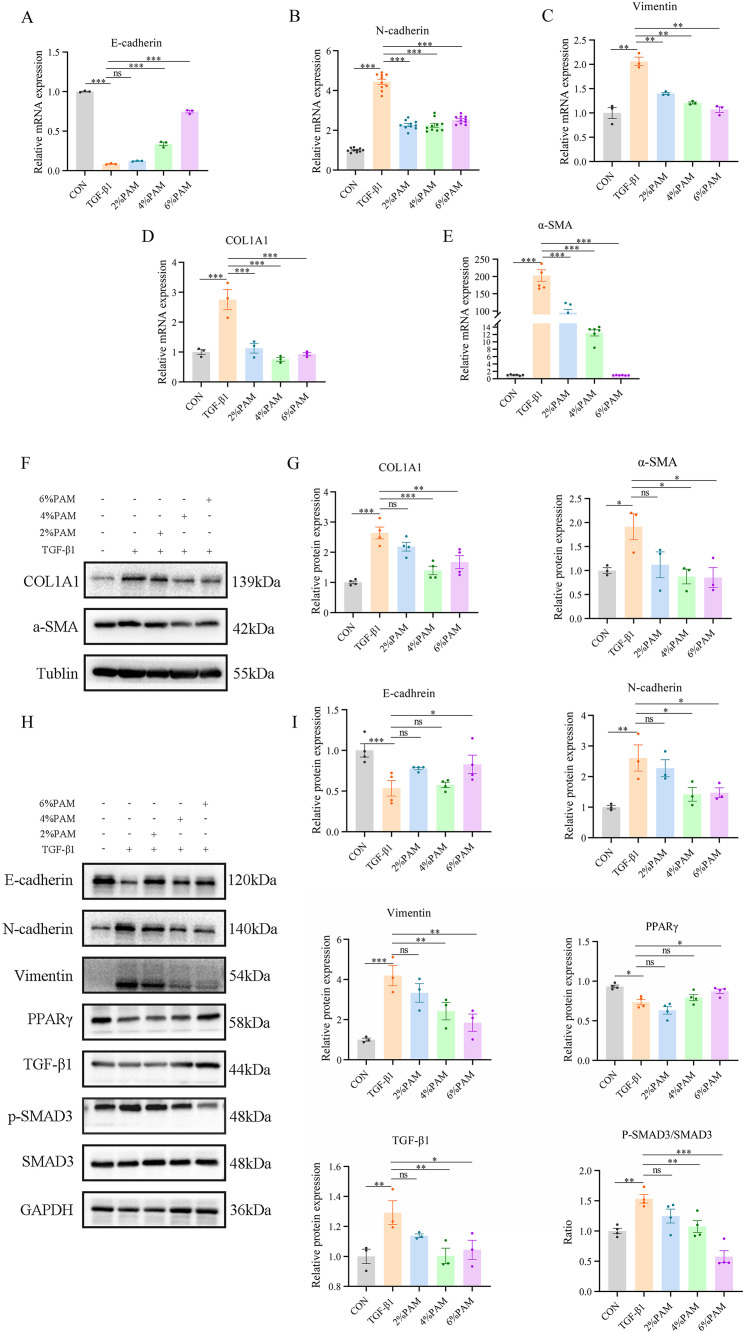
PAM inhibits TGF-β1-induced EMT fibrosis in intestinal epithelial cells through upregulating the expression of PPARγ and downregulating the expression of TGF-β1/SMAD3. (A) Changes in the mRNA expression level of E-cadherin. (B) Changes in the mRNA expression level of N-cadherin. (C) Changes in the mRNA expression level of V-cadherin. (D) Changes in the mRNA expression level of COL1A1. (E) Changes in the mRNA expression level of α-SMA. (F, G) Changes in the protein expression level of COL1A1 and α-SMA. (H, I) Changes in the expression levels of EMT and pathway-related proteins.

In an in vitro model where intestinal EMT was induced by TGF-β1, we noted an increase in the expression of fibrosis markers, including COL1A1 and α-SMA, accompanied by a decrease in the expression of E-cadherin. Furthermore, there was an elevation in the levels of N-cadherin and Vimentin. In parallel, PPARγ expression was diminished, while the levels of phosphorylated SMAD3 (P-SMAD3)/total SMAD3 and TGF-β1 were elevated. Treatment with plasma-activated medium (PAM) resulted in a restoration of PPARγ expression and a reduction in TGF-β1 and P-SMAD3/SMAD3 levels. This treatment resulted in the inhibition of EMT, evidenced by an increase in E-cadherin expression and a decrease in N-cadherin and Vimentin expression. Furthermore, there was a significant diminution in the expression of fibrosis markers (as shown in Figs 5A-I).

### 3.5 GW9662 intervention the effects of PAM in IEC-6 cells

**Fig 6:**
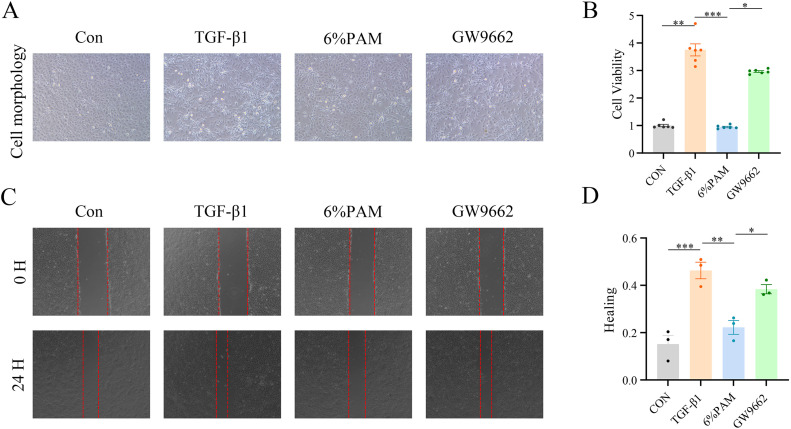
GW9662 (PPARγ inhibitor) inhibits PAM from blocking TGF-β1-induced proliferation and migration of intestinal epithelial cells. (A) Changes in cell morphology (100x). (B) Changes in cell proliferation. (C) The Cell scratch assay indicates cell migration (50x). (D) Changes in cell migration ability.

**Fig 7:**
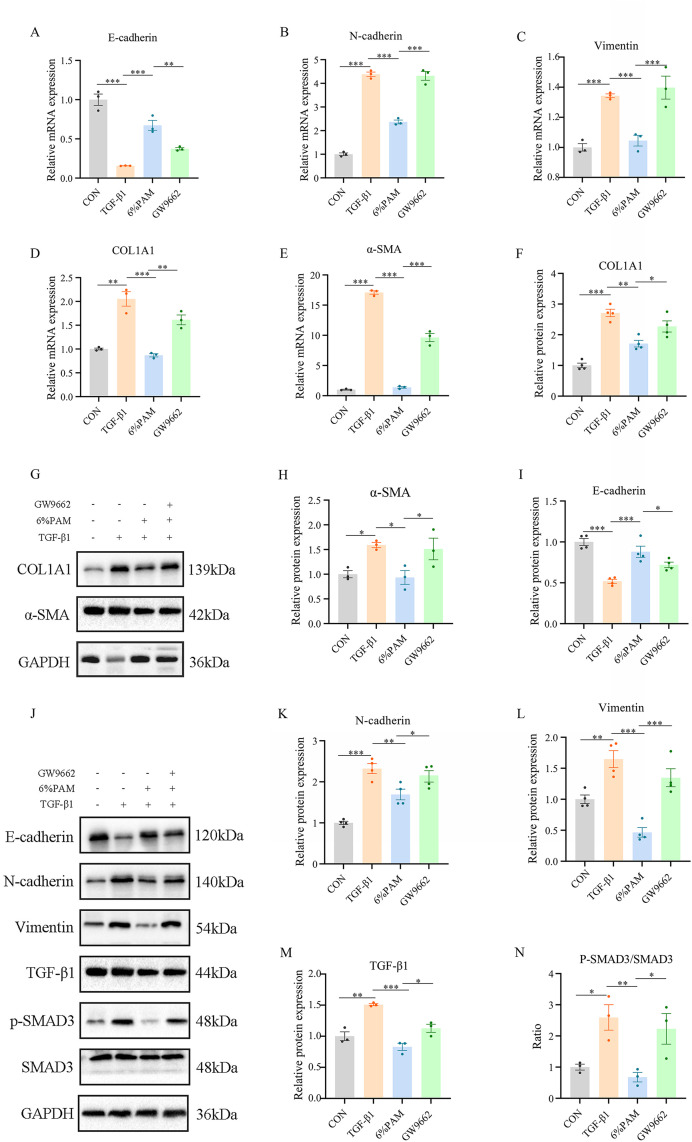
After GW9662 (PPARγ inhibitor) intervention, PPARγ expression was inhibited, and TGF-β1/SMAD3 expression was increased, thereby suppressing the inhibitory effect of PAM on the fibrotic process of intestinal epithelial cells induced by TGF-β1. (A) Changes in the mRNA expression level of E-cadherin. (B) Changes in the mRNA expression level of N-cadherin. (C) Changes in the mRNA expression level of V-cadherin. (D) Changes in the mRNA expression level of COL1A1. (E) Changes in the mRNA expression level of α-SMA. (F-H) Changes in the protein expression level of COL1A1 and α-SMA. (I-N) Changes in the expression levels of EMT and pathway-related proteins. (# p < 0.05; ##p < 0.005; ###p < 0.0005, * p < 0.05; ** p < 0.005; *** p < 0.0005).

6% PAM, which exhibited the most effective inhibition of EMT, was selected for further inhibitor experiments. When co-administered with the PPARγ inhibitor GW9662, a change in cell morphology was observed, with cells becoming elongated. This morphological alteration was accompanied by enhanced cell proliferation and migration abilities (Figs 6 A-D). Additionally, there was an upregulation of fibrosis-related indicators like COL1A1 and α-SMA, coupled with a downregulation of E-cadherin. Additionally, there was an increase in the expression of N-cadherin and Vimentin. Furthermore, there was a notable decrease in PPARγ expression, accompanied by a significant increase in the levels of P-SMAD3 and TGF-β1, when compared to total SMAD3 (Figs 7 A-N).

## 4. Discussion

In the process of intestinal fibrosis associated with IBD, EMT is a key pathological process. The TGF-β1/SMAD3 signaling pathway is one of the important mechanisms promoting EMT and fibrosis, while PPARγ has a potential protective effect in anti-fibrosis and anti-EMT [18,32]. EMT can be inhibited and intestinal fibrosis improved through the PPARγ/TGF-β1/SMAD3 pathway.In recent years, the application of plasma in the medical field has been expanding. Studies show that PAM can upregulate PPARγ, downregulate TGF-β1, inhibit EMT, and improve the degree of fibrosis in skin wound healing [29]. However, whether PAM can exhibit anti-fibrotic effects similar to those in skin wounds in intestinal fibrosis remains to be clarified. Therefore, the main objective of this study is to explore the improvement effect and mechanism of PAM in intestinal fibrosis. Our experimental results show that PAM significantly improved the clinical symptoms of intestinal fibrosis mice, including weight recovery, significant reduction in DAI scores, and recovery of intestinal tissue length. Additionally, PAM exerted anti-fibrotic effects by regulating EMT markers, fibrosis-related factors, and the PPARγ/TGF-β1/SMAD3 signaling pathway. Further in vitro experiments also indicated that PAM could effectively inhibit the TGF-β1-induced EMT and fibrosis process via the PPARγ/TGF-β1/SMAD3 signaling pathway. Notably, pathological evaluations of major organs such as the heart, liver, lungs, and kidneys confirmed that long-term PAM intervention has good safety.

Our research results show that PAM can significantly reduce intestinal mucosal tissue damage and lower the expression levels of inflammatory factors TNF-α and IL-6. Its efficacy is comparable to 5-ASA, which is consistent with the results of our previous research, suggesting that PAM can alleviate intestinal inflammation in IBD-induced intestinal fibrosis mice. Anti-inflammatory intervention is an important step in preventing intestinal fibrosis [33], but simply inhibiting the inflammatory response is insufficient to block the fibrosis process [34]. In this study, Masson staining revealed that PAM showed better improvement in reducing collagen fiber deposition compared to the 5-ASA group, indicating that its anti-fibrotic mechanism may extend beyond merely anti-inflammatory effects. It is worth noting that although TNF-α and IL-6 are routinely used as inflammation markers, they also play a pro-fibrotic role in the process of intestinal fibrosis [4]. This not only highlights the synergy between anti-inflammatory and anti-fibrotic mechanisms but also suggests that fibrosis regulation involves the integration of multiple pathway networks.

Our experimental results show that PAM upregulates PPARγ, inhibits EMT, and reduces collagen deposition, consistent with existing studies [29]. Additionally, PAM’s inhibitory effect on the TGF-β1/SMAD3 signaling pathway is also in line with findings from studies such as Frescaline N [35]. Through in vitro experiments, we first revealed that PAM upregulates PPARγ, downregulates the TGF-β1/SMAD3 signaling pathway, inhibits EMT, and improves fibrosis, which was further confirmed by the reversal effect of GW9662 on PPARγ inhibition. It is worth noting that GW9662 only partially reverses the effects of PAM, suggesting that besides the PPARγ/TGF-β1/SMAD3 core pathway, other regulatory pathways may be involved in a synergistic effect. For example, existing literature indicates that PAM can improve skin wound fibrosis by inhibiting fibroblast activation and reducing collagen production [29]. In the pathological mechanism of intestinal fibrosis, various cells, including fibroblasts, are involved in regulation [12]. Inhibiting fibroblast activity can alleviate intestinal fibrosis [30]. These results suggest that PAM may exert its anti-fibrotic effects through multiple cellular targets and a coordinated network of signaling pathways.

The treatment of IBD-induced intestinal fibrosis has always been a clinical challenge, and currently, there is a lack of effective treatment options. This study, through systematic in vivo and in vitro experiments, demonstrates that PAM, as a novel therapeutic strategy, can improve clinical symptoms in intestinal fibrosis model mice, reduce intestinal tissue fibrosis, and exert its effects by inhibiting EMT through the PPARγ/TGF-β1/SMAD3 signaling pathway. This provides a theoretical basis for expanding the application of PAM in basic medical research.

Although this study provides preliminary evidence that PAM can slow the progression of intestinal fibrosis, there are still some limitations. First, GW9662 only partially reverses the effects of PAM, suggesting that PAM may exert its effects through multiple targets and pathways. Therefore, future studies should further explore the molecular mechanisms underlying PAM’s action. Additionally, we assessed the long-term safety of PAM only through H&E staining, and further comprehensive experiments are needed to verify its safety.

## 5. Conclusions

In summary, our study indicates that PAM can ameliorate DSS-induced intestinal fibrosis. The mechanism may be related to the inhibition of the epithelial-mesenchymal transition of intestinal cells. Moreover, we have also found that PAM can inhibit the epithelial-mesenchymal transition through the PPARγ/TGF-β1/SMAD signaling pathway. However, the anti-fibrotic effect of PAM involves multiple cells and targets. Therefore, further in-depth exploration of the anti-fibrotic mechanism of PAM is required, and its complete therapeutic potential also needs to be further investigated.

## Supporting information

S1 Fig The effect of different concentrations of TGF-β1 on cell viability (*** indicates p < 0.001, ns: no significant. All the different concentration groups were compared with the 0 group). (TIF)

S2 Fig The effect of different concentrations of PAM on the viability of IEC-6 cells (** indicates p < 0.01, ns: no significant. All the different concentration groups were compared with the 0 group). (TIF)

S1 Table Detailed scoring criteria for DAI in animal experiments. (DOCX)

S2 Table Primer sequence list for experimental use (mouse). (DOCX)

S3 Table Primer sequence list for experimental use (rat). (DOCX)

S1 File Animal WB. (ZIP)

S2 File Cell WB. (ZIP)

S3 File Original data for the overall chart. (XLSX)

S4 File PCR and data of scratches. (ZIP)

S5 File Response experiment WB. (ZIP)

S6 File S1_raw_images. (PDF)
