# Supplementary figures and images for: Plasma-activated media inhibits epithelial-mesenchymal transition and ameliorates intestinal fibrosis through the PPARγ/TGF-β1/SMAD3 pathway (part 1 of 2)
Source: PLoS One. 2025 Oct 22;20(10):e0335225. doi: 10.1371/journal.pone.0335225 (PMC12543144; doi:10.1371/journal.pone.0335225)

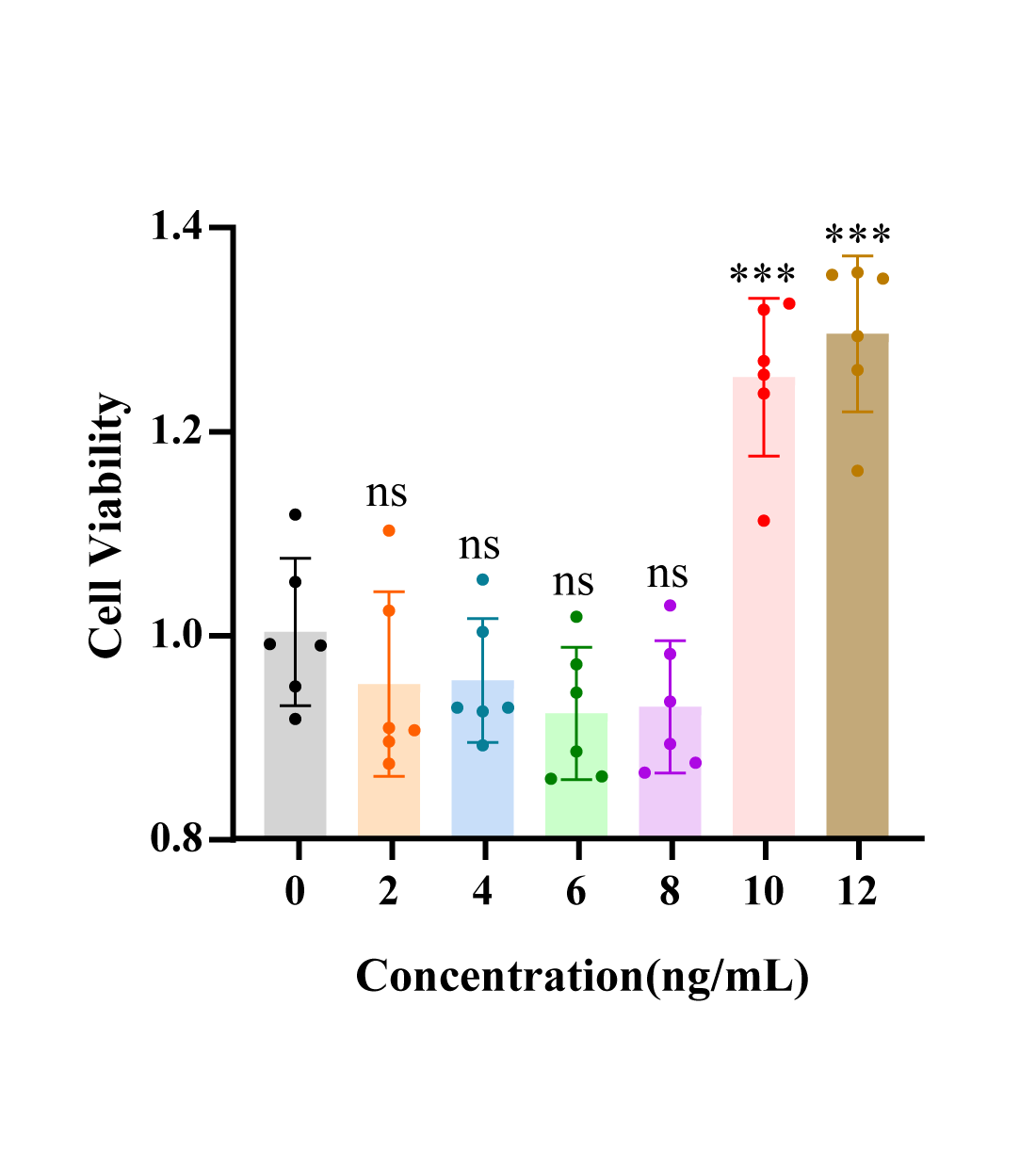

Supplement: S1 Fig — (TIF) [file pone.0335225.s001.tif]

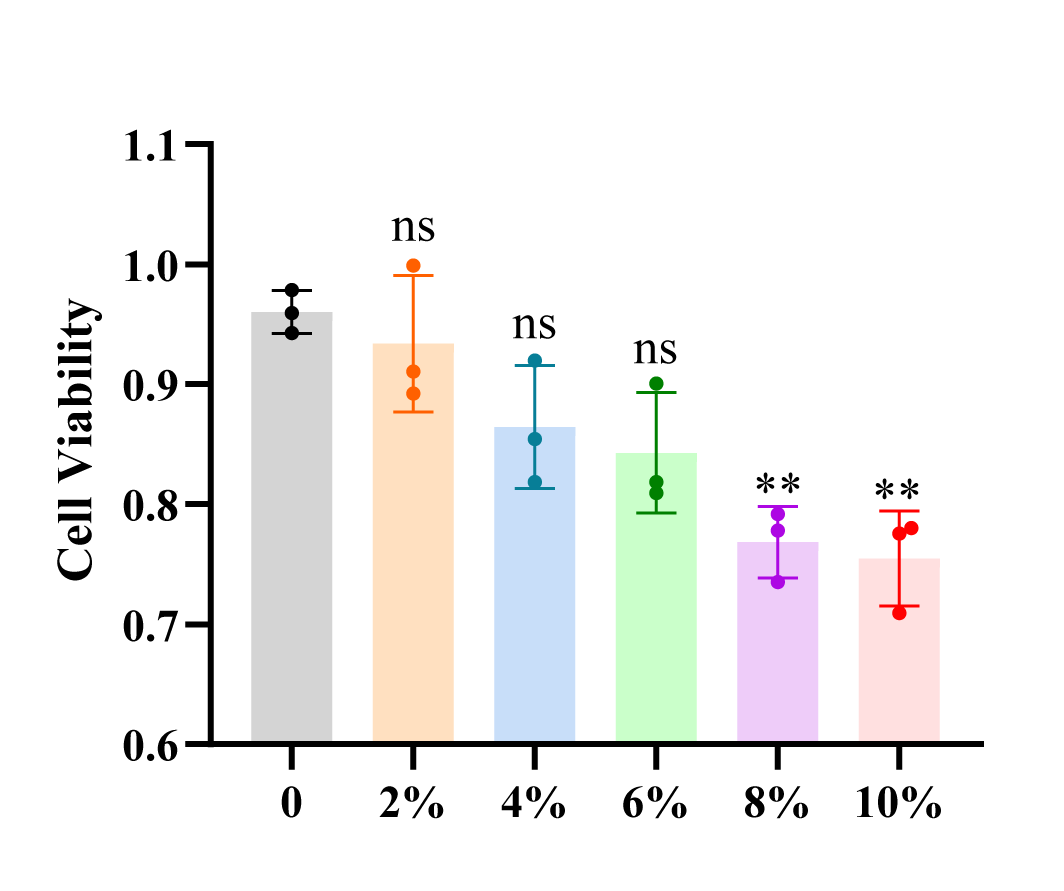

Supplement: S2 Fig — (TIF) [file pone.0335225.s002.tif]

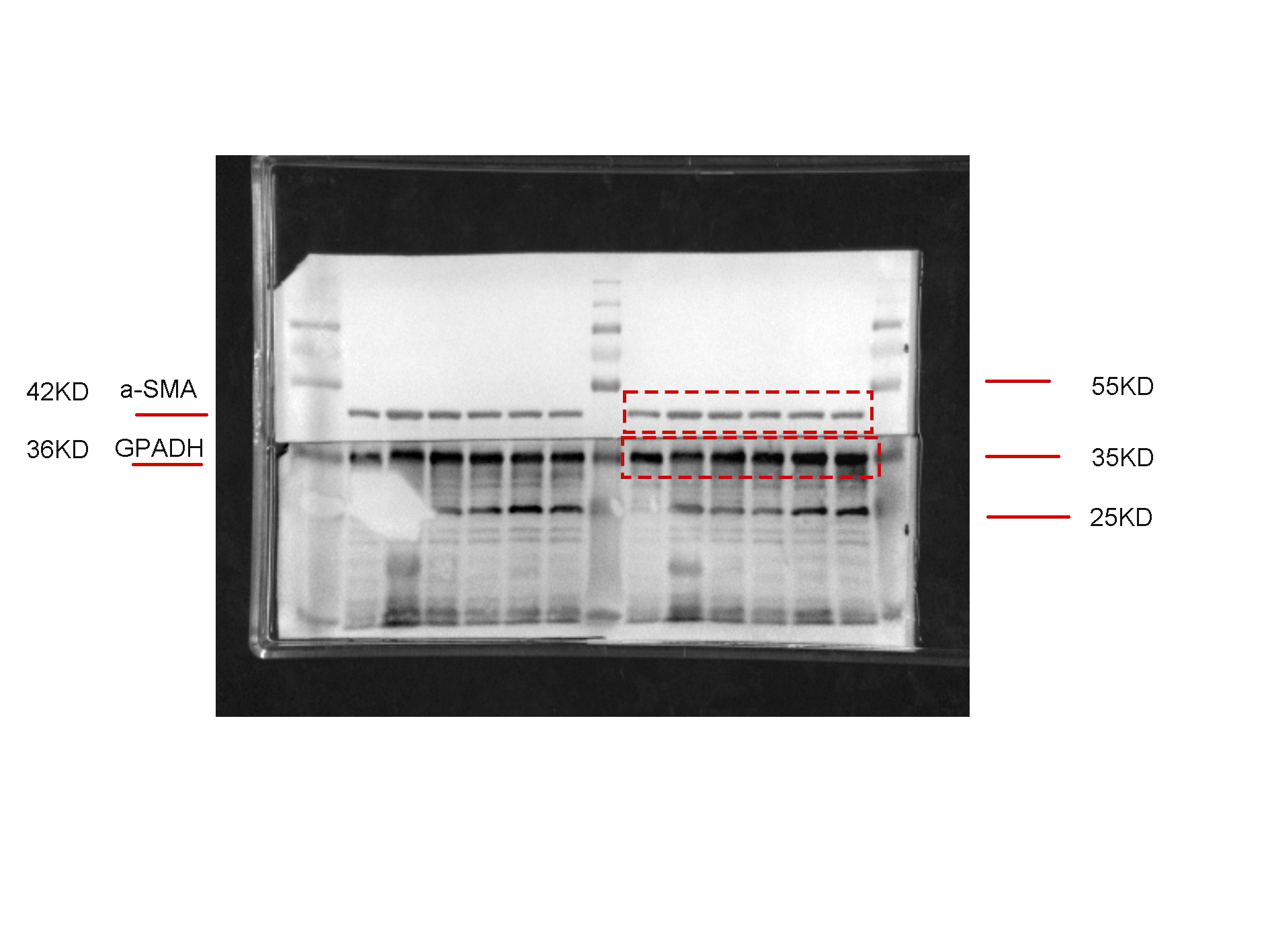

Supplement: S1 File — (ZIP) [file pone.0335225.s006.zip › Animal WB/A/animal a-SMA.tif]

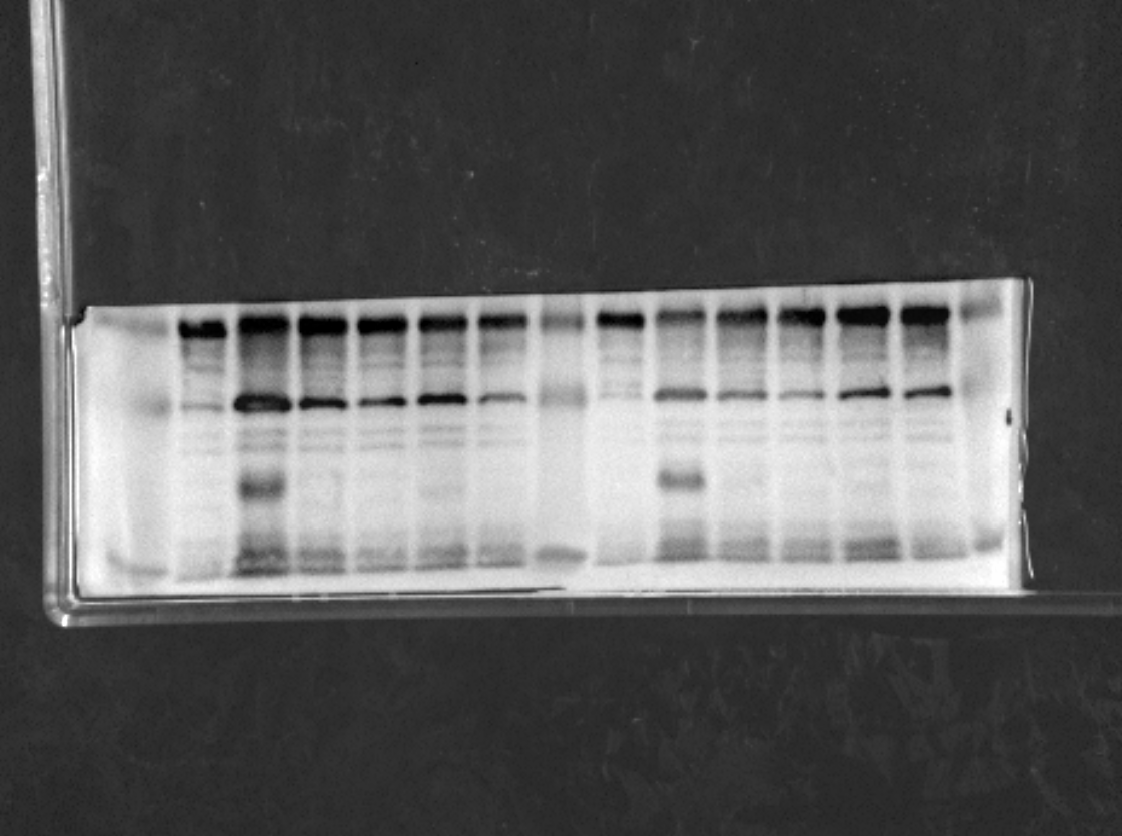

Supplement: S1 File — (ZIP) [file pone.0335225.s006.zip › Animal WB/A/gapdh 4_2s 1 hb.tif]

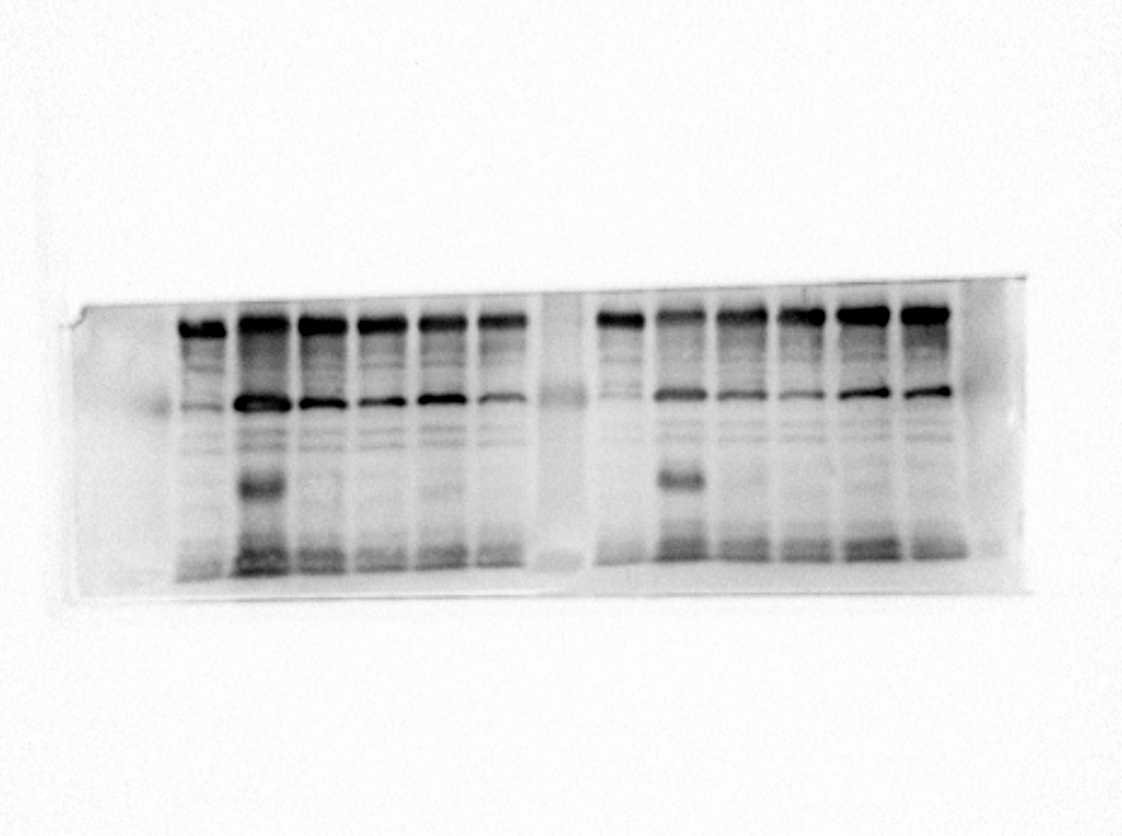

Supplement: S1 File — (ZIP) [file pone.0335225.s006.zip › Animal WB/A/gapdh 4_2s 1.tif]

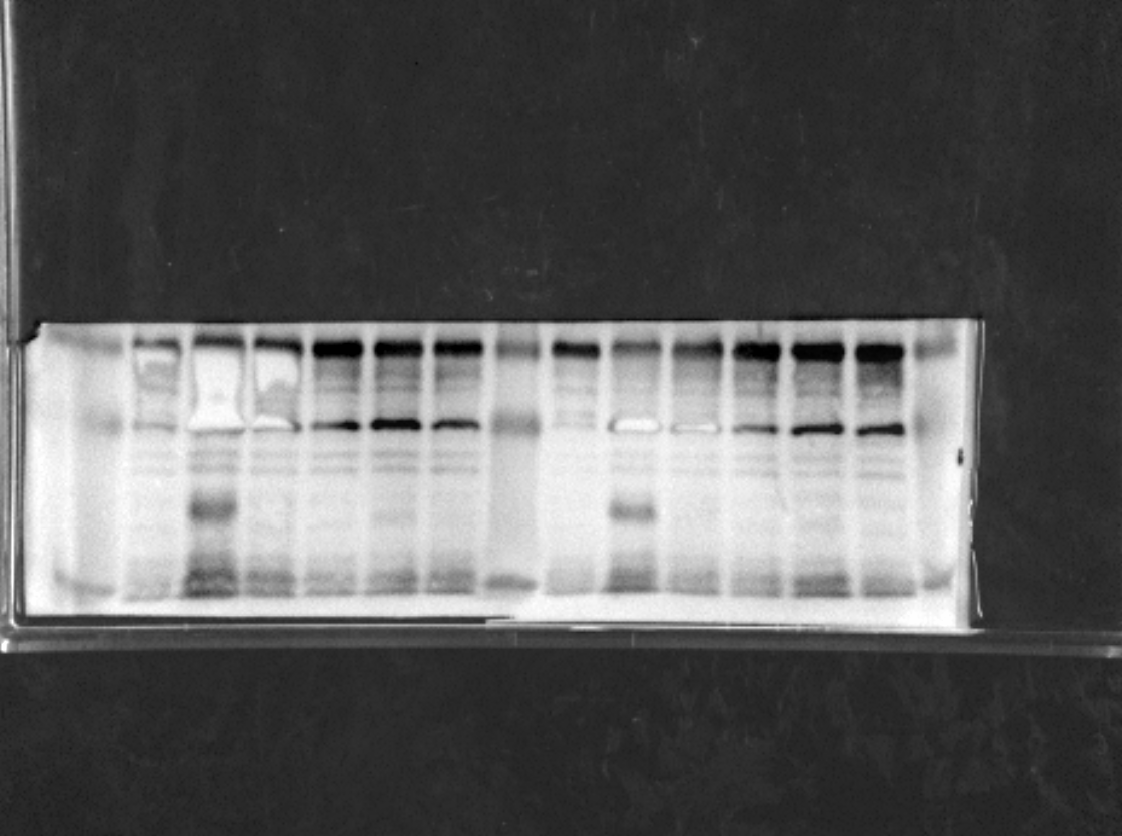

Supplement: S1 File — (ZIP) [file pone.0335225.s006.zip › Animal WB/A/gapdh 4_2s 1_1 hb.tif]

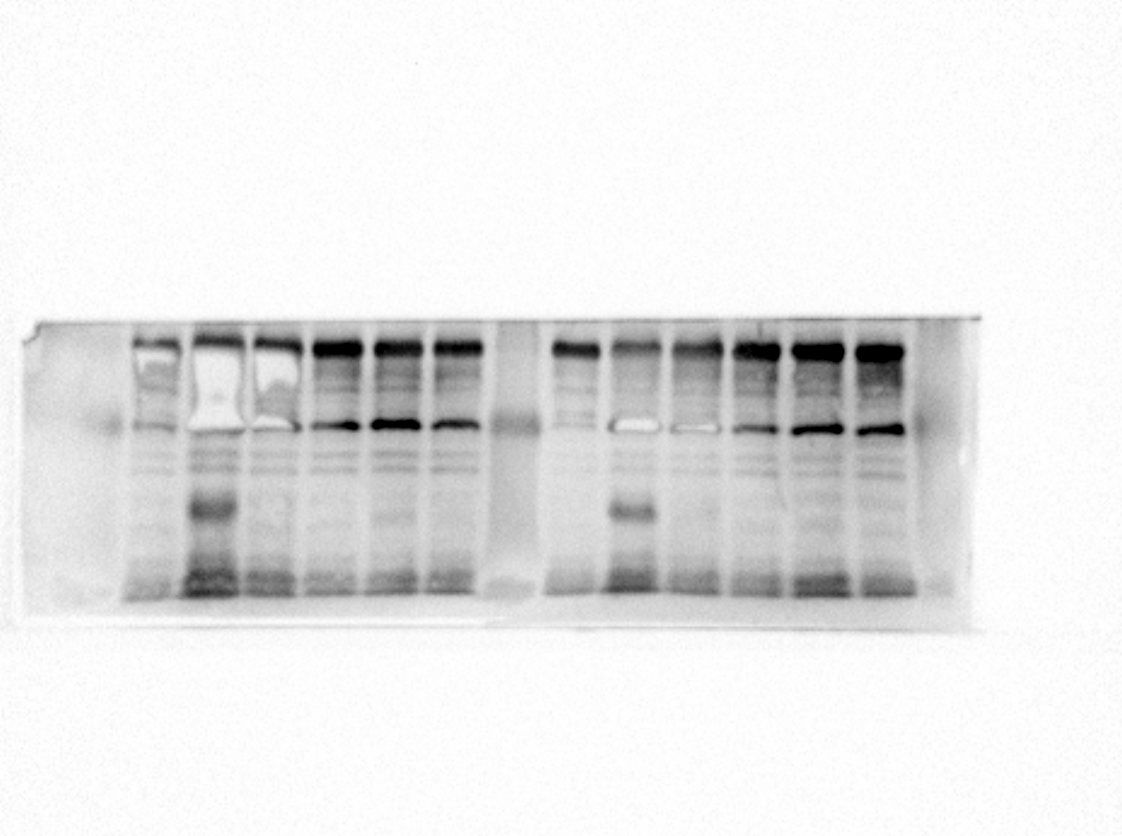

Supplement: S1 File — (ZIP) [file pone.0335225.s006.zip › Animal WB/A/gapdh 4_2s 1_1.tif]

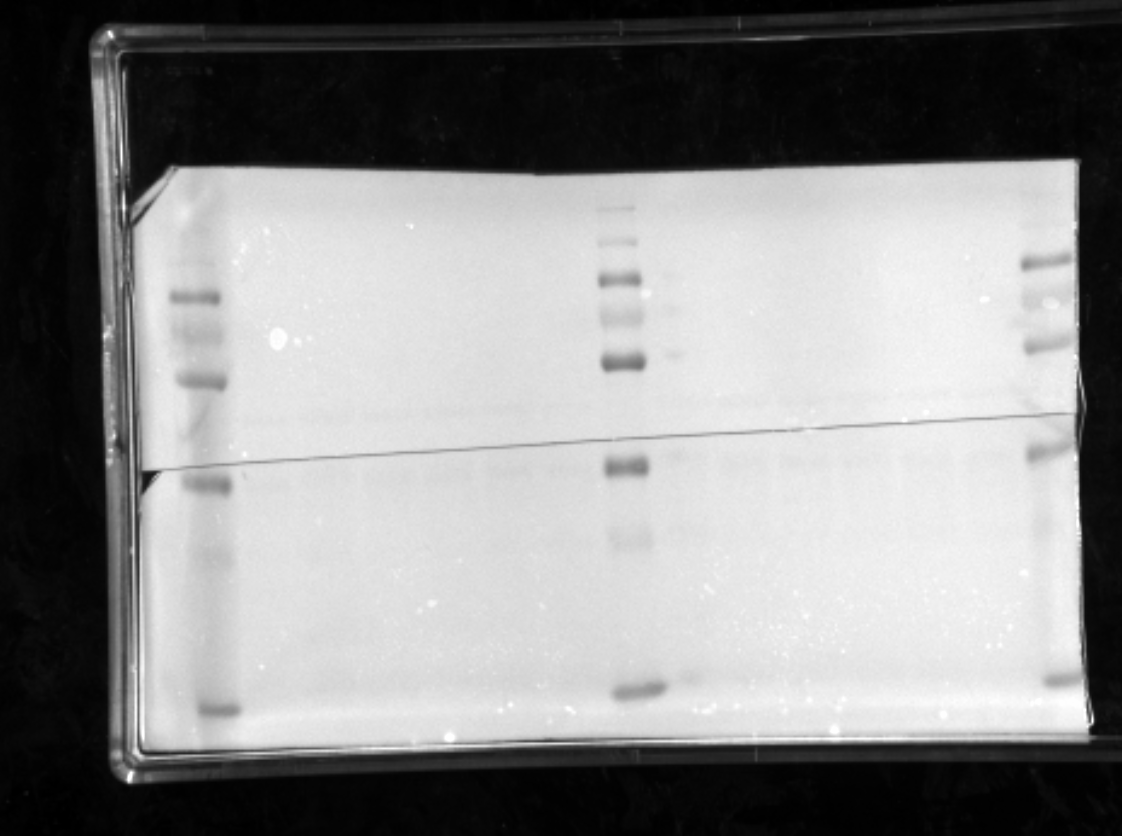

Supplement: S1 File — (ZIP) [file pone.0335225.s006.zip › Animal WB/A/maker 2 a.tif]

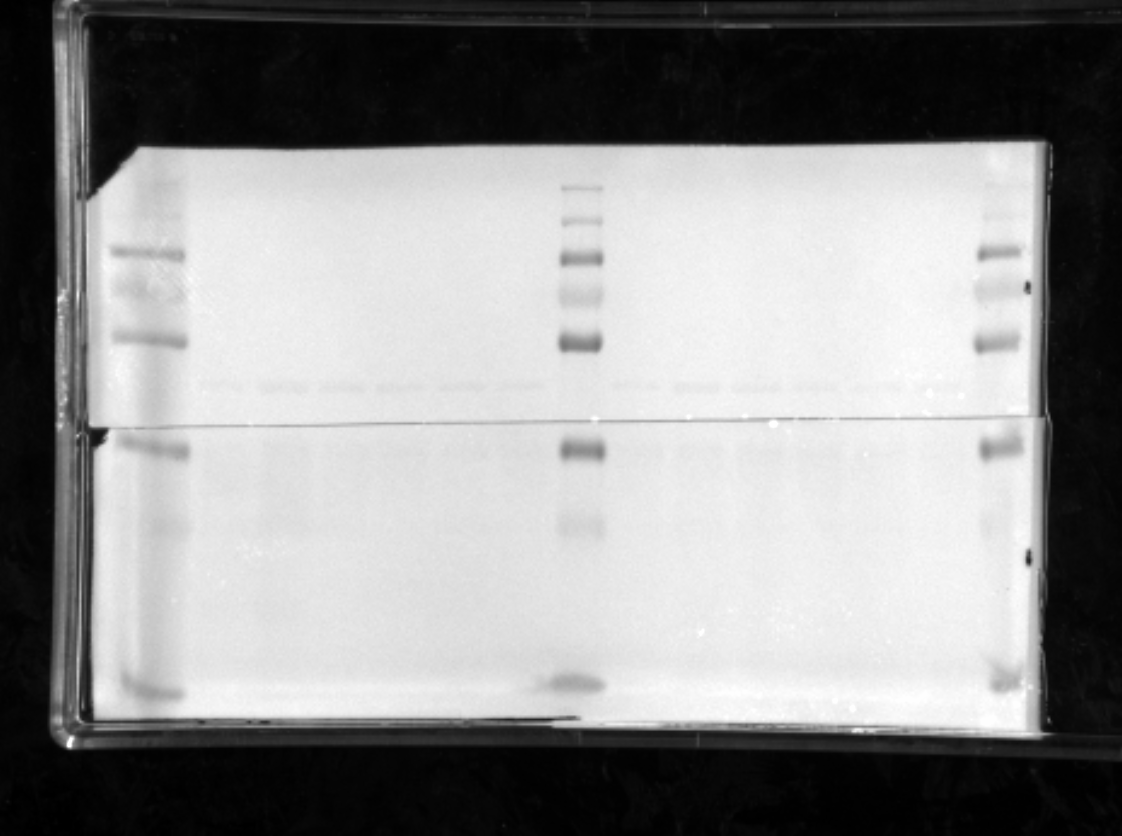

Supplement: S1 File — (ZIP) [file pone.0335225.s006.zip › Animal WB/A/图 maker 1 a.tif]

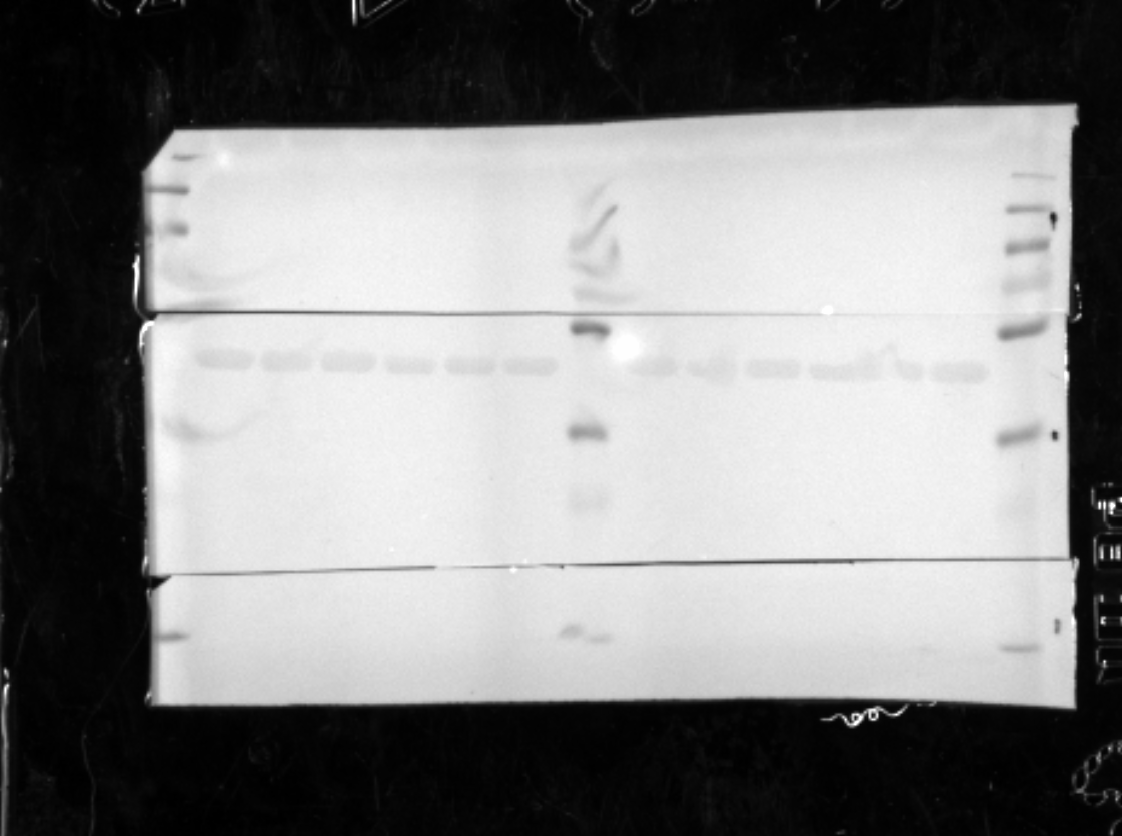

Supplement: S1 File — (ZIP) [file pone.0335225.s006.zip › Animal WB/COL/col1 maker.tif]

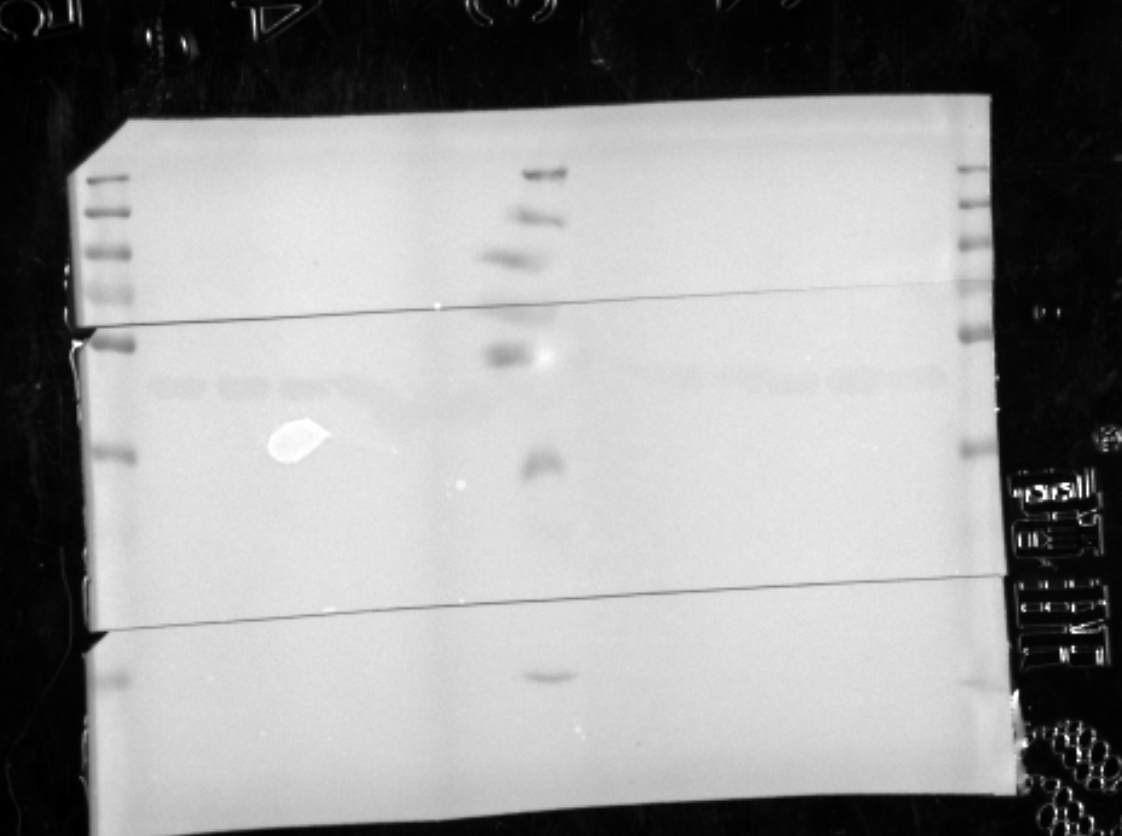

Supplement: S1 File — (ZIP) [file pone.0335225.s006.zip › Animal WB/COL/col3 maker 3.tif]

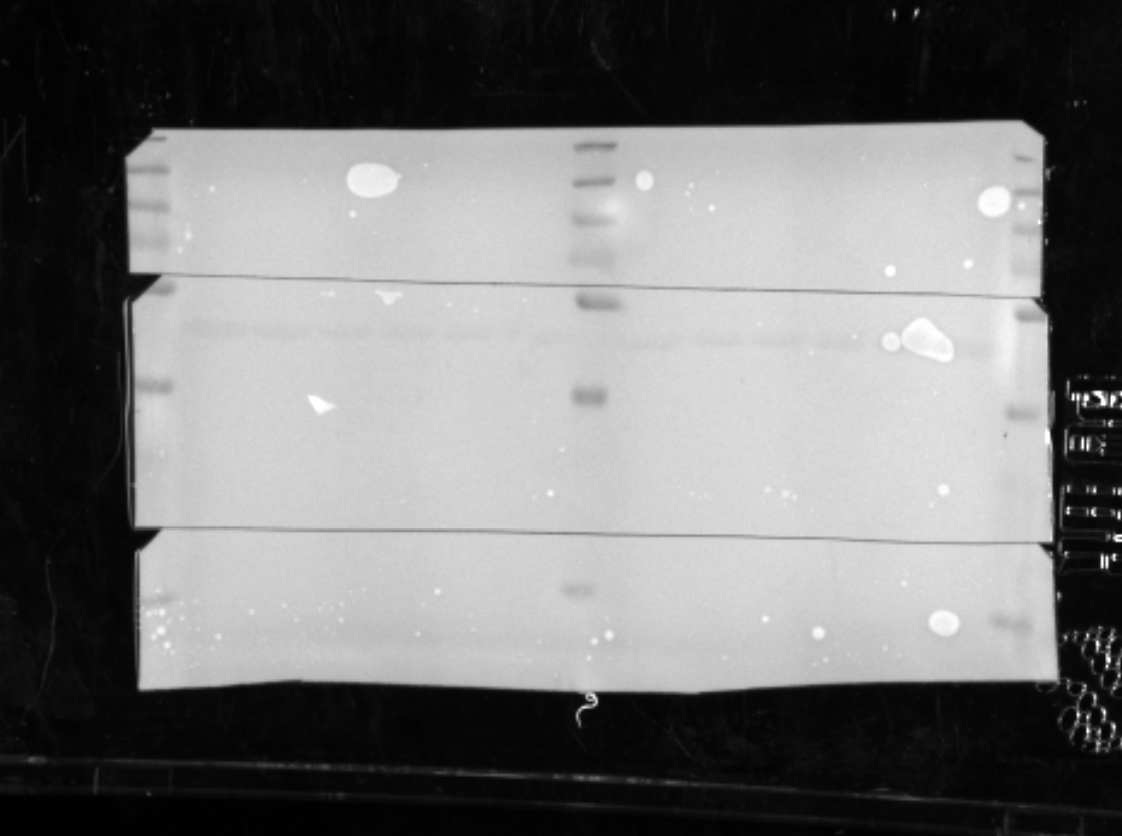

Supplement: S1 File — (ZIP) [file pone.0335225.s006.zip › Animal WB/COL/图 col2 maker2.tif]

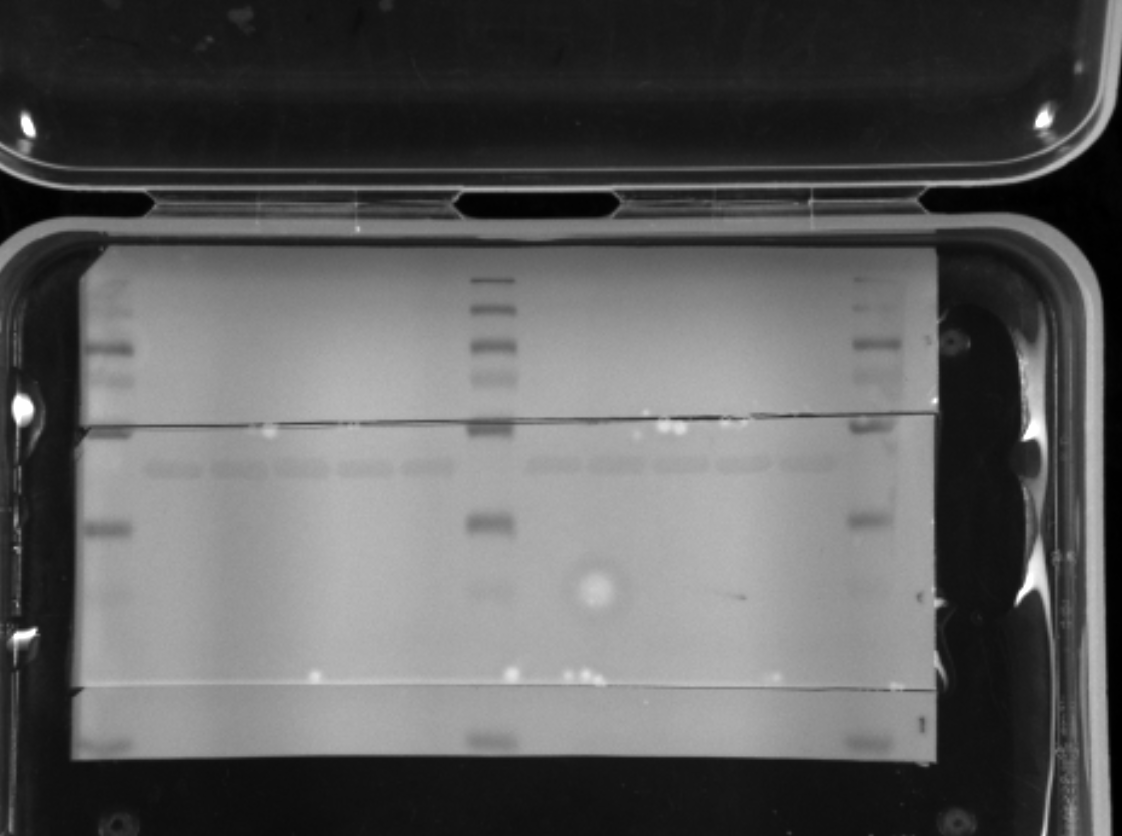

Supplement: S1 File — (ZIP) [file pone.0335225.s006.zip › Animal WB/E/E1/maker1.tif]

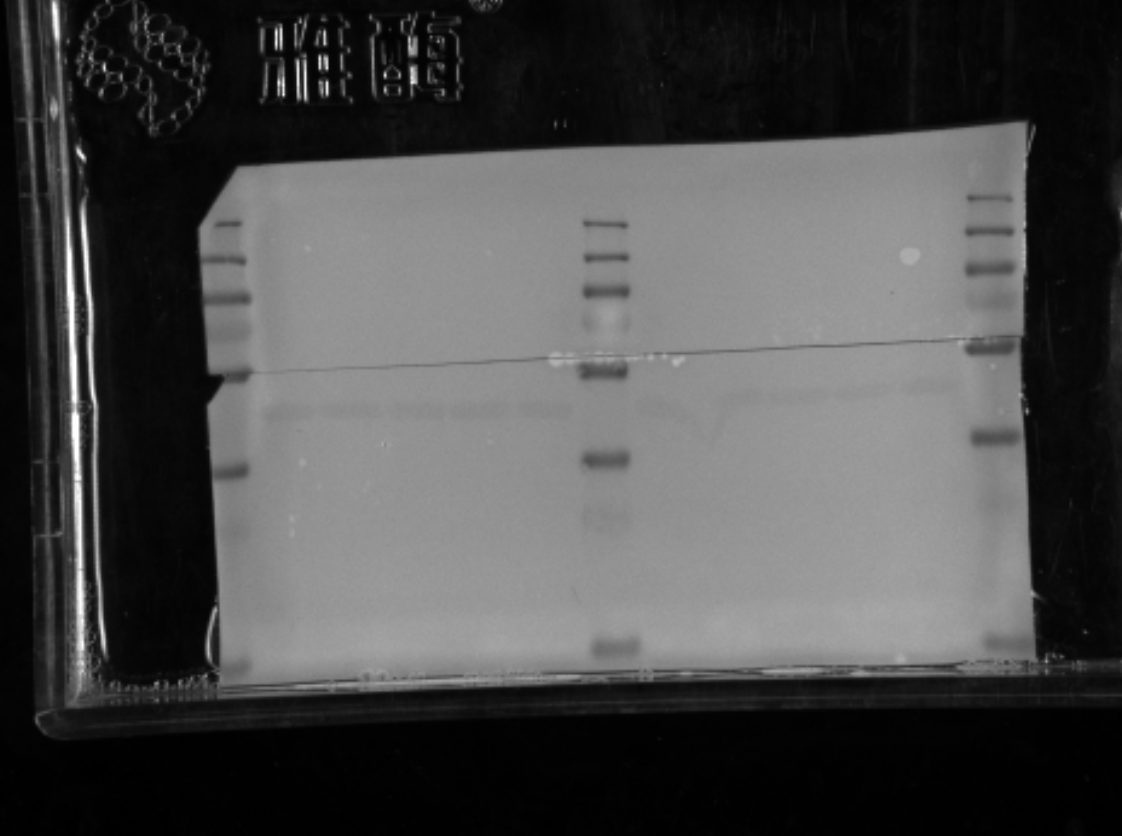

Supplement: S1 File — (ZIP) [file pone.0335225.s006.zip › Animal WB/E/E2/MAKER 1.tif]

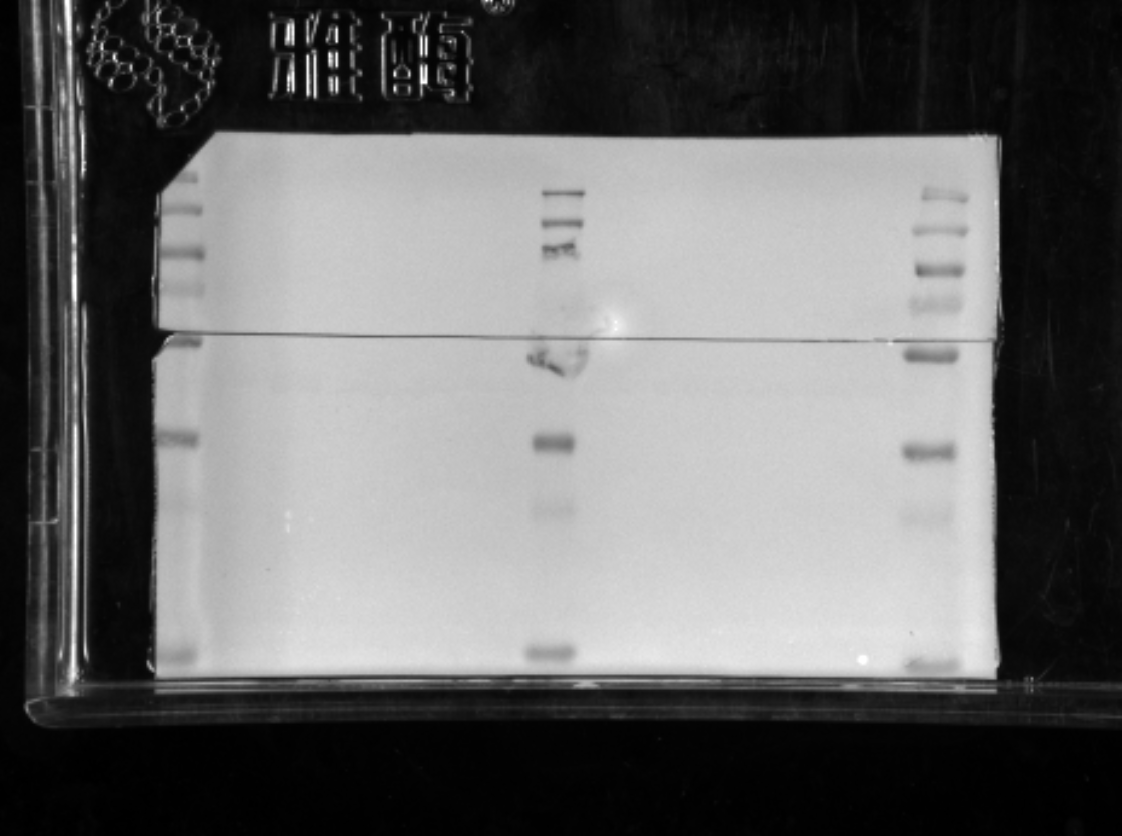

Supplement: S1 File — (ZIP) [file pone.0335225.s006.zip › Animal WB/E/E2/MAKER 2.tif]

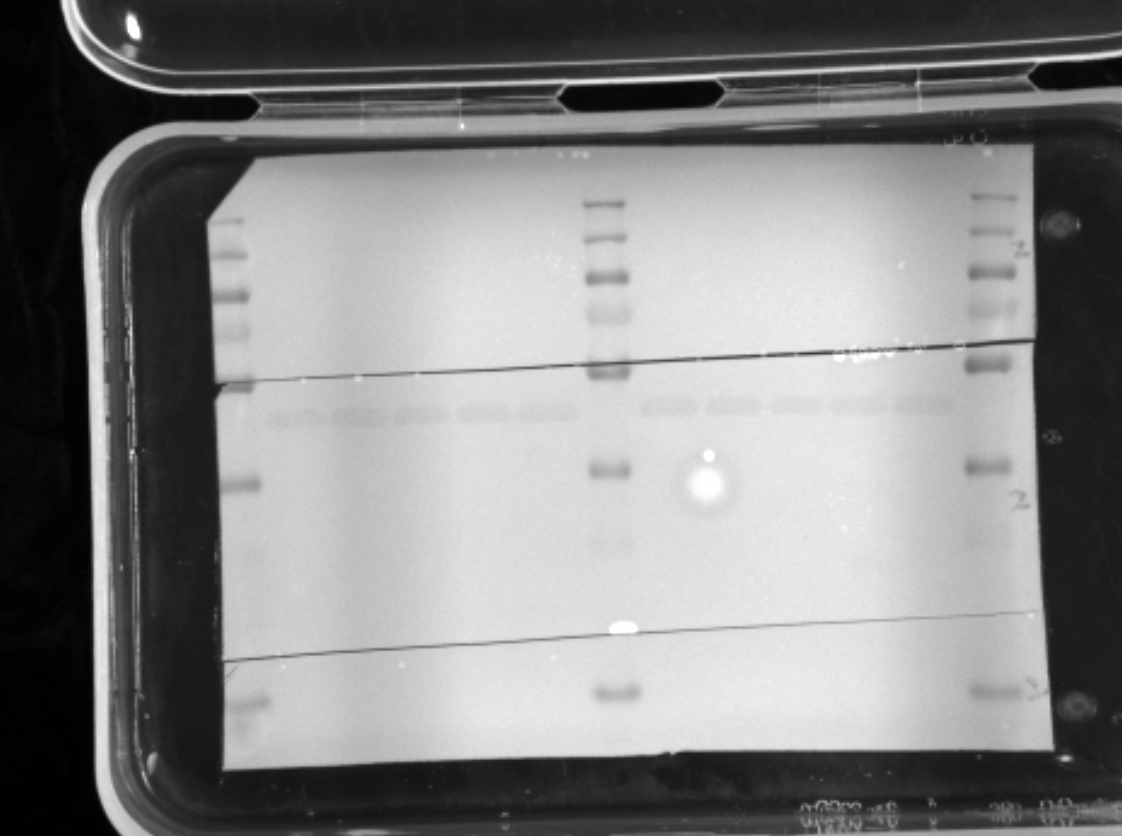

Supplement: S1 File — (ZIP) [file pone.0335225.s006.zip › Animal WB/N/N1/maker2.tif]

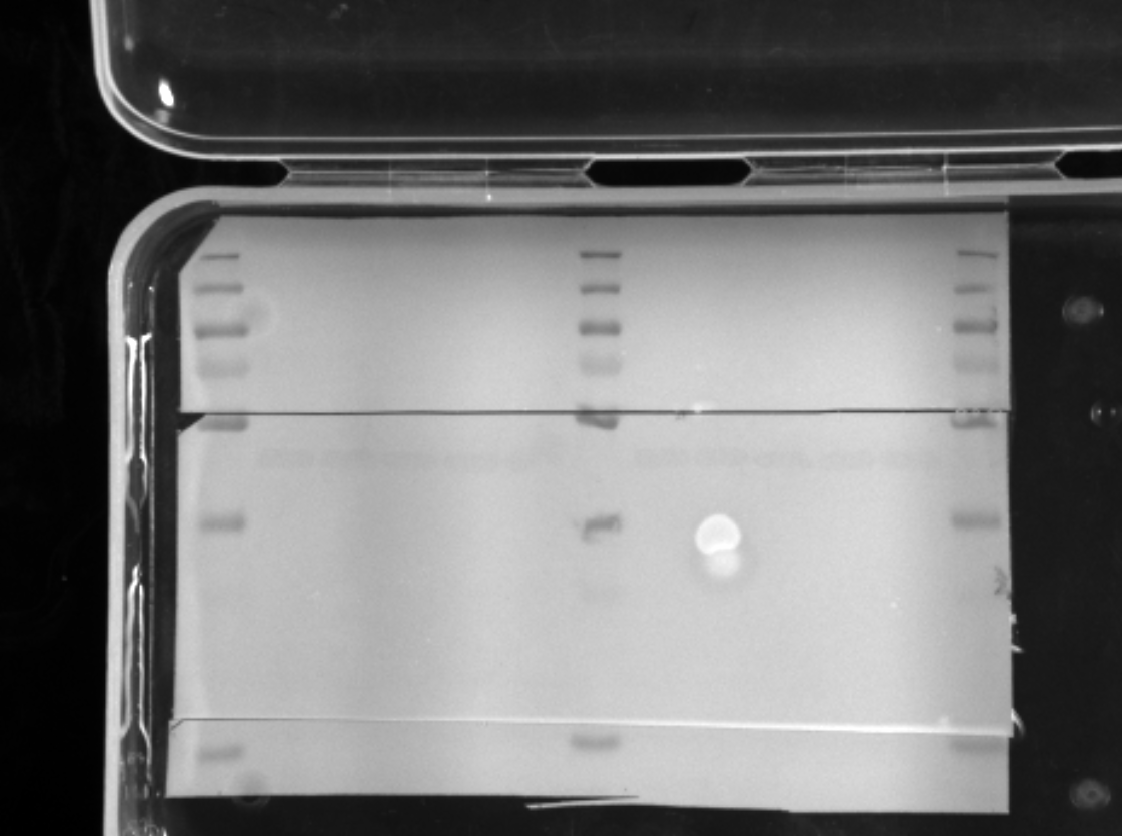

Supplement: S1 File — (ZIP) [file pone.0335225.s006.zip › Animal WB/N/N1/maker3.tif]

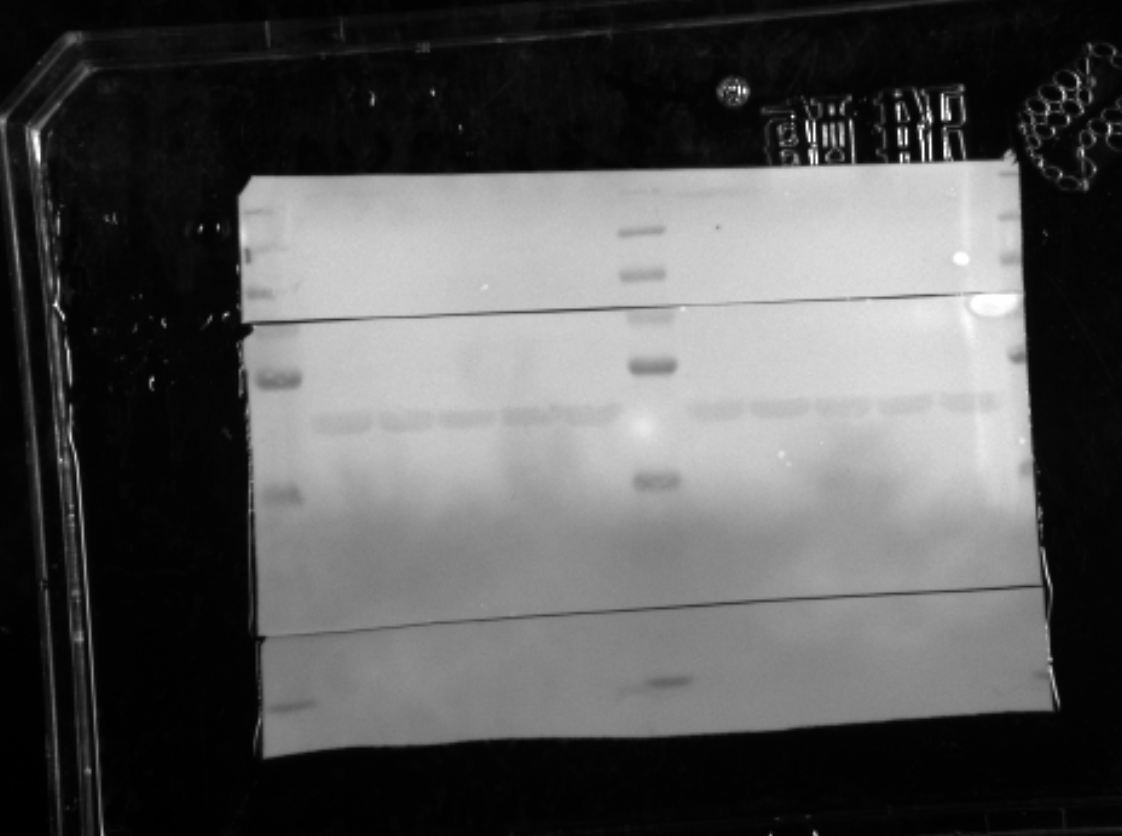

Supplement: S1 File — (ZIP) [file pone.0335225.s006.zip › Animal WB/N/N2/maker1_1.tif]

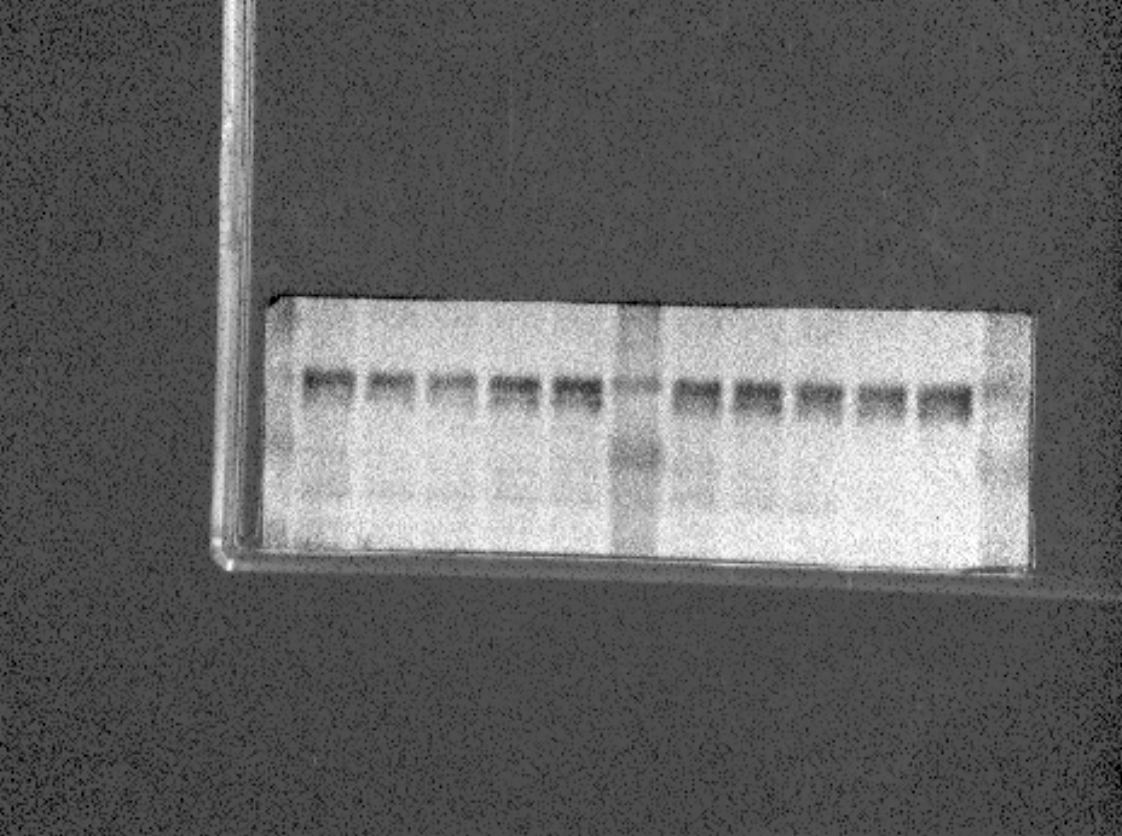

Supplement: S1 File — (ZIP) [file pone.0335225.s006.zip › Animal WB/PPAR/g 11-1430s 2 hb.tif]

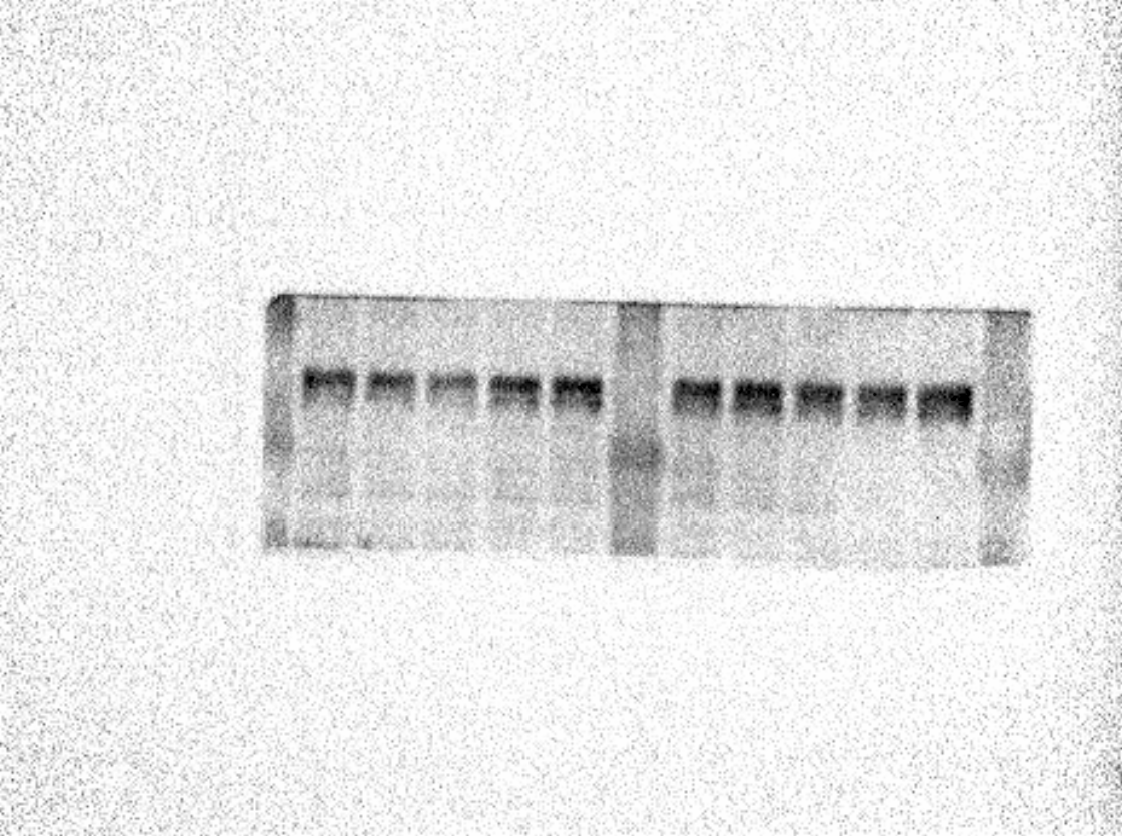

Supplement: S1 File — (ZIP) [file pone.0335225.s006.zip › Animal WB/PPAR/g 11-1430s 2.tif]

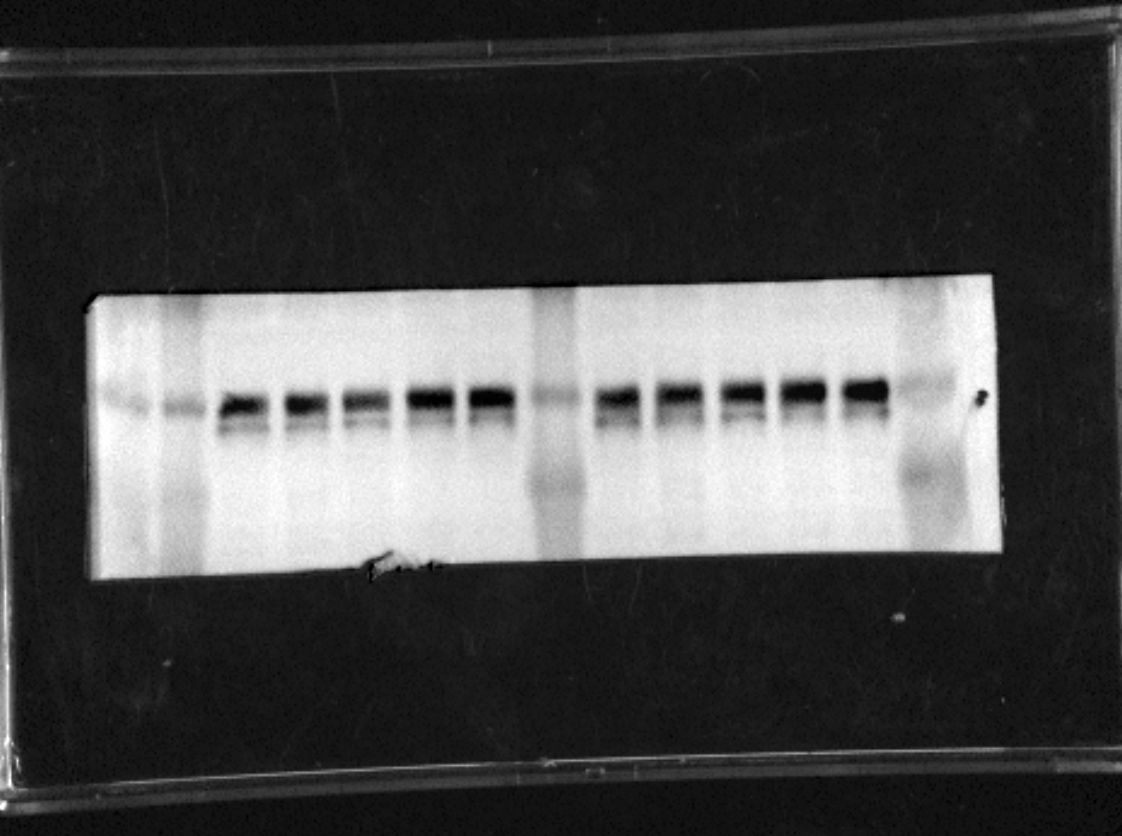

Supplement: S1 File — (ZIP) [file pone.0335225.s006.zip › Animal WB/PPAR/g 1_1 7_4s hb.tif]

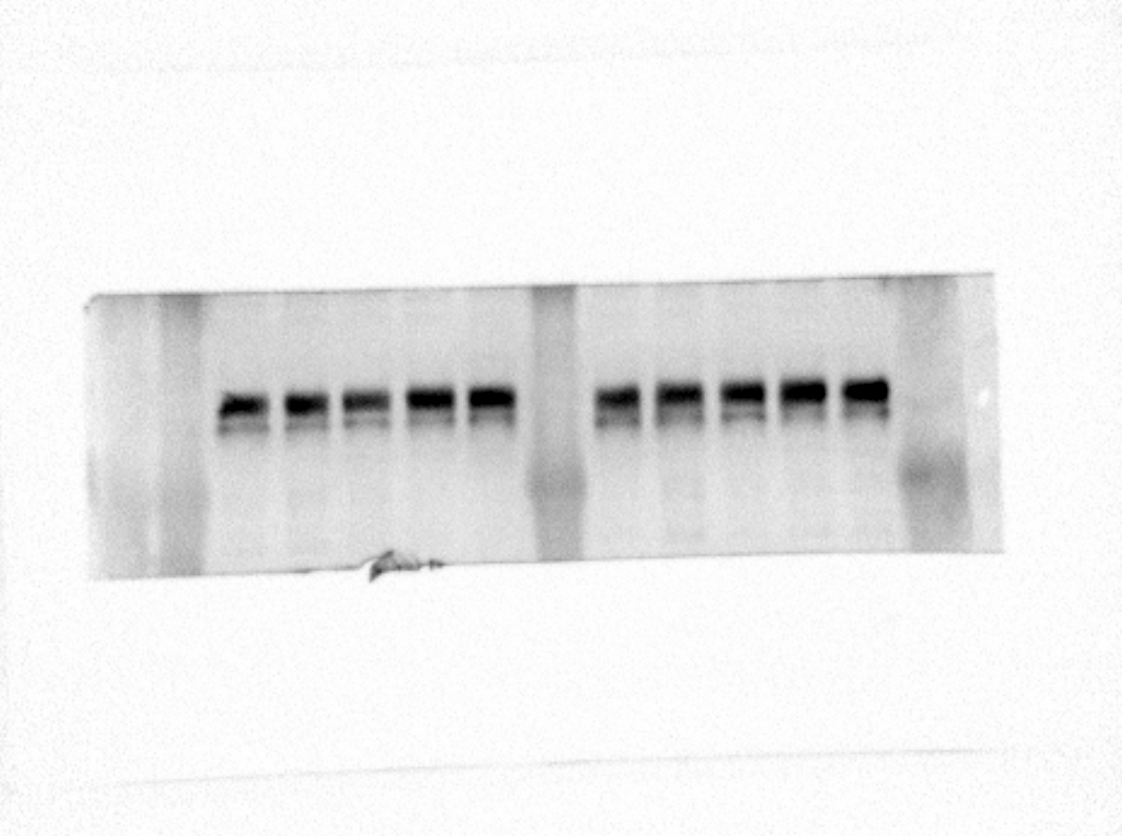

Supplement: S1 File — (ZIP) [file pone.0335225.s006.zip › Animal WB/PPAR/g 1_1 7_4s.tif]

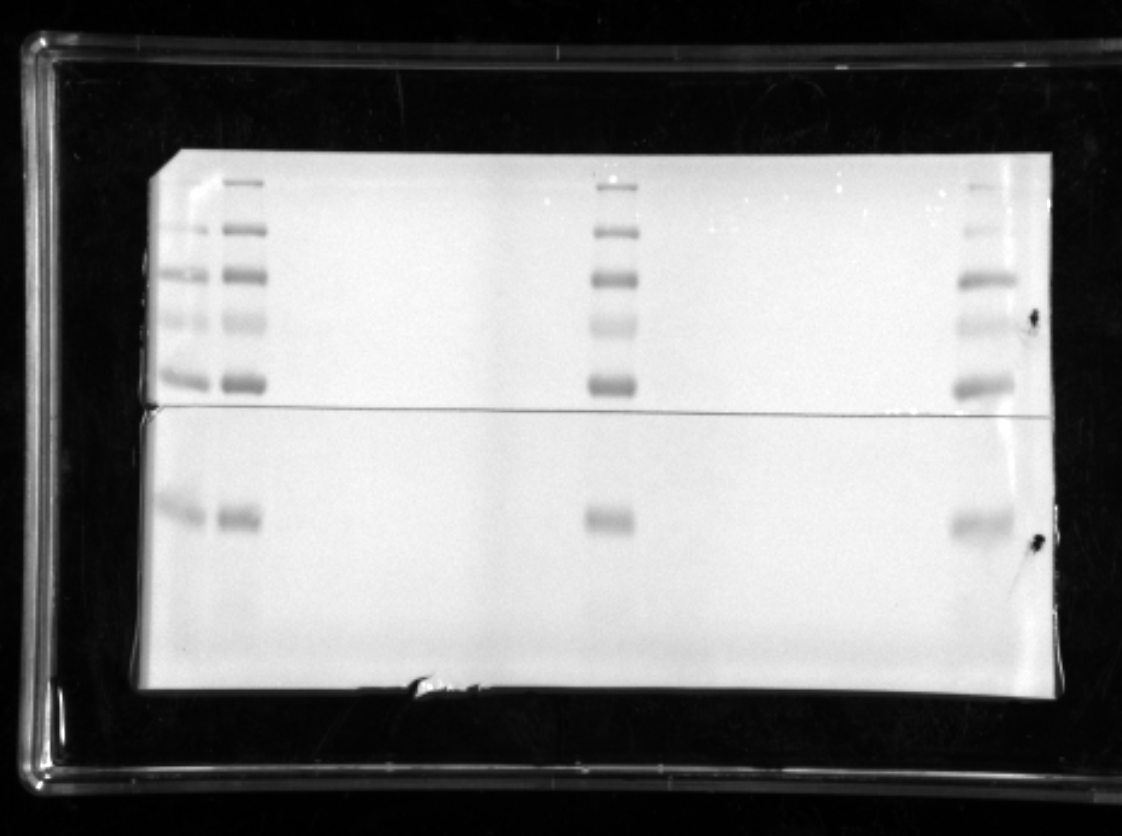

Supplement: S1 File — (ZIP) [file pone.0335225.s006.zip › Animal WB/PPAR/maker1.tif]

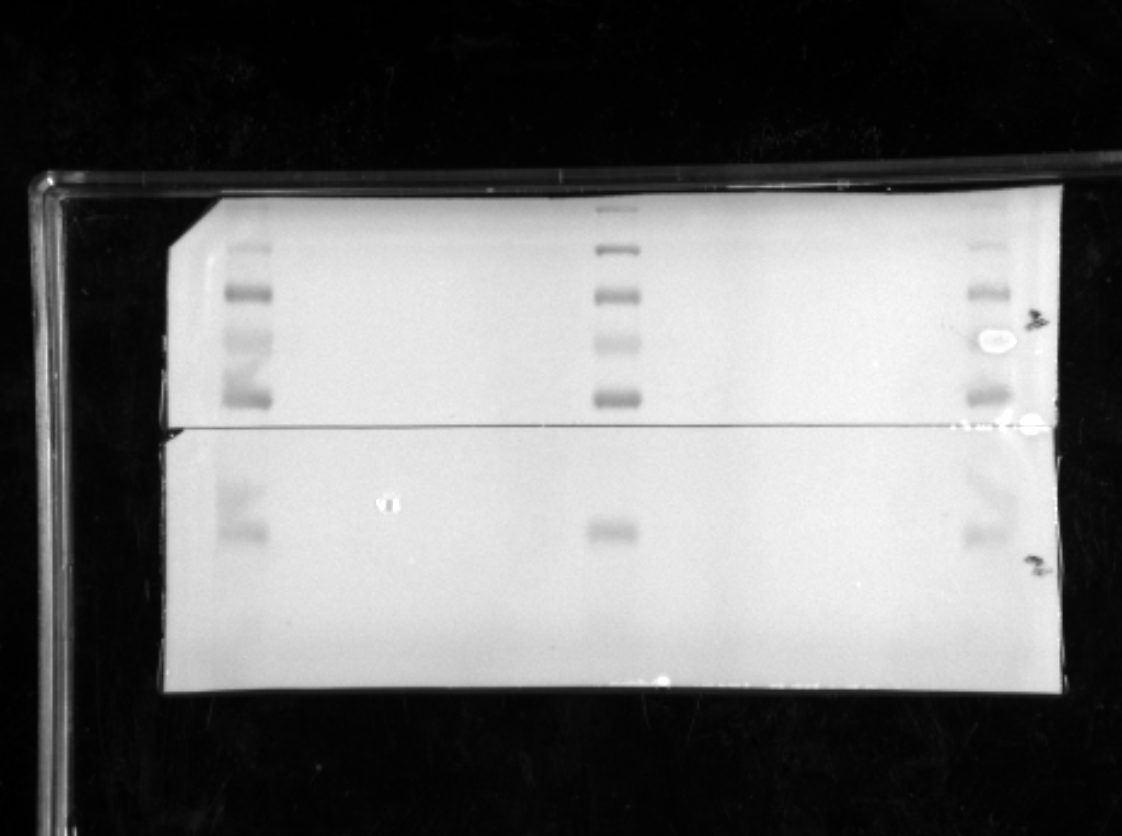

Supplement: S1 File — (ZIP) [file pone.0335225.s006.zip › Animal WB/PPAR/图 maker2.tif]

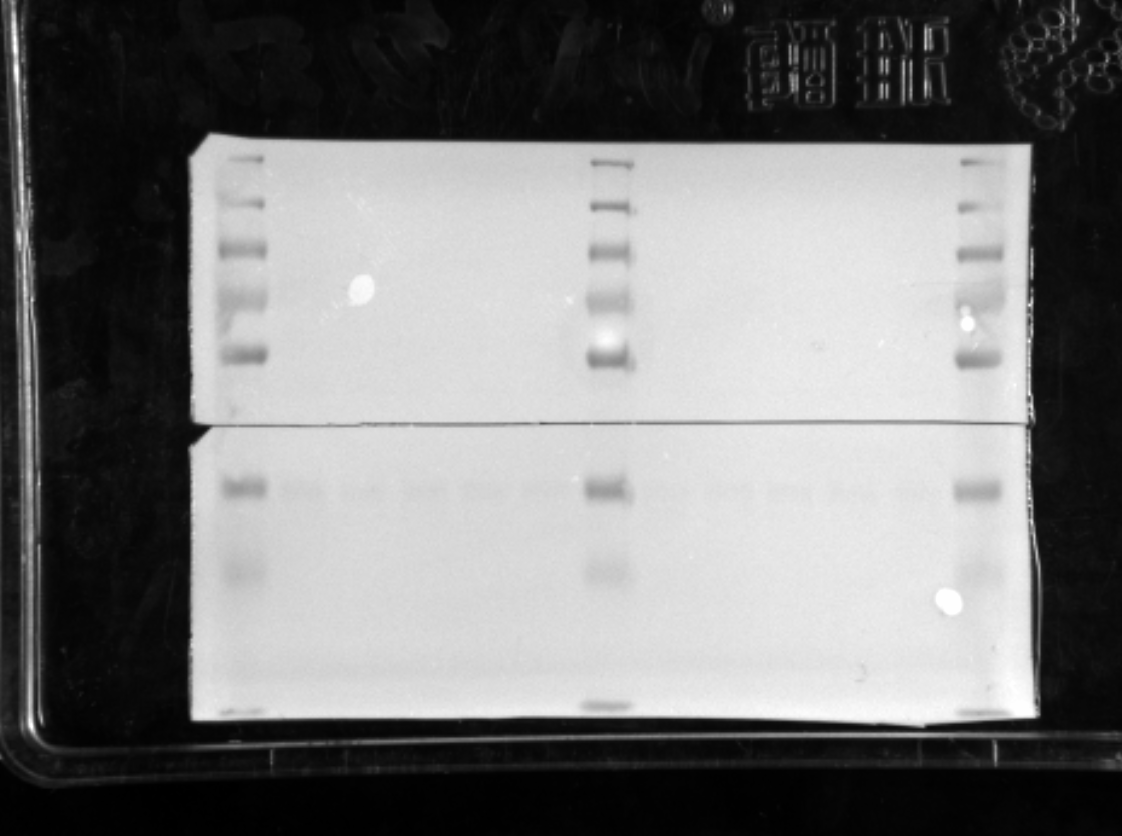

Supplement: S1 File — (ZIP) [file pone.0335225.s006.zip › Animal WB/Psmad/Smad1/maker 2.tif]

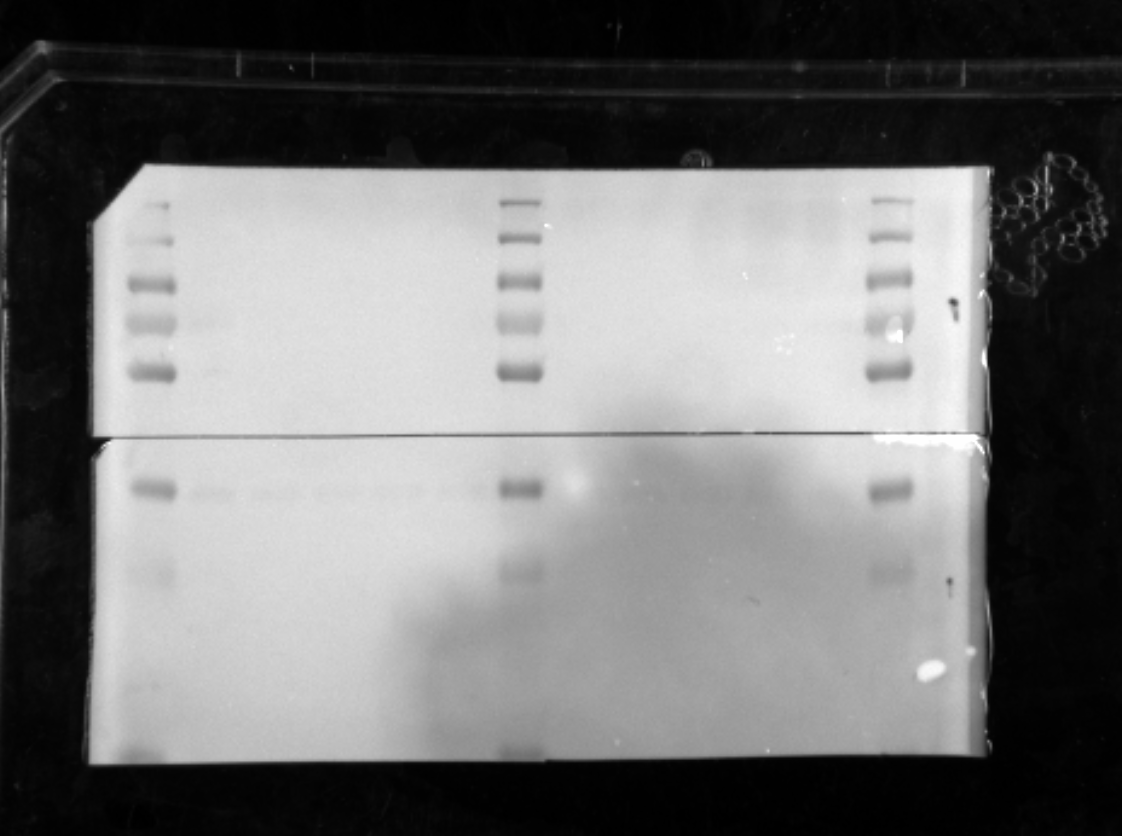

Supplement: S1 File — (ZIP) [file pone.0335225.s006.zip › Animal WB/Psmad/Smad1/图 maker 1.tif]

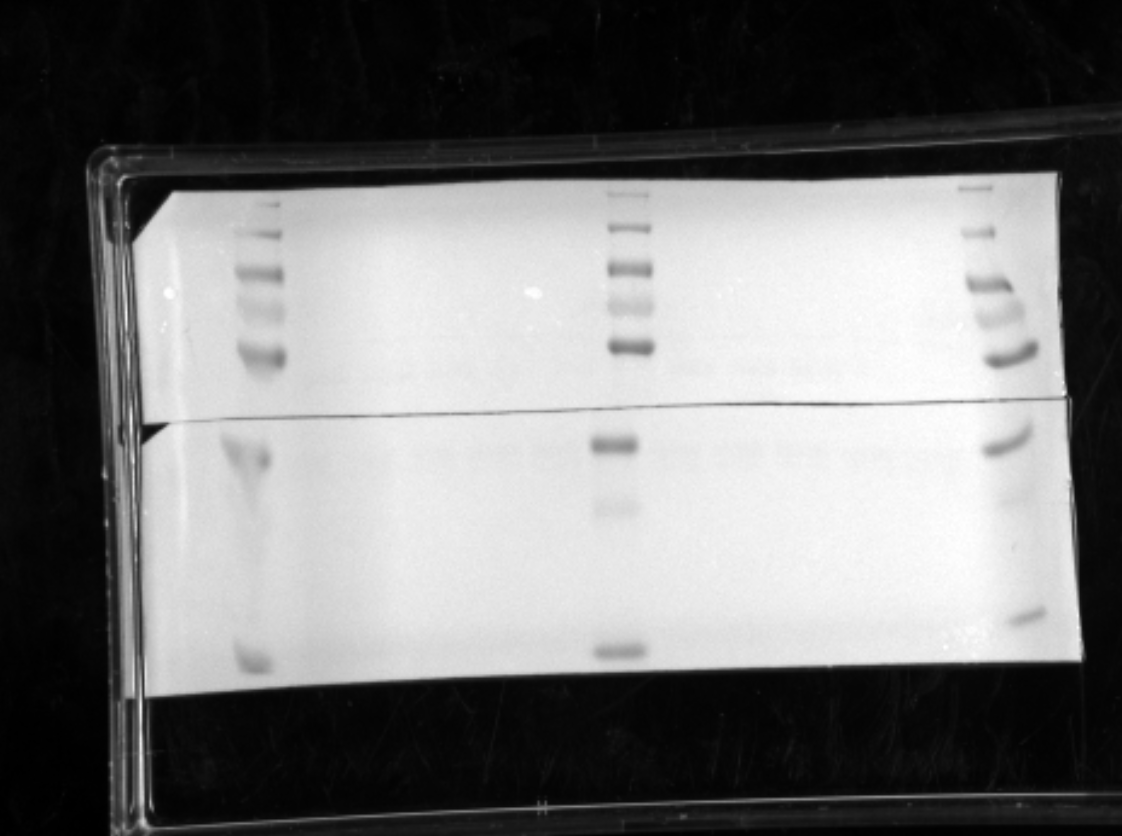

Supplement: S1 File — (ZIP) [file pone.0335225.s006.zip › Animal WB/Psmad/p-SMAD/maker 2.tif]

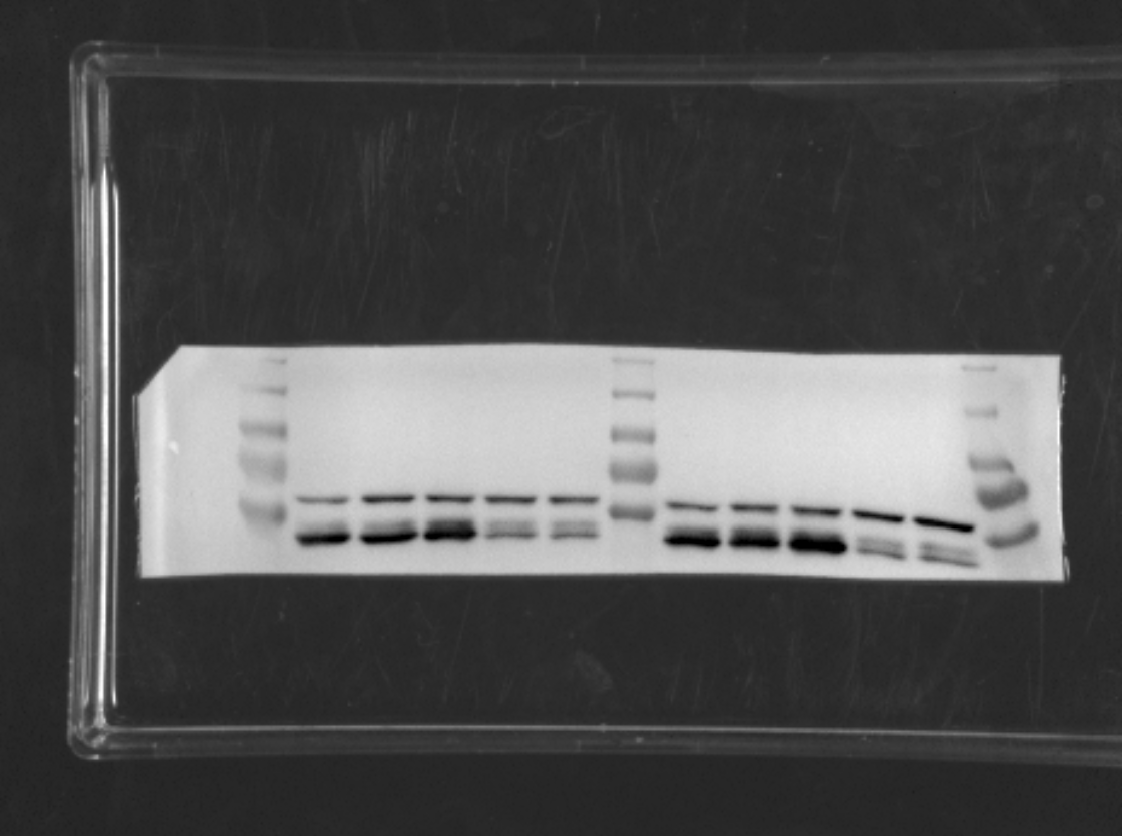

Supplement: S1 File — (ZIP) [file pone.0335225.s006.zip › Animal WB/Psmad/p-SMAD/sma 14_1s hb.tif]

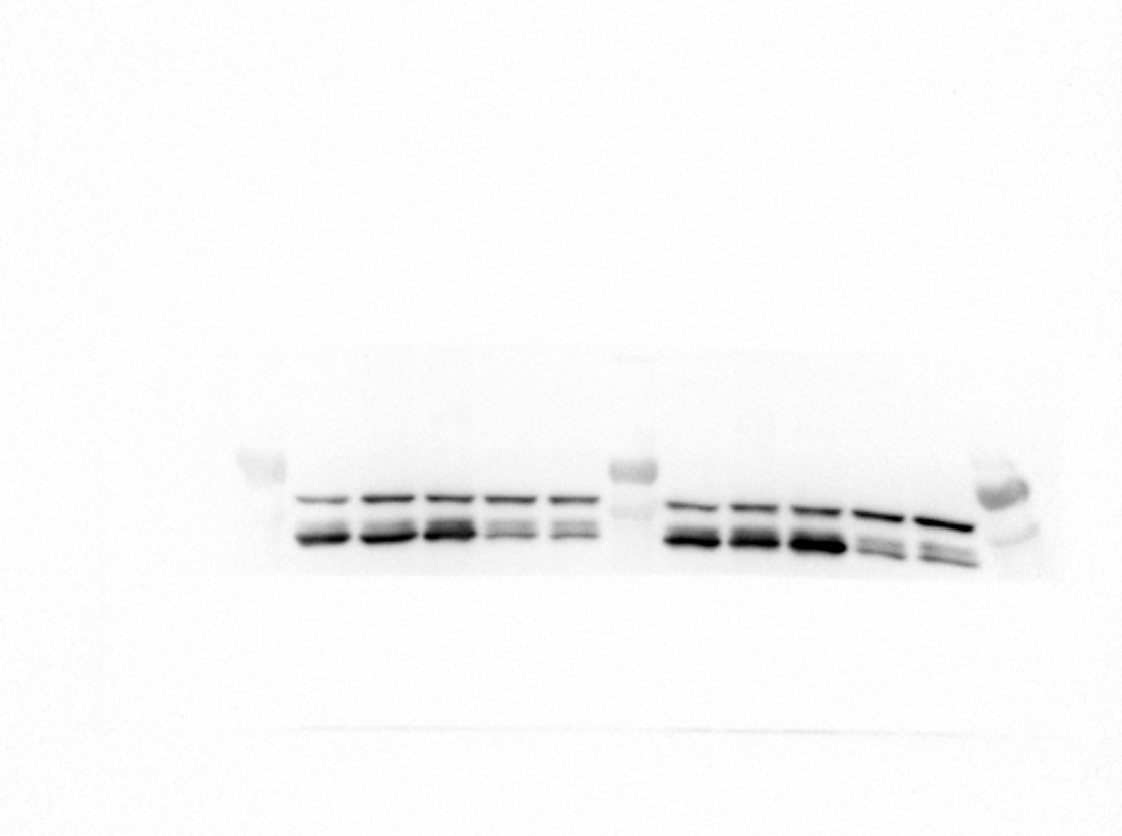

Supplement: S1 File — (ZIP) [file pone.0335225.s006.zip › Animal WB/Psmad/p-SMAD/sma 14_1s.tif]

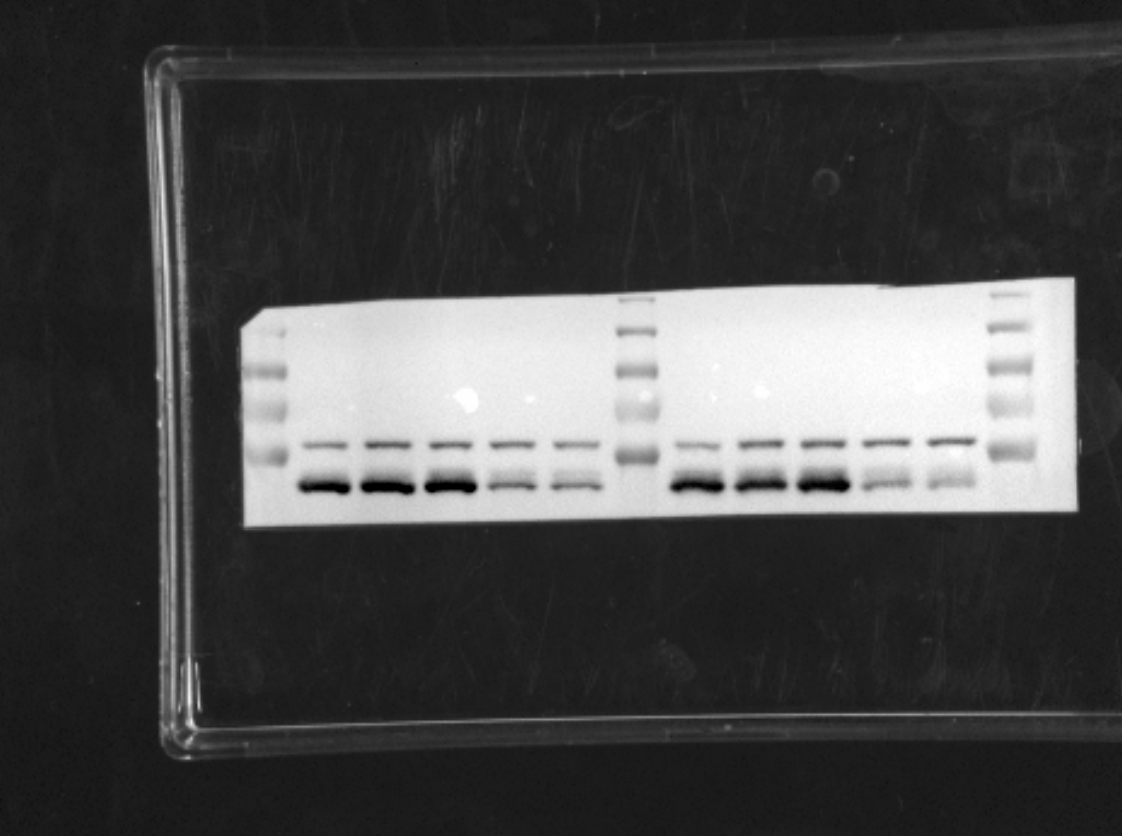

Supplement: S1 File — (ZIP) [file pone.0335225.s006.zip › Animal WB/Psmad/p-SMAD/sma3 7_6s hb.tif]

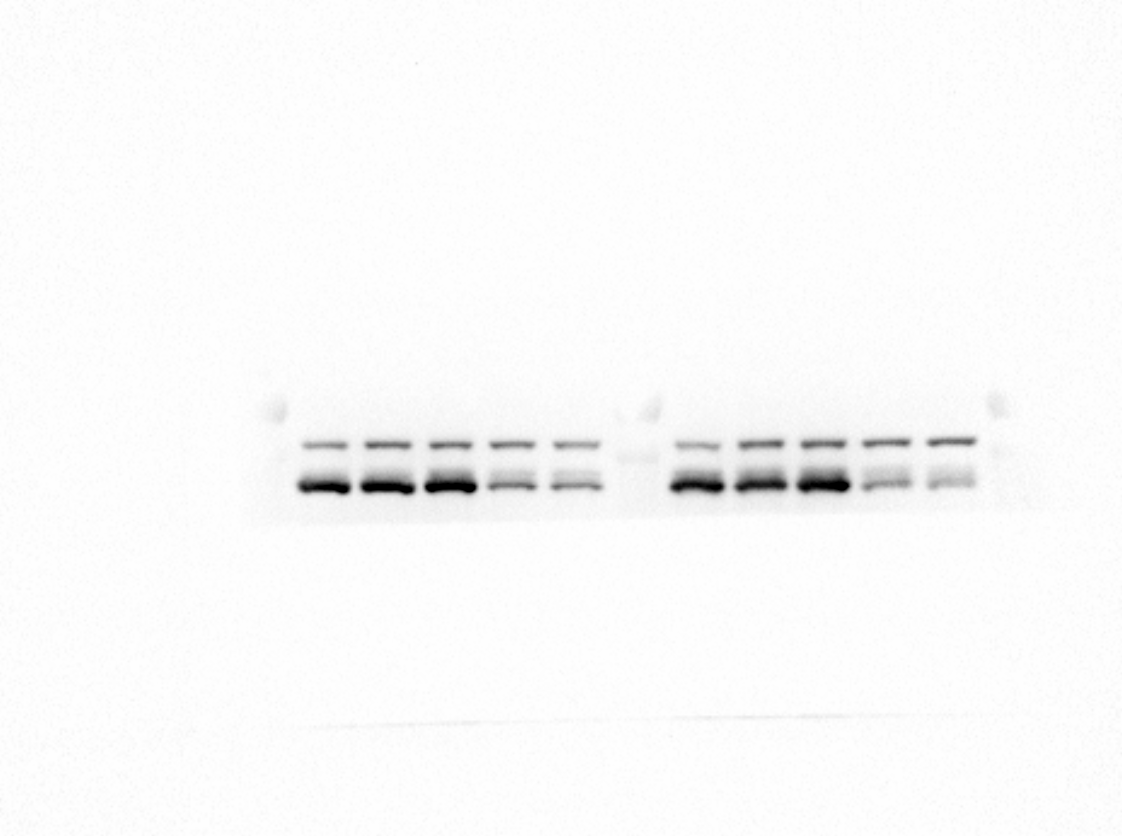

Supplement: S1 File — (ZIP) [file pone.0335225.s006.zip › Animal WB/Psmad/p-SMAD/sma3 7_6s.tif]

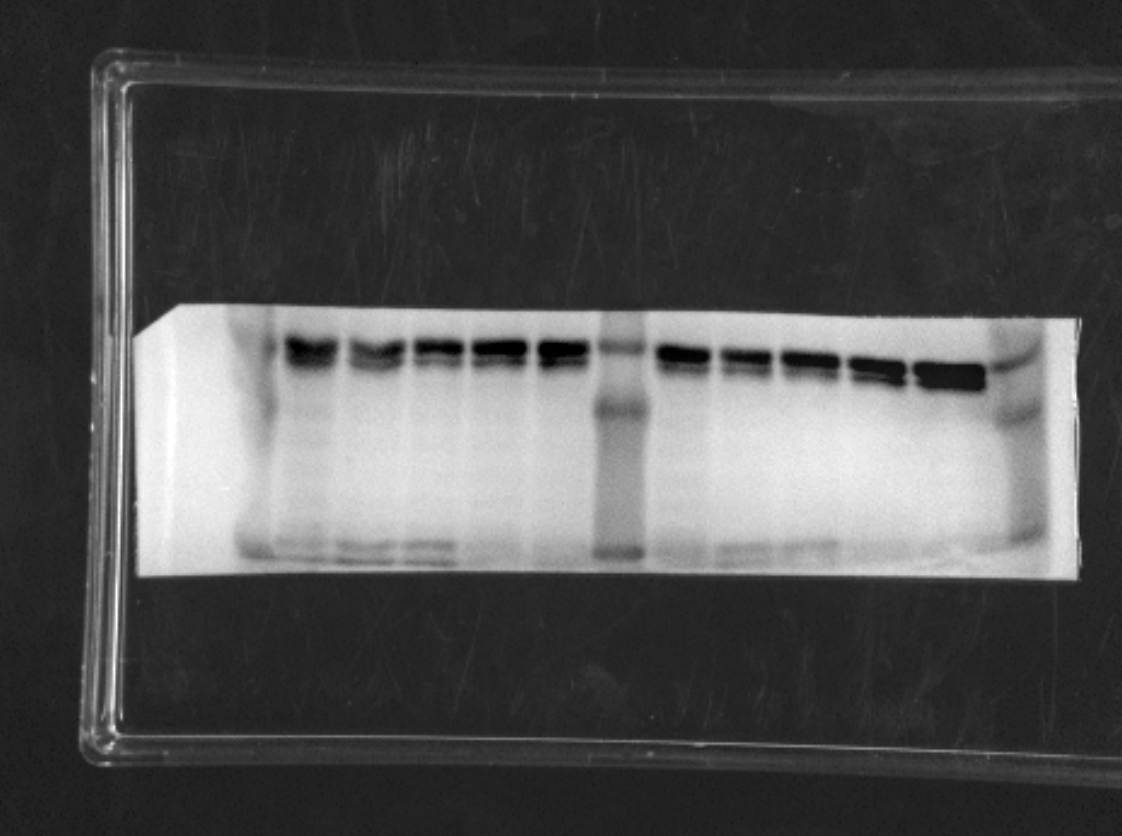

Supplement: S1 File — (ZIP) [file pone.0335225.s006.zip › Animal WB/Psmad/p-SMAD/yong.g 2_2 7_4s hb.tif]

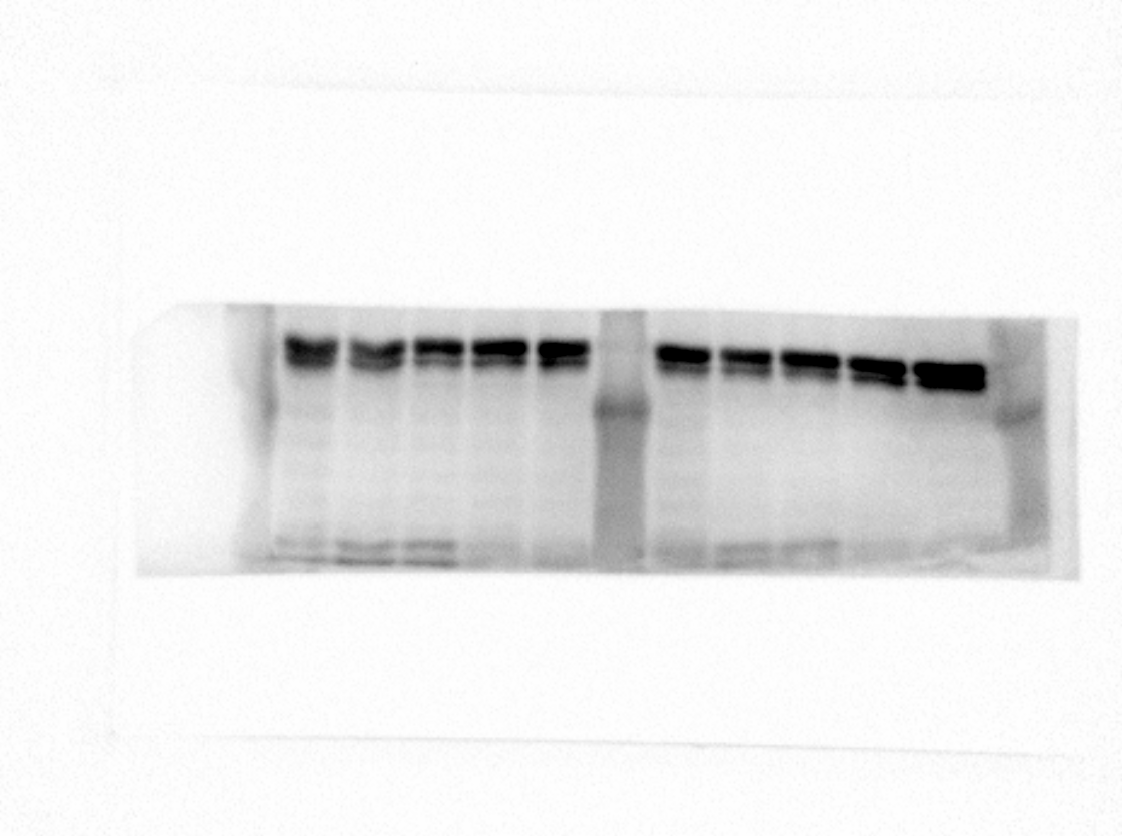

Supplement: S1 File — (ZIP) [file pone.0335225.s006.zip › Animal WB/Psmad/p-SMAD/yong.g 2_2 7_4s.tif]

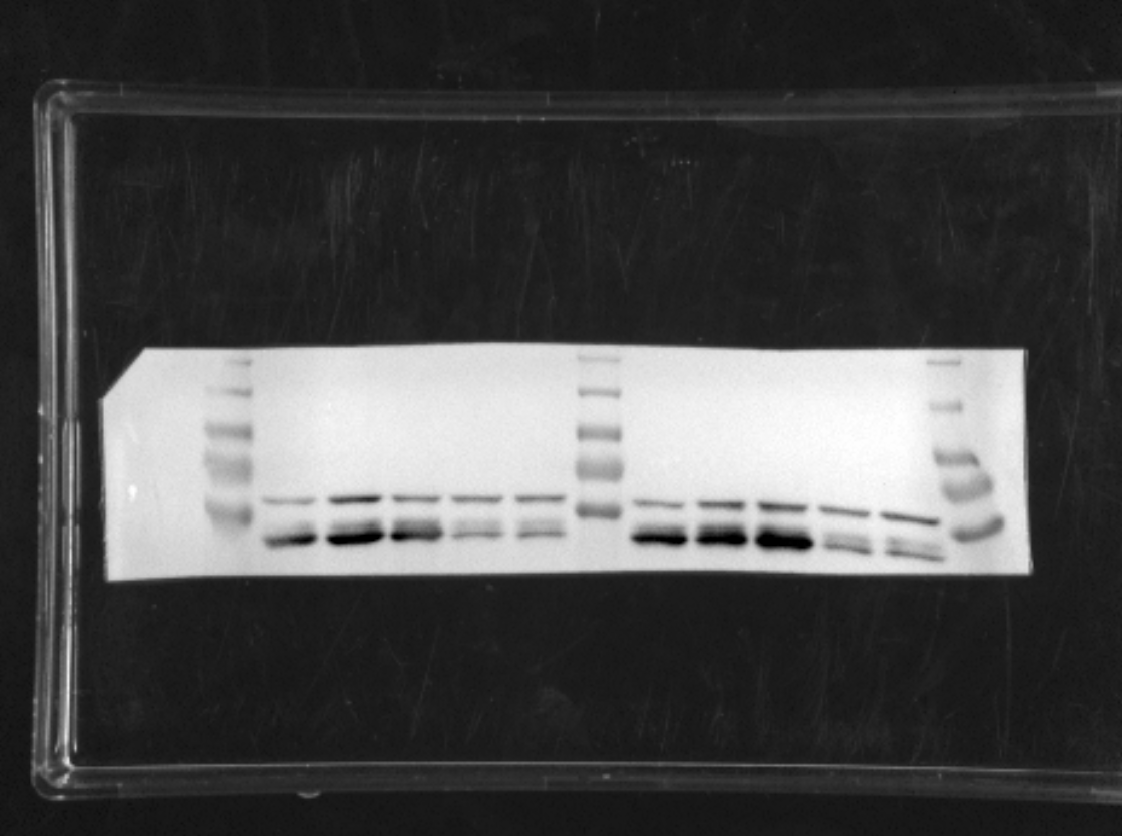

Supplement: S1 File — (ZIP) [file pone.0335225.s006.zip › Animal WB/Psmad/p-SMAD/yong.sma 2 20_7s hb.tif]

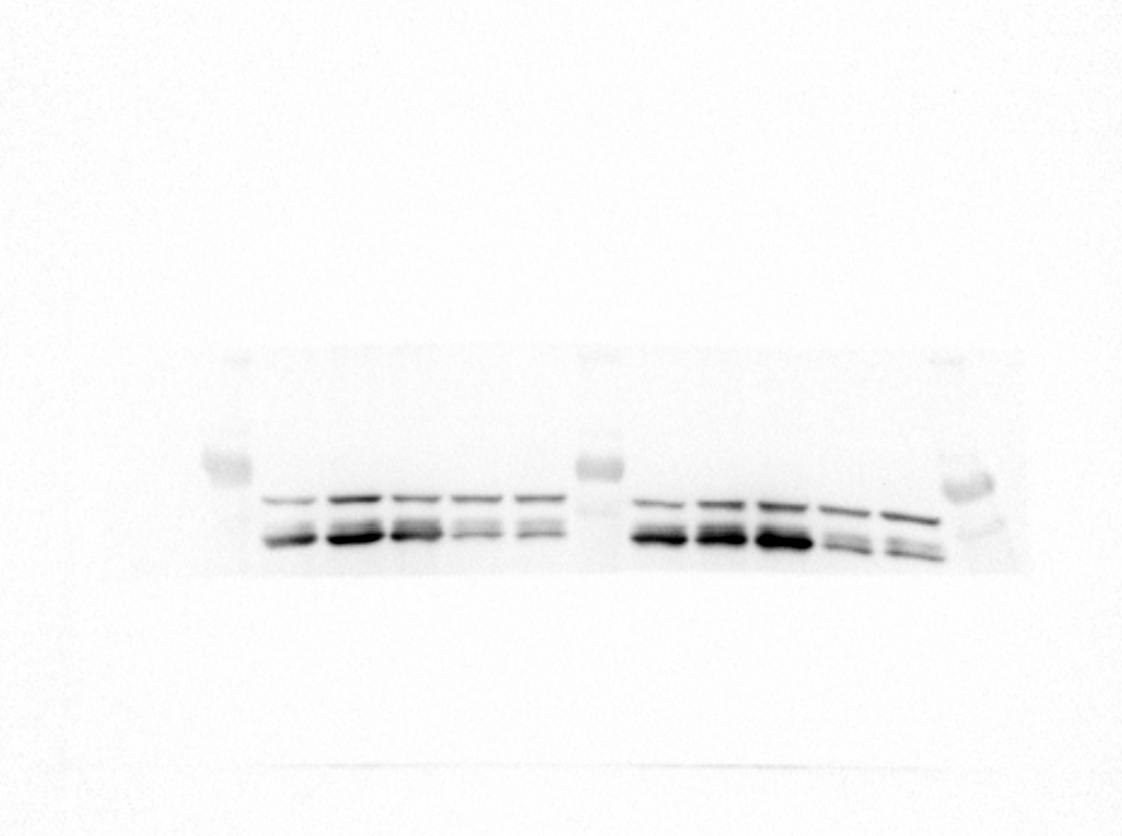

Supplement: S1 File — (ZIP) [file pone.0335225.s006.zip › Animal WB/Psmad/p-SMAD/yong.sma 2 20_7s.tif]

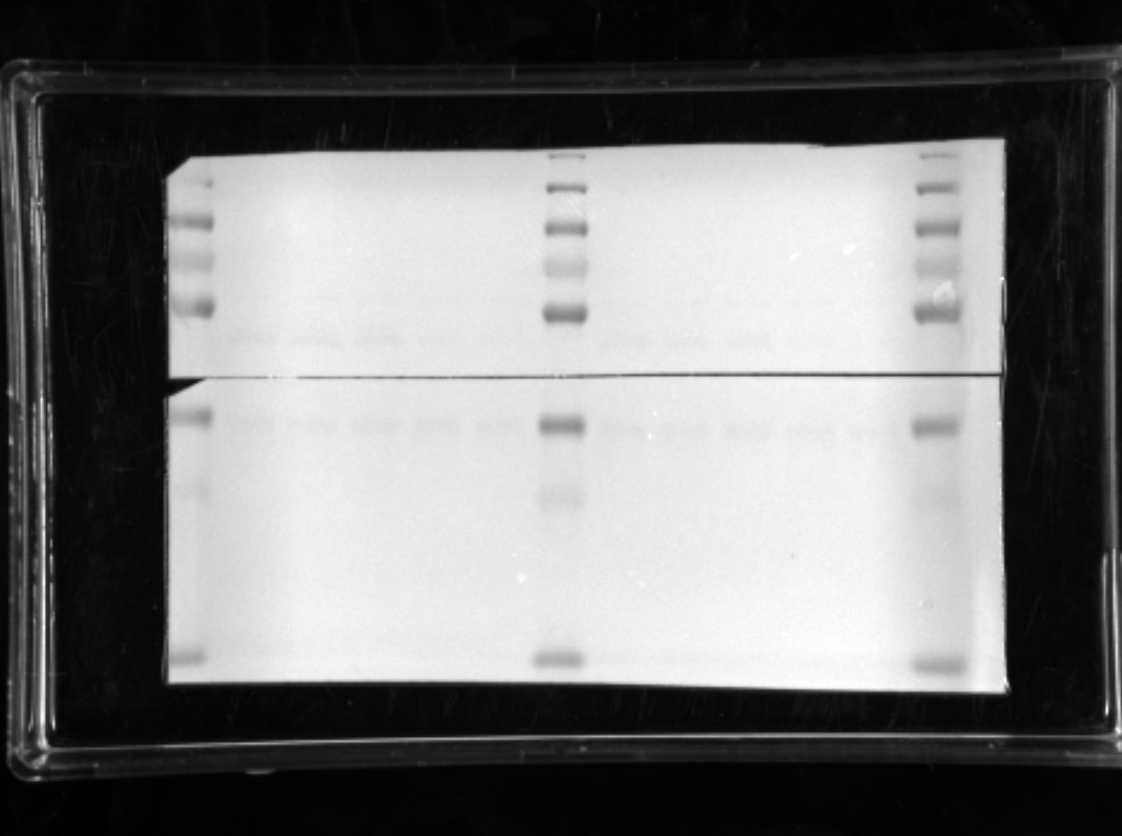

Supplement: S1 File — (ZIP) [file pone.0335225.s006.zip › Animal WB/Psmad/p-SMAD/图 maker 1.tif]

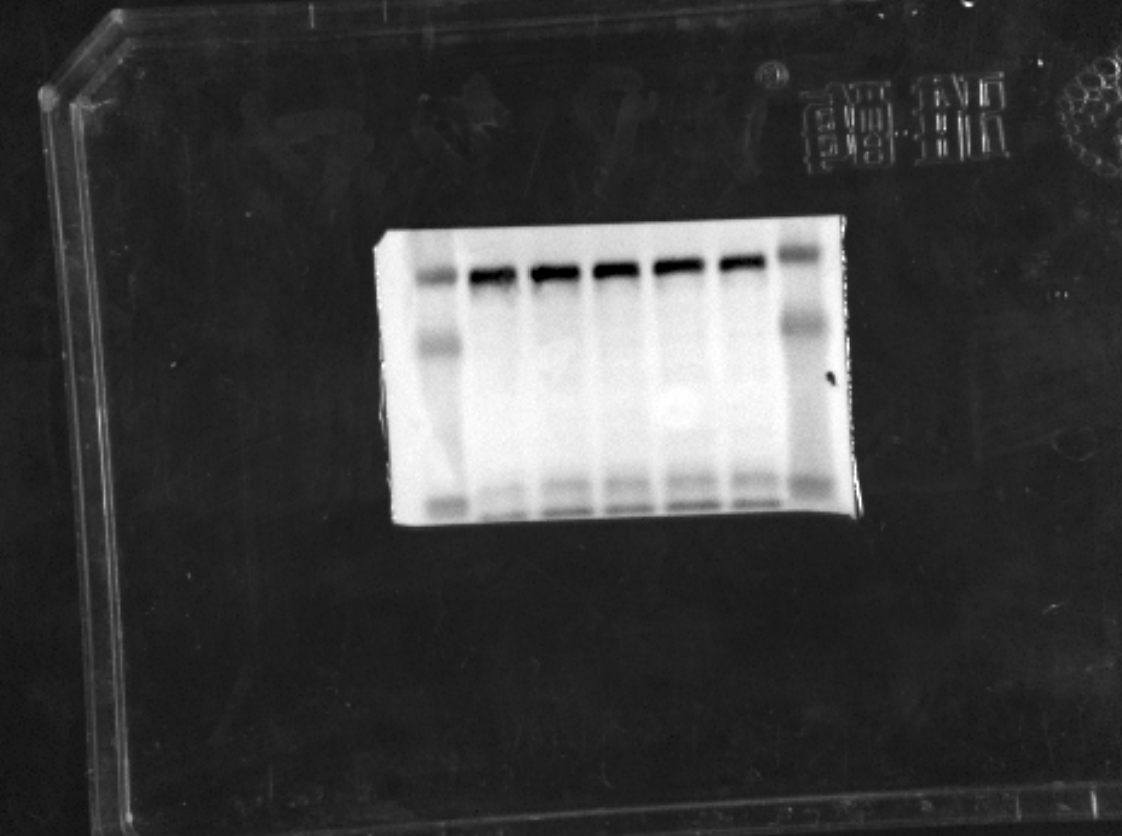

Supplement: S1 File — (ZIP) [file pone.0335225.s006.zip › Animal WB/Psmad/samd2/g 1 10_7s hb.tif]

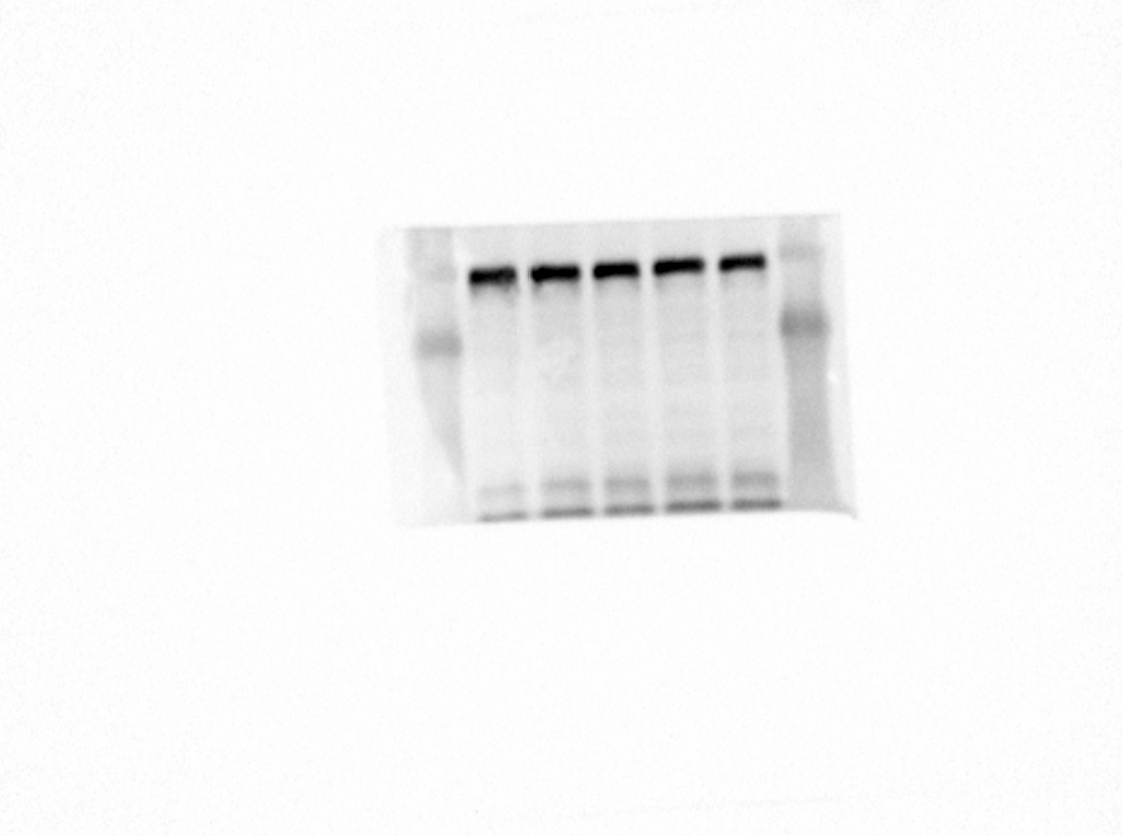

Supplement: S1 File — (ZIP) [file pone.0335225.s006.zip › Animal WB/Psmad/samd2/g 1 10_7s.tif]

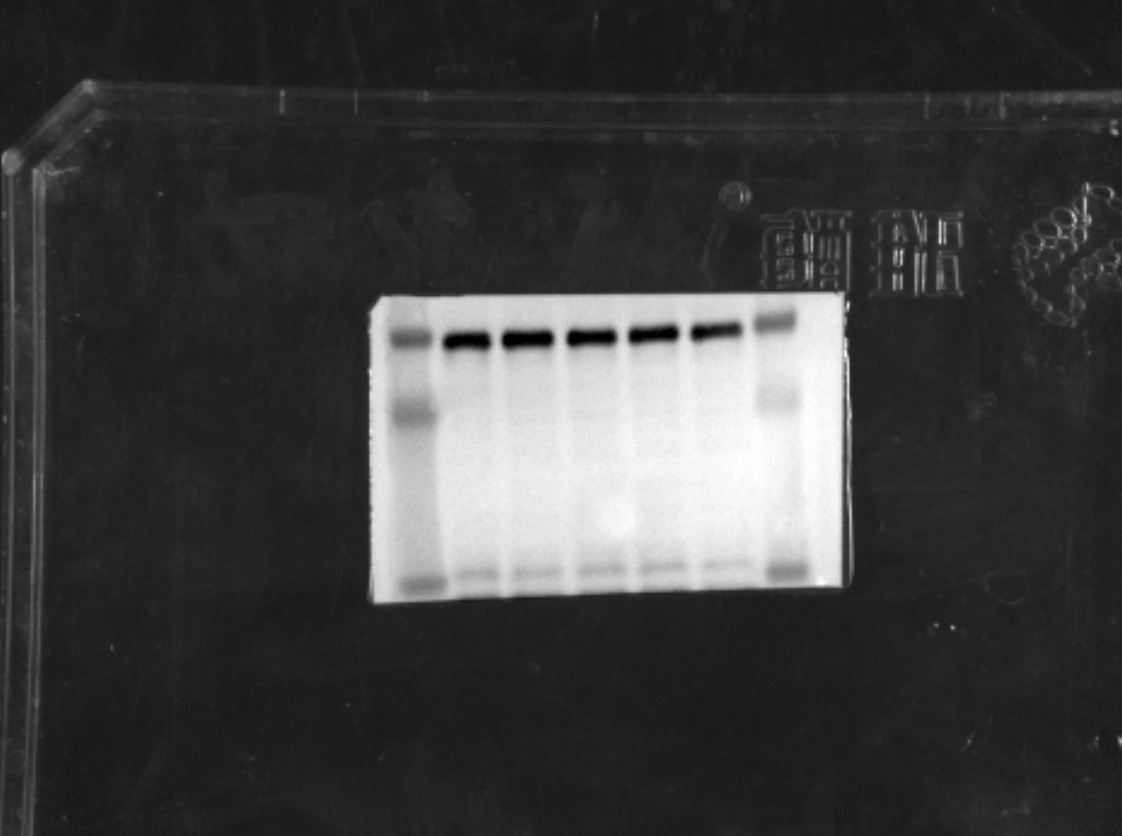

Supplement: S1 File — (ZIP) [file pone.0335225.s006.zip › Animal WB/Psmad/samd2/g 2 7_4s hb.tif]

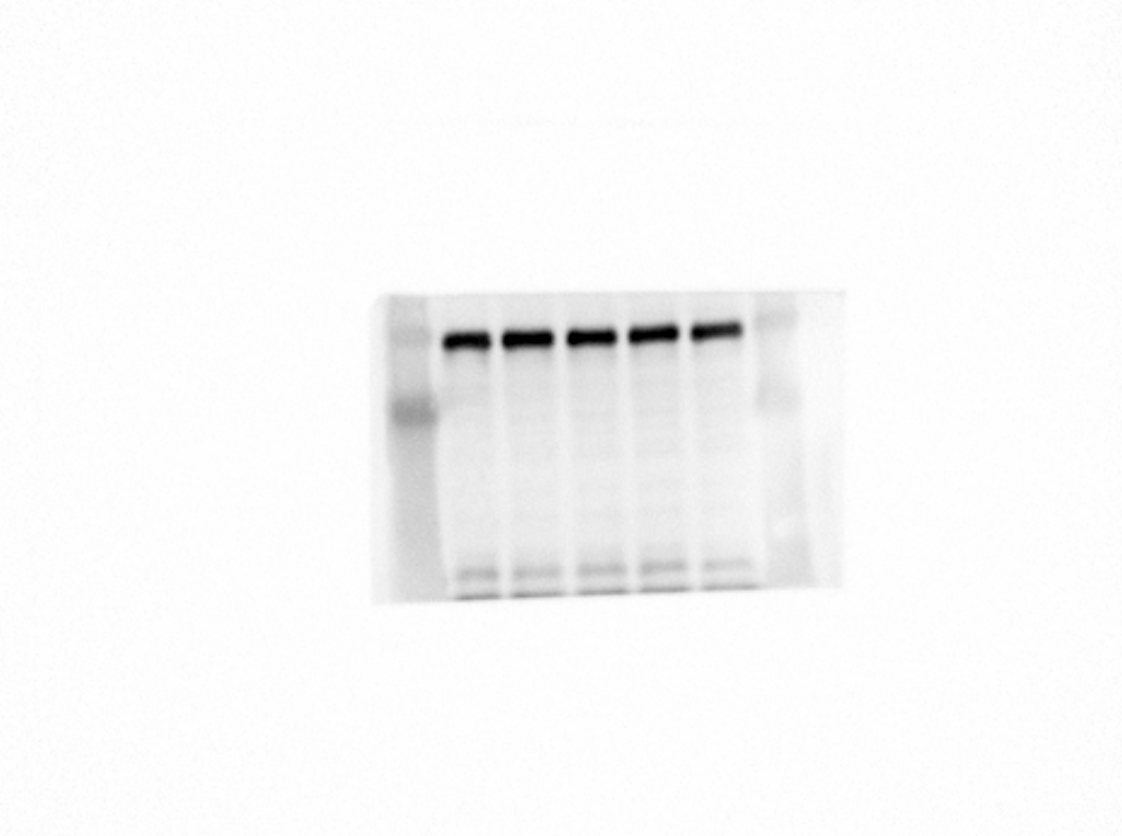

Supplement: S1 File — (ZIP) [file pone.0335225.s006.zip › Animal WB/Psmad/samd2/g 2 7_4s.tif]

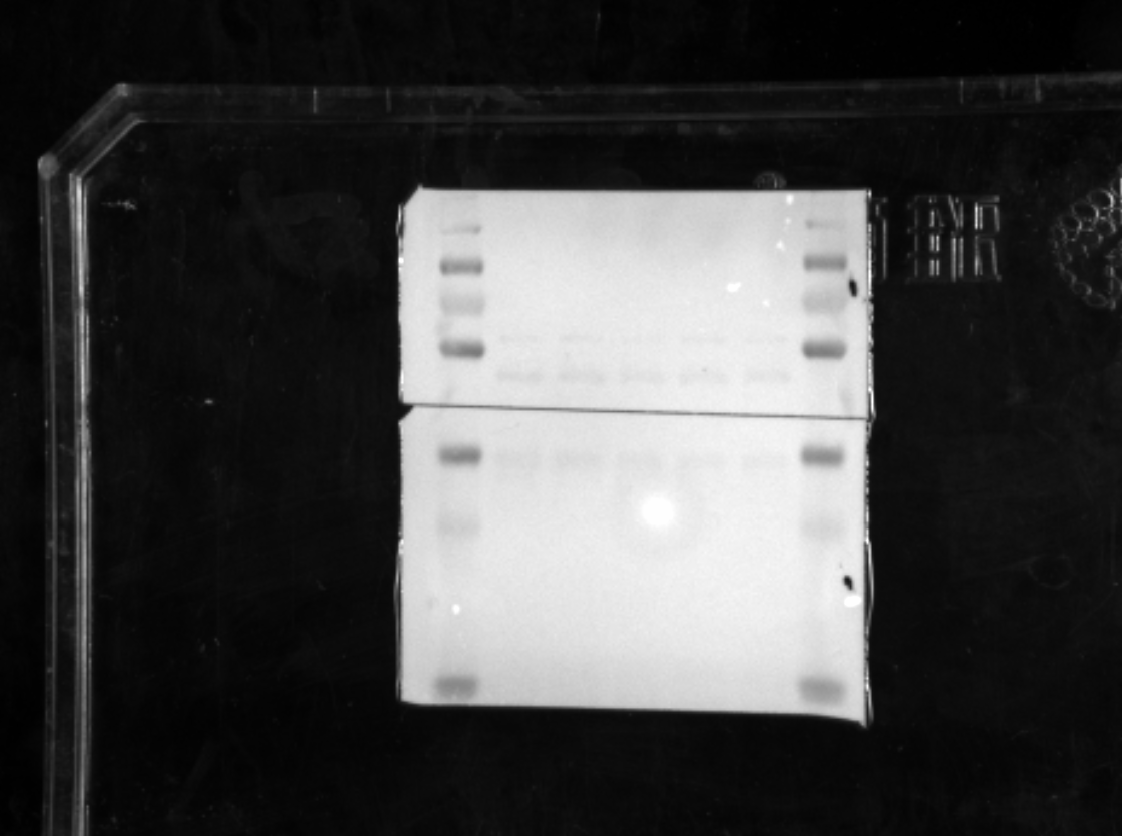

Supplement: S1 File — (ZIP) [file pone.0335225.s006.zip › Animal WB/Psmad/samd2/maker1.tif]

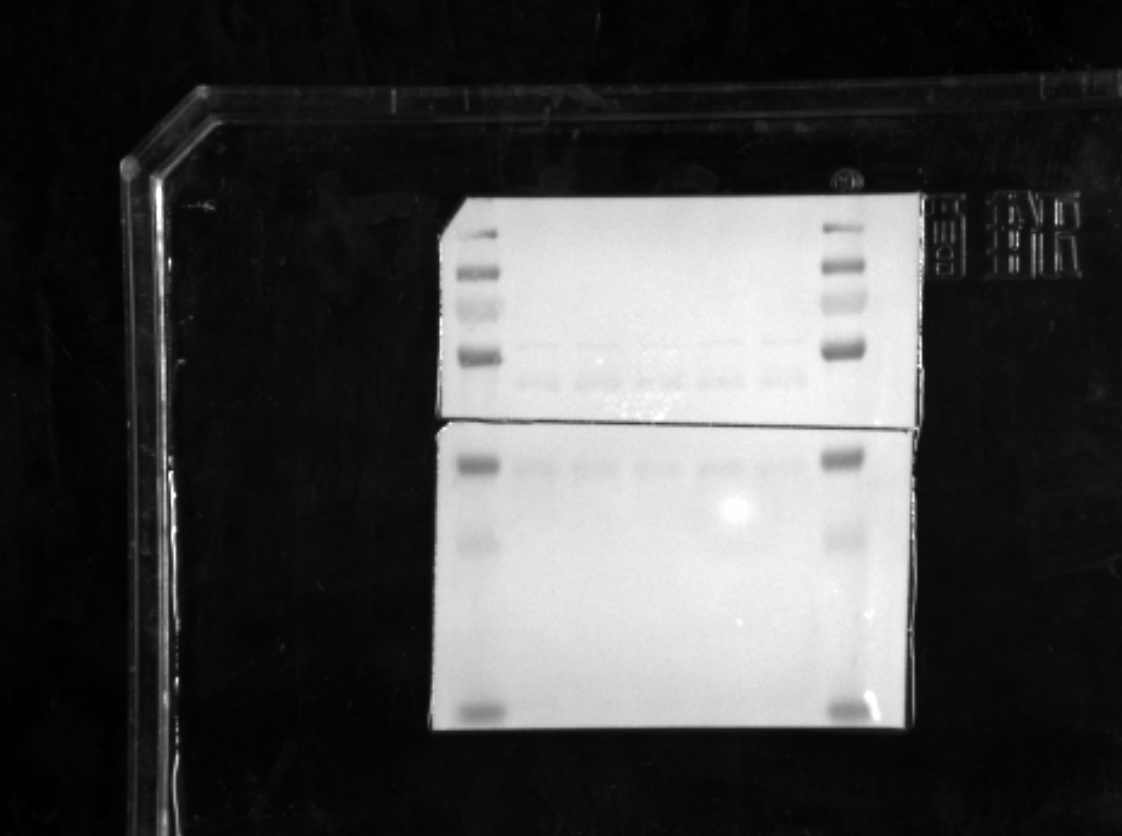

Supplement: S1 File — (ZIP) [file pone.0335225.s006.zip › Animal WB/Psmad/samd2/maker2.tif]

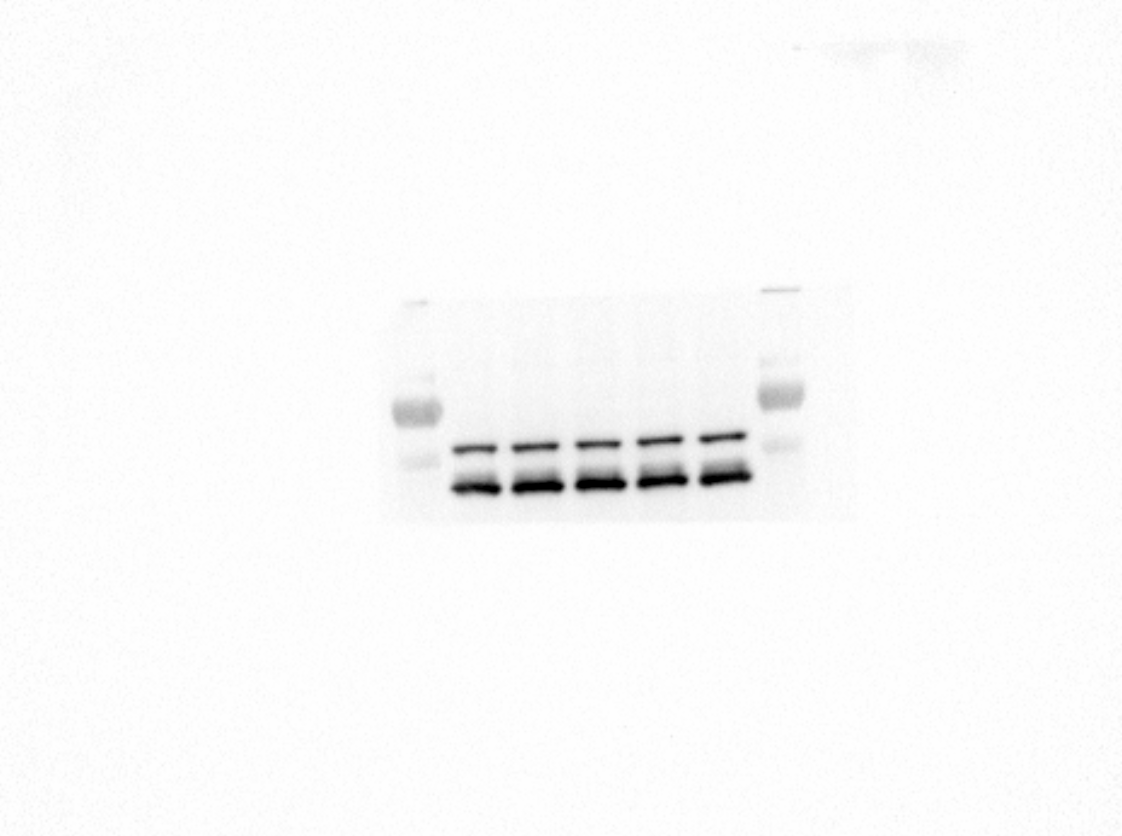

Supplement: S1 File — (ZIP) [file pone.0335225.s006.zip › Animal WB/Psmad/samd2/sma3 14_1s 2.tif]

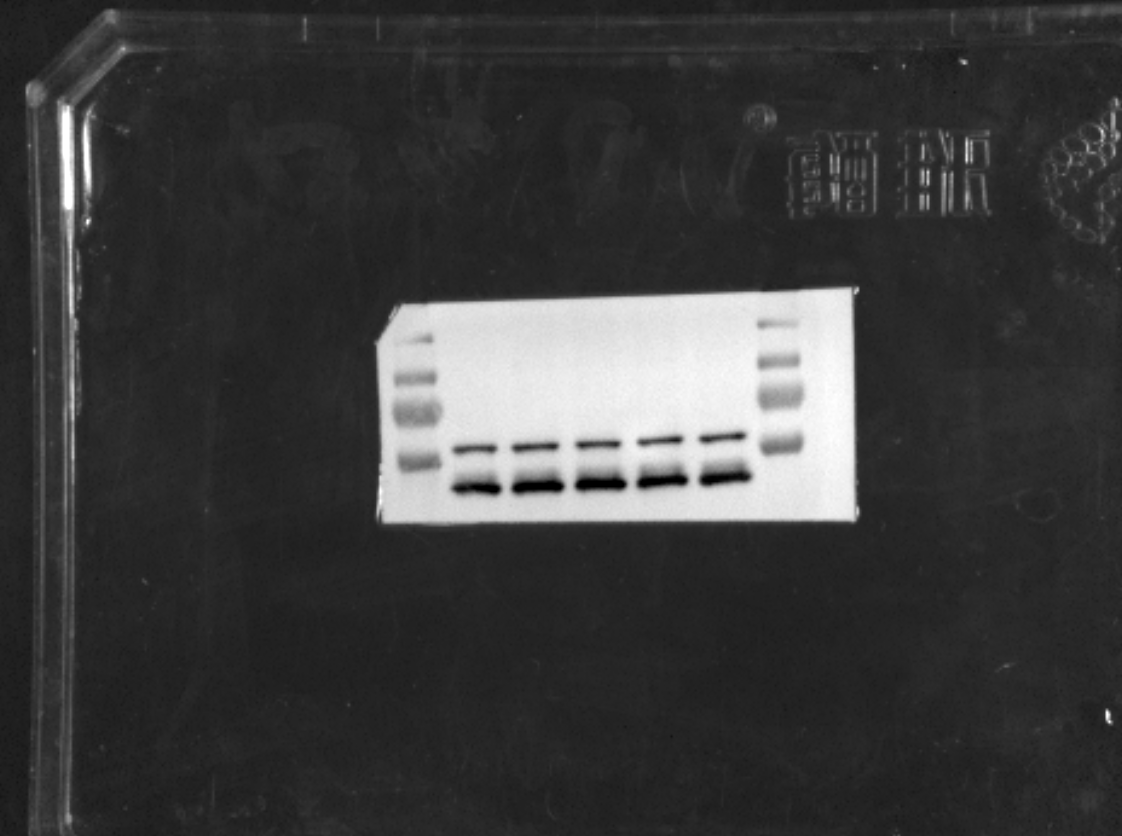

Supplement: S1 File — (ZIP) [file pone.0335225.s006.zip › Animal WB/Psmad/samd2/sma3 14_1s hb 2.tif]

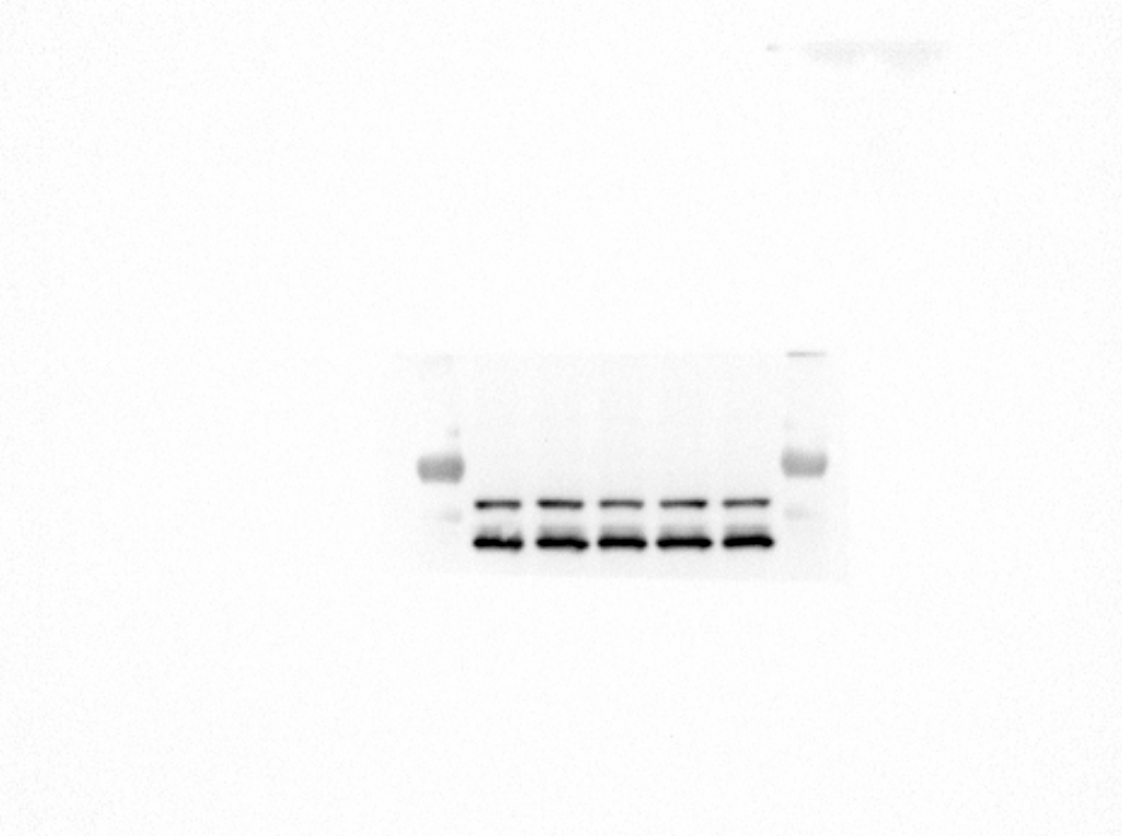

Supplement: S1 File — (ZIP) [file pone.0335225.s006.zip › Animal WB/Psmad/samd2/sma3 20_7s 1.tif]

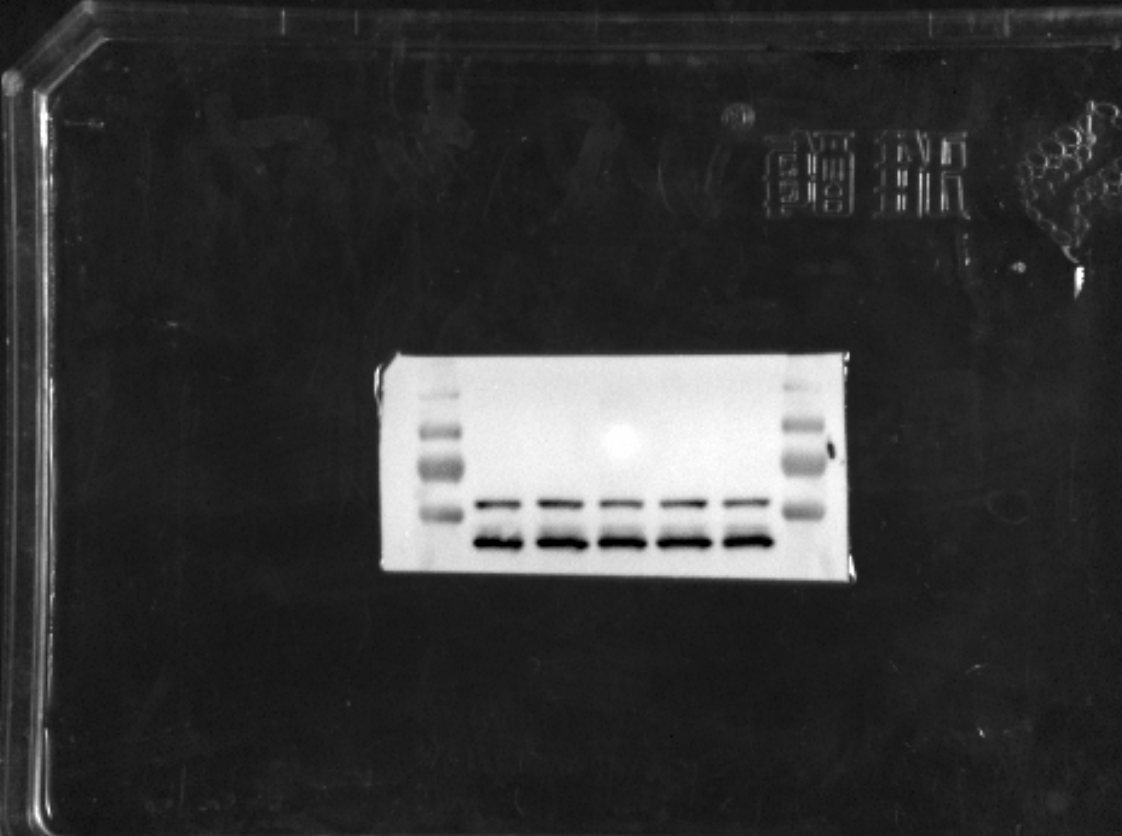

Supplement: S1 File — (ZIP) [file pone.0335225.s006.zip › Animal WB/Psmad/samd2/sma3 20_7s hb 1.tif]

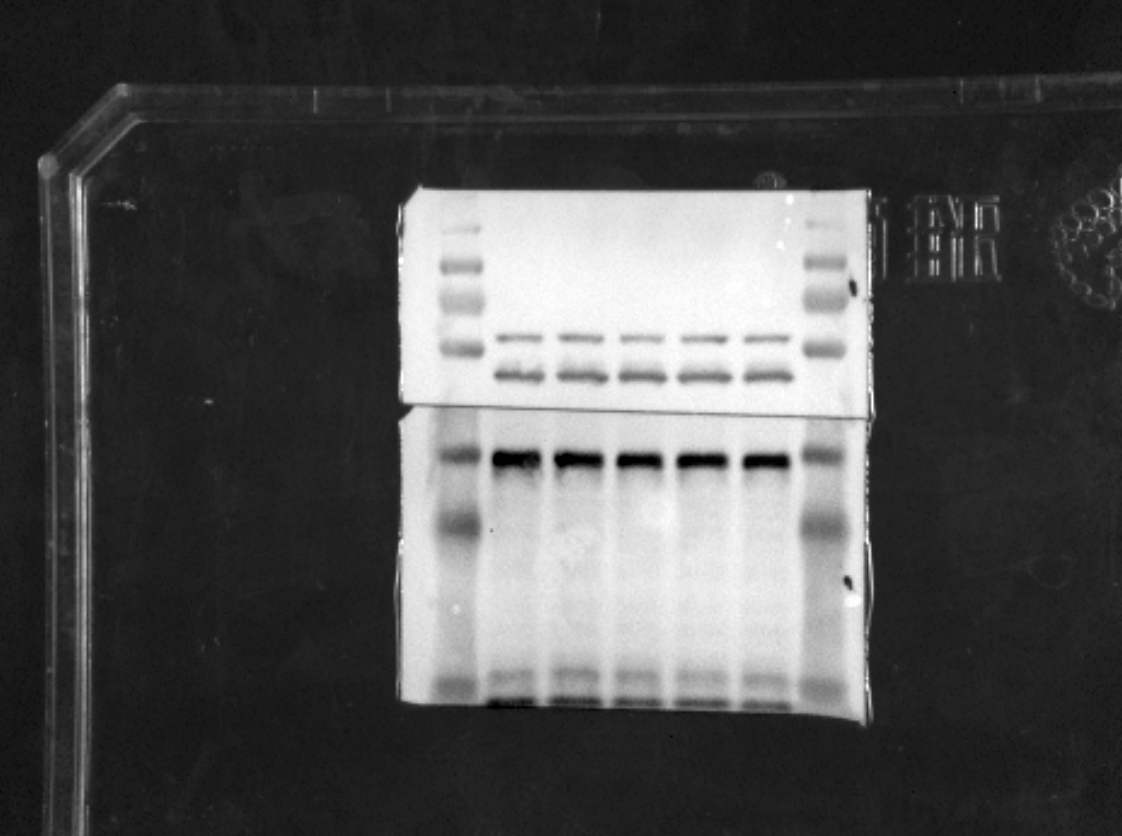

Supplement: S1 File — (ZIP) [file pone.0335225.s006.zip › Animal WB/Psmad/samd2/zhengmo 1.tif]

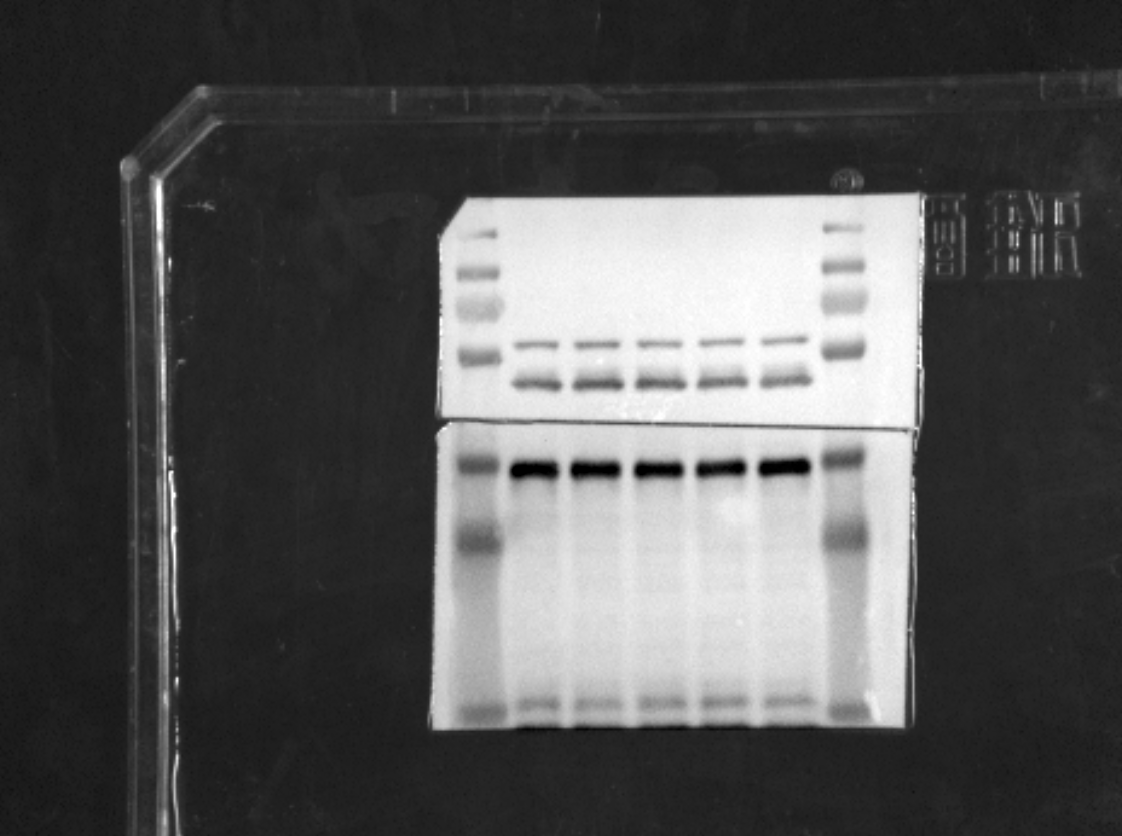

Supplement: S1 File — (ZIP) [file pone.0335225.s006.zip › Animal WB/Psmad/samd2/zhengmo 2.tif]

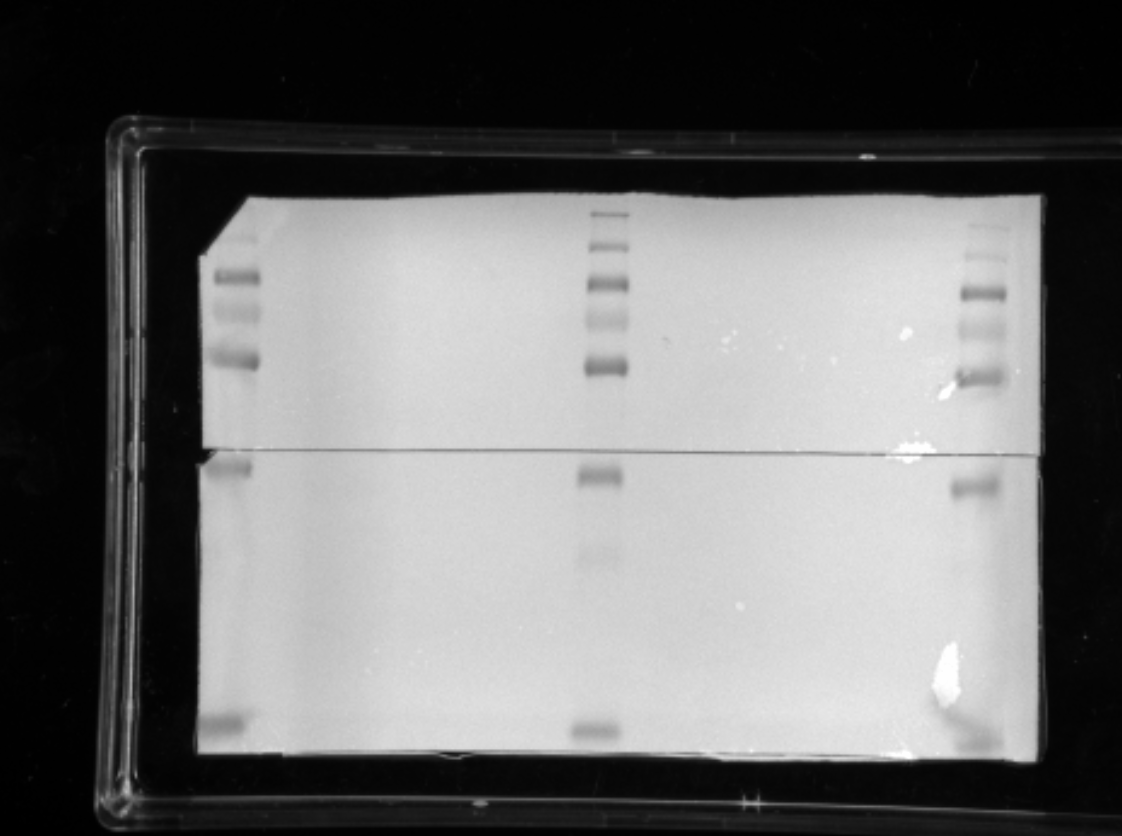

Supplement: S1 File — (ZIP) [file pone.0335225.s006.zip › Animal WB/TGF/MAKER 2.tif]

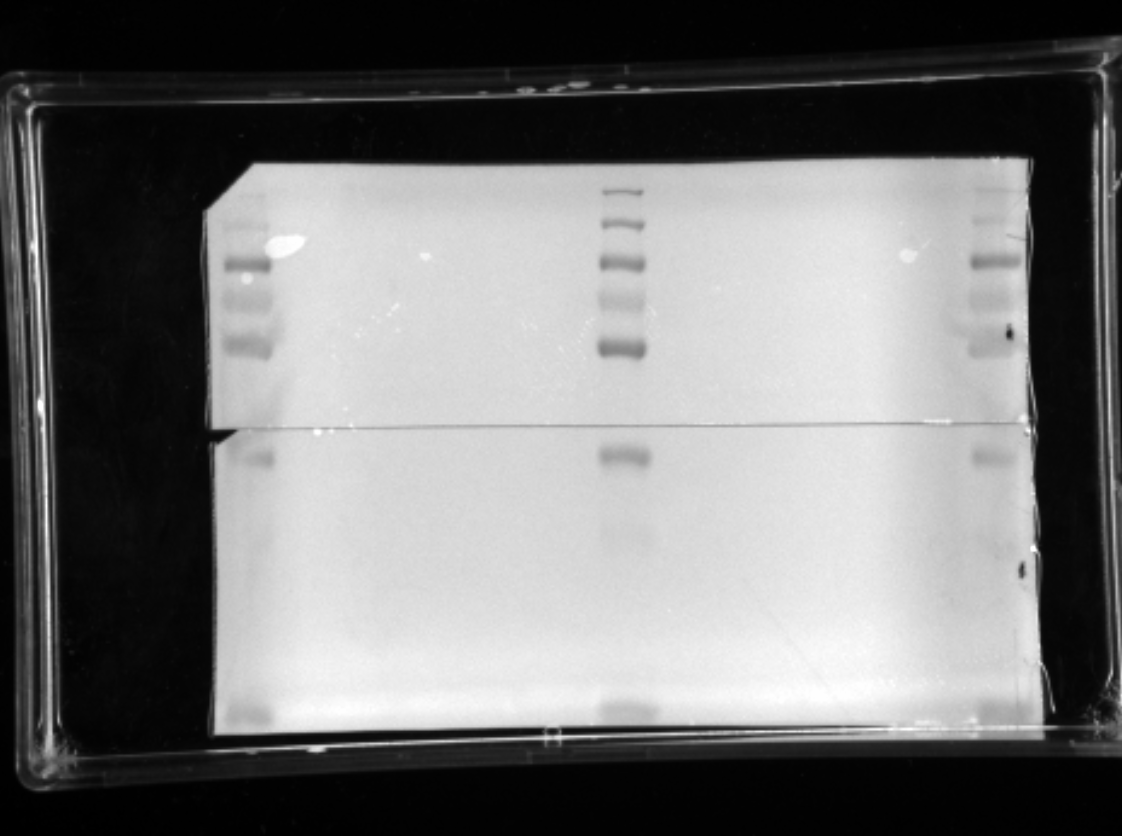

Supplement: S1 File — (ZIP) [file pone.0335225.s006.zip › Animal WB/TGF/图 MAKER 1.tif]

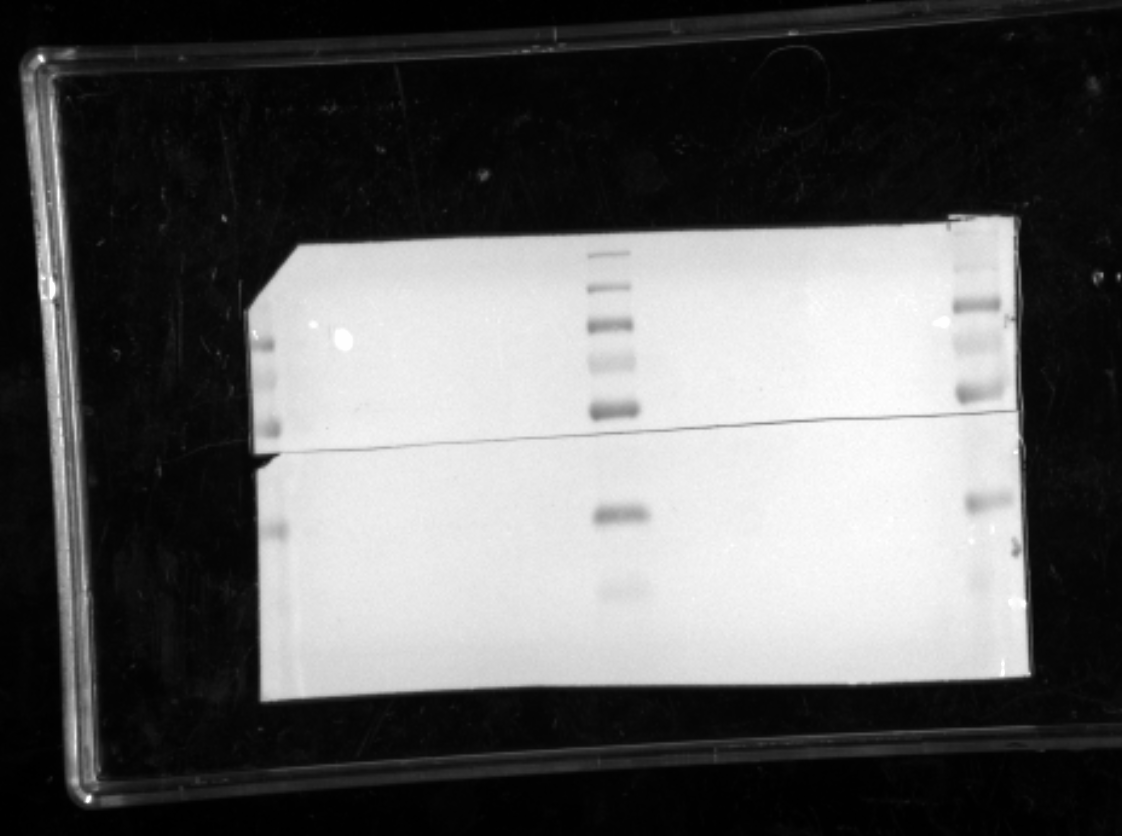

Supplement: S1 File — (ZIP) [file pone.0335225.s006.zip › Animal WB/V/maker1.tif]

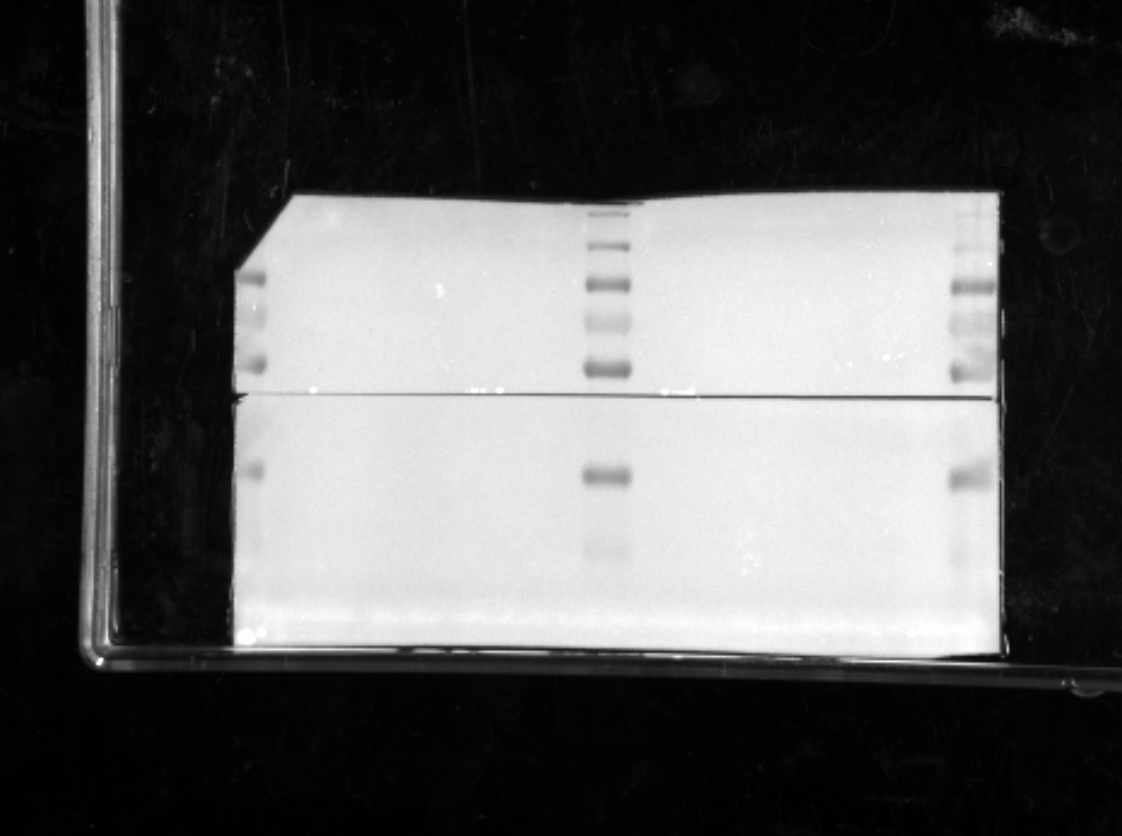

Supplement: S1 File — (ZIP) [file pone.0335225.s006.zip › Animal WB/V/图 maker2.tif]

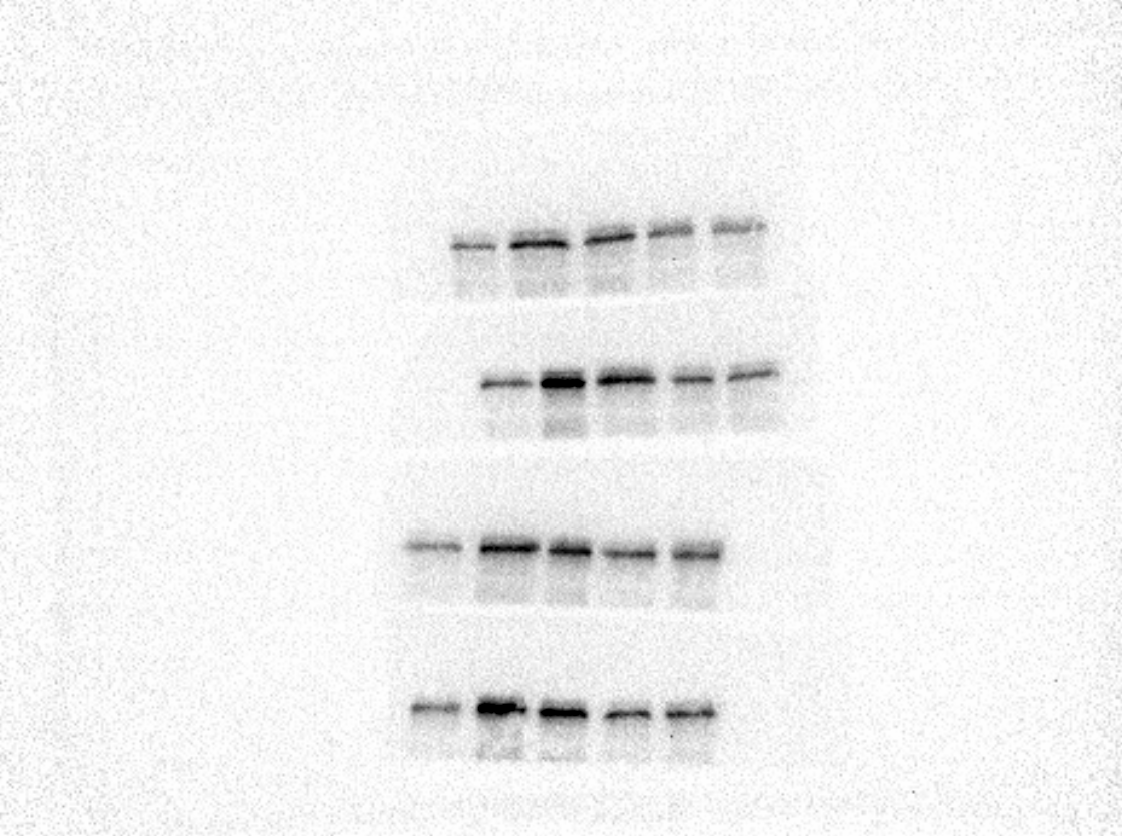

Supplement: S2 File — (ZIP) [file pone.0335225.s007.zip › Cell WB/C N/COL 40S 7 4 3 6.tif]

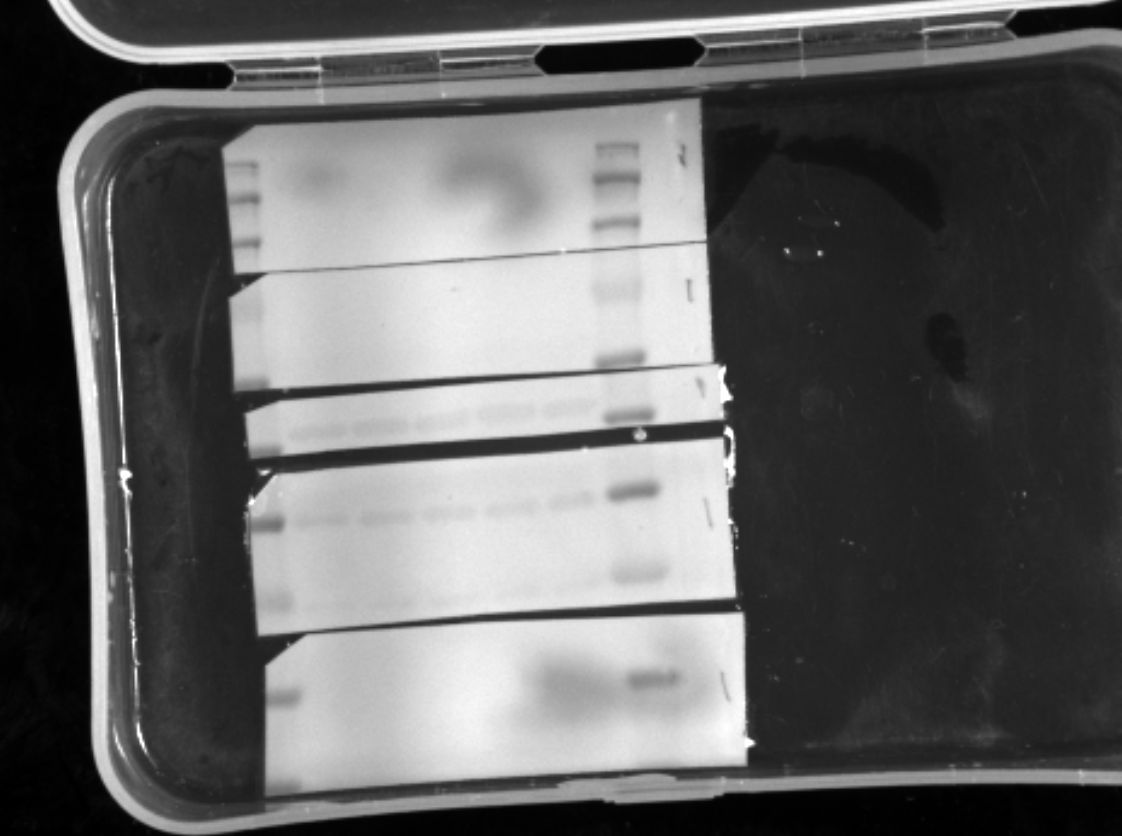

Supplement: S2 File — (ZIP) [file pone.0335225.s007.zip › Cell WB/C N/MAKER 1.tif]

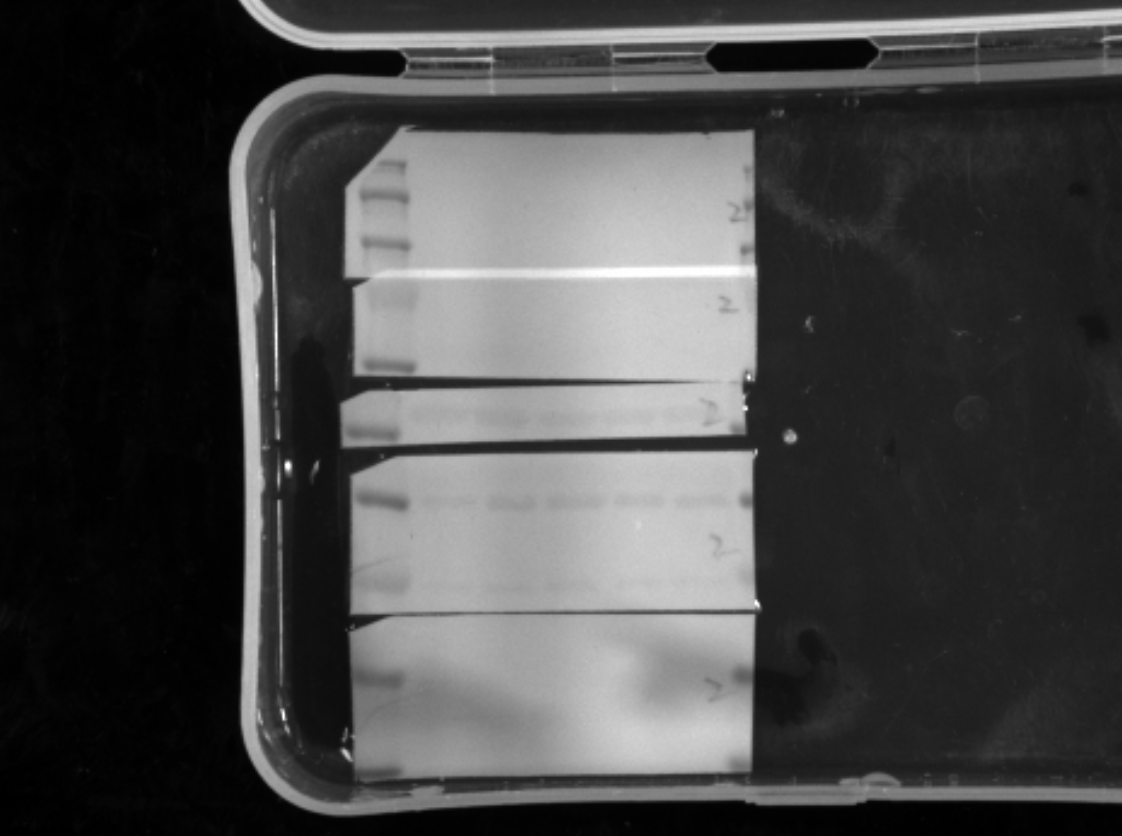

Supplement: S2 File — (ZIP) [file pone.0335225.s007.zip › Cell WB/C N/MAKER 2.tif]

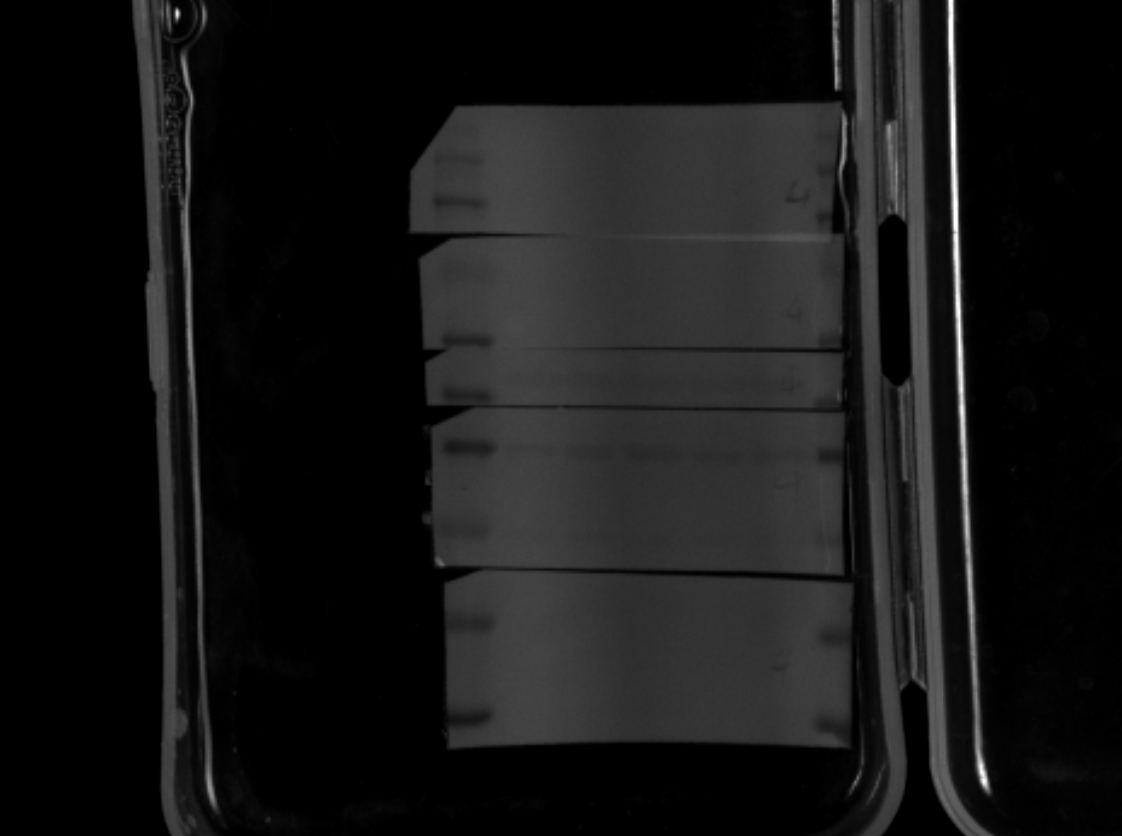

Supplement: S2 File — (ZIP) [file pone.0335225.s007.zip › Cell WB/C N/MAKER 4.tif]

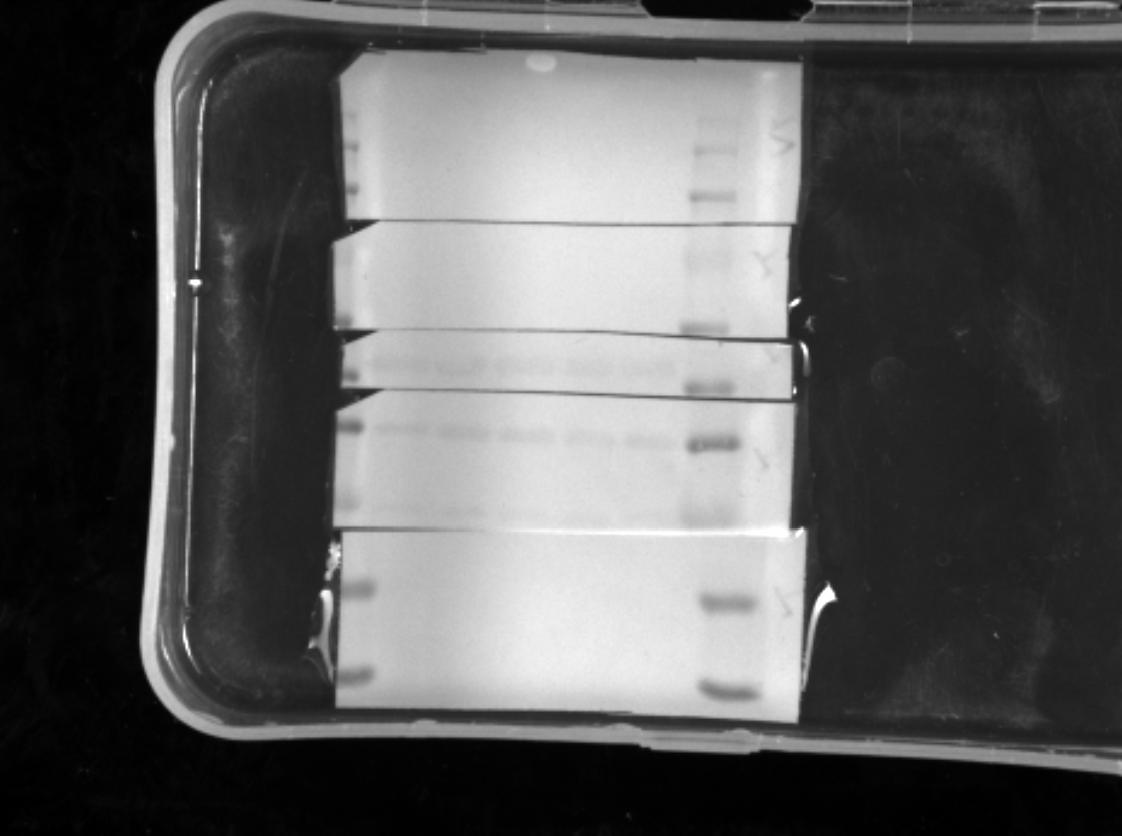

Supplement: S2 File — (ZIP) [file pone.0335225.s007.zip › Cell WB/C N/MAKER 5.tif]

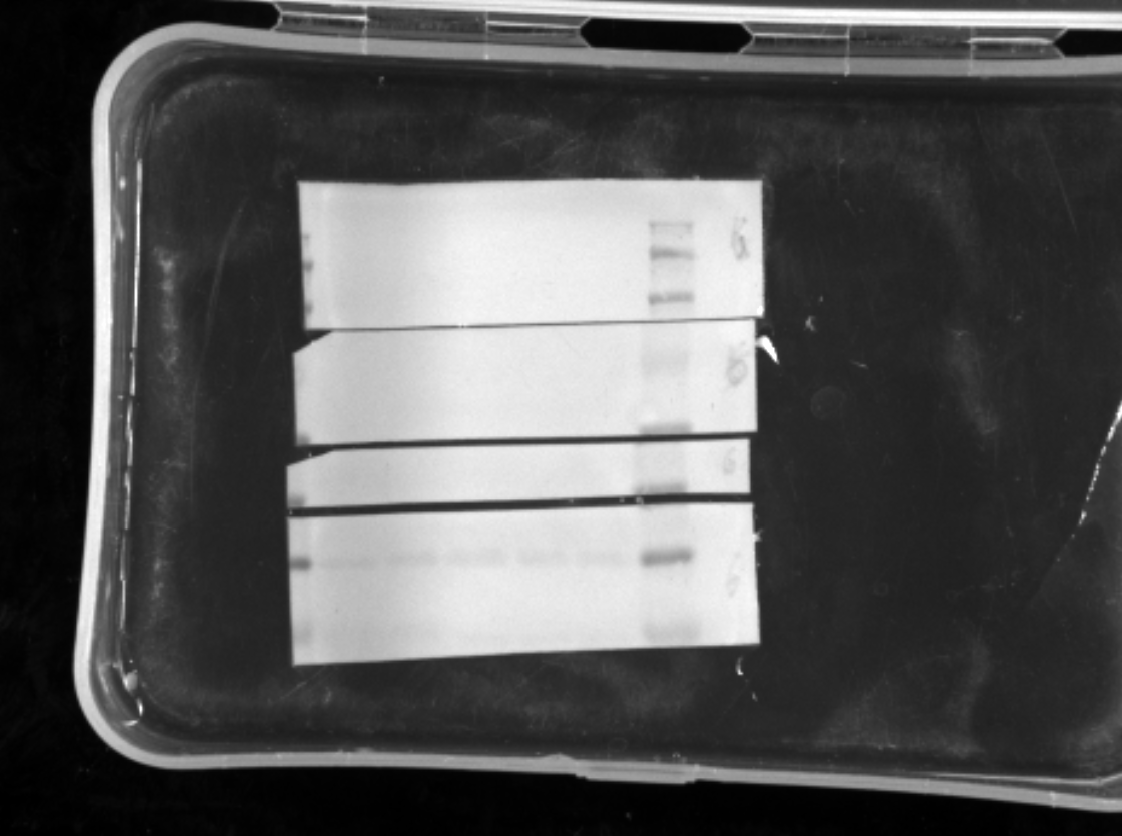

Supplement: S2 File — (ZIP) [file pone.0335225.s007.zip › Cell WB/C N/MAKER 6.tif]

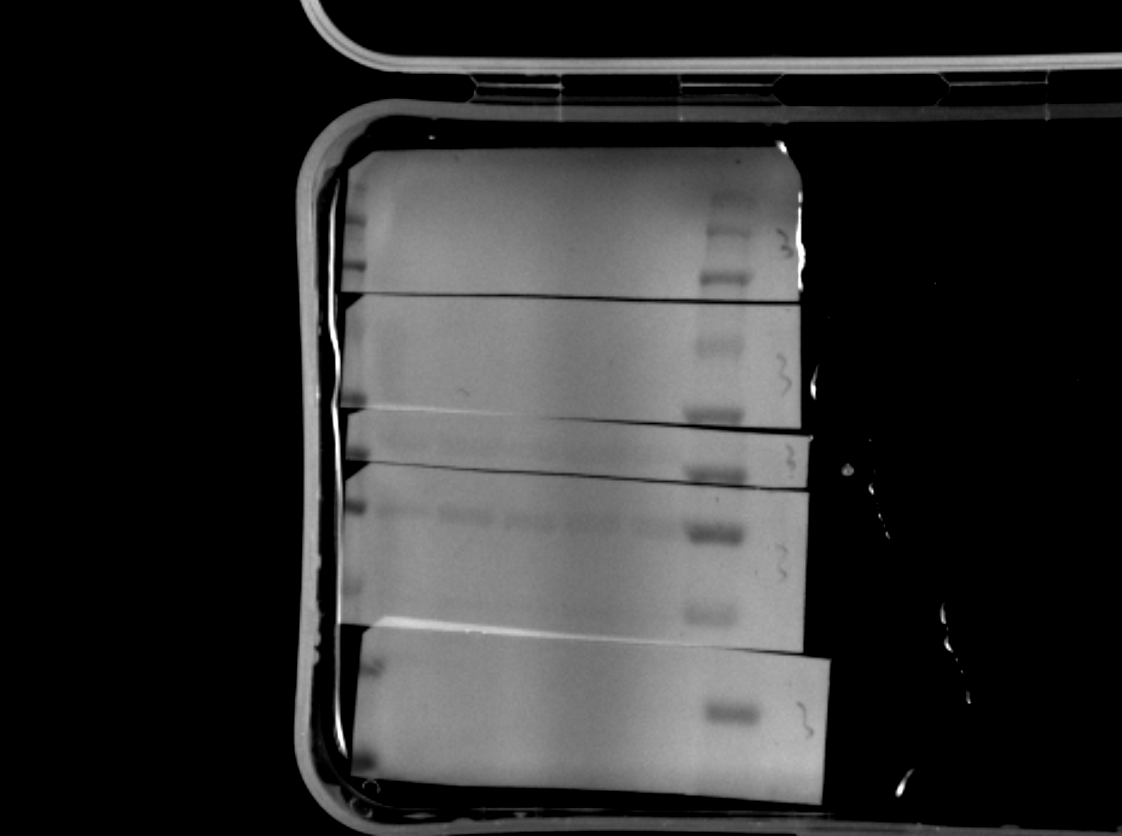

Supplement: S2 File — (ZIP) [file pone.0335225.s007.zip › Cell WB/C N/MARKER 3.tif]

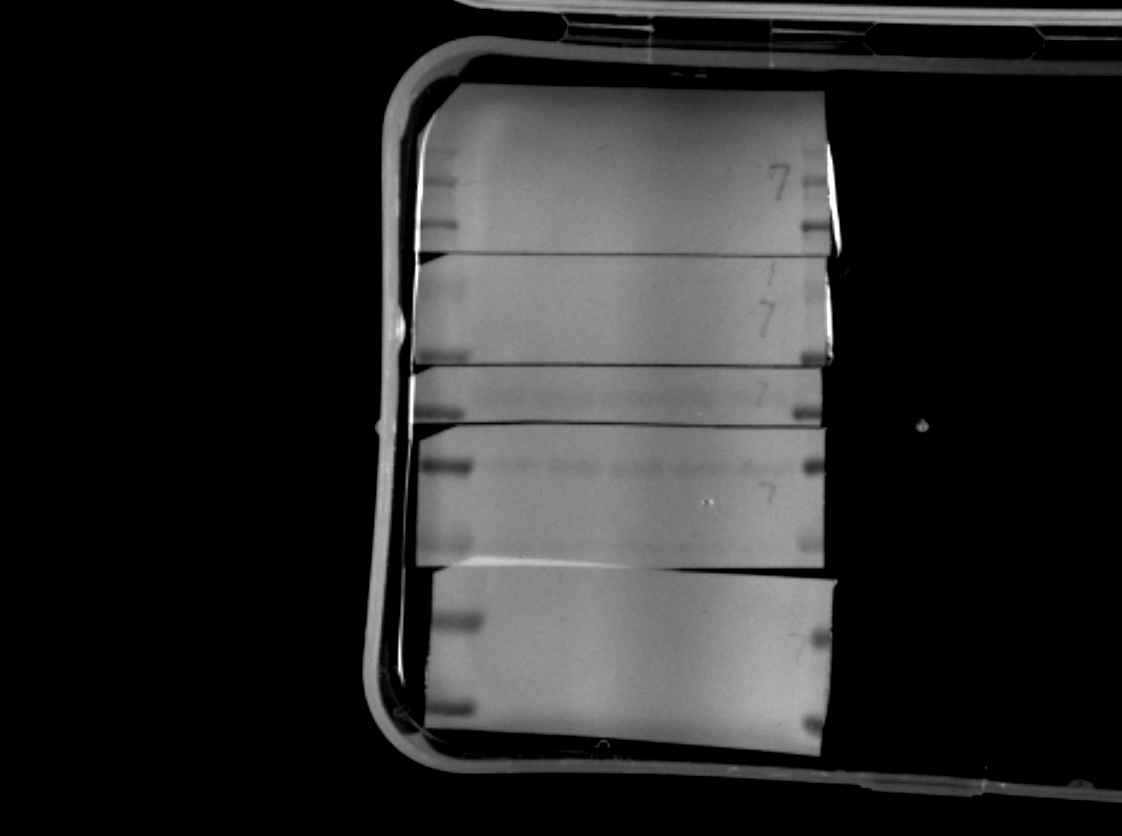

Supplement: S2 File — (ZIP) [file pone.0335225.s007.zip › Cell WB/C N/MARKER 7.tif]

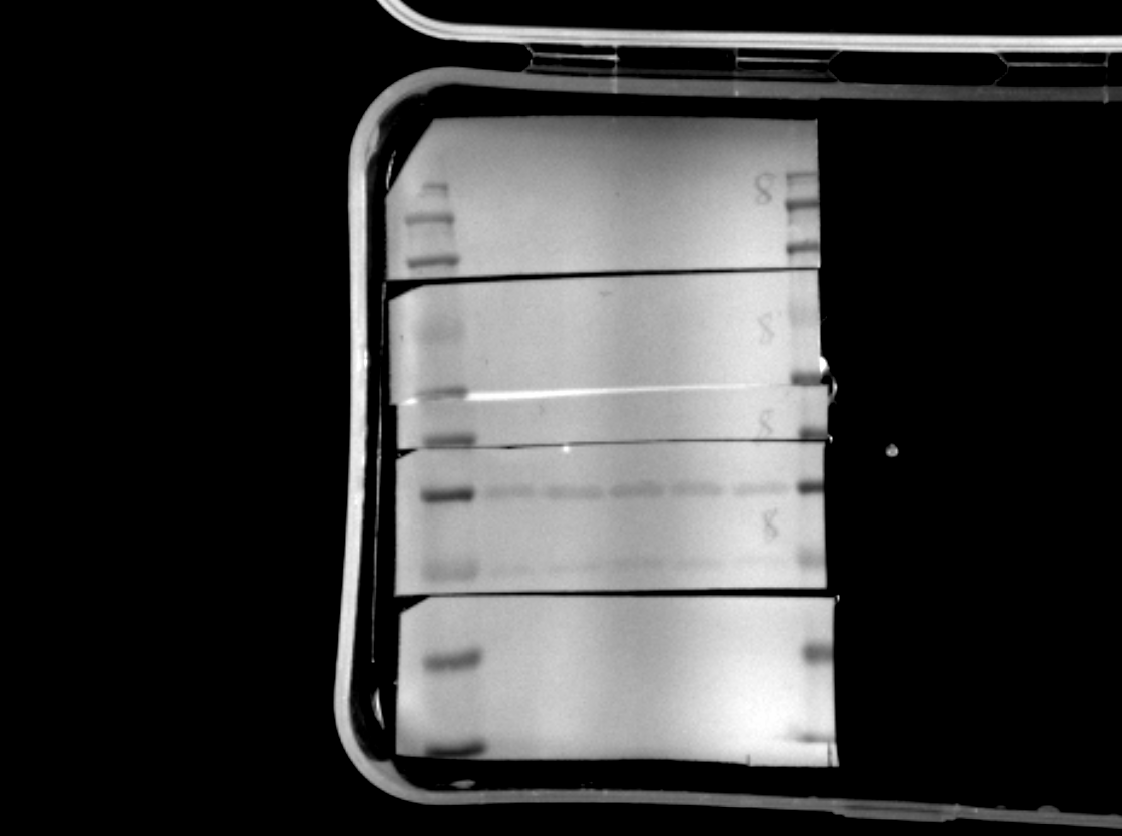

Supplement: S2 File — (ZIP) [file pone.0335225.s007.zip › Cell WB/C N/MARKER 8.tif]

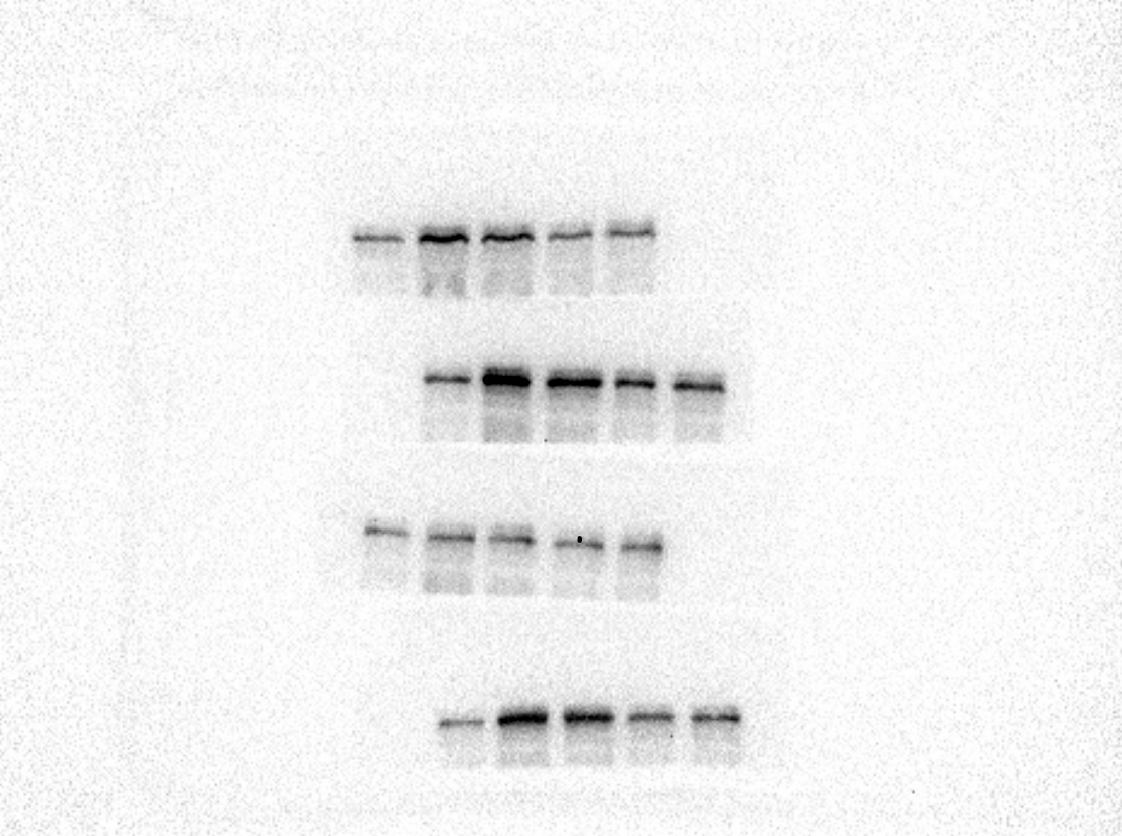

Supplement: S2 File — (ZIP) [file pone.0335225.s007.zip › Cell WB/C N/N 30S 5 2 1 8.tif]

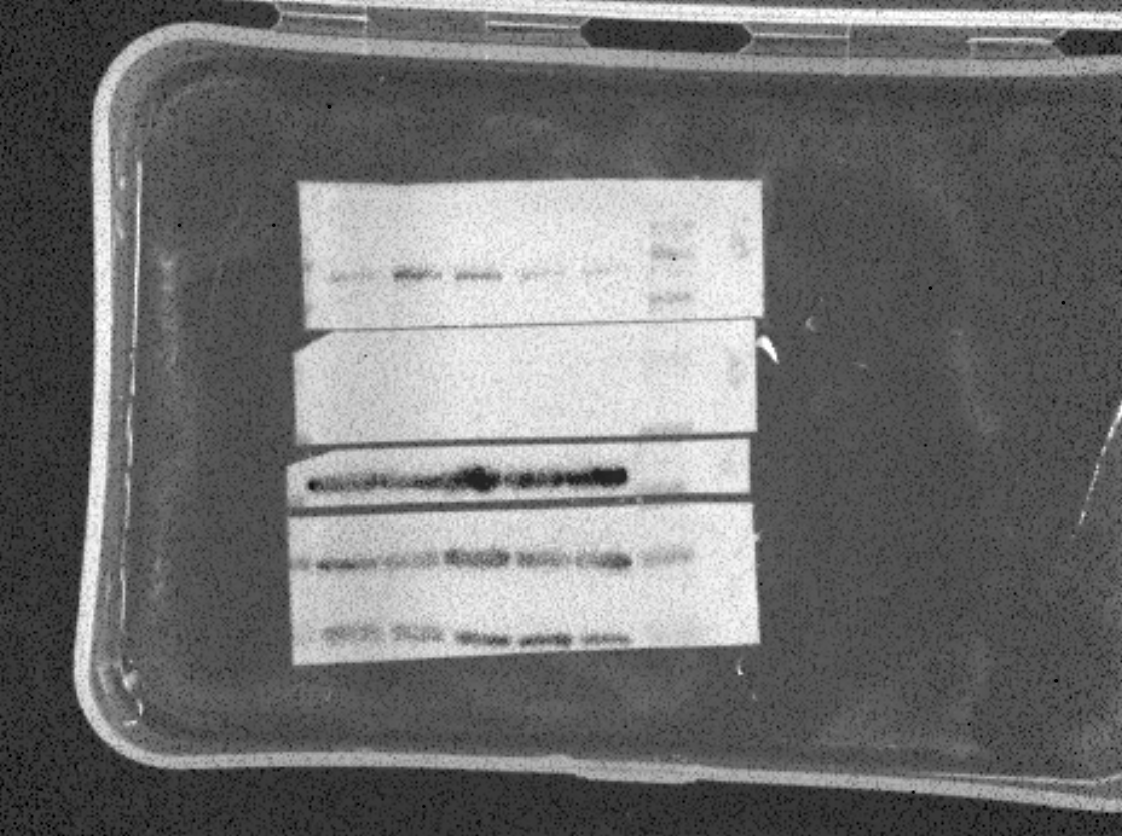

Supplement: S2 File — (ZIP) [file pone.0335225.s007.zip › Cell WB/C N/ZHENGM 6.tif]

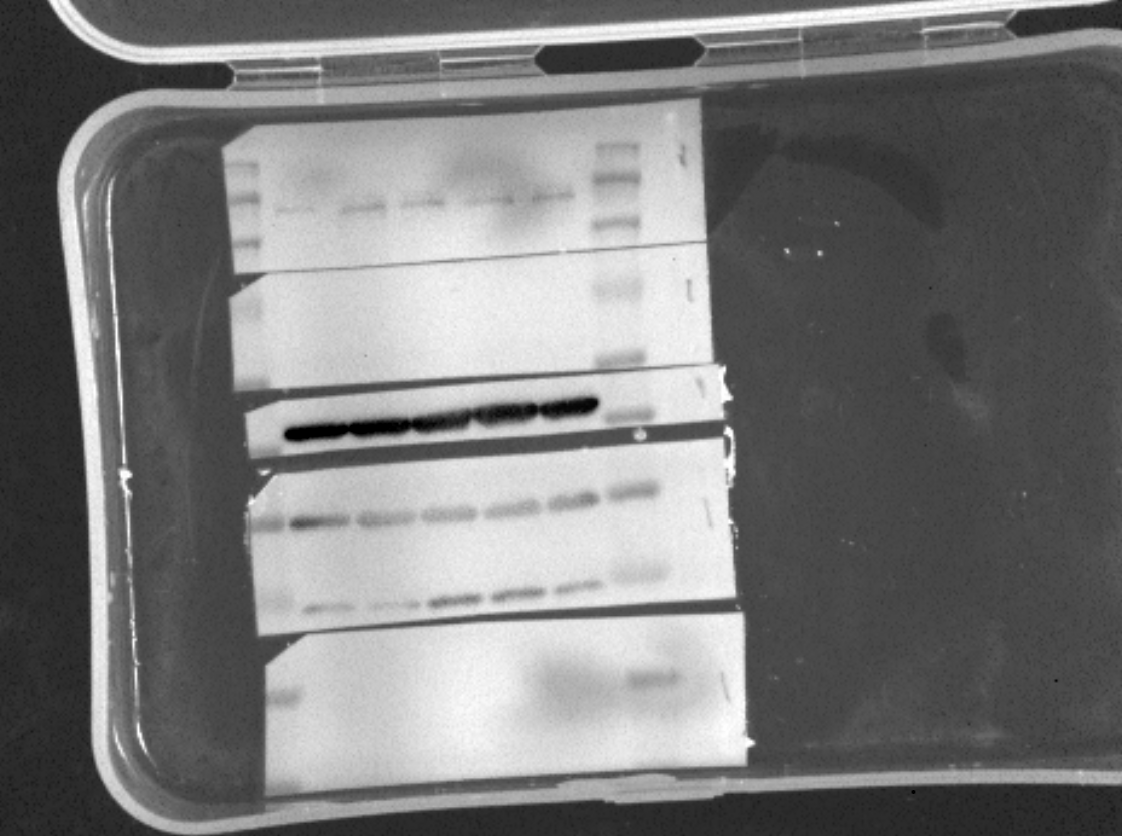

Supplement: S2 File — (ZIP) [file pone.0335225.s007.zip › Cell WB/C N/ZHENGMO 1.tif]

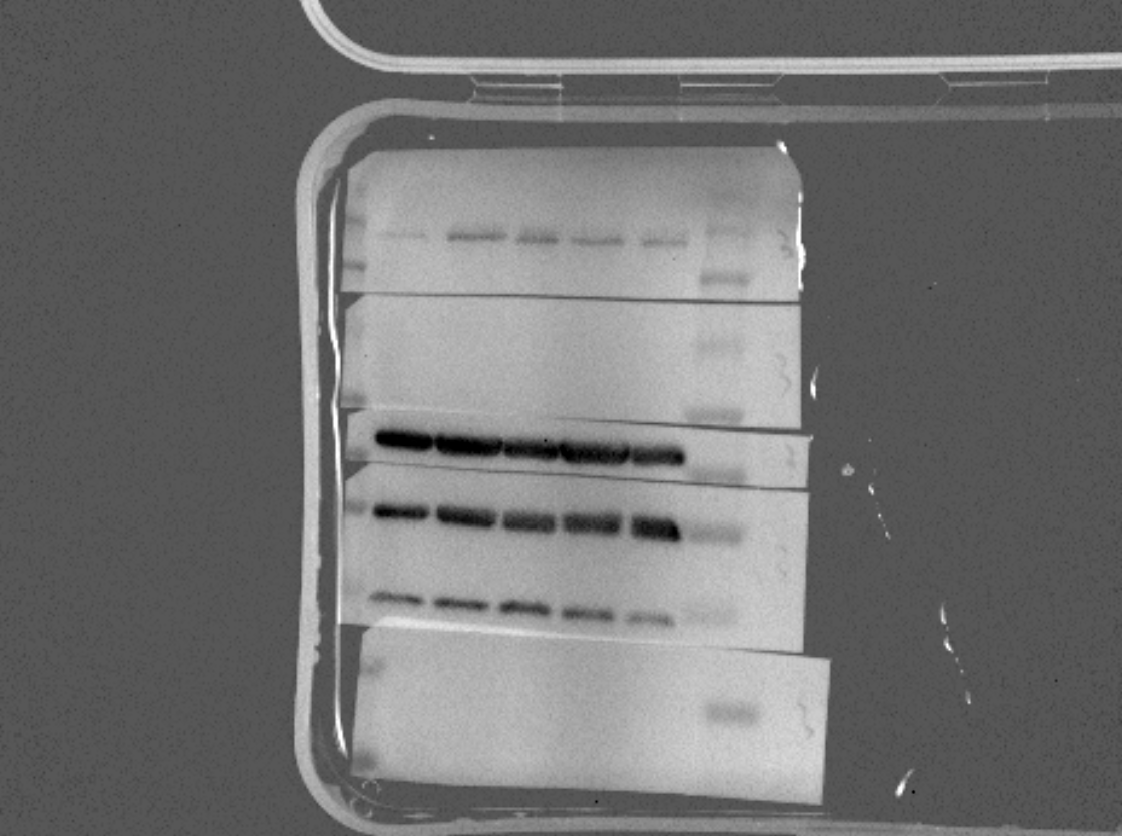

Supplement: S2 File — (ZIP) [file pone.0335225.s007.zip › Cell WB/C N/ZHENGMO 3.tif]

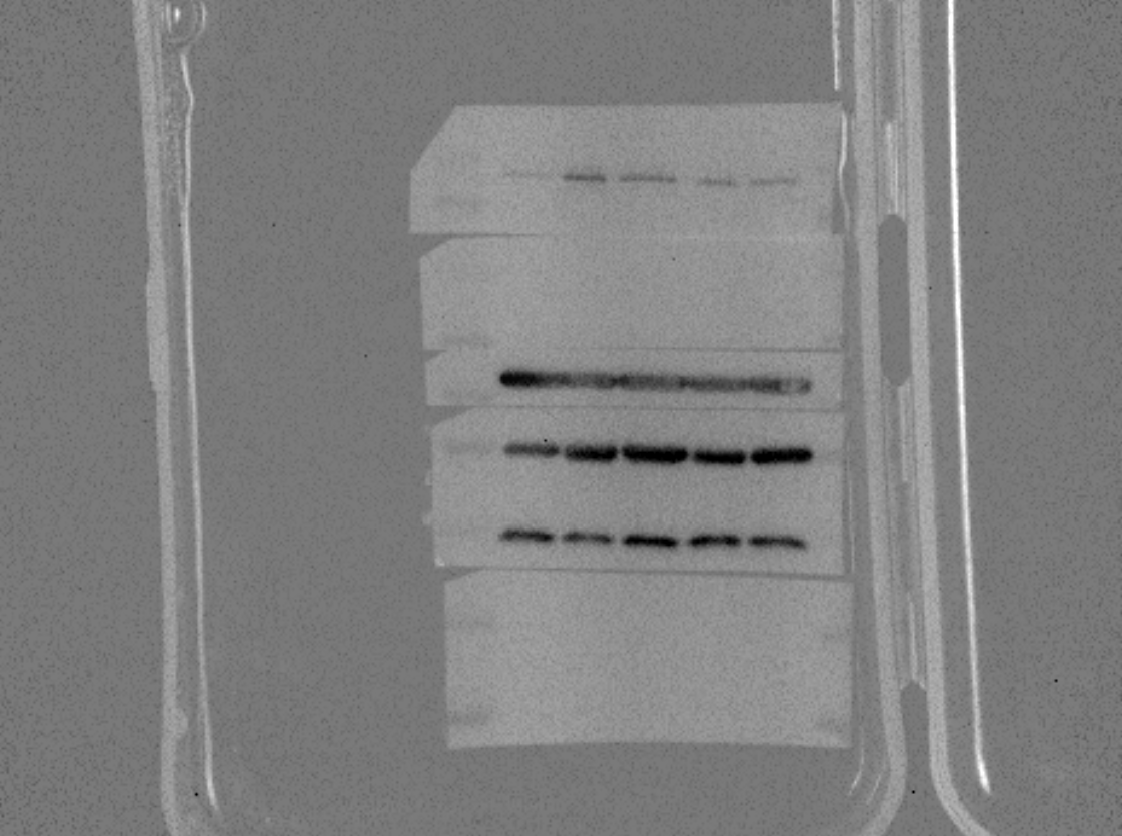

Supplement: S2 File — (ZIP) [file pone.0335225.s007.zip › Cell WB/C N/ZHENGMO 4.tif]

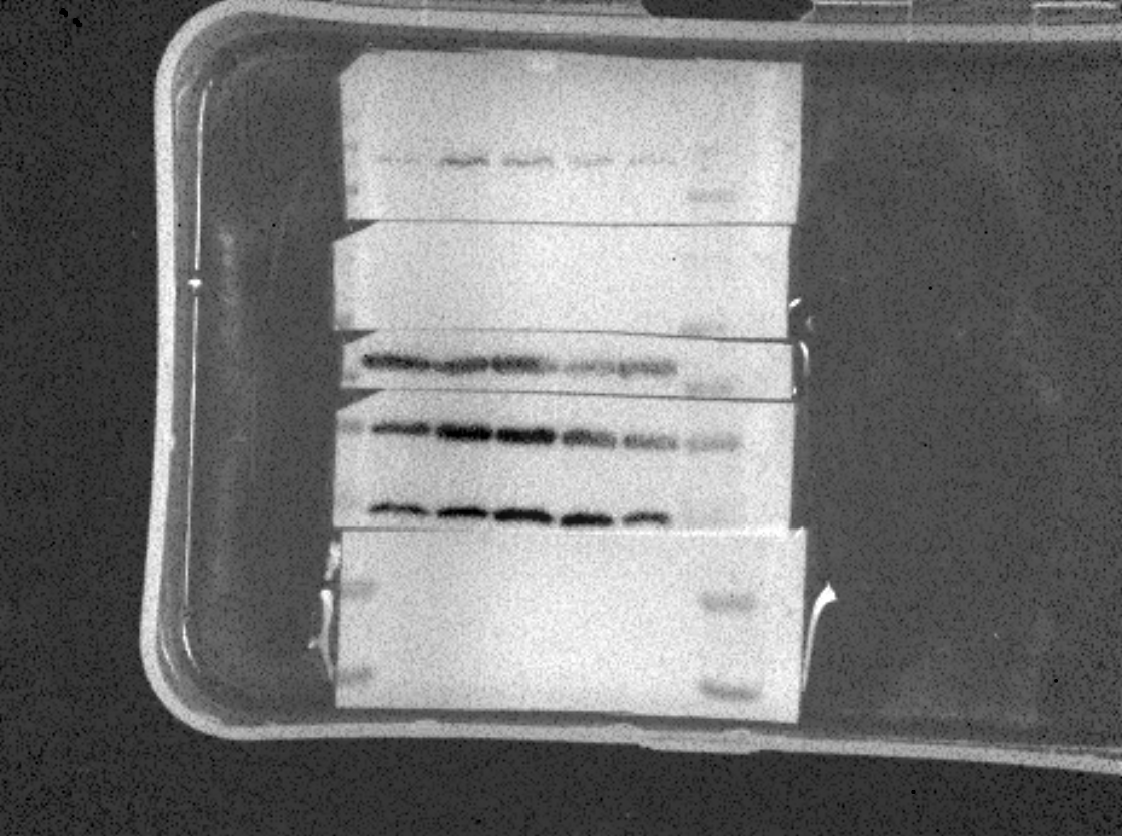

Supplement: S2 File — (ZIP) [file pone.0335225.s007.zip › Cell WB/C N/ZHENGMO 5.tif]

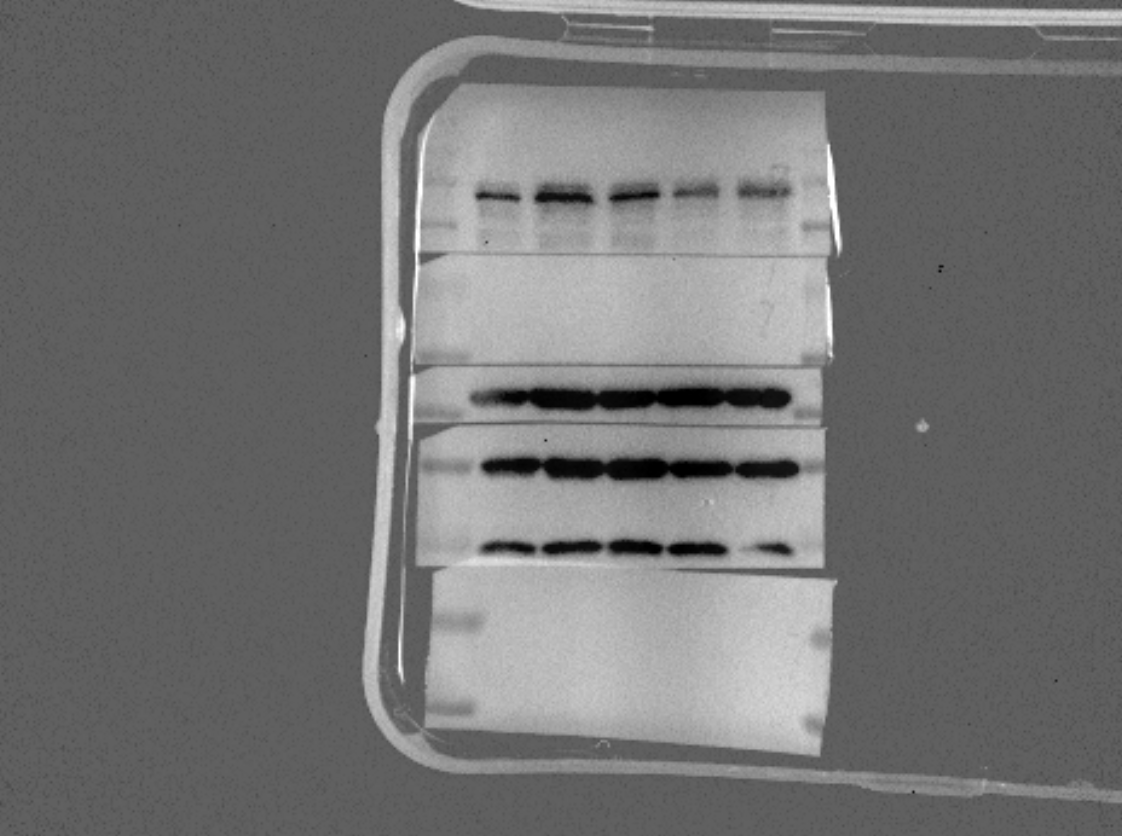

Supplement: S2 File — (ZIP) [file pone.0335225.s007.zip › Cell WB/C N/ZHENGMO 7.tif]

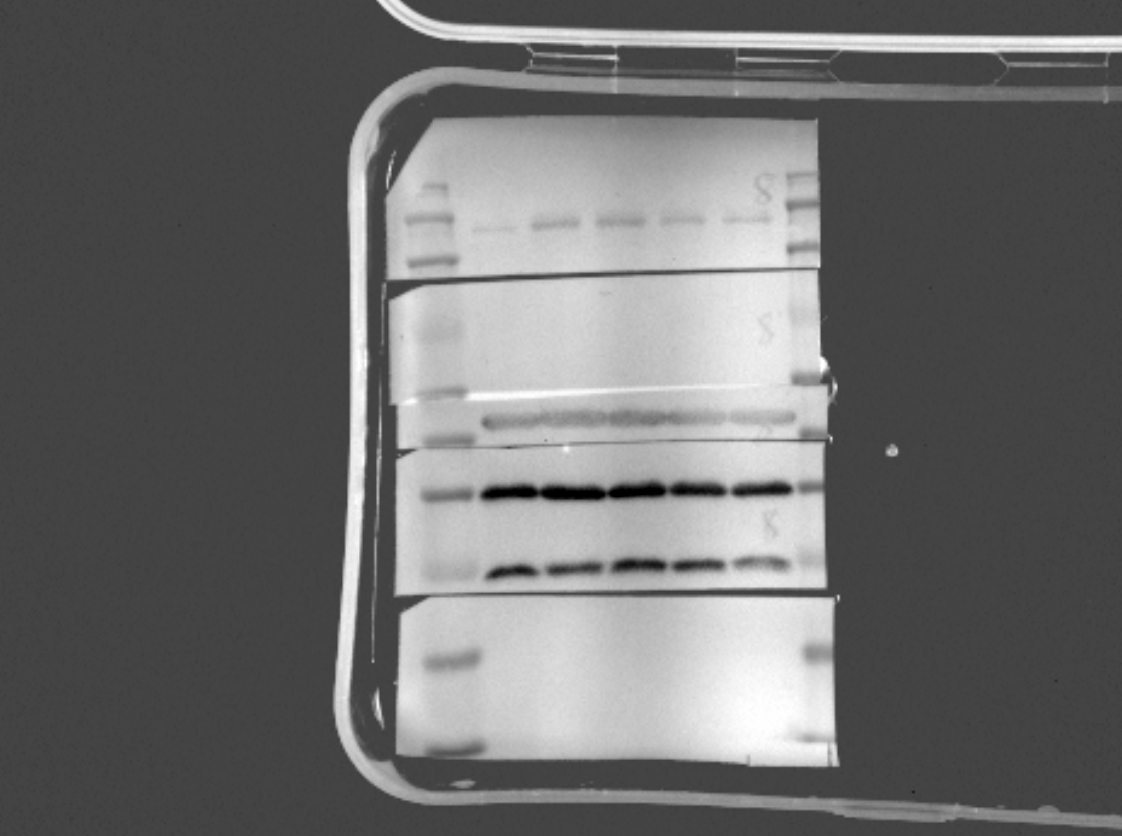

Supplement: S2 File — (ZIP) [file pone.0335225.s007.zip › Cell WB/C N/ZHENGMO 8.tif]

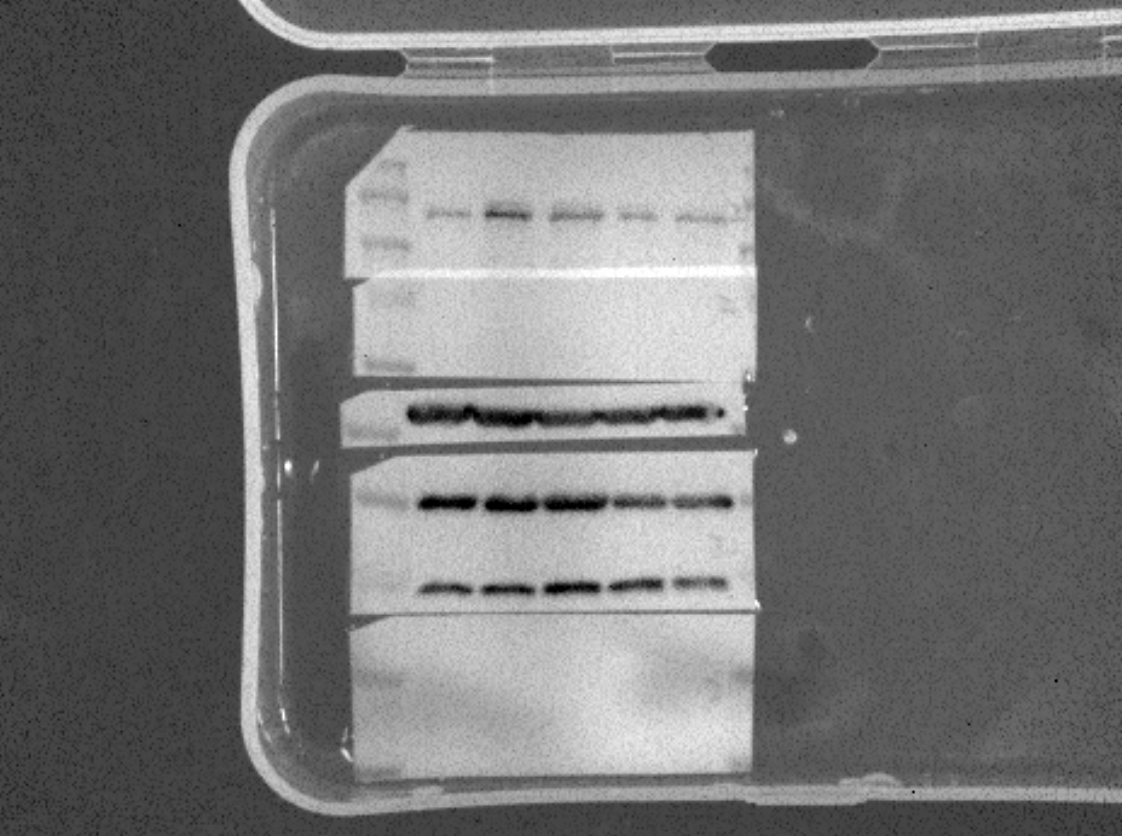

Supplement: S2 File — (ZIP) [file pone.0335225.s007.zip › Cell WB/C N/ZHENMO 2.tif]

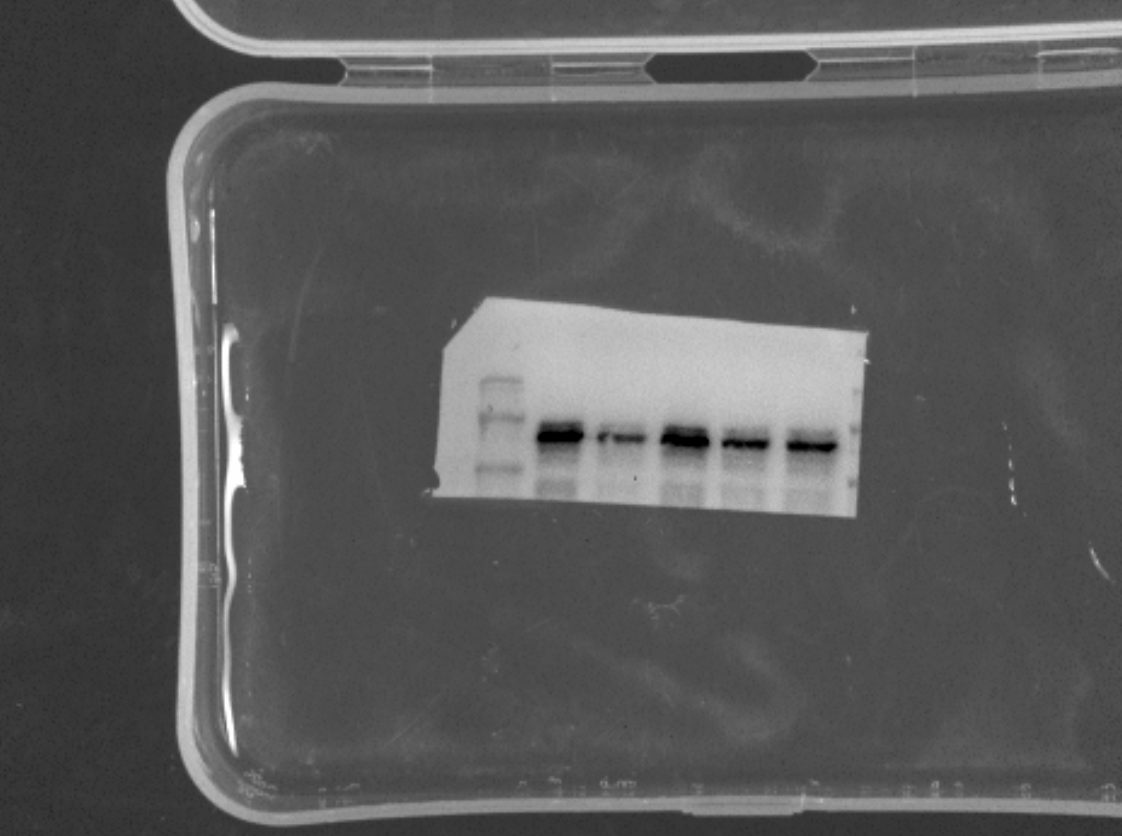

Supplement: S2 File — (ZIP) [file pone.0335225.s007.zip › Cell WB/E/E1/E-cadhrein 14_1s 1 hb.tif]

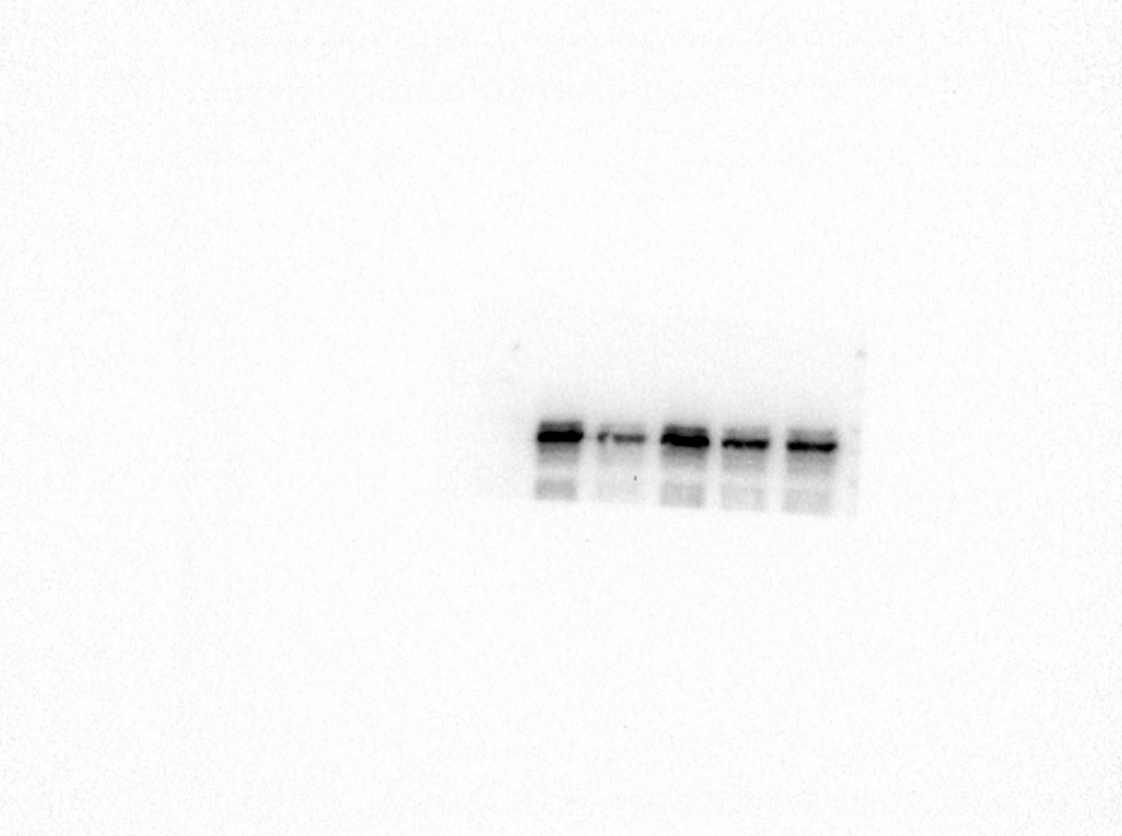

Supplement: S2 File — (ZIP) [file pone.0335225.s007.zip › Cell WB/E/E1/E-cadhrein 14_1s 1.tif]

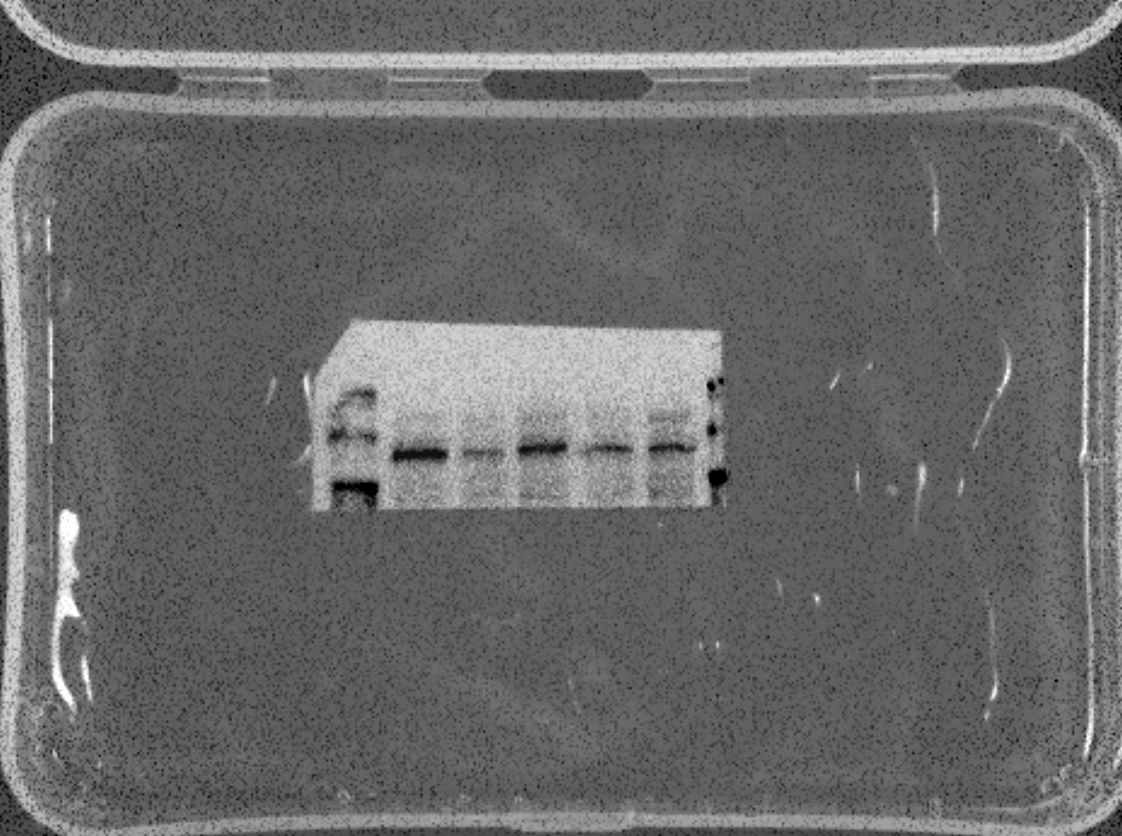

Supplement: S2 File — (ZIP) [file pone.0335225.s007.zip › Cell WB/E/E1/E-cadhrein 14_1s 3 hb.tif]

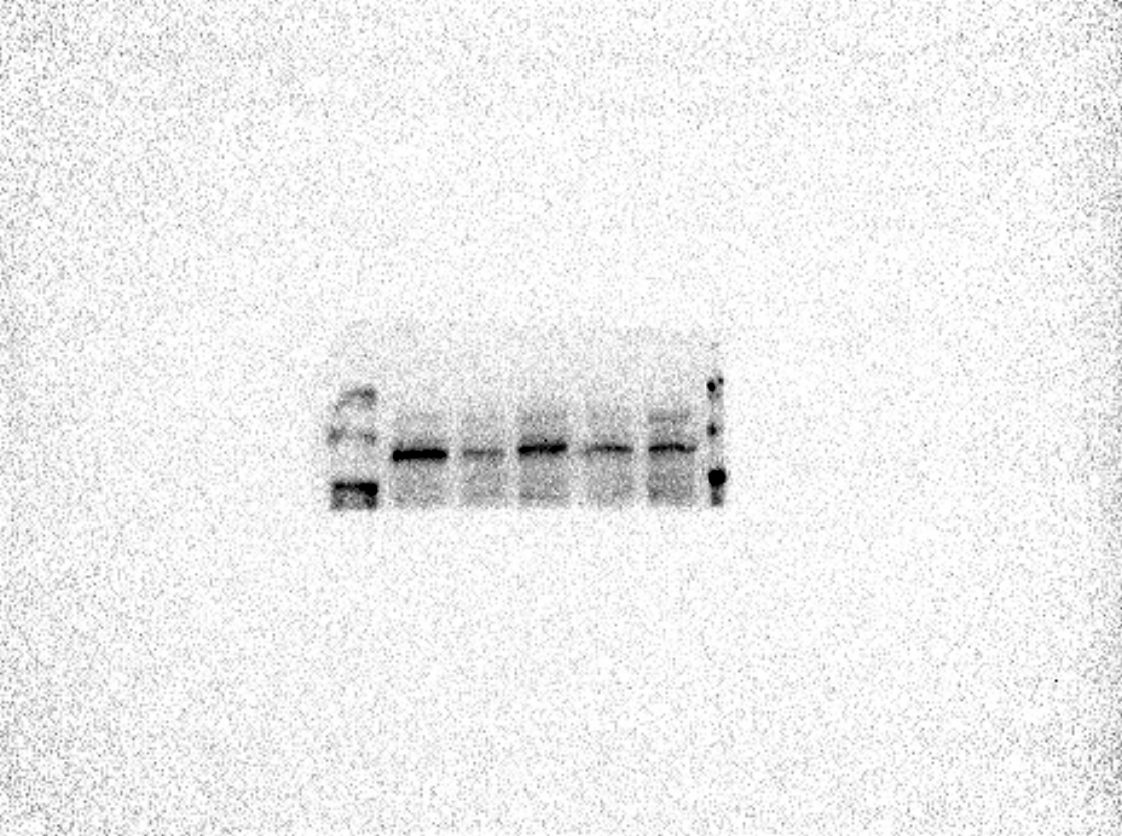

Supplement: S2 File — (ZIP) [file pone.0335225.s007.zip › Cell WB/E/E1/E-cadhrein 14_1s 3.tif]

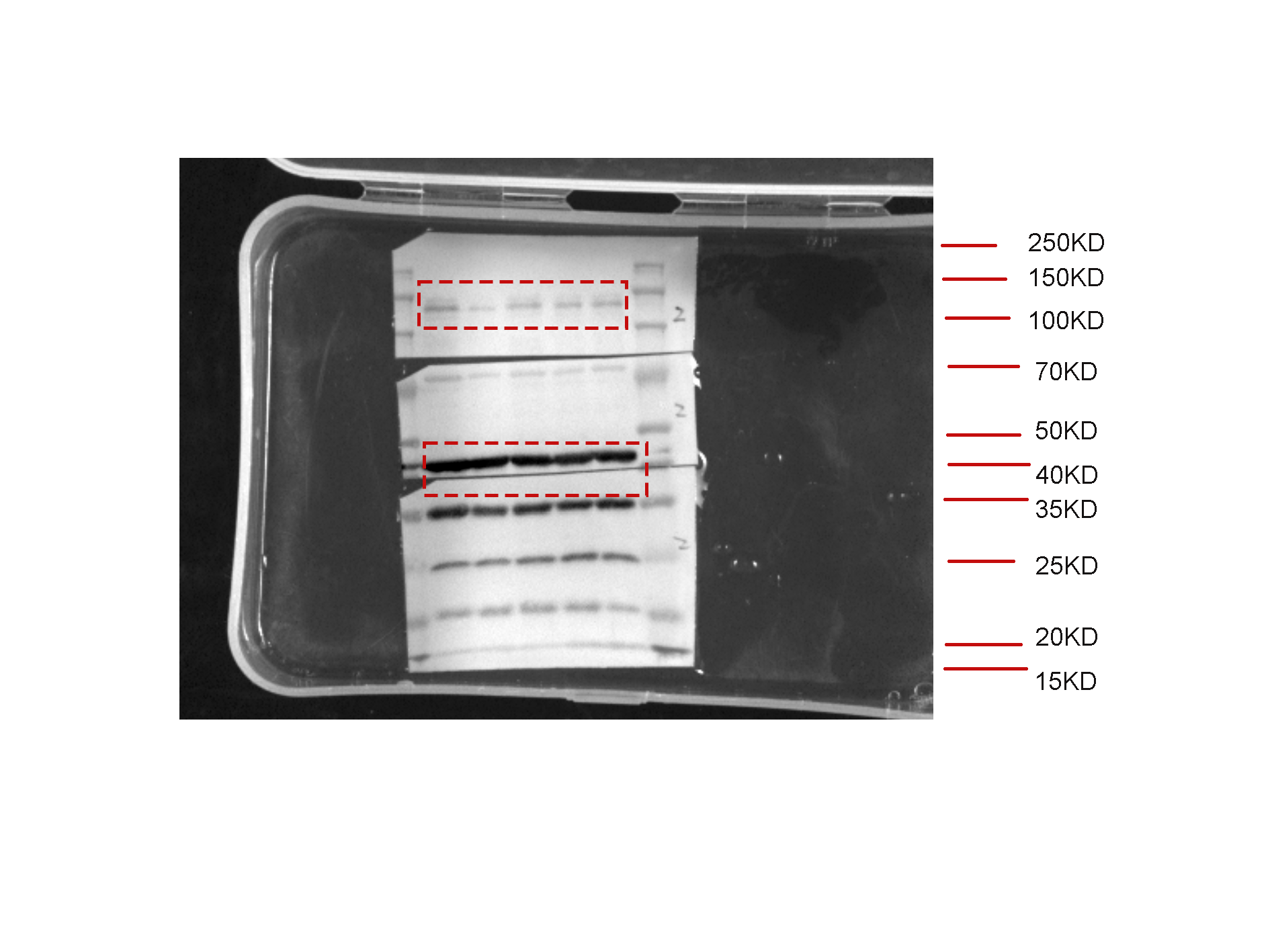

Supplement: S2 File — (ZIP) [file pone.0335225.s007.zip › Cell WB/E/E1/细胞 E.tif]

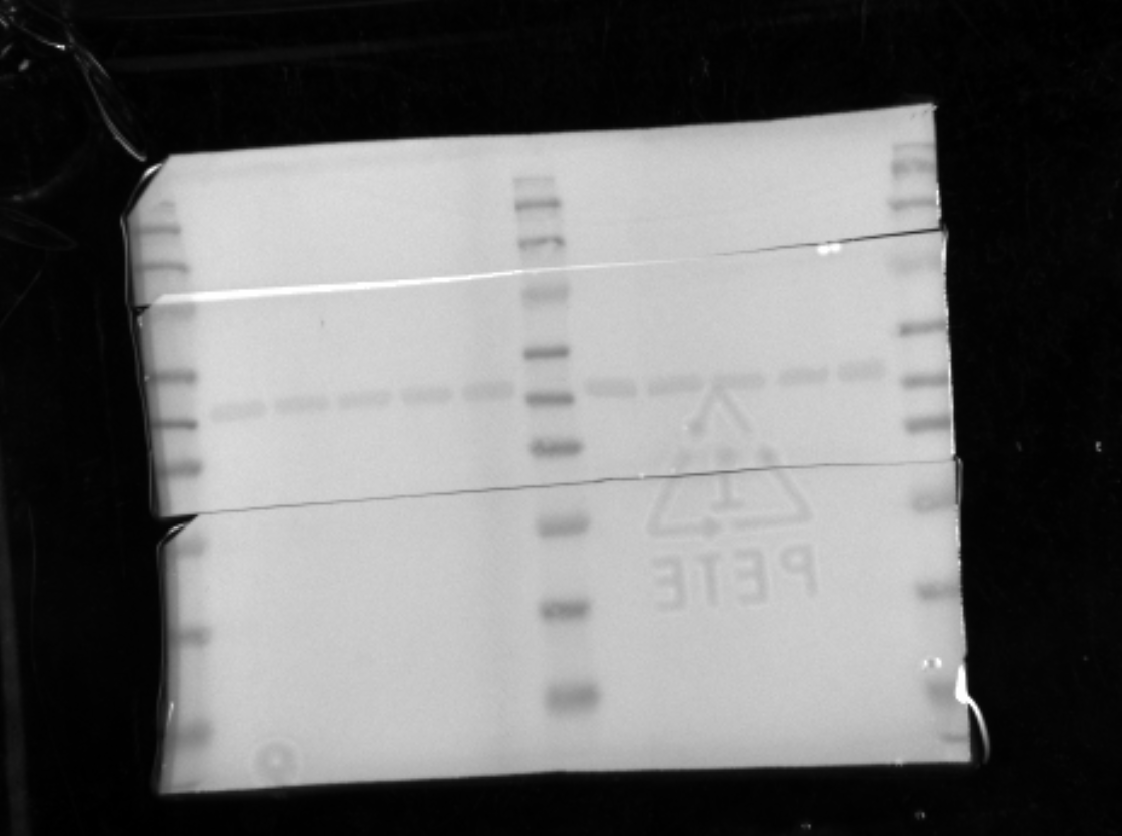

Supplement: S2 File — (ZIP) [file pone.0335225.s007.zip › Cell WB/E/E2/maker.tif]

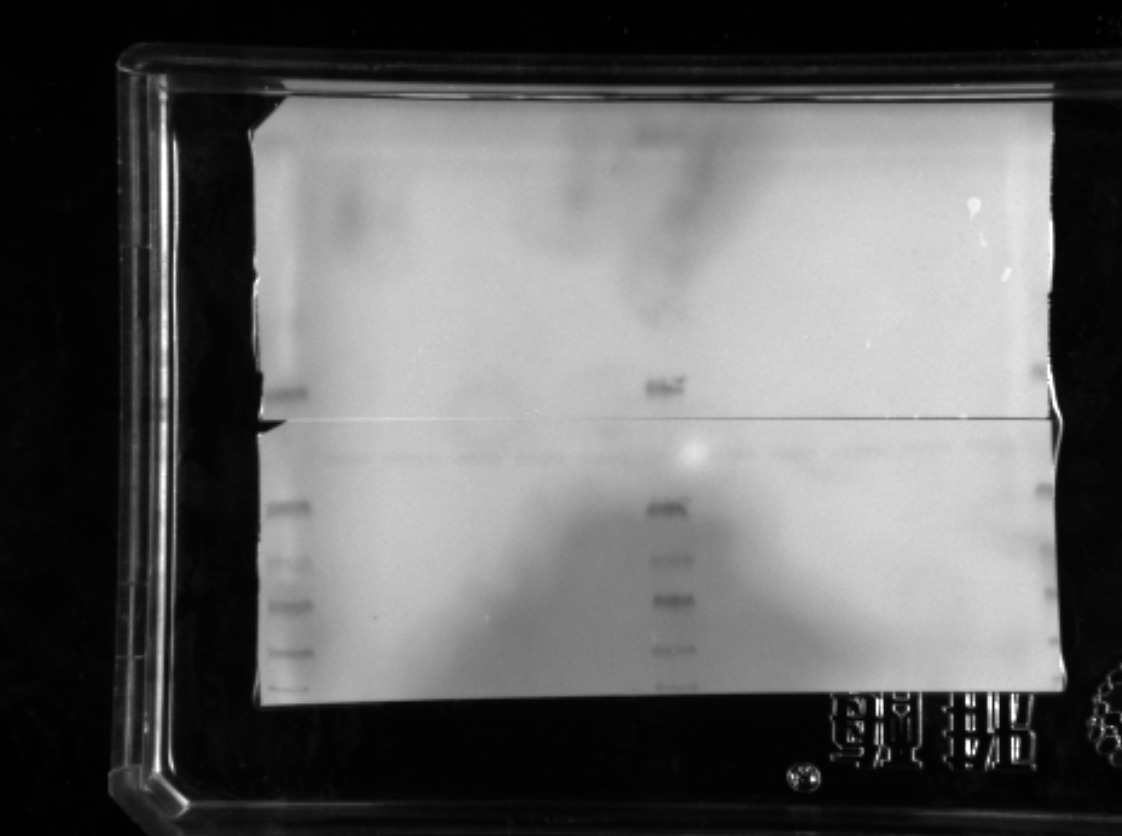

Supplement: S2 File — (ZIP) [file pone.0335225.s007.zip › Cell WB/PPAR/MAKER 2 PPAR.tif]

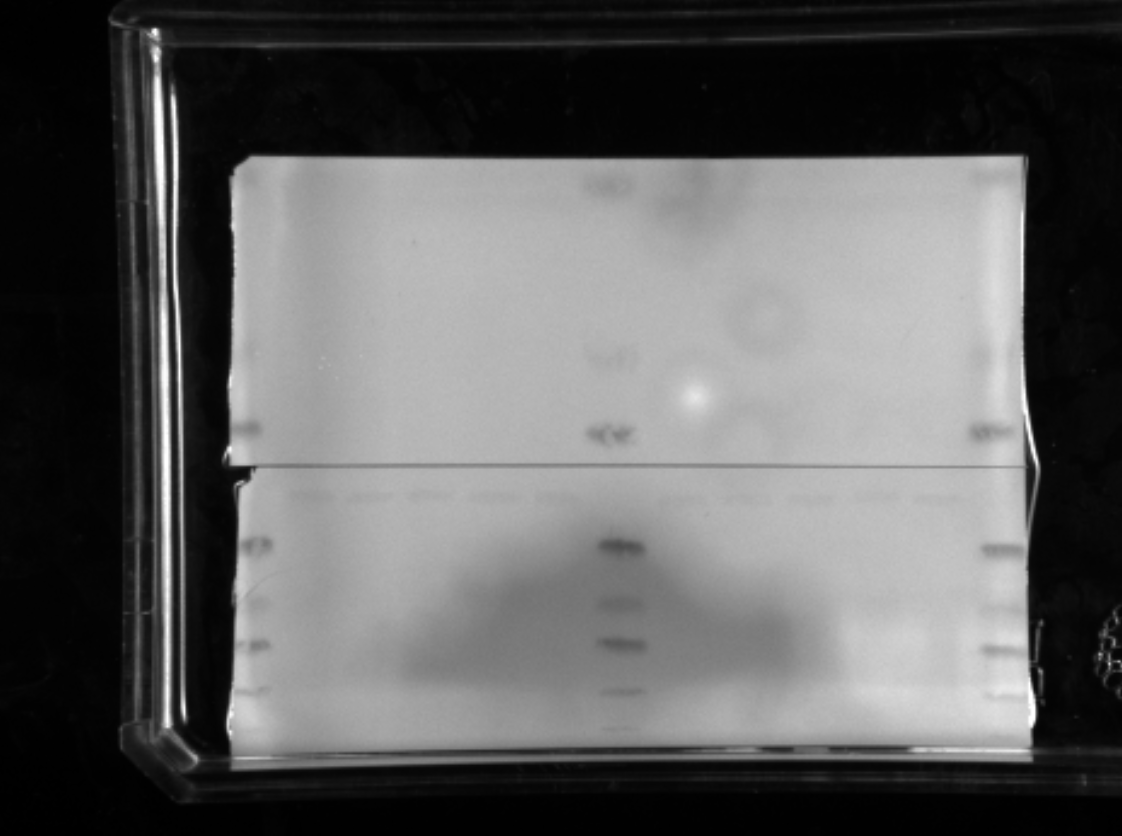

Supplement: S2 File — (ZIP) [file pone.0335225.s007.zip › Cell WB/PPAR/MAKER-PPAR 1.tif]

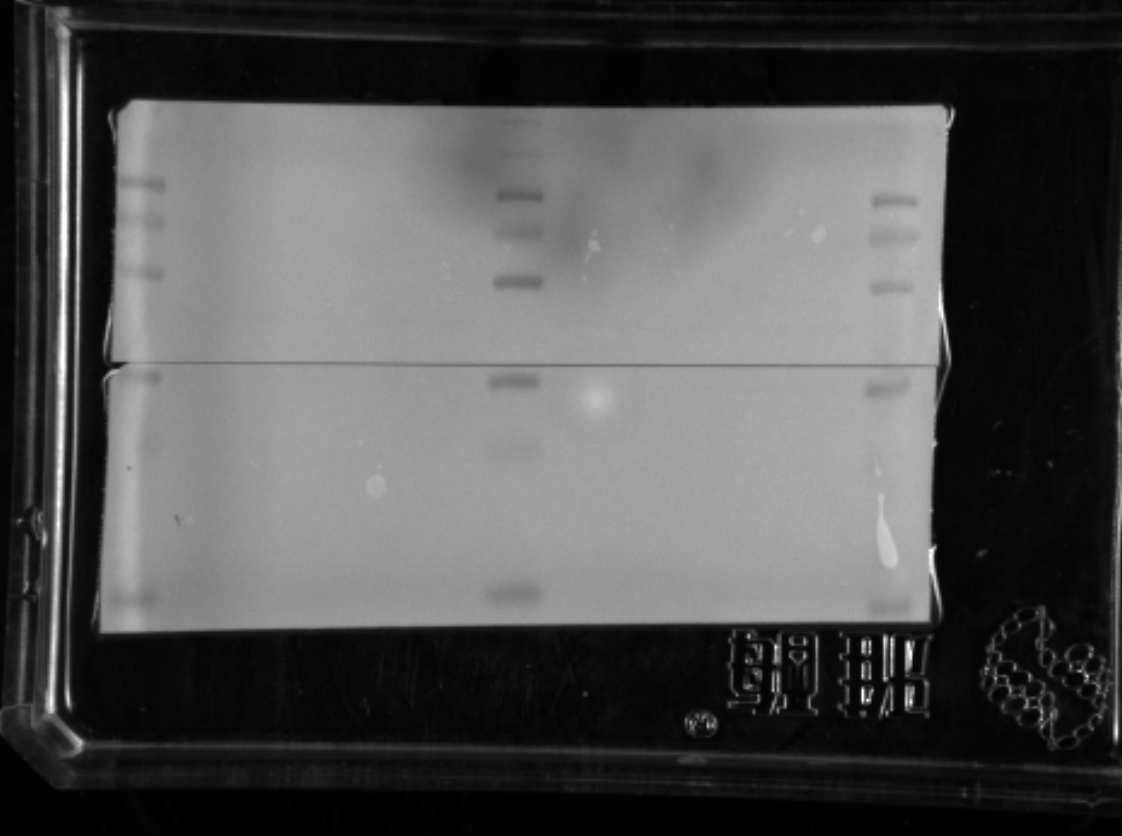

Supplement: S2 File — (ZIP) [file pone.0335225.s007.zip › Cell WB/TGF/maker2.tif]

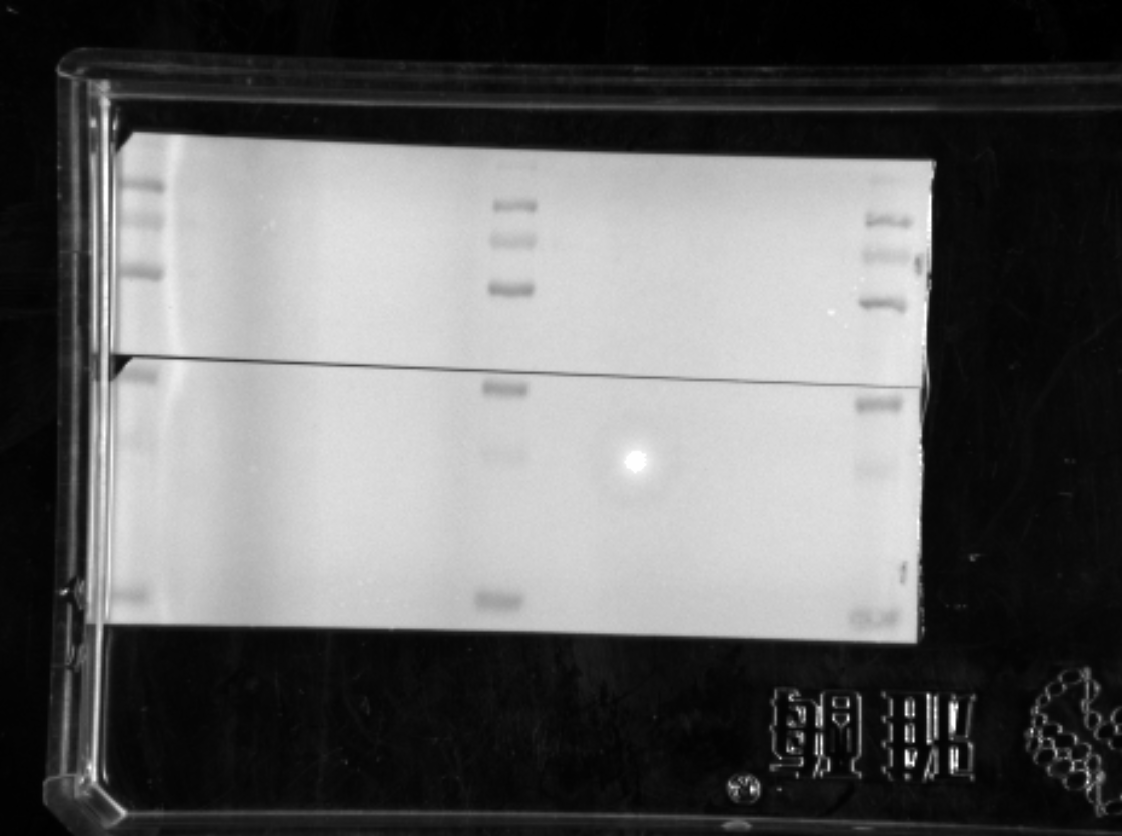

Supplement: S2 File — (ZIP) [file pone.0335225.s007.zip › Cell WB/TGF/图 maker1.tif]

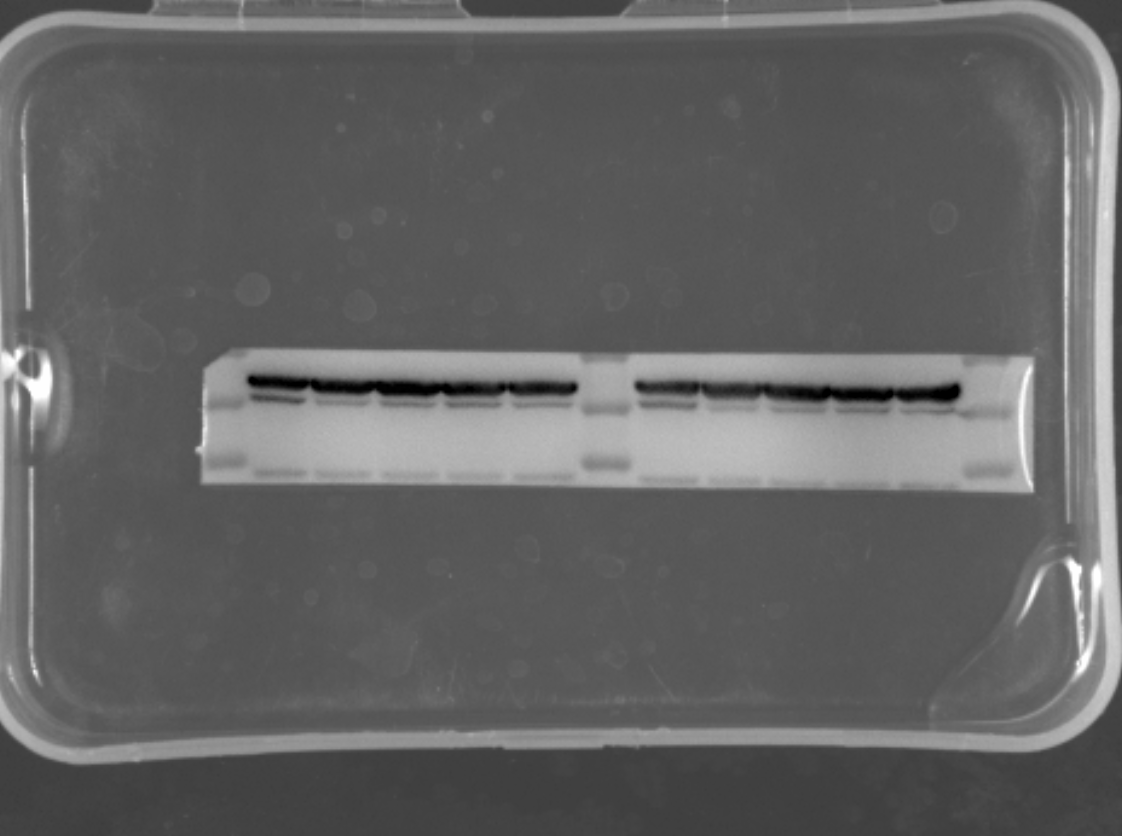

Supplement: S2 File — (ZIP) [file pone.0335225.s007.zip › Cell WB/V/actin 1 1s hb.tif]

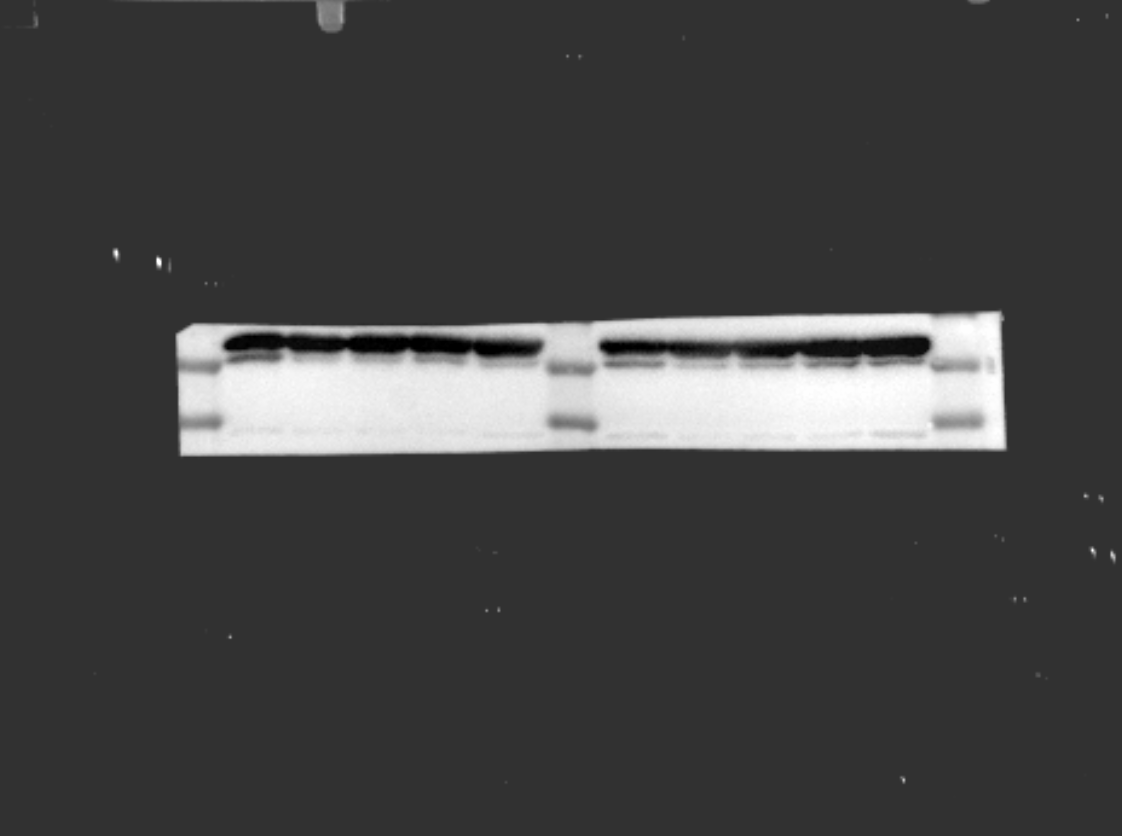

Supplement: S2 File — (ZIP) [file pone.0335225.s007.zip › Cell WB/V/actin 2 0_5s hb.tif]

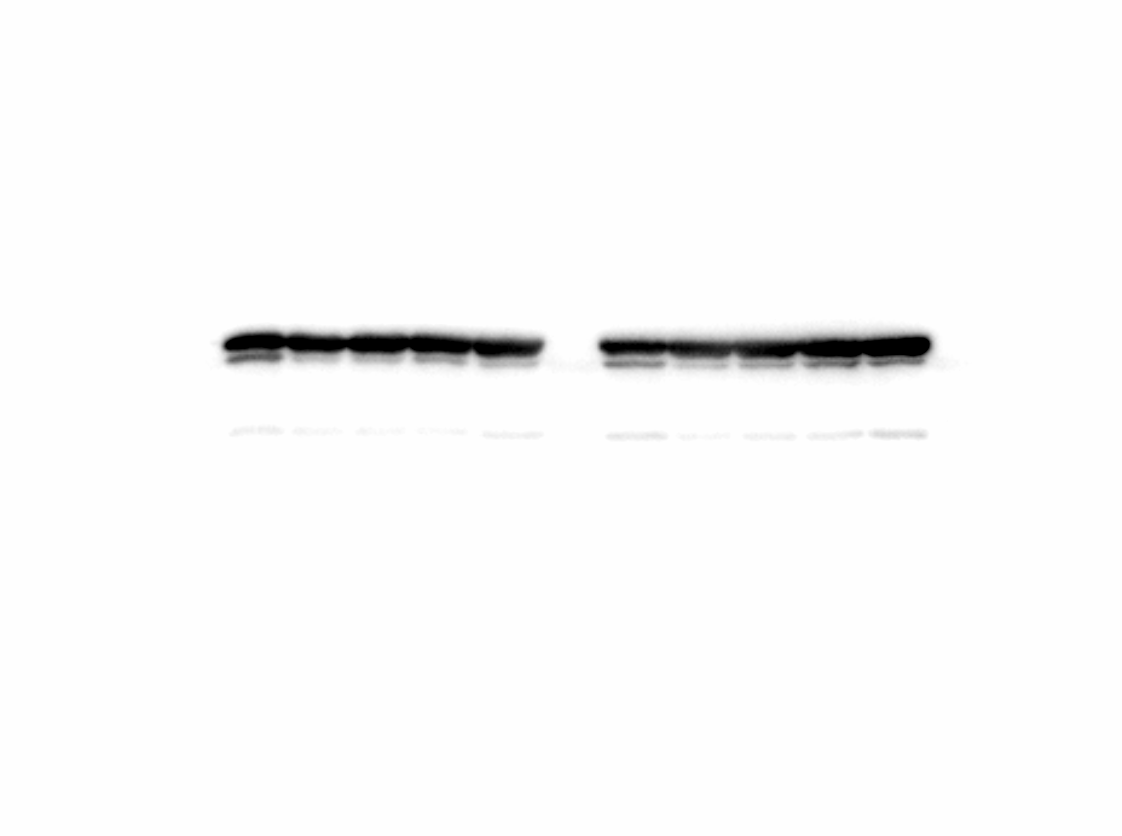

Supplement: S2 File — (ZIP) [file pone.0335225.s007.zip › Cell WB/V/actin2 0_5s.tif]

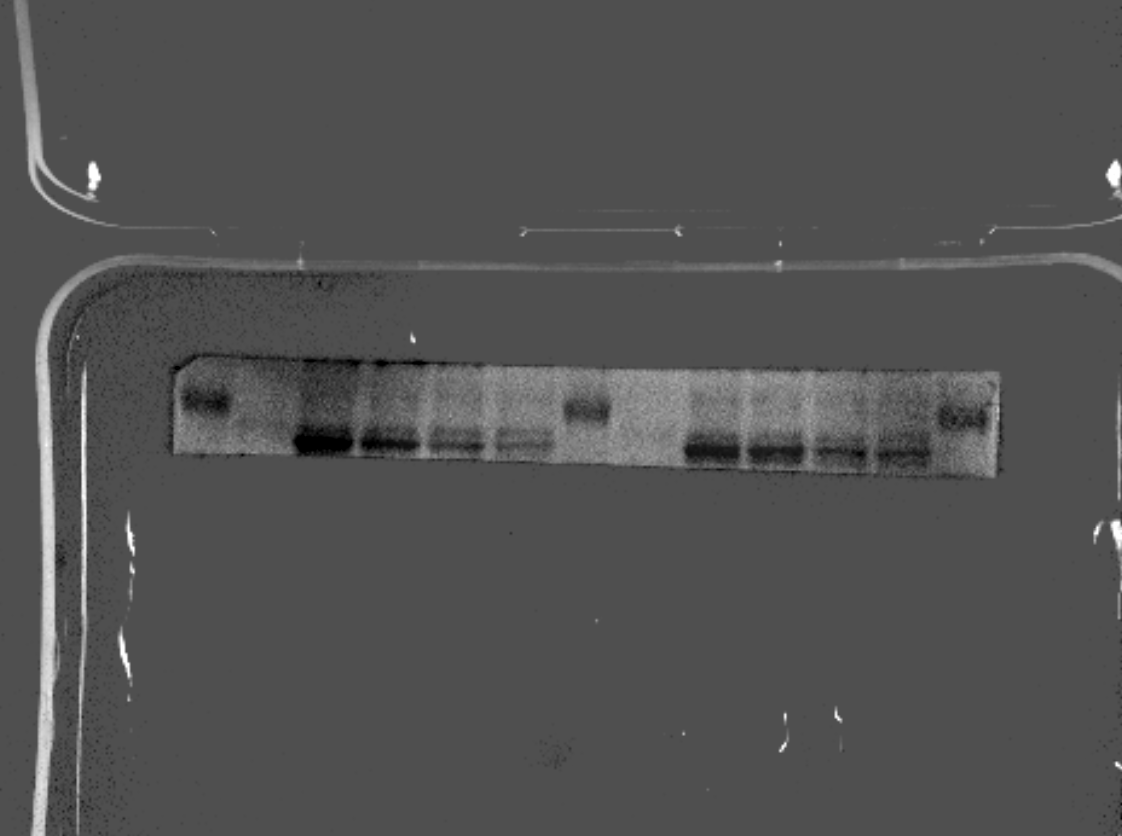

Supplement: S2 File — (ZIP) [file pone.0335225.s007.zip › Cell WB/V/vimentin 2 24_6 hb.tif]

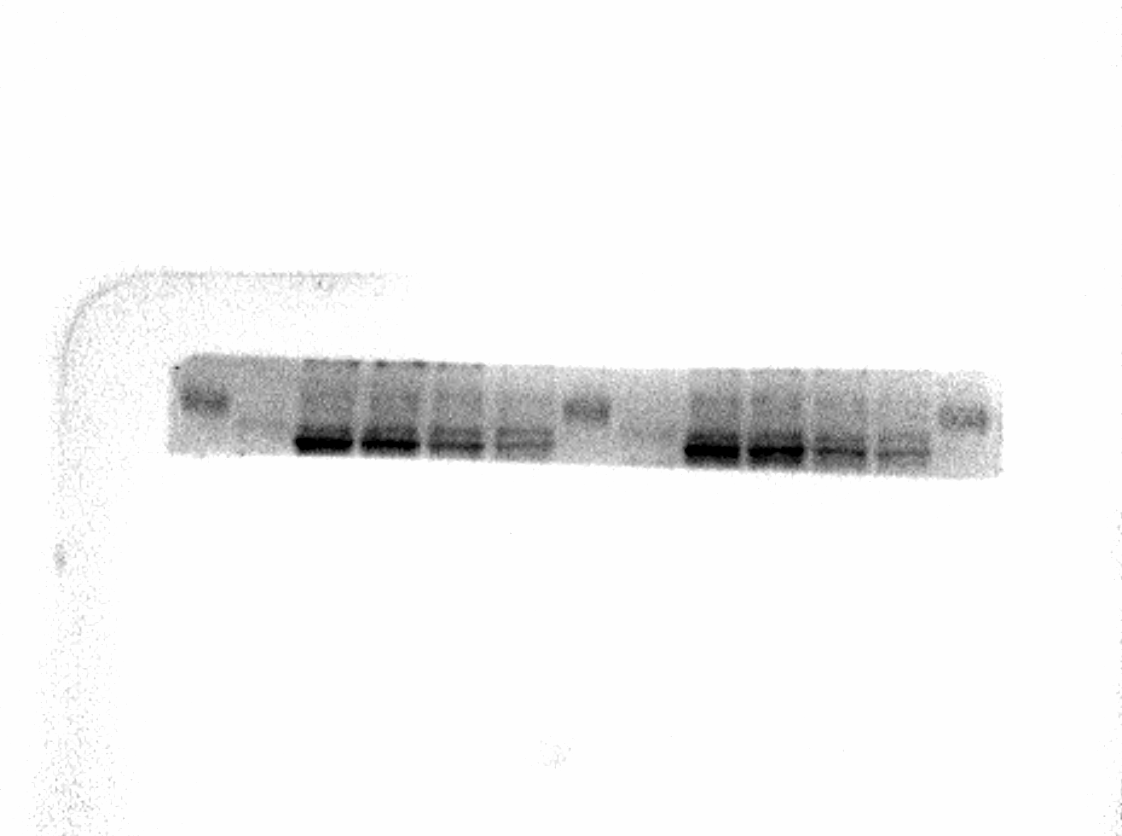

Supplement: S2 File — (ZIP) [file pone.0335225.s007.zip › Cell WB/V/vimentin 2 24_6s.tif]

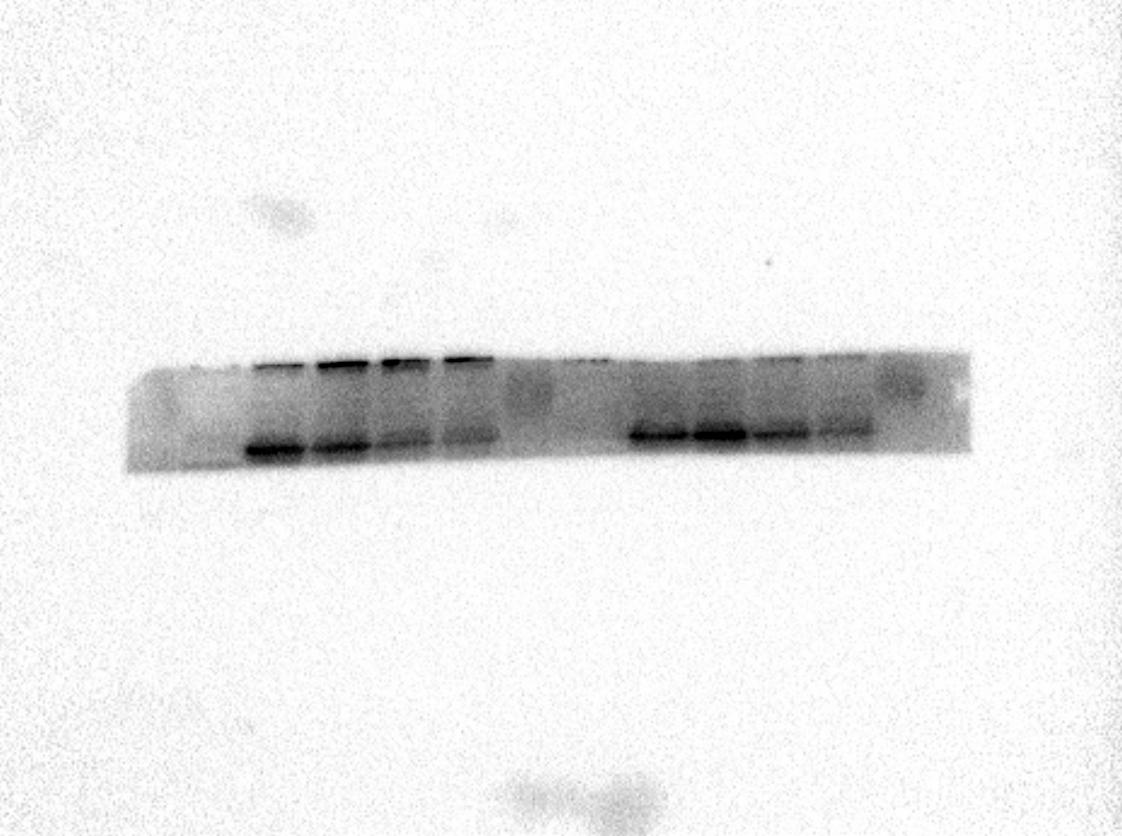

Supplement: S2 File — (ZIP) [file pone.0335225.s007.zip › Cell WB/V/vimentin2 14_7s.tif]

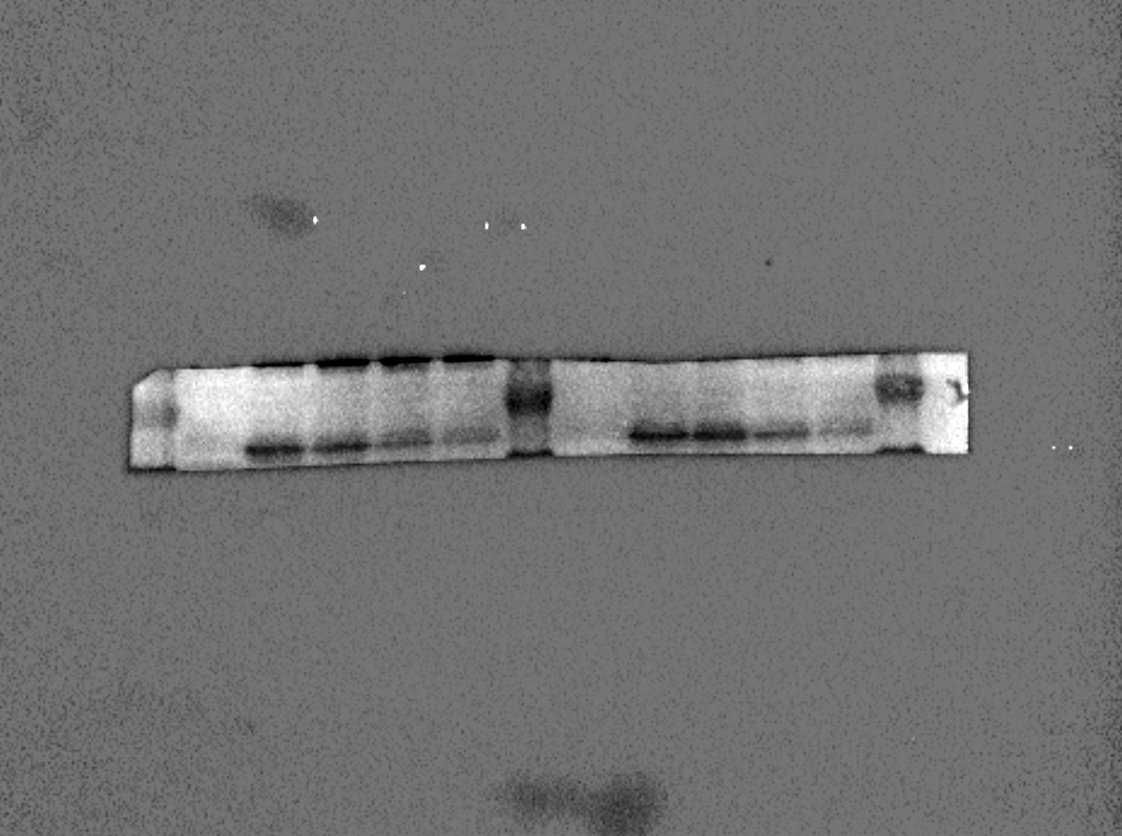

Supplement: S2 File — (ZIP) [file pone.0335225.s007.zip › Cell WB/V/vimentin2 14_7 hb.tif]

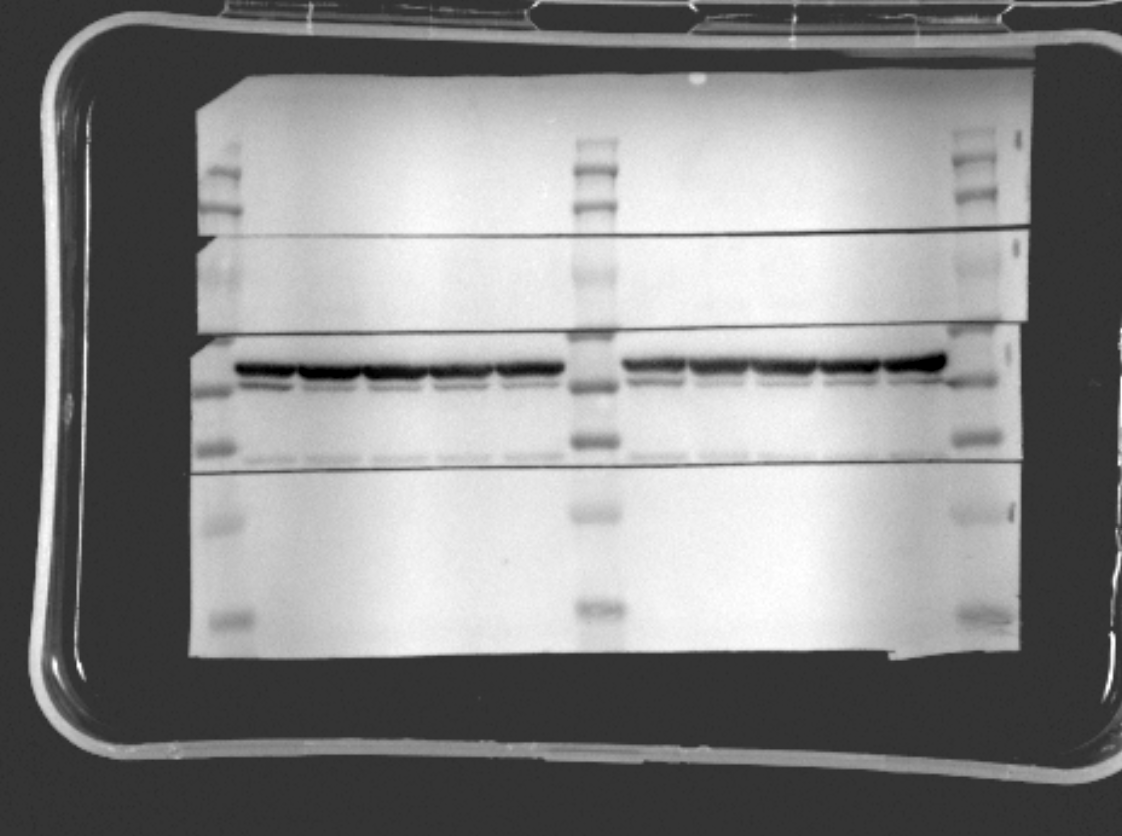

Supplement: S2 File — (ZIP) [file pone.0335225.s007.zip › Cell WB/V/zhengmo 1 actin.tif]

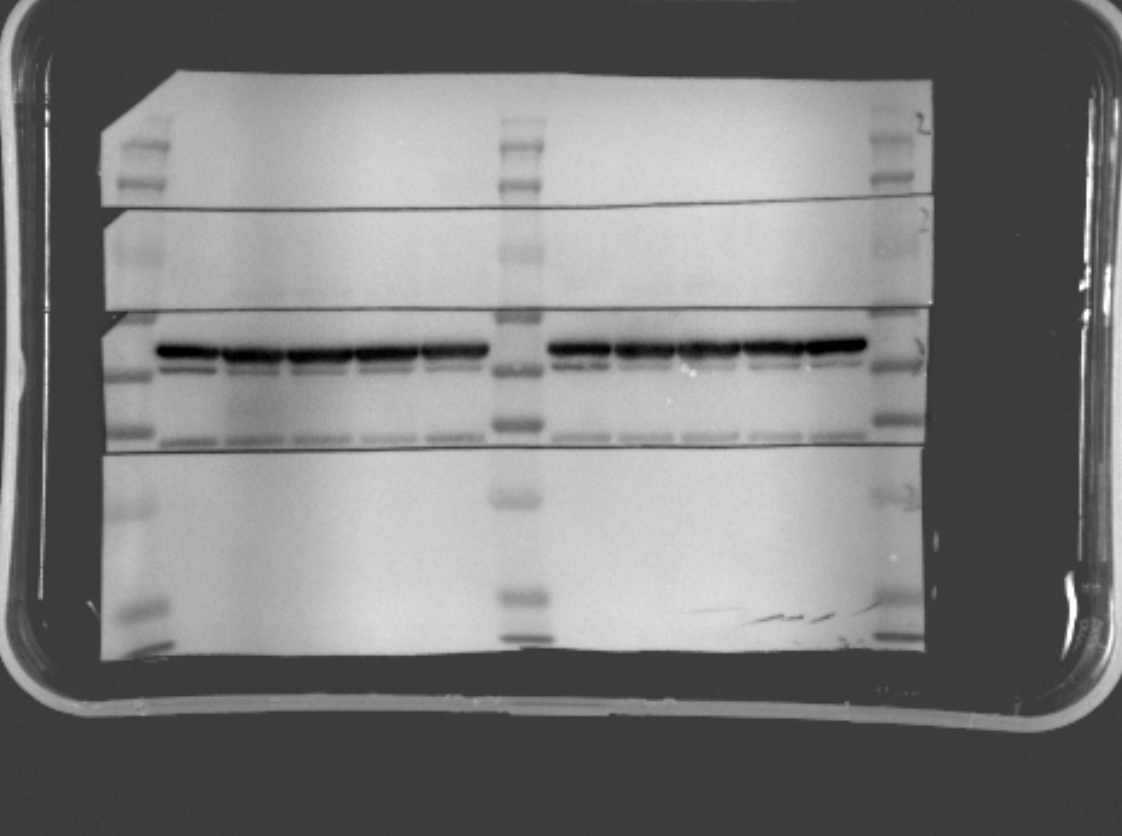

Supplement: S2 File — (ZIP) [file pone.0335225.s007.zip › Cell WB/V/zhengmo 2 actin.tif]

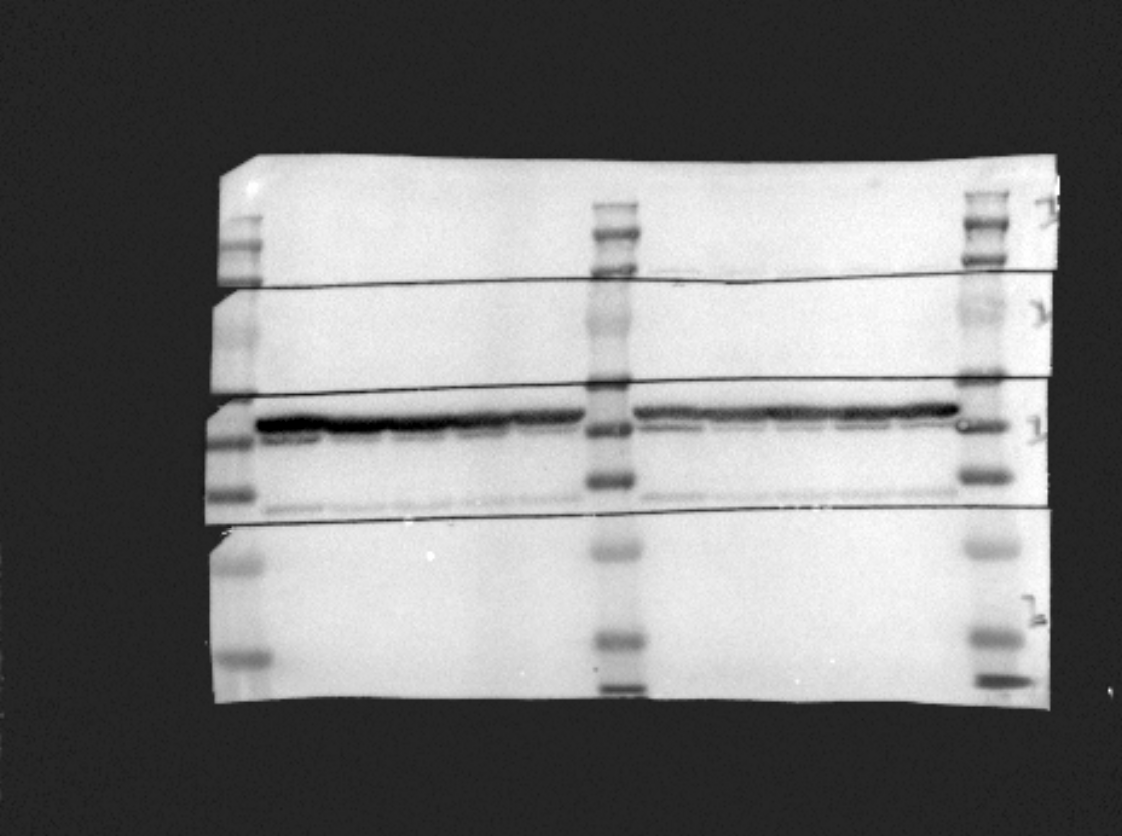

Supplement: S2 File — (ZIP) [file pone.0335225.s007.zip › Cell WB/V/zhengmo 2.tif]

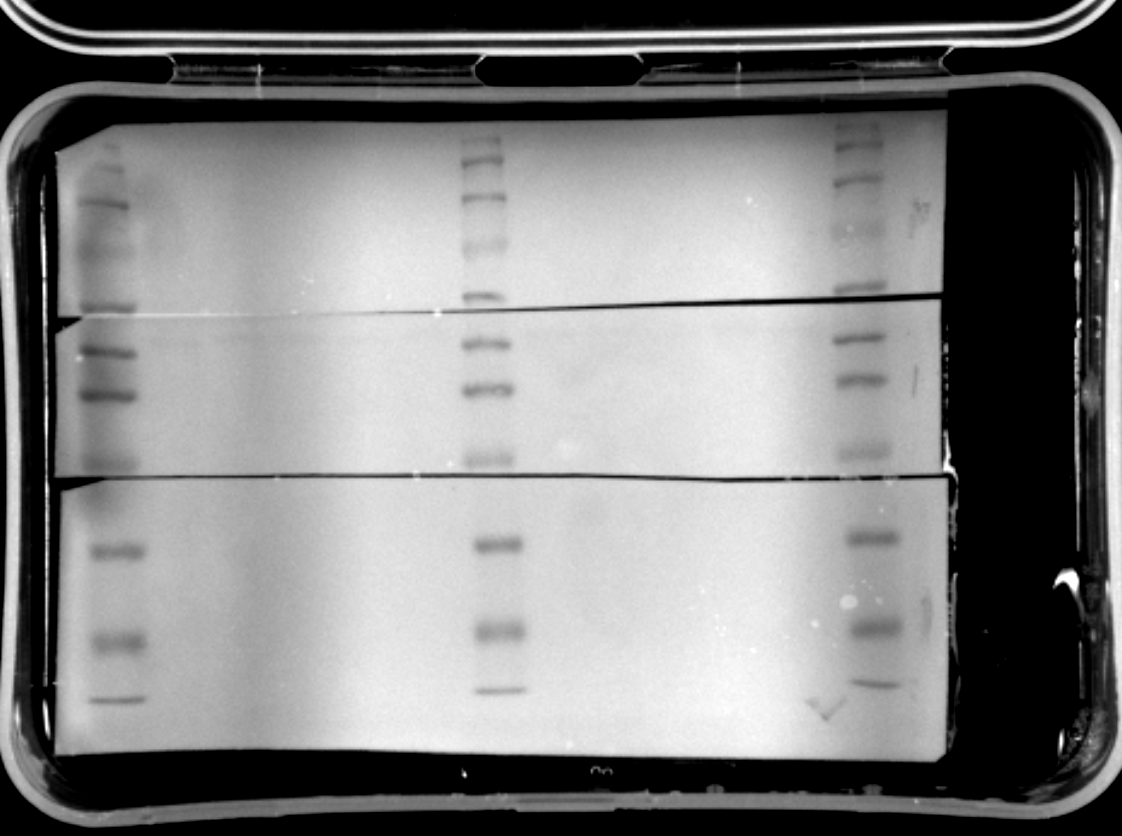

Supplement: S2 File — (ZIP) [file pone.0335225.s007.zip › Cell WB/a/A1/maker 1.tif]

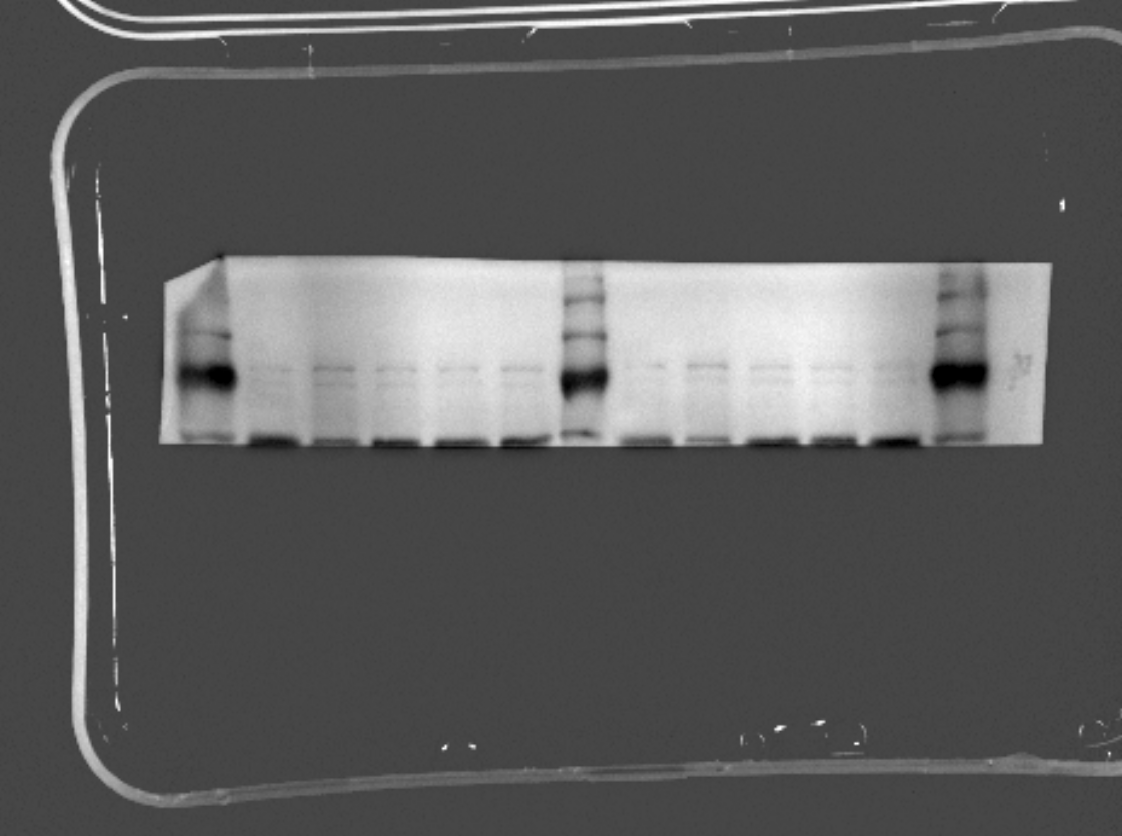

Supplement: S2 File — (ZIP) [file pone.0335225.s007.zip › Cell WB/a/A1/tubblin 23_6s 1 hb.tif]

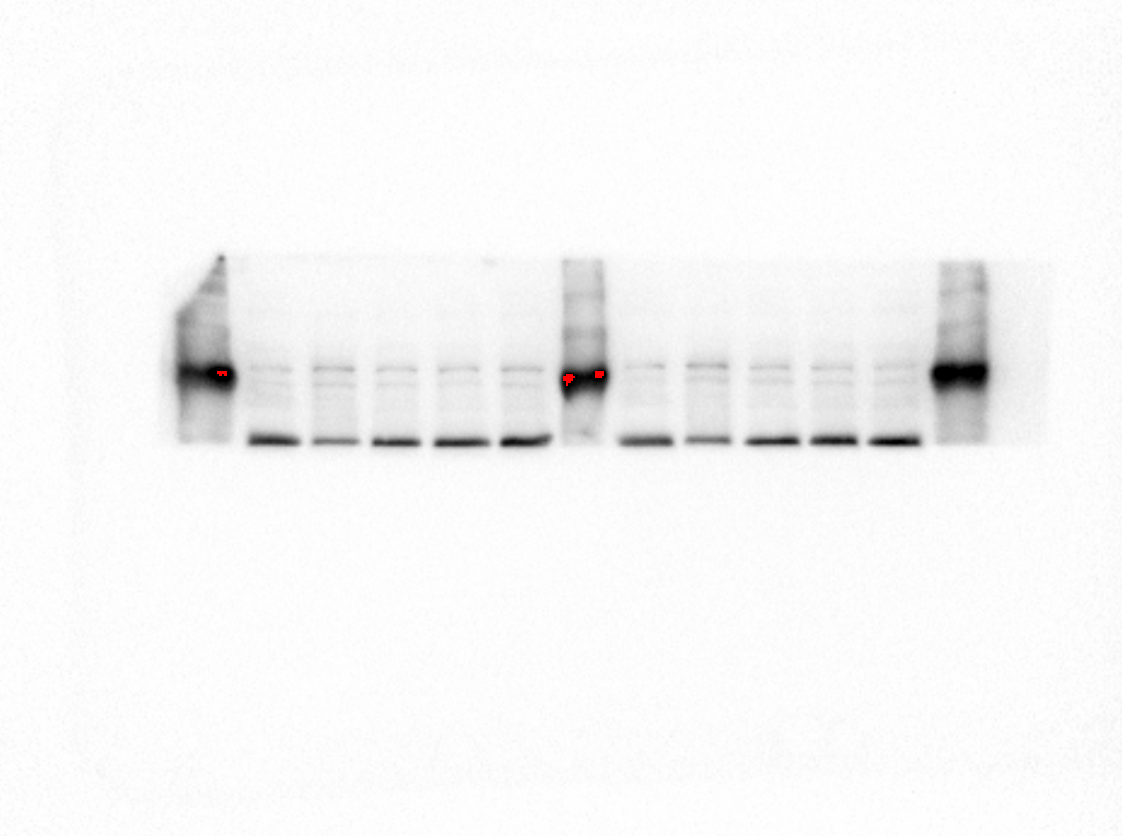

Supplement: S2 File — (ZIP) [file pone.0335225.s007.zip › Cell WB/a/A1/tubblin 23_6s 1.tif]

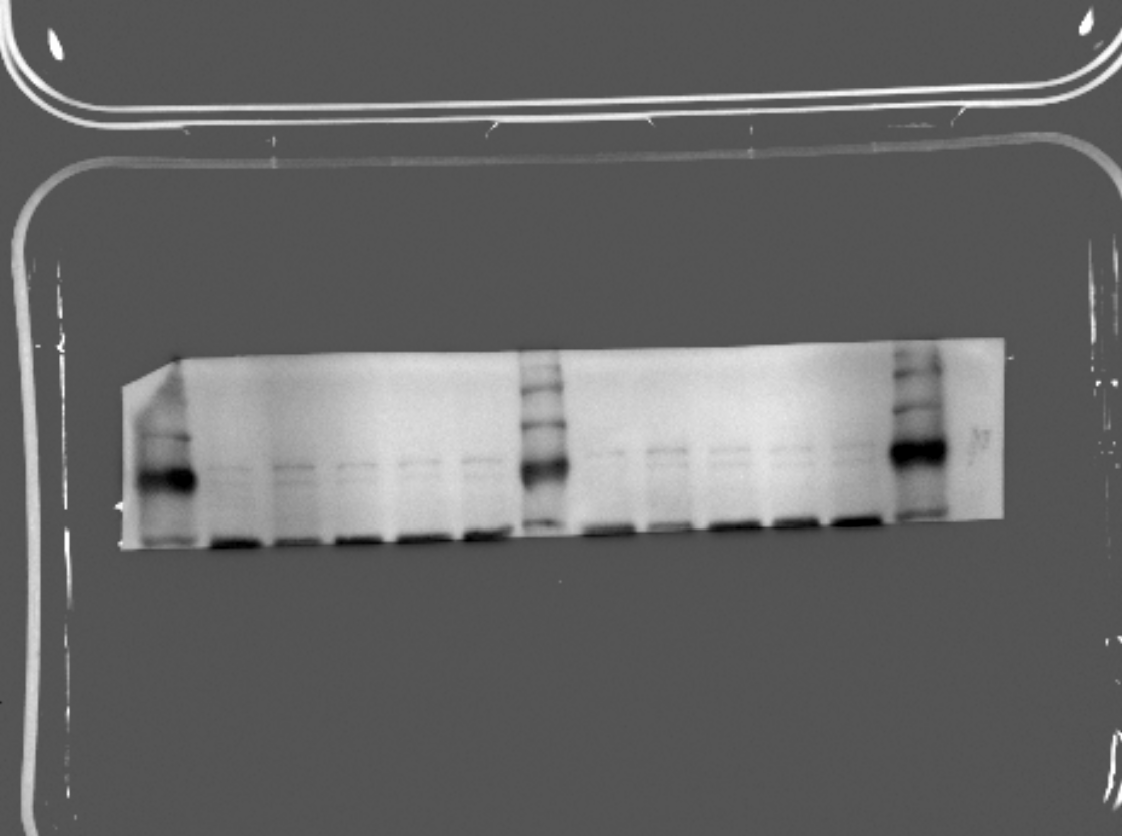

Supplement: S2 File — (ZIP) [file pone.0335225.s007.zip › Cell WB/a/A1/tublin 10_7s hb.tif]

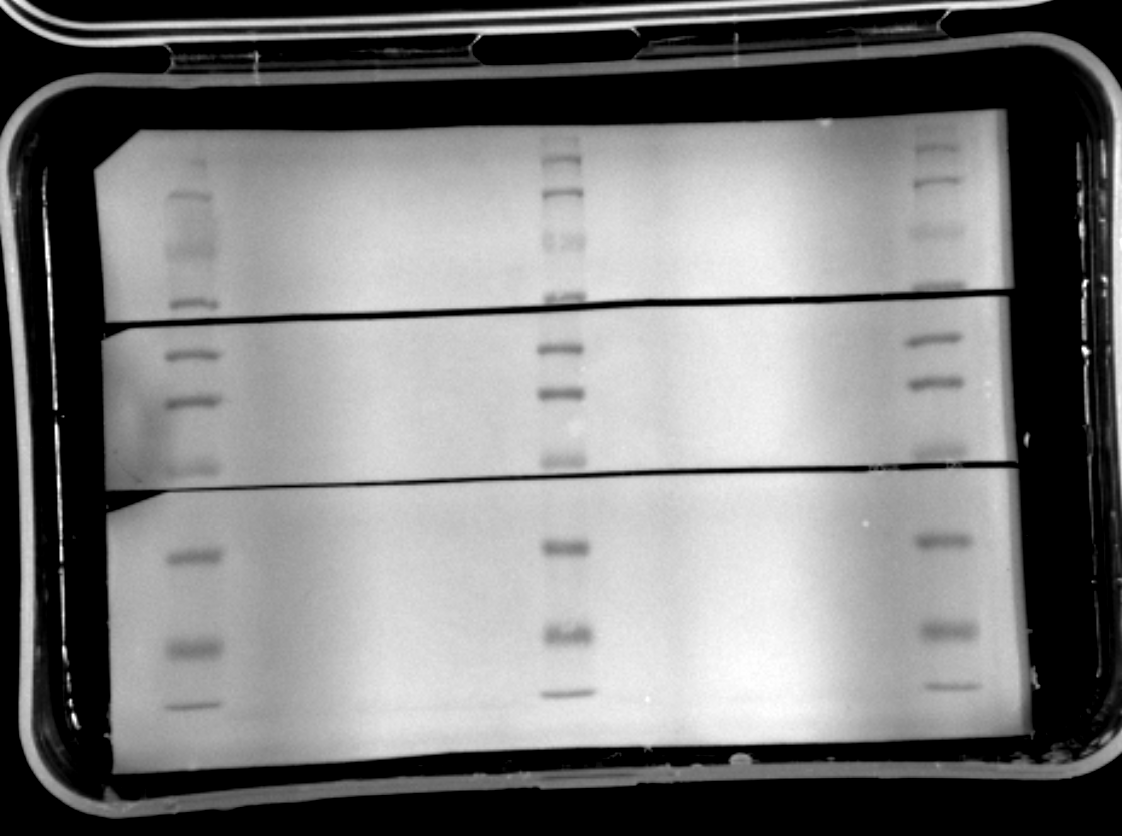

Supplement: S2 File — (ZIP) [file pone.0335225.s007.zip › Cell WB/a/A1/图 maker 2.tif]

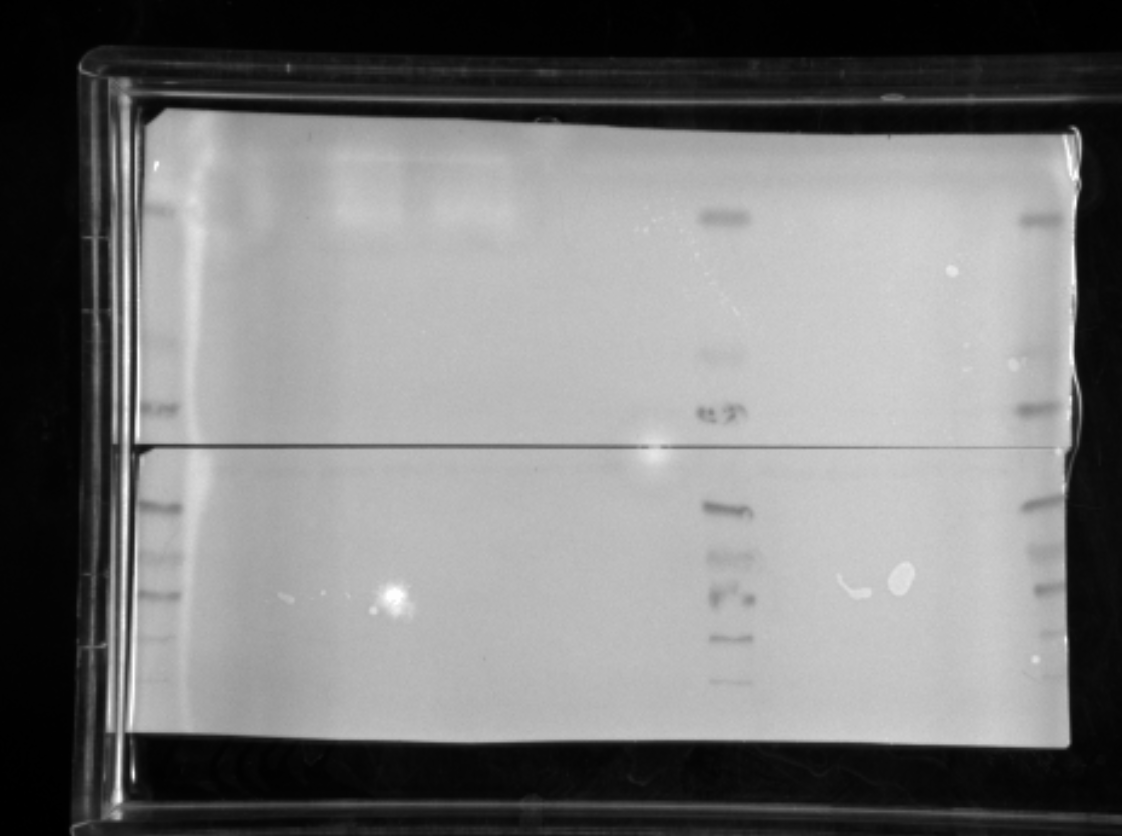

Supplement: S2 File — (ZIP) [file pone.0335225.s007.zip › Cell WB/p-SMAD/Smad.1/图maker2.tif]

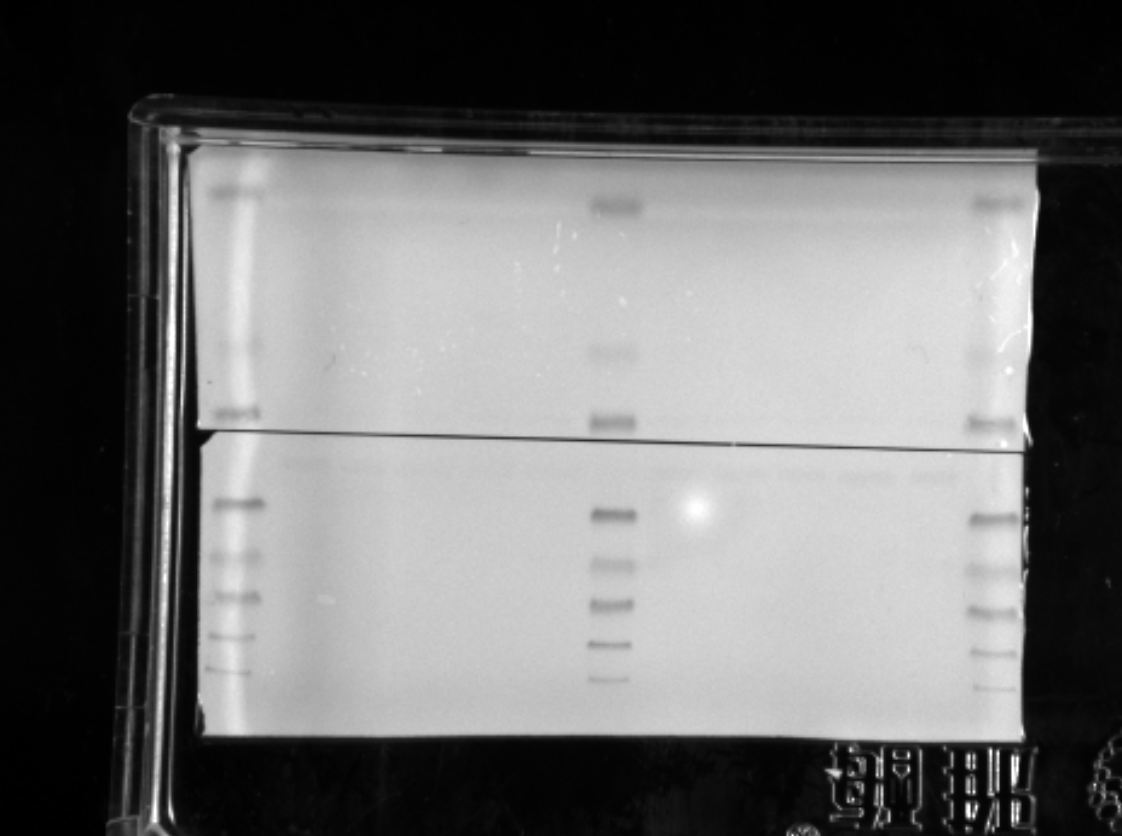

Supplement: S2 File — (ZIP) [file pone.0335225.s007.zip › Cell WB/p-SMAD/Smad2/maker-samd2.tif]

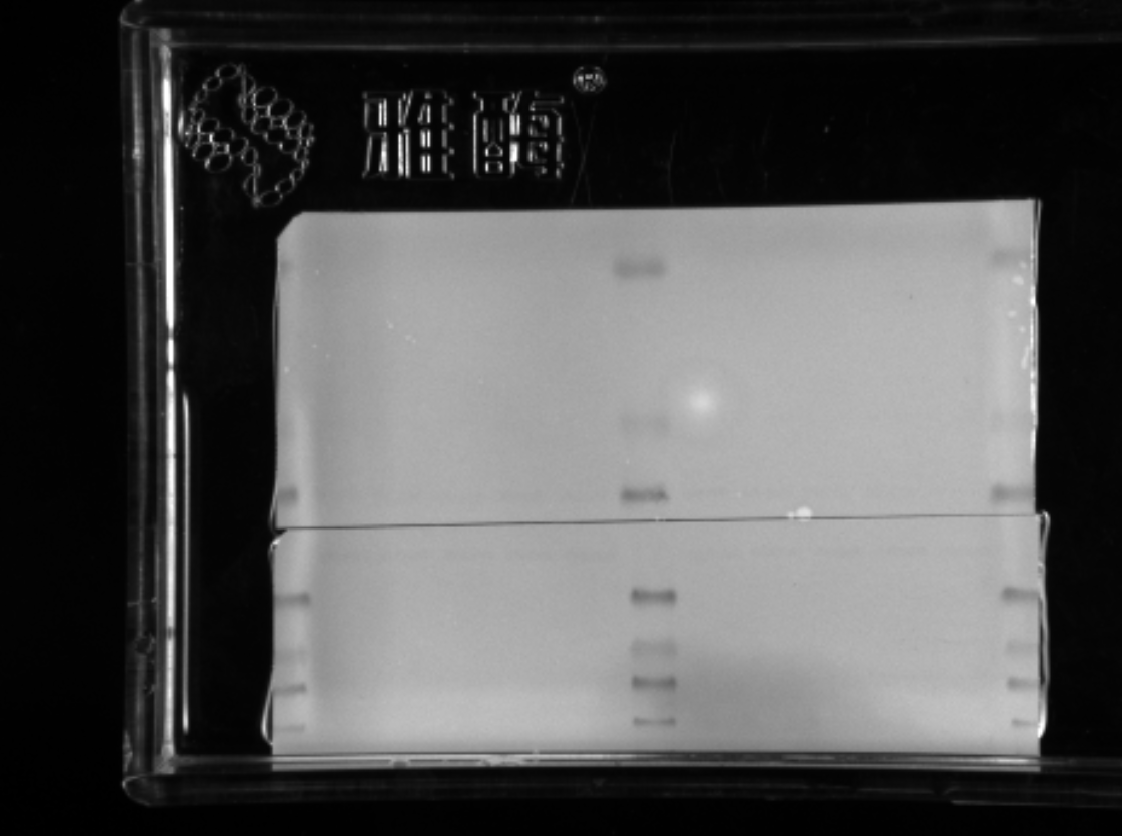

Supplement: S2 File — (ZIP) [file pone.0335225.s007.zip › Cell WB/p-SMAD/p-SMAD/maker4.tif]

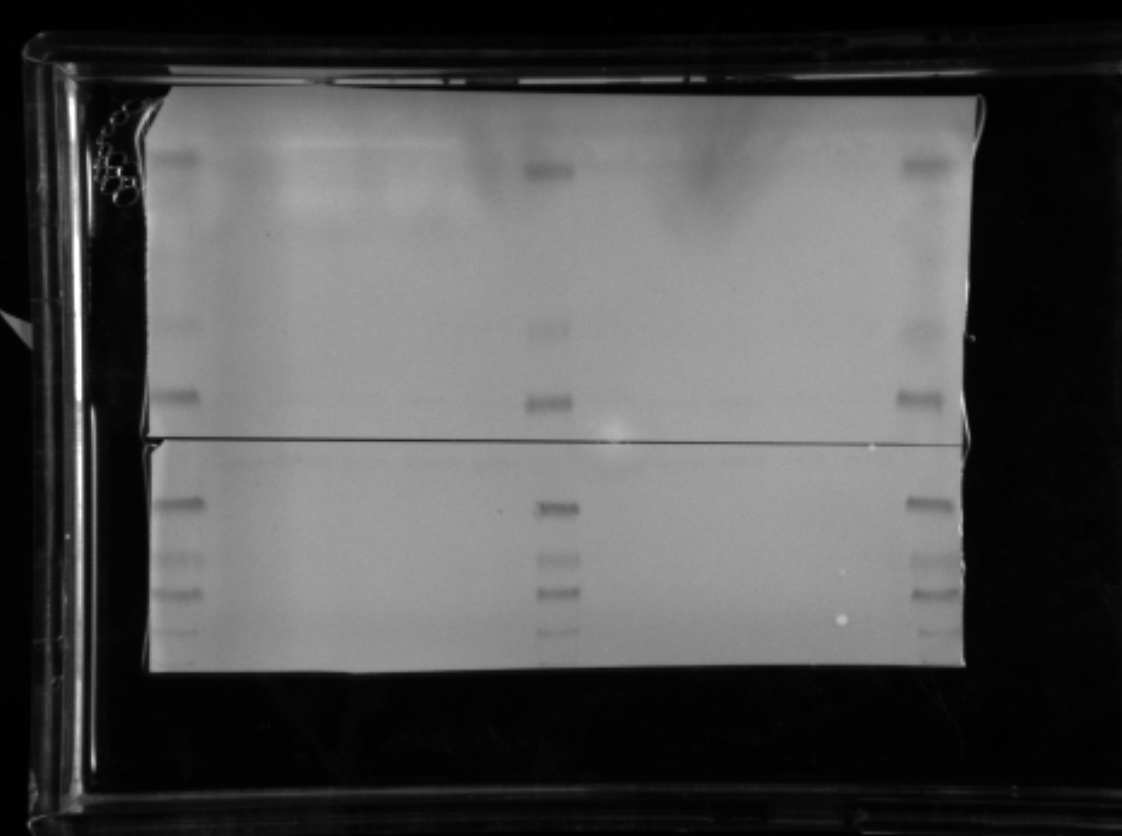

Supplement: S2 File — (ZIP) [file pone.0335225.s007.zip › Cell WB/p-SMAD/p-SMAD/图maker3.tif]

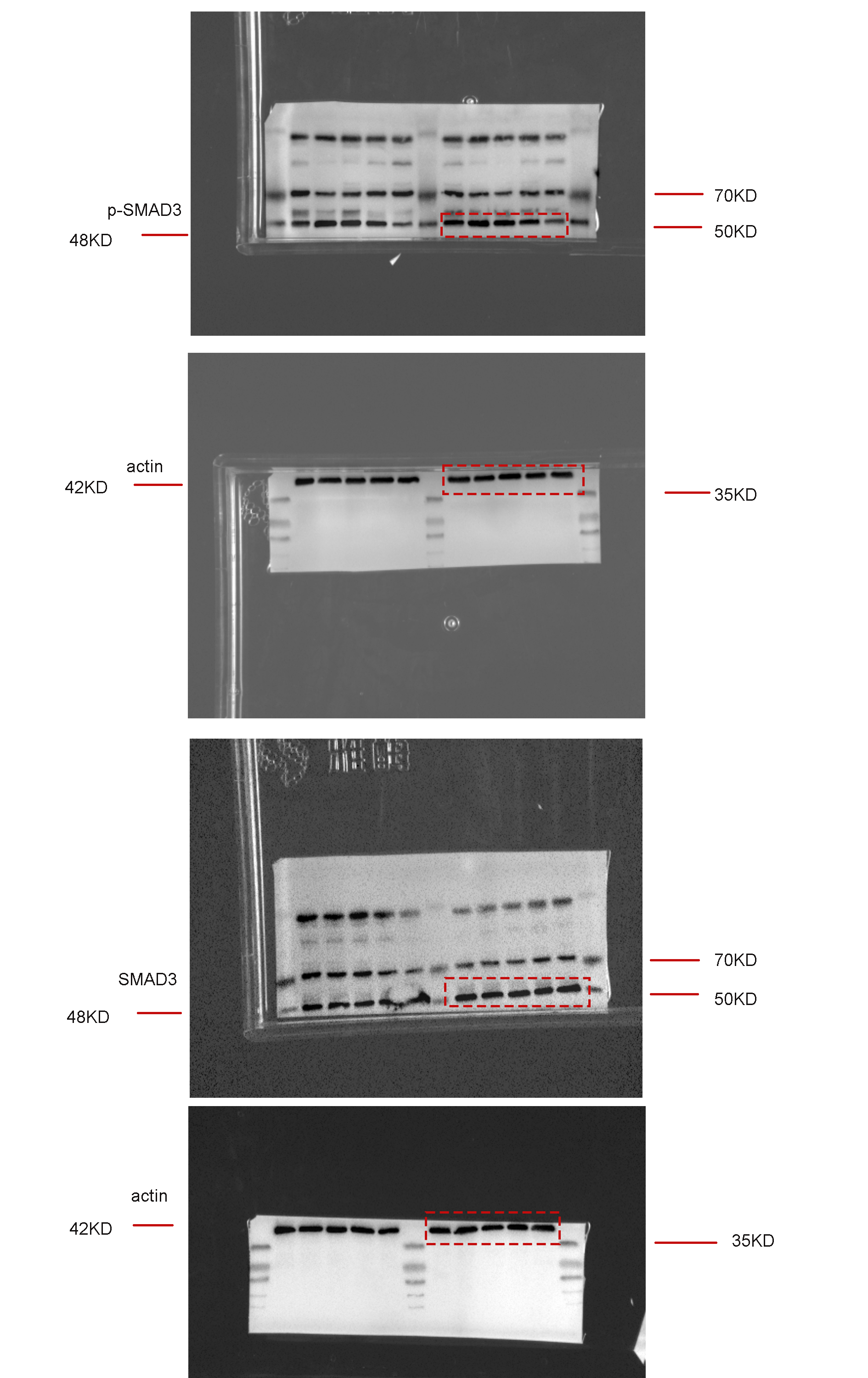

Supplement: S2 File — (ZIP) [file pone.0335225.s007.zip › Cell WB/p-SMAD/细胞 SMAD.tif]
