# Supplementary material for: Plasma-activated media inhibits epithelial-mesenchymal transition and ameliorates intestinal fibrosis through the PPARγ/TGF-β1/SMAD3 pathway
Source: PLoS One. 2025 Oct 22;20(10):e0335225. doi: 10.1371/journal.pone.0335225 (PMC12543144; doi:10.1371/journal.pone.0335225)
Supplement: S1 Table — (DOCX) [file pone.0335225.s003.docx]

Supplementary Table 1

| Scores | Percentage of weight loss | Fecal consistency | Fecal occult blood |
| --- | --- | --- | --- |
| 0 | 0 | Normal | Negative |
| 1 | 1%-5% | Soft stool | Cambridge blue |
| 2 | 5%-10% | Mucoid stool | Blue |
| 3 | 10%-20% | Watery stool | Dark blue |
| 4 | ＞20% |  | Visible blood in stool |

Note. Percentage of weight loss = (original weight - weight after loss) / original weight x 100%. Fecal consistency: firm and formed is normal, soft and loose is soft stool, unformed and adhered is mucoid stool, watery is watery stool. Fecal occult blood: On the mouse feces, successively add 2% o-tolidine and 3% hydrogen peroxide. Observe the color change within 2 minutes.
