# Supplementary material for: Plasma-activated media inhibits epithelial-mesenchymal transition and ameliorates intestinal fibrosis through the PPARγ/TGF-β1/SMAD3 pathway
Source: PLoS One. 2025 Oct 22;20(10):e0335225. doi: 10.1371/journal.pone.0335225 (PMC12543144; doi:10.1371/journal.pone.0335225)
Supplement: S2 Table — (DOCX) [file pone.0335225.s004.docx]

Supplementary Table 2

| Primer name | Upstream sequence | Downstream sequence |
| --- | --- | --- |
| E-cadherin (mouse) | AACCCAAGCACGTATCAGGG | GAGTGTTGGGGGCATCATCA |
| N-cadherin (mouse) | GGCCTTGCTTCAGGCGT | CATTGAGAAGGGGCTGTCCT |
| Vimentin (mouse) | CGGCTGCGAGAGAAATTGC | CCACTTTCCGTTCAAGGTCAAG |
| a-SMA (mouse) | GCCATCTTTCATTGGGATGGA | CCCCTGACAGGACGTTGTTA |
| COL1A1 (mouse) | CGATGGATTCCCGTTCGAGT | GAGGCCTCGGTGGACATTAG |
| GAPDH (mouse) | TGGCCTTCCGTGTTCCTAC | GAGTTGCTGTTGAAGTCGCA |
