# Supplementary material for: Plasma-activated media inhibits epithelial-mesenchymal transition and ameliorates intestinal fibrosis through the PPARγ/TGF-β1/SMAD3 pathway
Source: PLoS One. 2025 Oct 22;20(10):e0335225. doi: 10.1371/journal.pone.0335225 (PMC12543144; doi:10.1371/journal.pone.0335225)
Supplement: S3 Table — (DOCX) [file pone.0335225.s005.docx]

Supplementary Table 3

| Primer name | Upstream sequence | Downstream sequence |
| --- | --- | --- |
| E-cadherin (Rat) | ATGTCCTGGGCAGAGTGAGA | TGGAGCTTTAGATGCCGCTT |
| N-cadherin (Rat) | CCTCCAACGGGCATCTTCAT | ATGTGCCCTCAGGTGAAACC |
| Vimentin (Rat) | TGCGGCTGCGAGAAAAATTG | GGTCAAGACGTGCCAGAGAA |
| a-SMA (Rat) | AGACCTTCAATGTCCCTGCC | GTCCAGCACAATACCAGTTGT |
| COL1A1 (Rat) | CTTTGTGGACCTCCGGCTC | AGCTGACTTCAGGGATGTCTTC |
| GAPDH (Rat) | GAAGGTGAAGGTCGGAGTC | GAAGATTGGTGATGGGATTTC |
