# Supplementary figures and images for: Plasma-activated media inhibits epithelial-mesenchymal transition and ameliorates intestinal fibrosis through the PPARγ/TGF-β1/SMAD3 pathway (part 2 of 2)
Source: PLoS One. 2025 Oct 22;20(10):e0335225. doi: 10.1371/journal.pone.0335225 (PMC12543144; doi:10.1371/journal.pone.0335225)

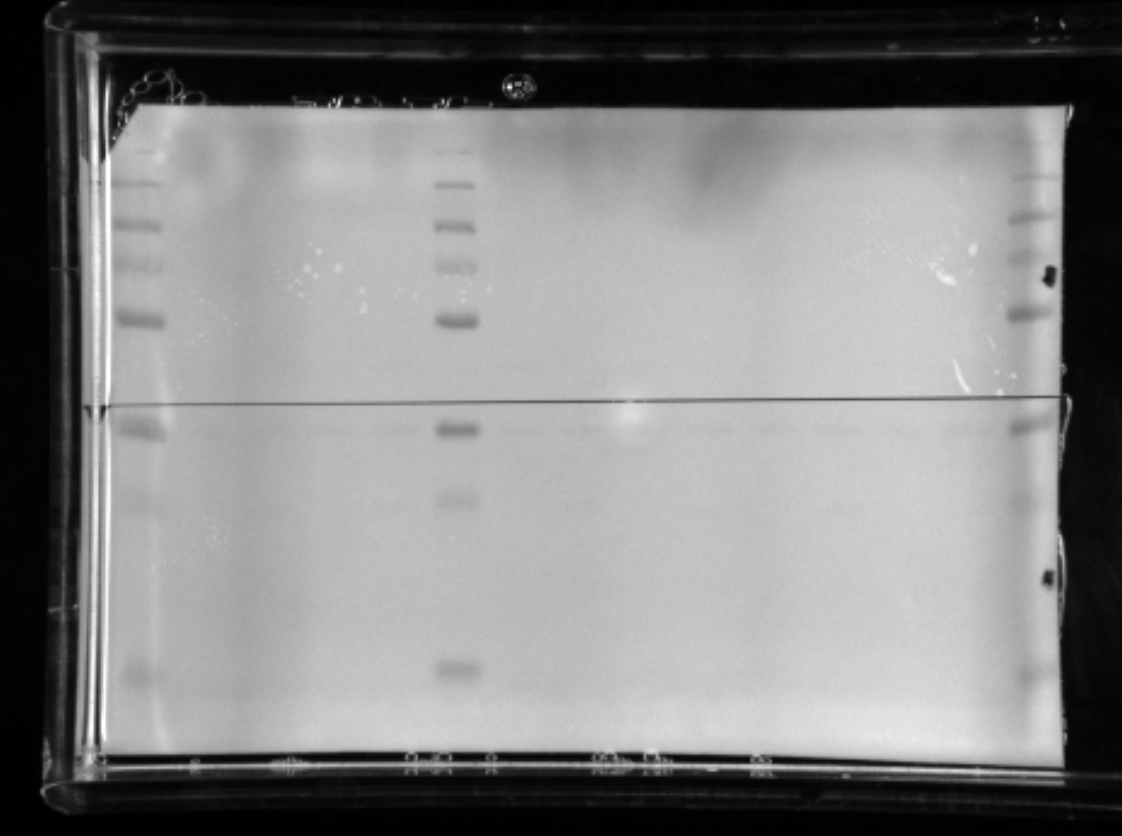

Supplement: S5 File — (ZIP) [file pone.0335225.s010.zip › Response experiment WB/A/图maker1.tif]

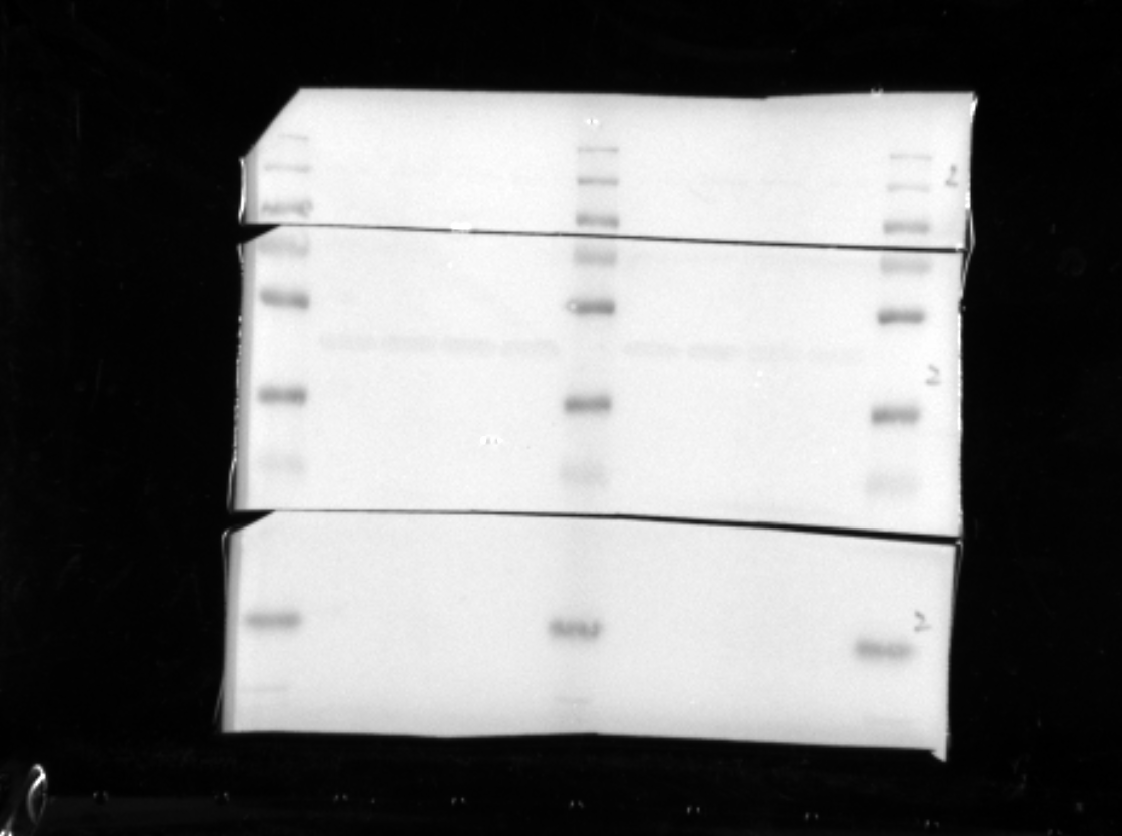

Supplement: S5 File — (ZIP) [file pone.0335225.s010.zip › Response experiment WB/COL/MAKER2.tif]

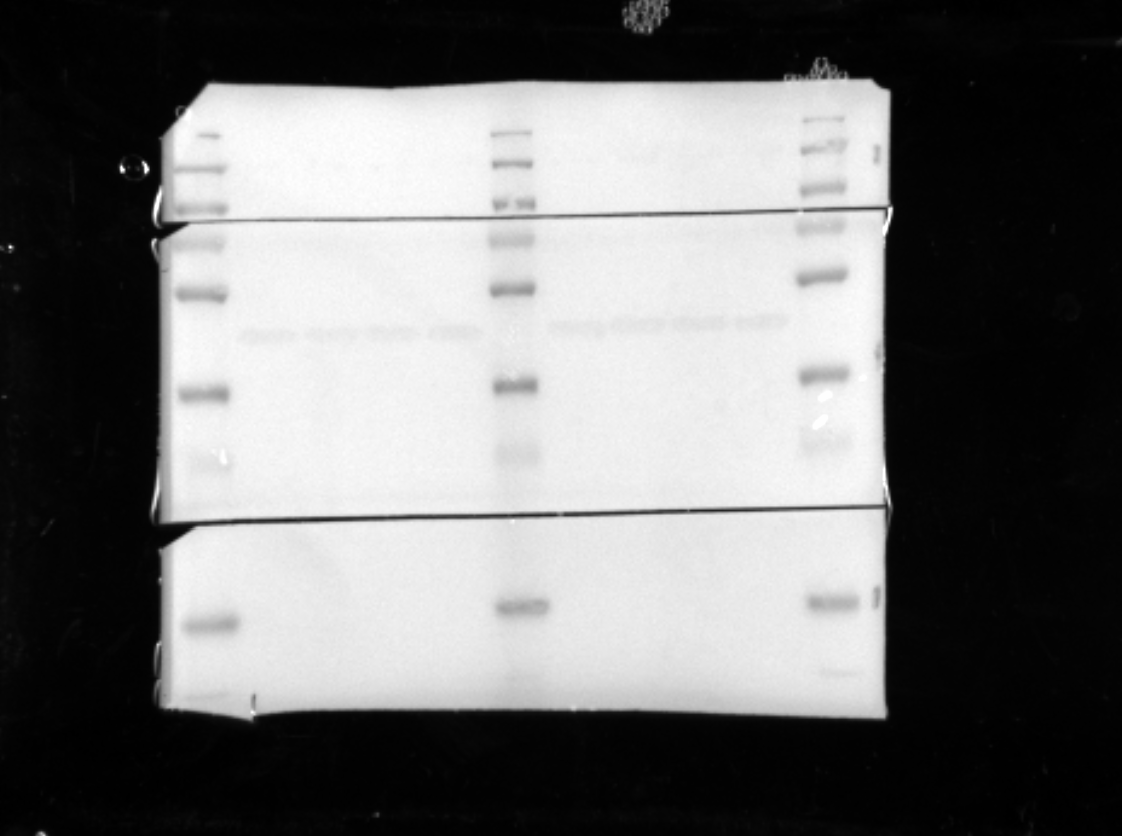

Supplement: S5 File — (ZIP) [file pone.0335225.s010.zip › Response experiment WB/COL/图MAKER1.tif]

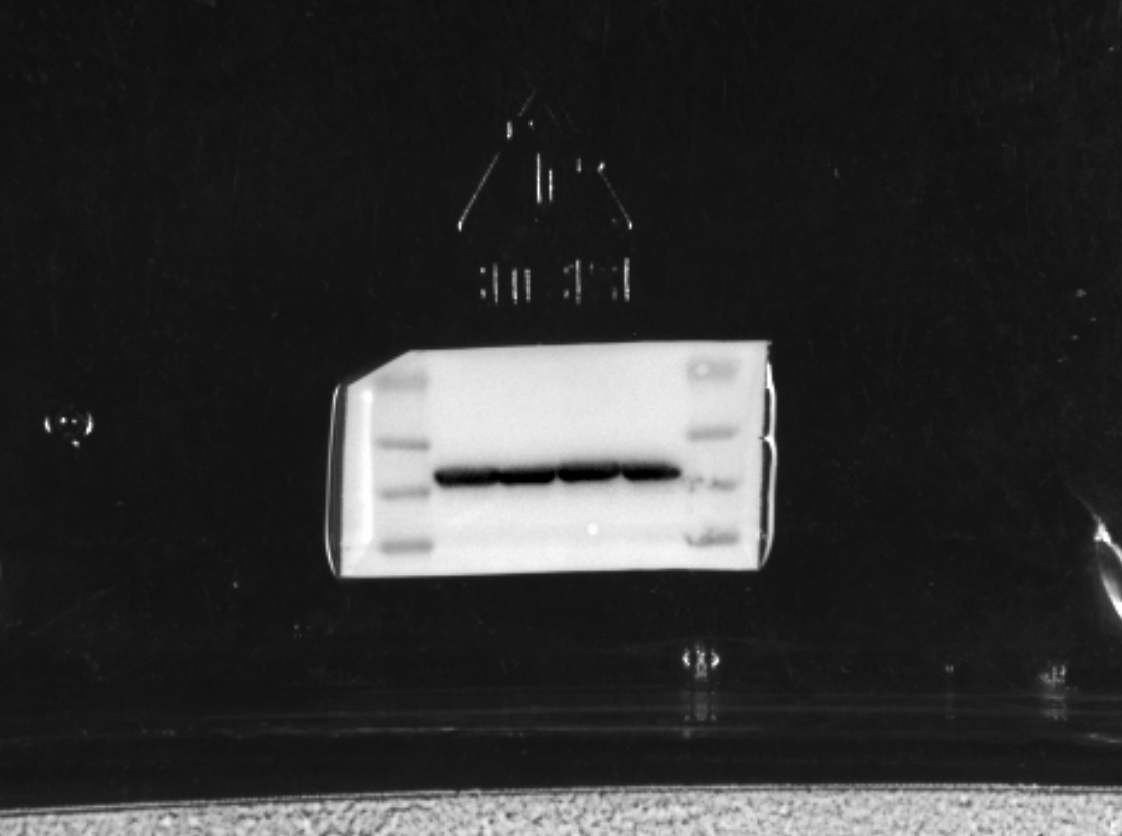

Supplement: S5 File — (ZIP) [file pone.0335225.s010.zip › Response experiment WB/E/E1/ACTIN 0_3S HB.tif]

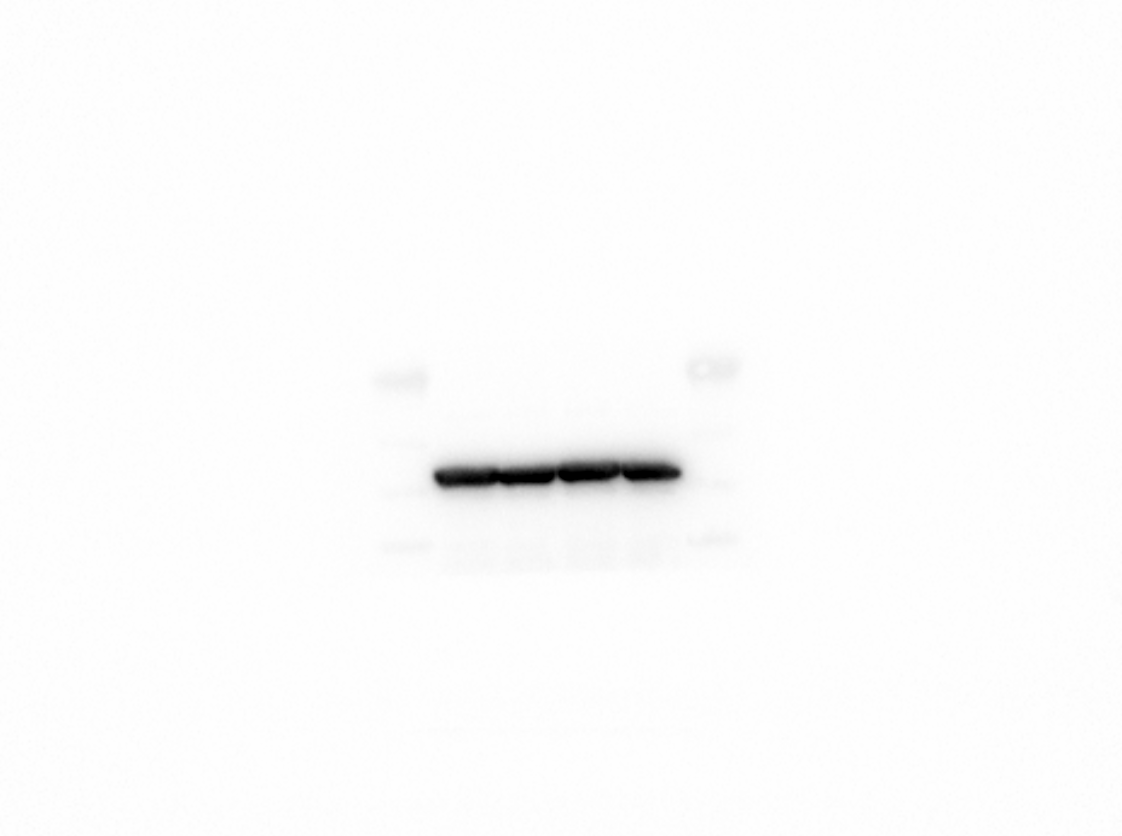

Supplement: S5 File — (ZIP) [file pone.0335225.s010.zip › Response experiment WB/E/E1/ACTIN 0_3S.tif]

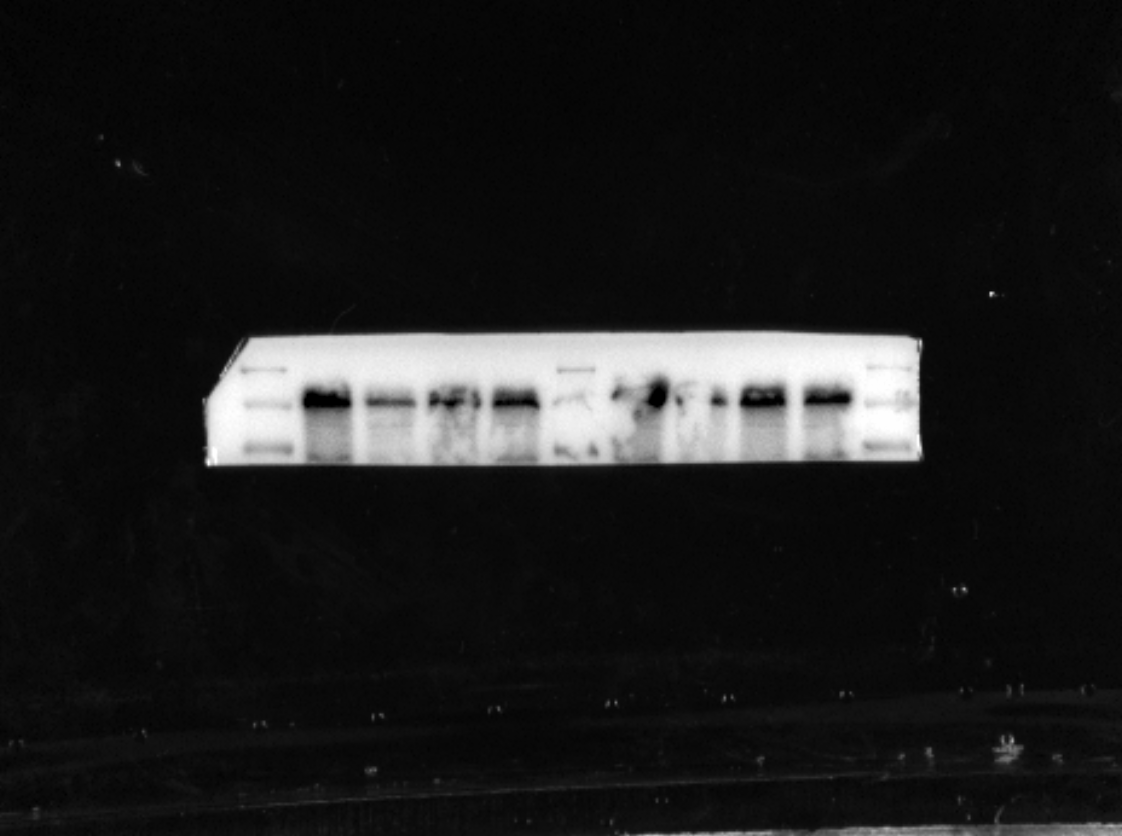

Supplement: S5 File — (ZIP) [file pone.0335225.s010.zip › Response experiment WB/E/E1/E 1S 4 HB.tif]

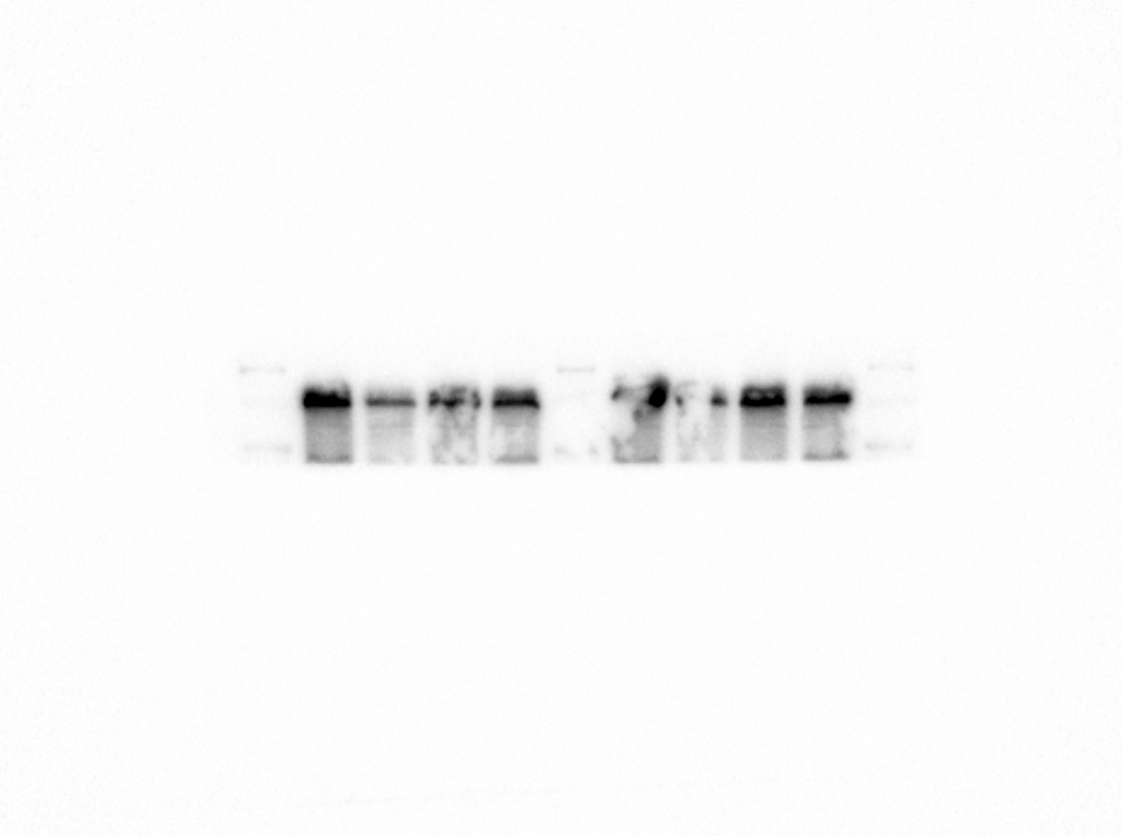

Supplement: S5 File — (ZIP) [file pone.0335225.s010.zip › Response experiment WB/E/E1/E 1S 4.tif]

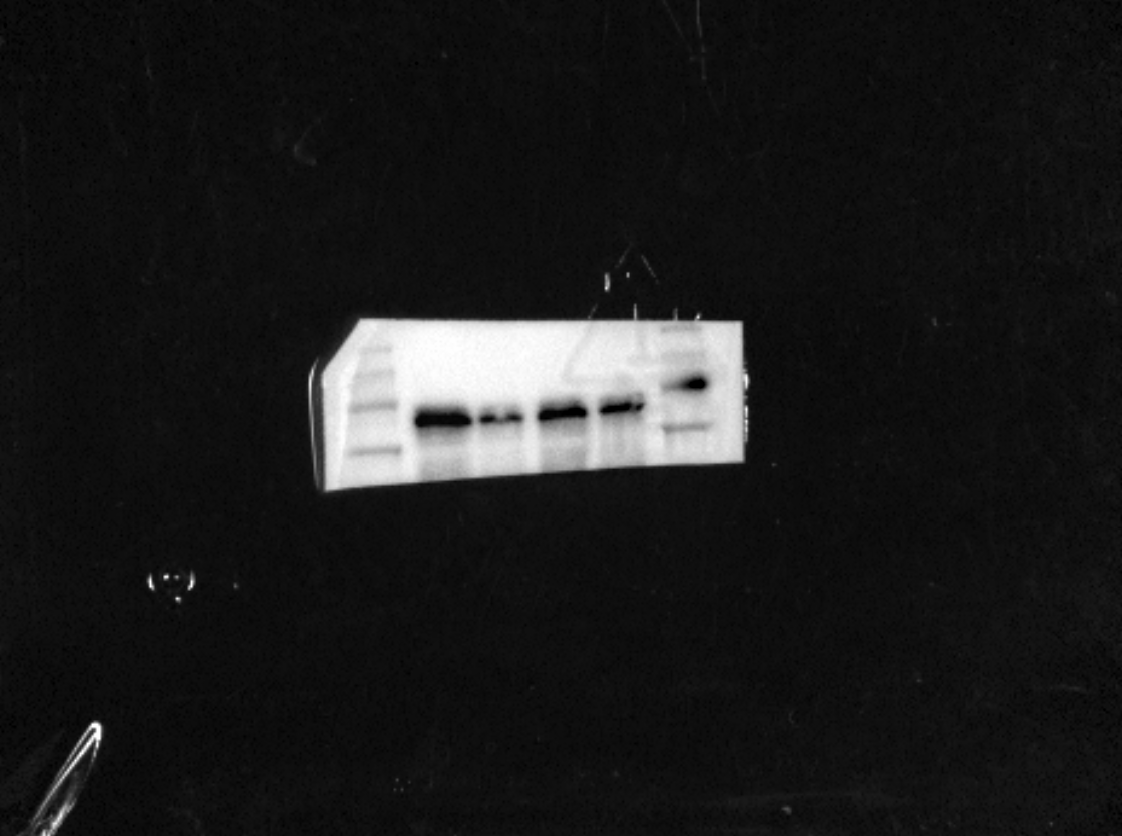

Supplement: S5 File — (ZIP) [file pone.0335225.s010.zip › Response experiment WB/E/E1/E 5S HB.tif]

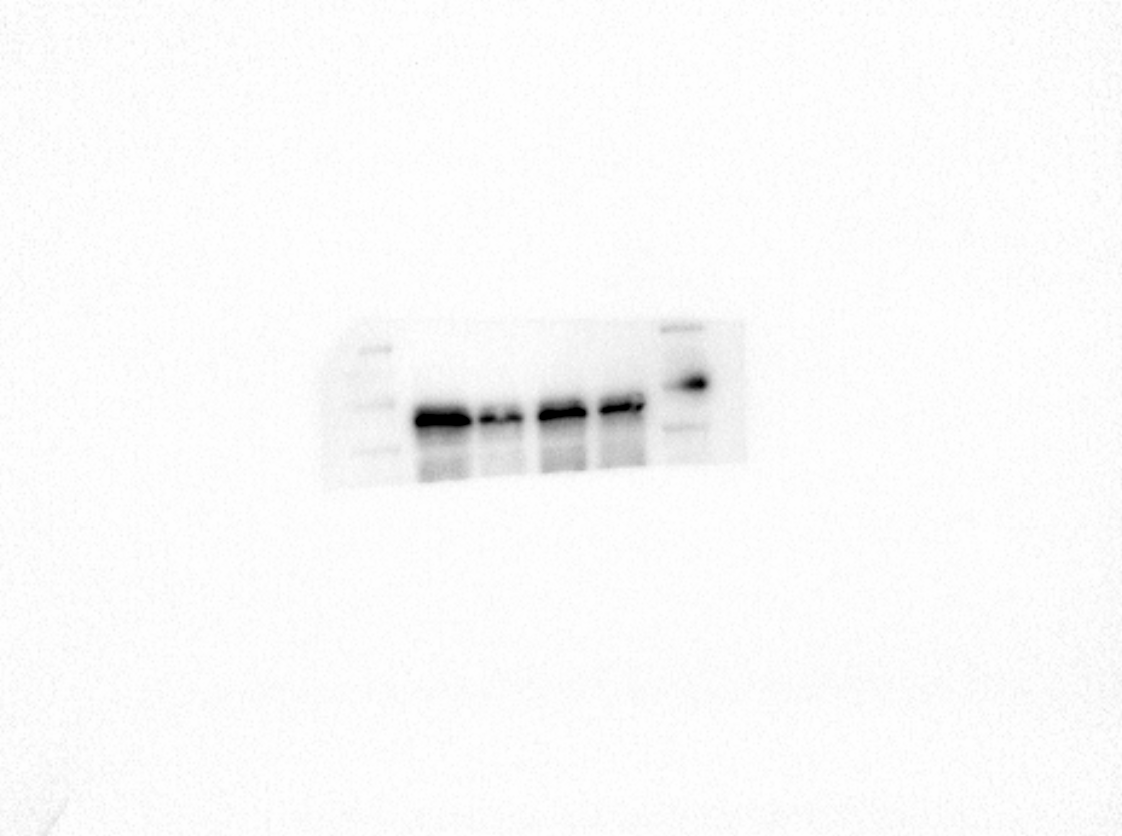

Supplement: S5 File — (ZIP) [file pone.0335225.s010.zip › Response experiment WB/E/E1/E 5S.tif]

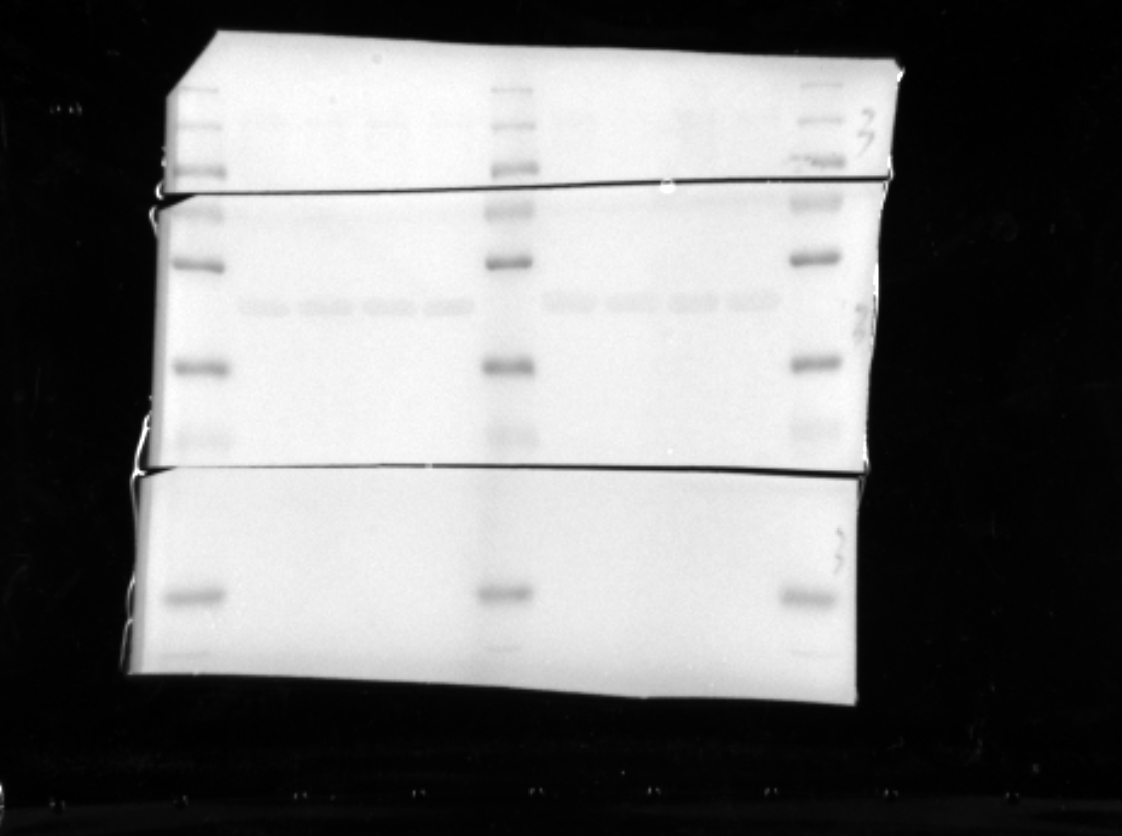

Supplement: S5 File — (ZIP) [file pone.0335225.s010.zip › Response experiment WB/E/E1/MAKER 3.tif]

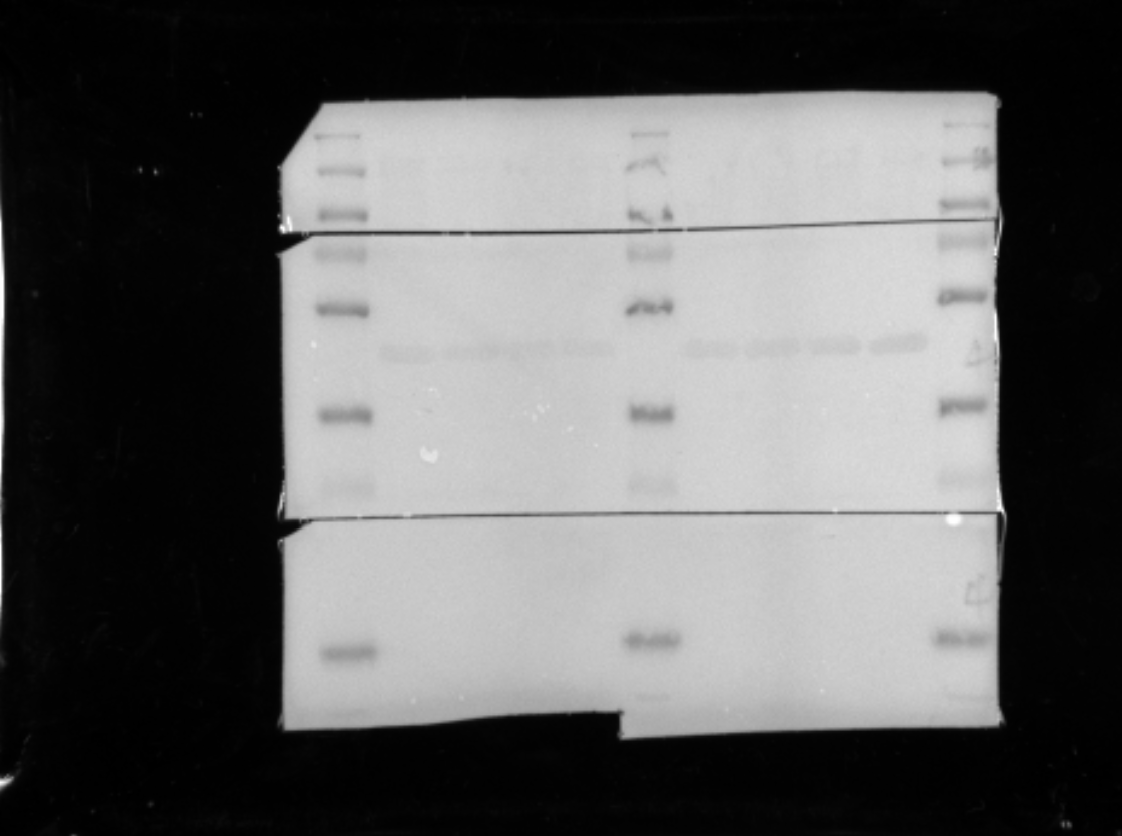

Supplement: S5 File — (ZIP) [file pone.0335225.s010.zip › Response experiment WB/E/E1/MAKER4.tif]

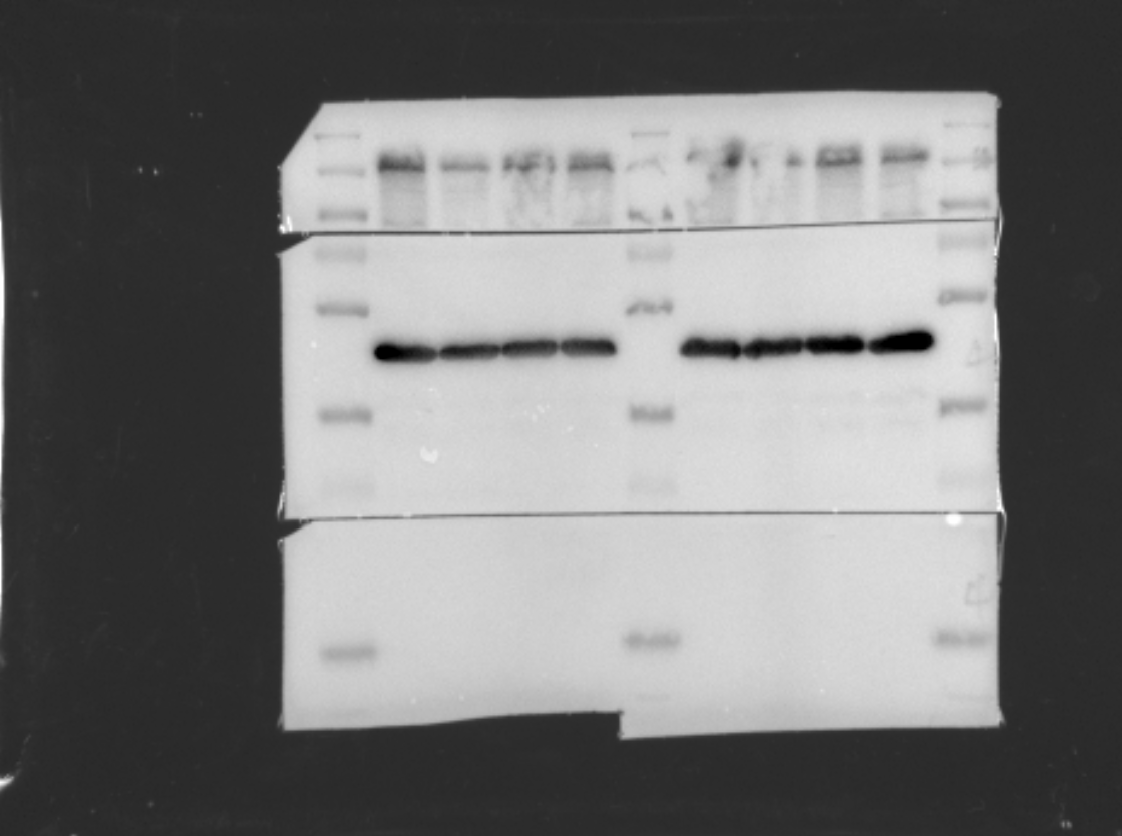

Supplement: S5 File — (ZIP) [file pone.0335225.s010.zip › Response experiment WB/E/E1/ZHEGMO 4.tif]

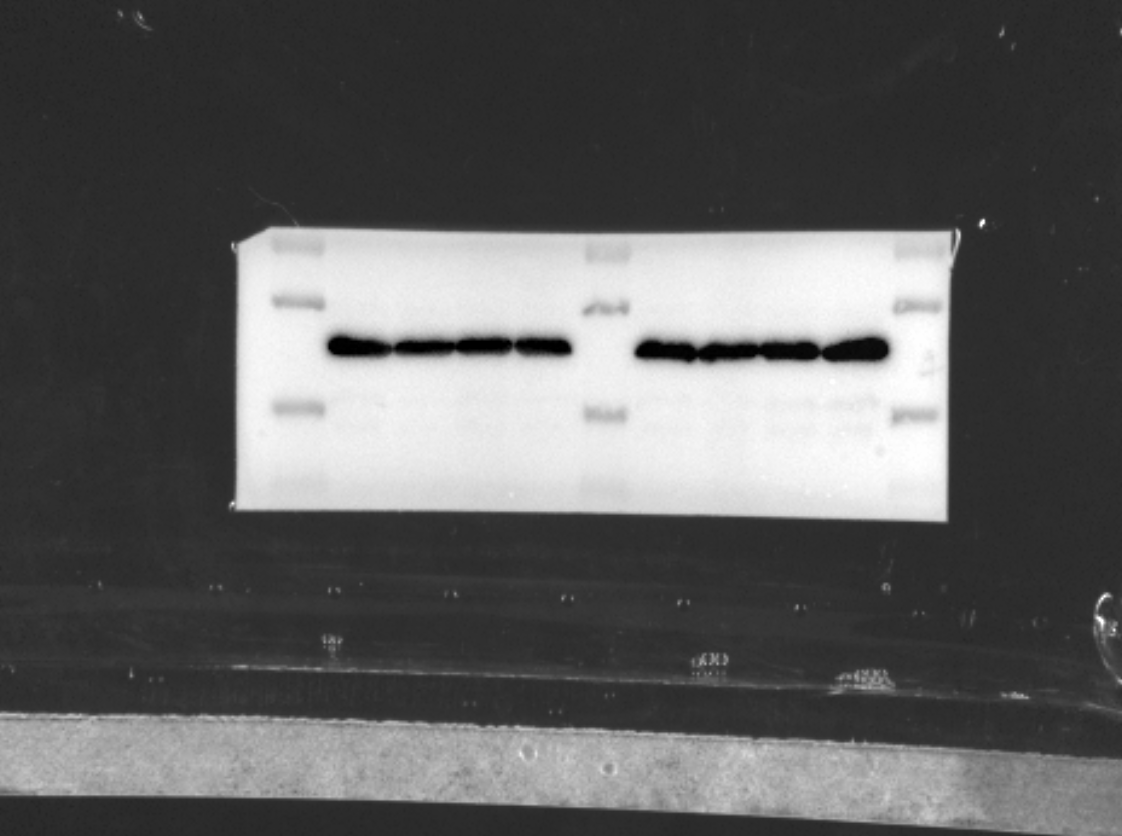

Supplement: S5 File — (ZIP) [file pone.0335225.s010.zip › Response experiment WB/E/E1/actin 4 0_5s hb.tif]

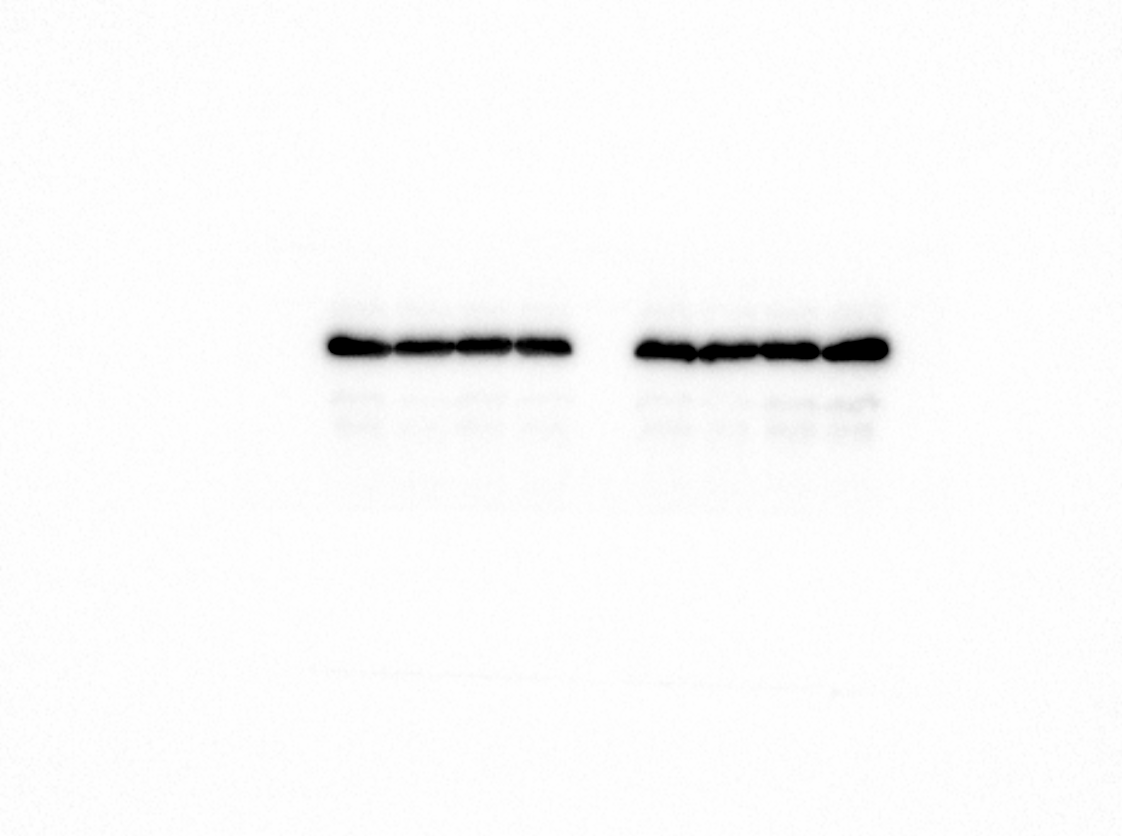

Supplement: S5 File — (ZIP) [file pone.0335225.s010.zip › Response experiment WB/E/E1/actin 4 0_5s.tif]

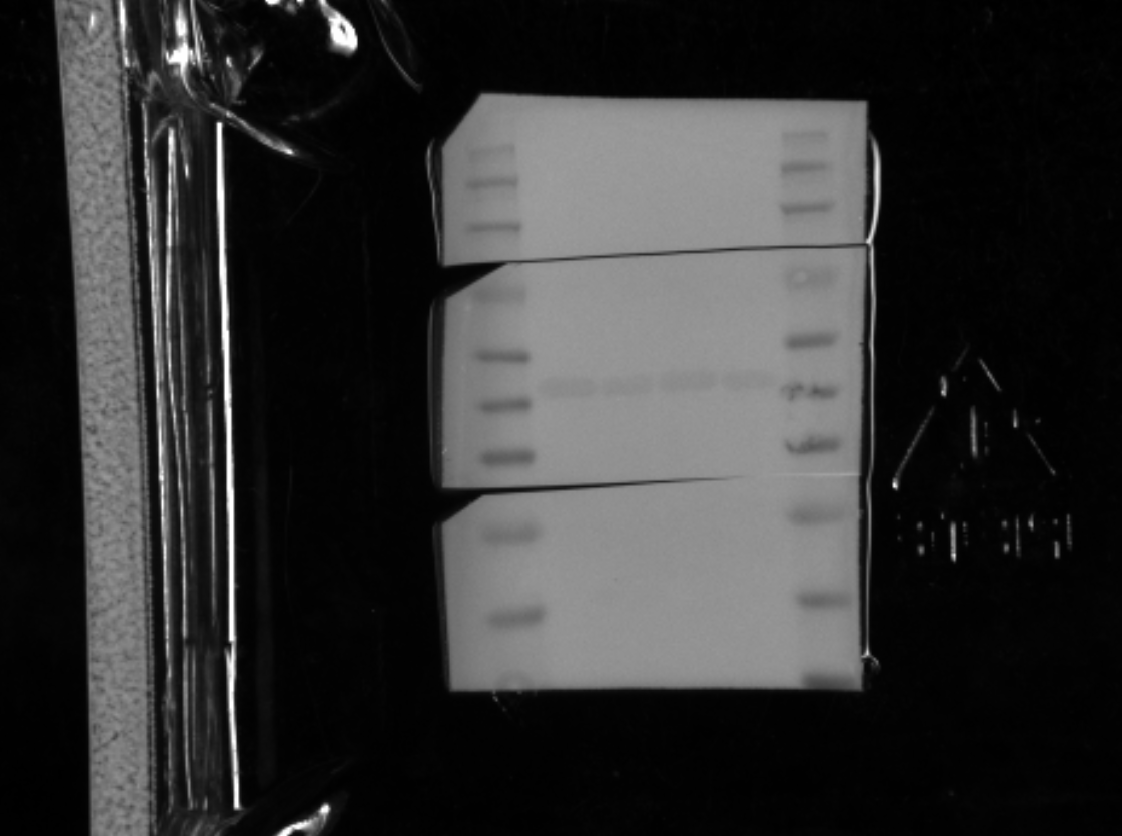

Supplement: S5 File — (ZIP) [file pone.0335225.s010.zip › Response experiment WB/E/E1/maker1.tif]

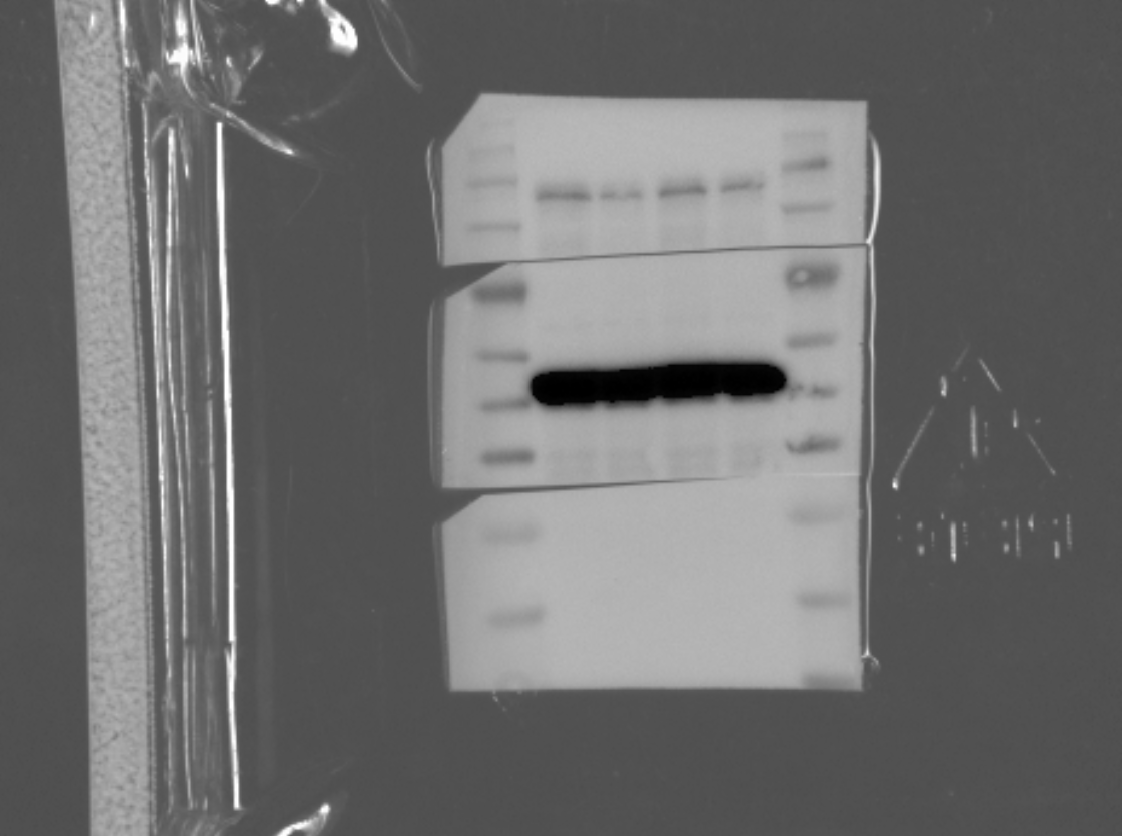

Supplement: S5 File — (ZIP) [file pone.0335225.s010.zip › Response experiment WB/E/E1/zhengmo.tif]

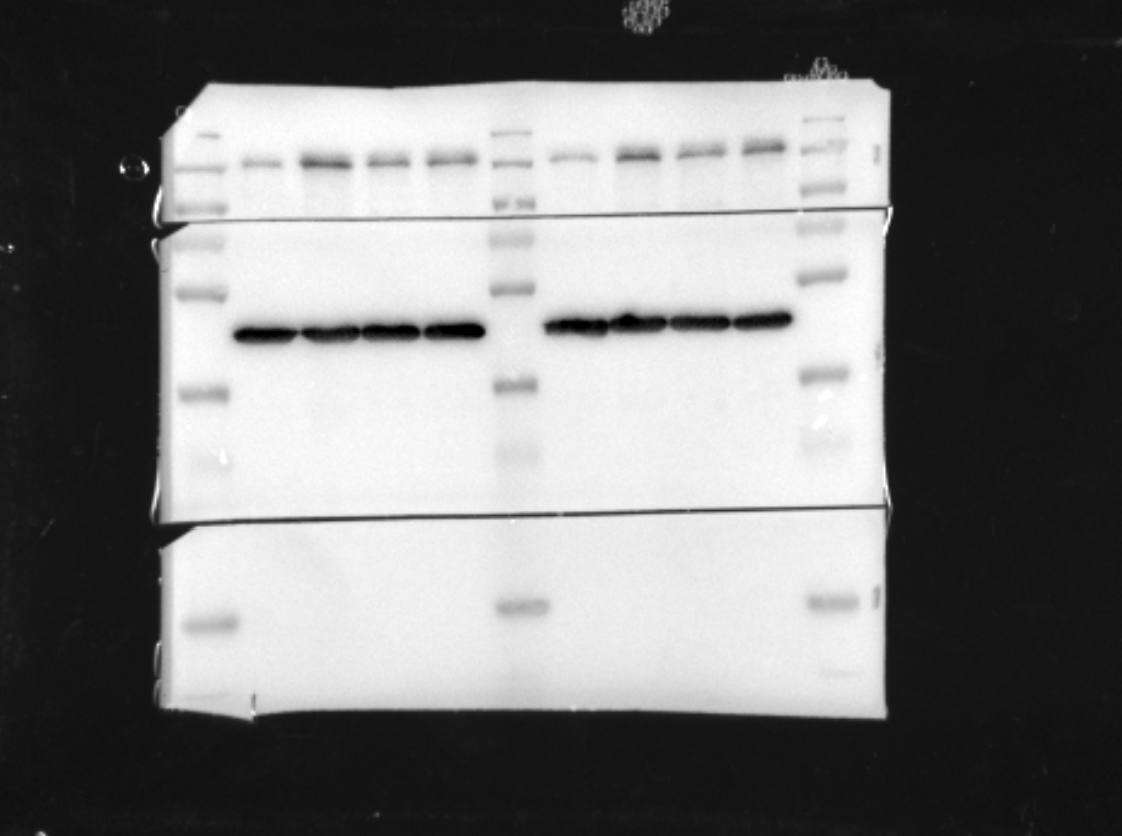

Supplement: S5 File — (ZIP) [file pone.0335225.s010.zip › Response experiment WB/E/E2/ZHENGMO 1.tif]

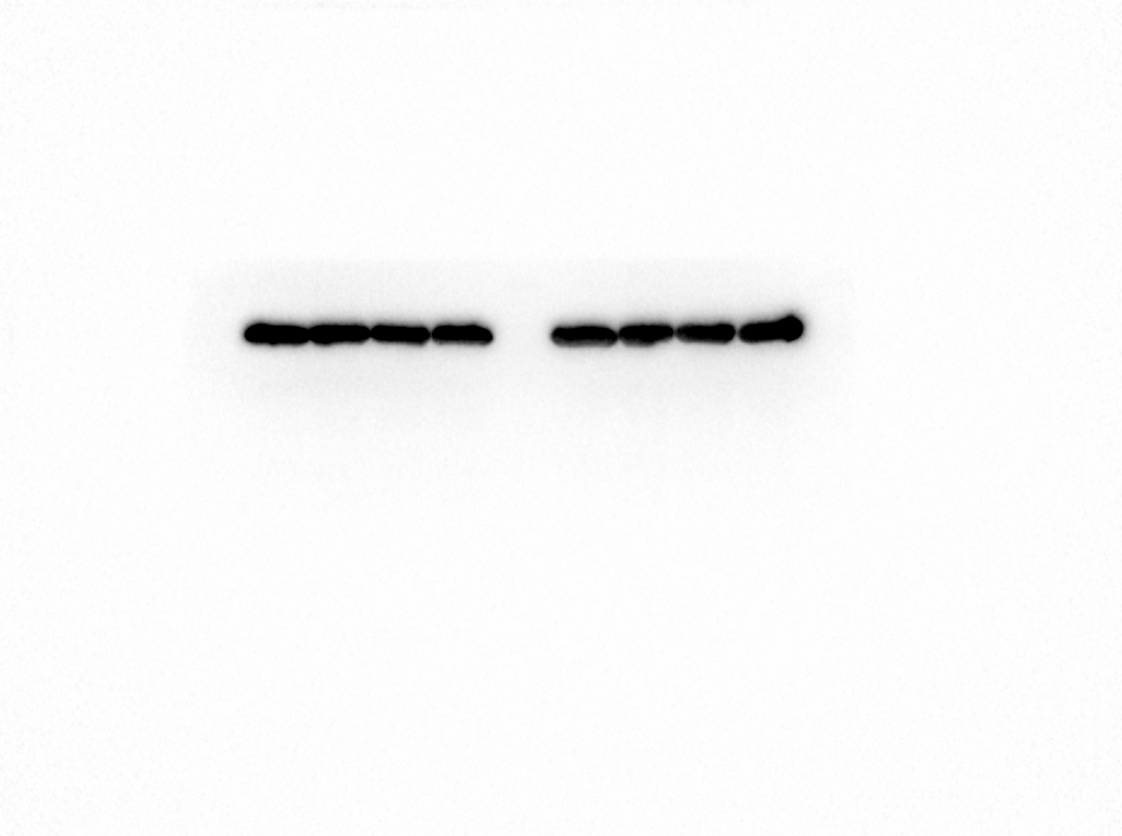

Supplement: S5 File — (ZIP) [file pone.0335225.s010.zip › Response experiment WB/N/N/ACTIN 0_3S 2_1.tif]

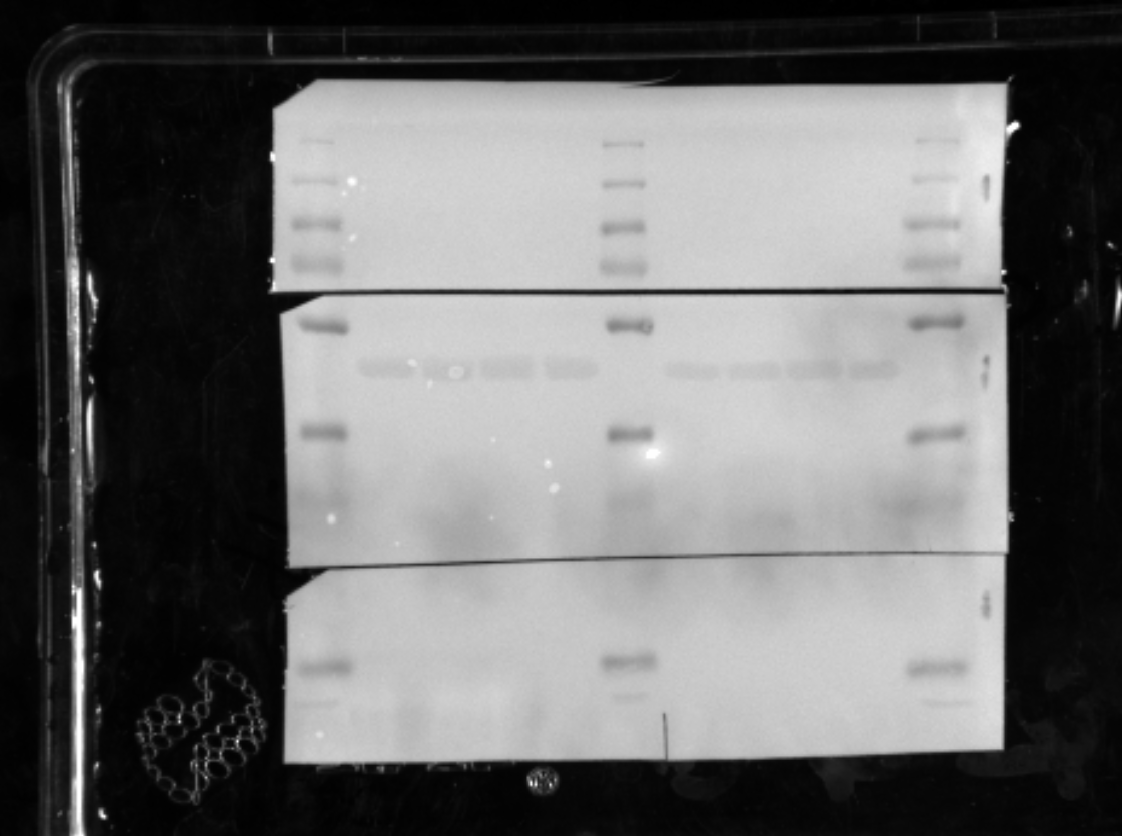

Supplement: S5 File — (ZIP) [file pone.0335225.s010.zip › Response experiment WB/N/N/MAKER1.tif]

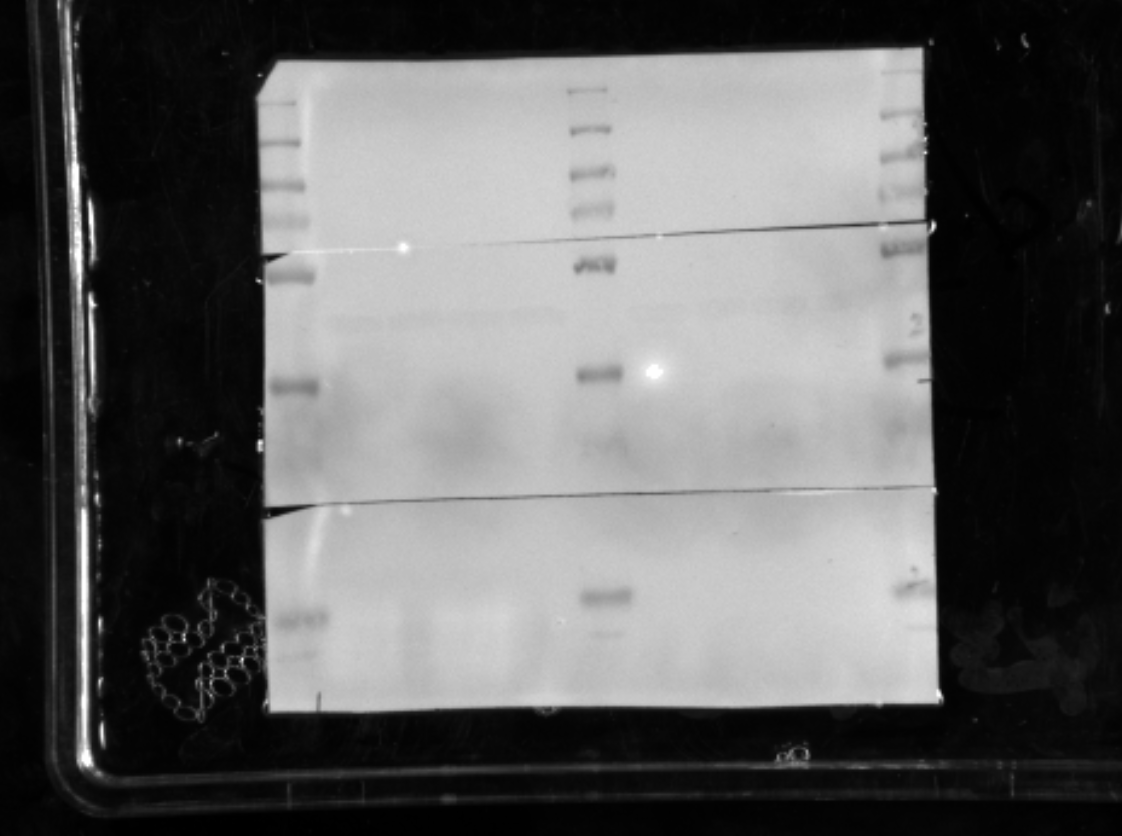

Supplement: S5 File — (ZIP) [file pone.0335225.s010.zip › Response experiment WB/N/N/图MAKER2.tif]

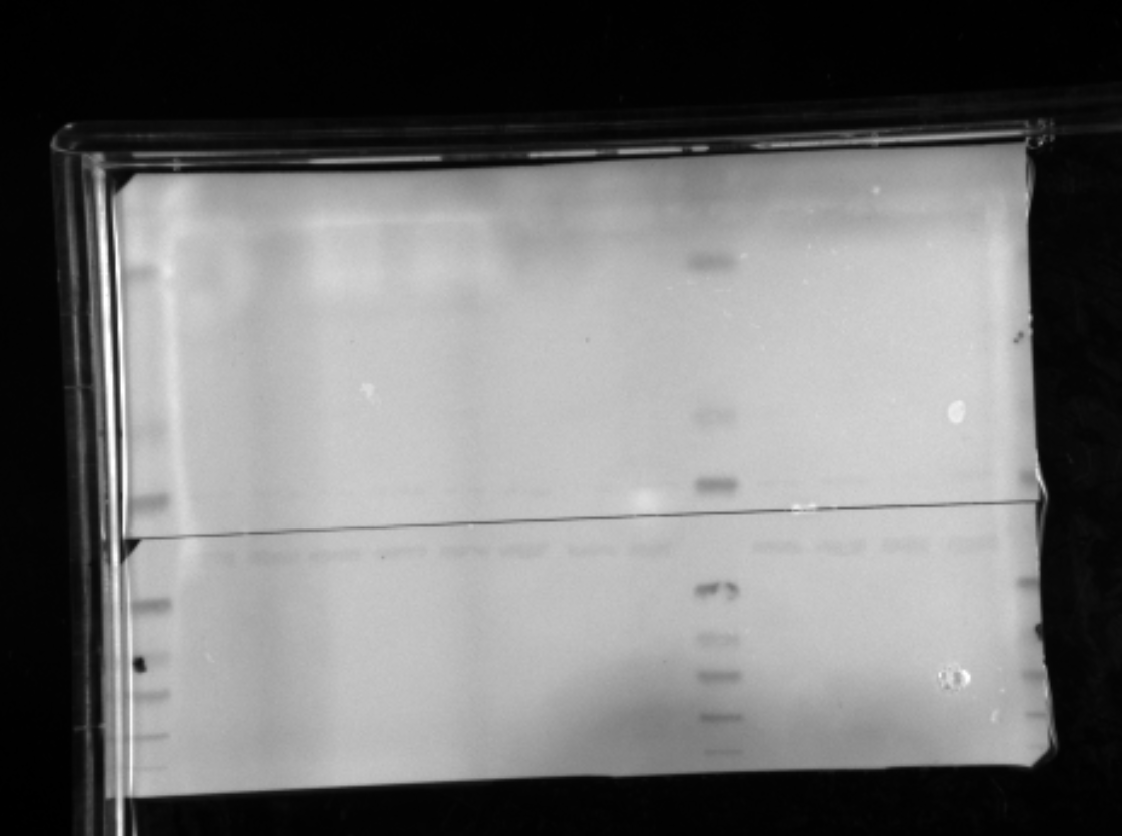

Supplement: S5 File — (ZIP) [file pone.0335225.s010.zip › Response experiment WB/P-SMAD/p-smad/maker1.tif]

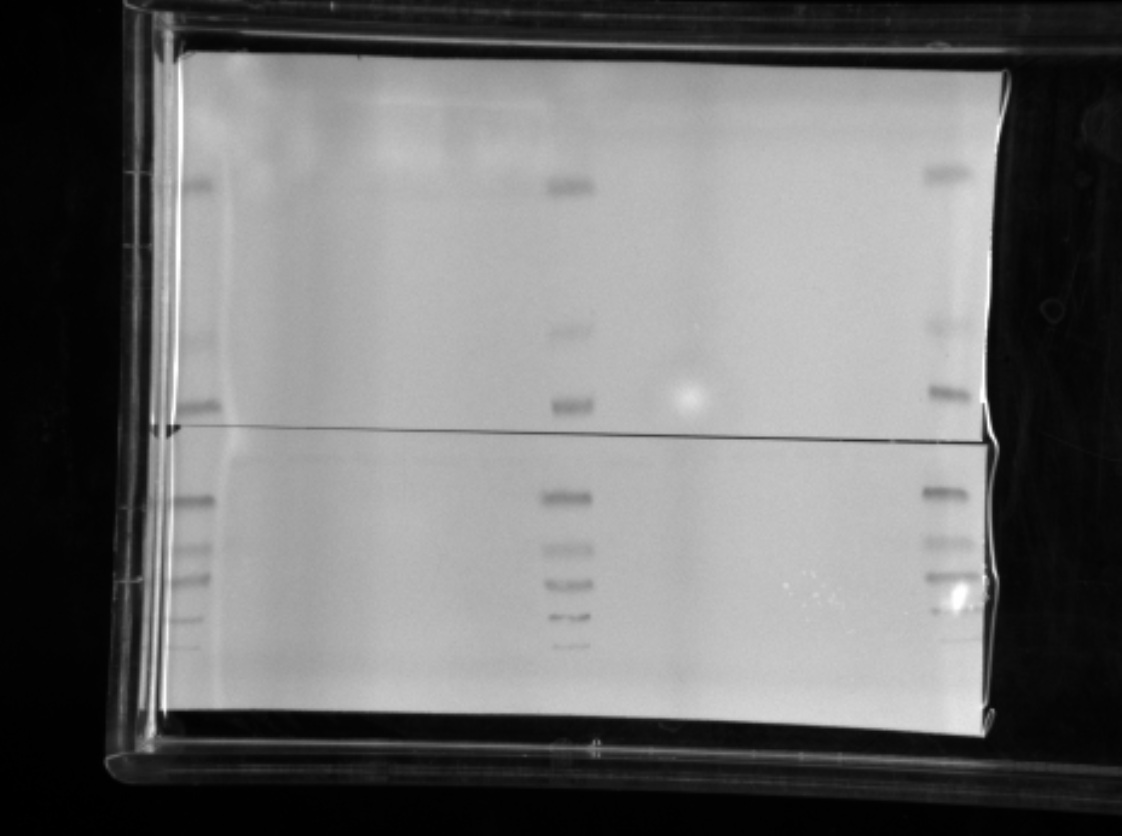

Supplement: S5 File — (ZIP) [file pone.0335225.s010.zip › Response experiment WB/P-SMAD/samd/maker1.tif]

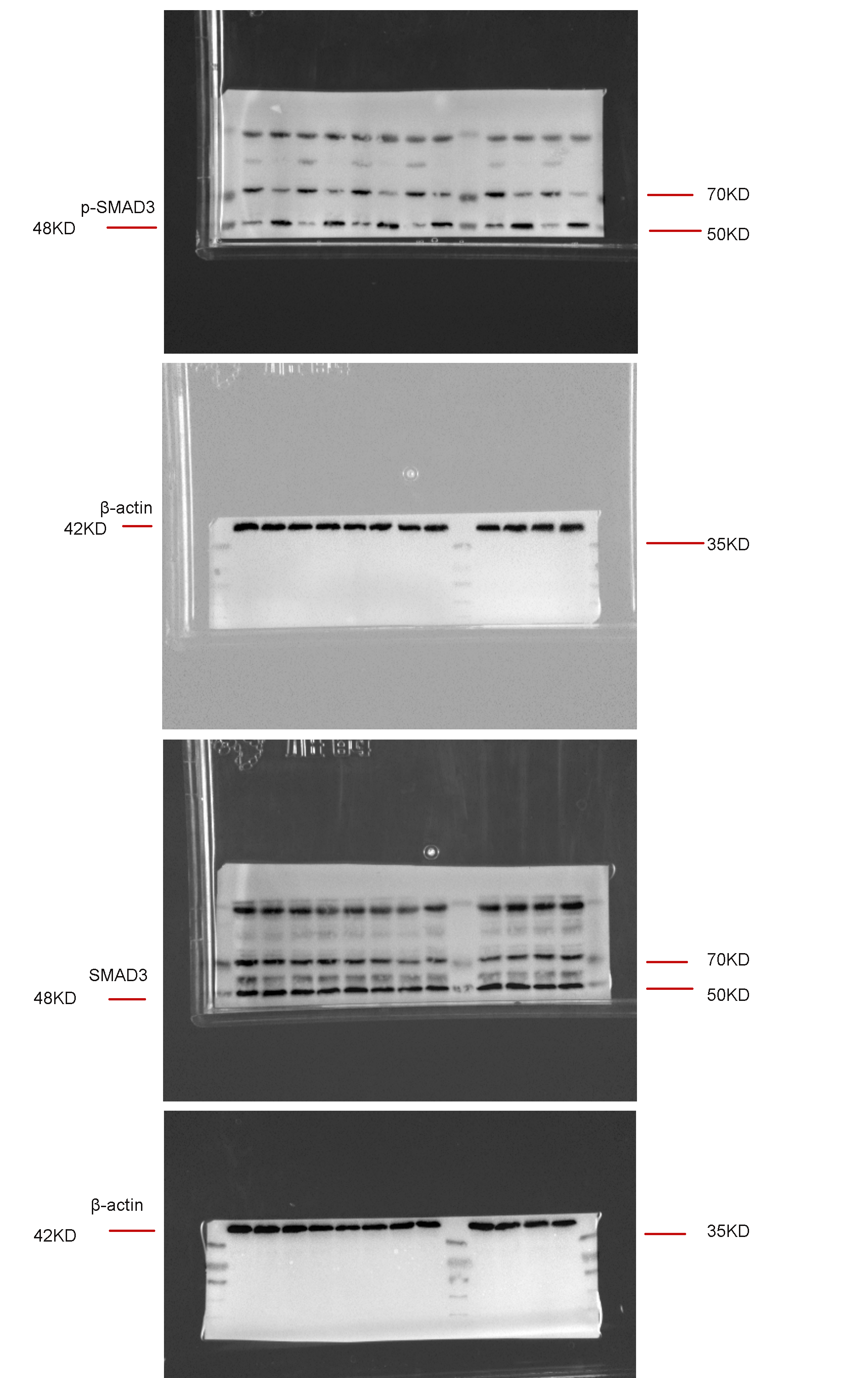

Supplement: S5 File — (ZIP) [file pone.0335225.s010.zip › Response experiment WB/P-SMAD/回复p-SMAD3.tif]

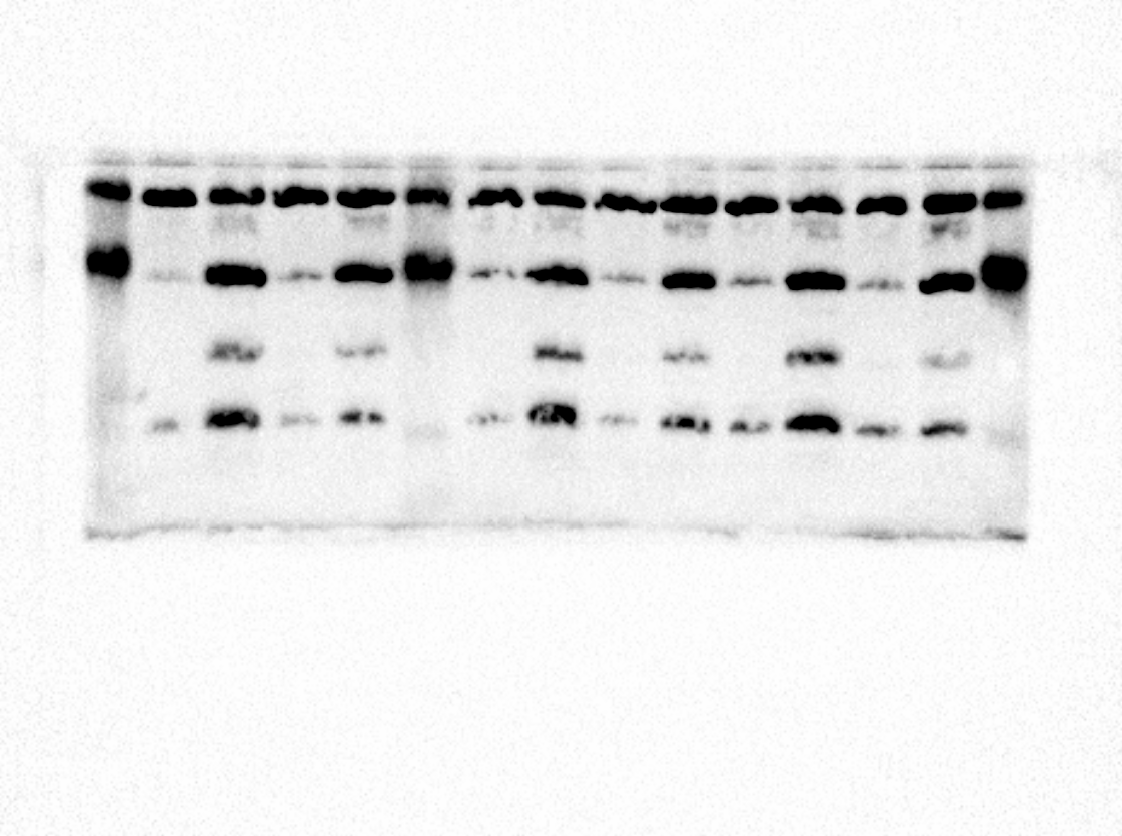

Supplement: S5 File — (ZIP) [file pone.0335225.s010.zip › Response experiment WB/TGF/图GAPDH 0_5S 2.tif]

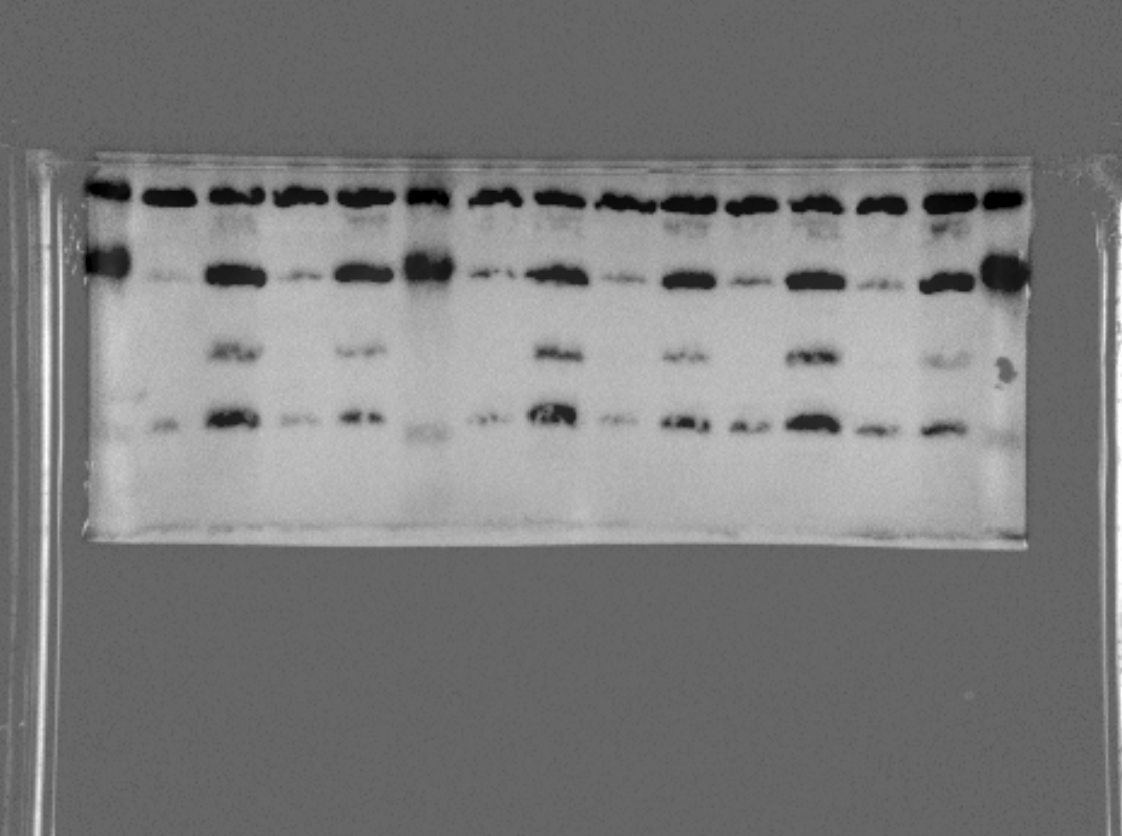

Supplement: S5 File — (ZIP) [file pone.0335225.s010.zip › Response experiment WB/TGF/图GAPDH 0_5S HB.tif]

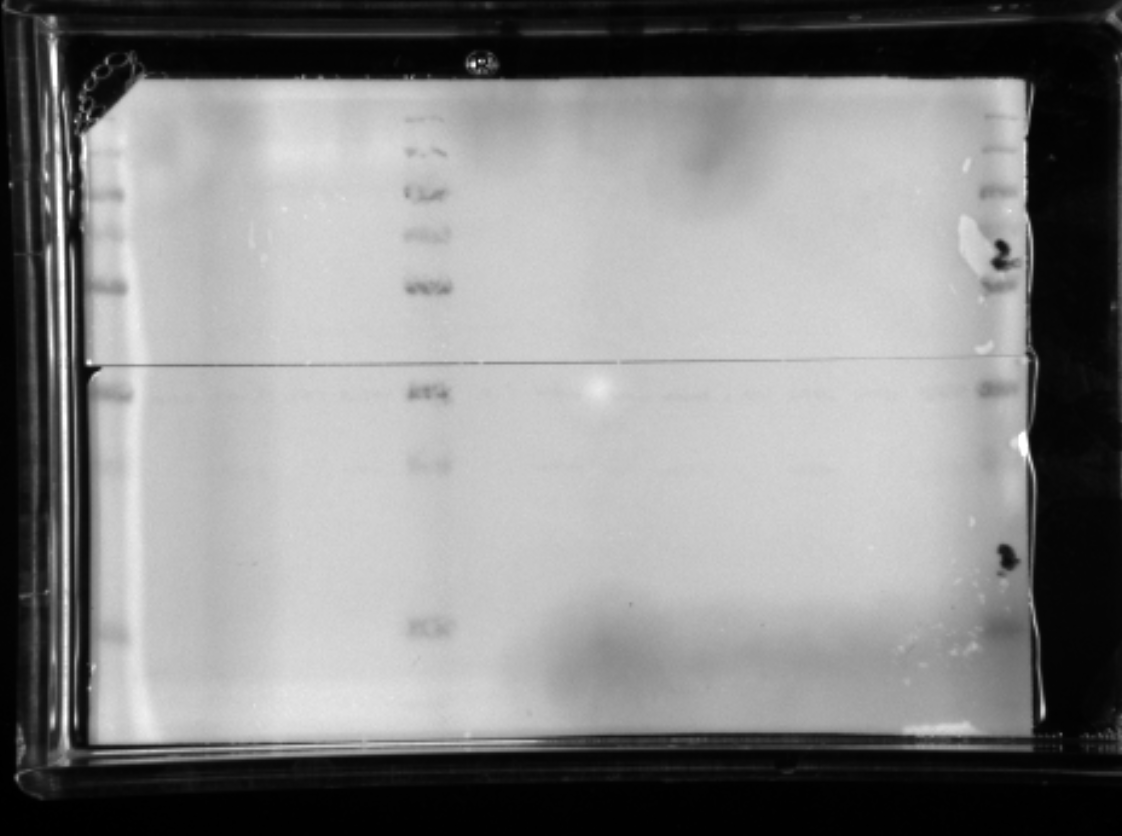

Supplement: S5 File — (ZIP) [file pone.0335225.s010.zip › Response experiment WB/TGF/图maker2.tif]

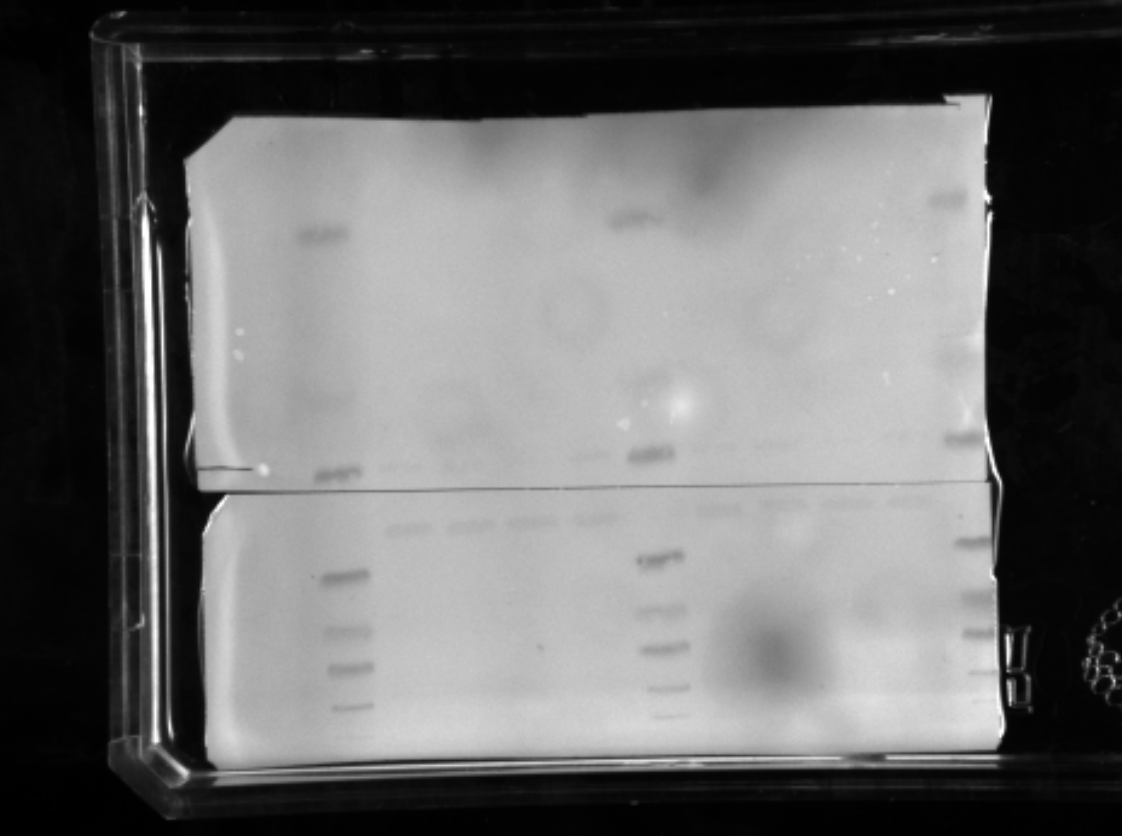

Supplement: S5 File — (ZIP) [file pone.0335225.s010.zip › Response experiment WB/V/maker2.tif]

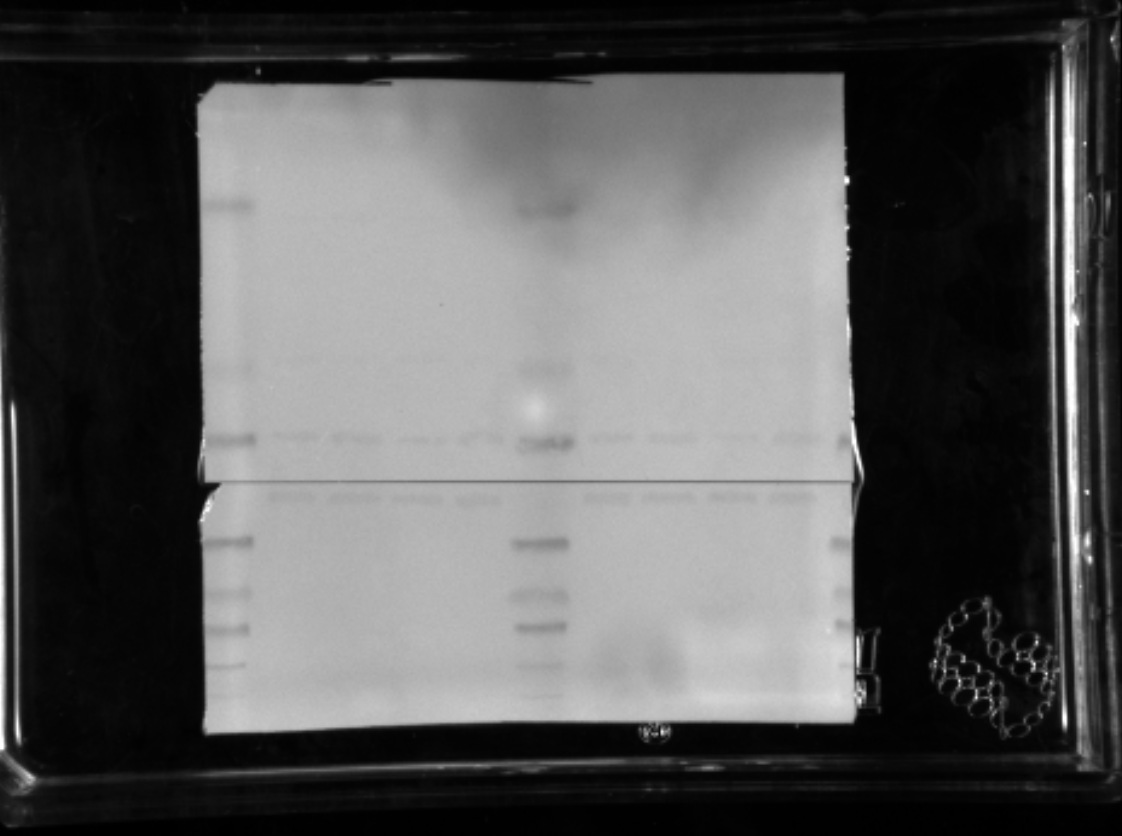

Supplement: S5 File — (ZIP) [file pone.0335225.s010.zip › Response experiment WB/V/图maker 1.tif]
