# Supplementary material for: Plasma-activated media inhibits epithelial-mesenchymal transition and ameliorates intestinal fibrosis through the PPARγ/TGF-β1/SMAD3 pathway
Source: PLoS One. 2025 Oct 22;20(10):e0335225. doi: 10.1371/journal.pone.0335225 (PMC12543144; doi:10.1371/journal.pone.0335225)

Image acquisition: Soak the membrane in the ultra-sensitive ECL (enhanced chemiluminescence) substrate and expose it on the fully automatic chemiluminescence gel imaging analysis system (ChemiDoc XRS+). Select an appropriate exposure time (0-60 seconds) and exposure interval to ensure that the exposed image is clear and save the image.

Fig. 3F (mouse)

The sample addition sequence is Con, DSS, L-PAM, M-PAM, H-PAM, 5-ASA.

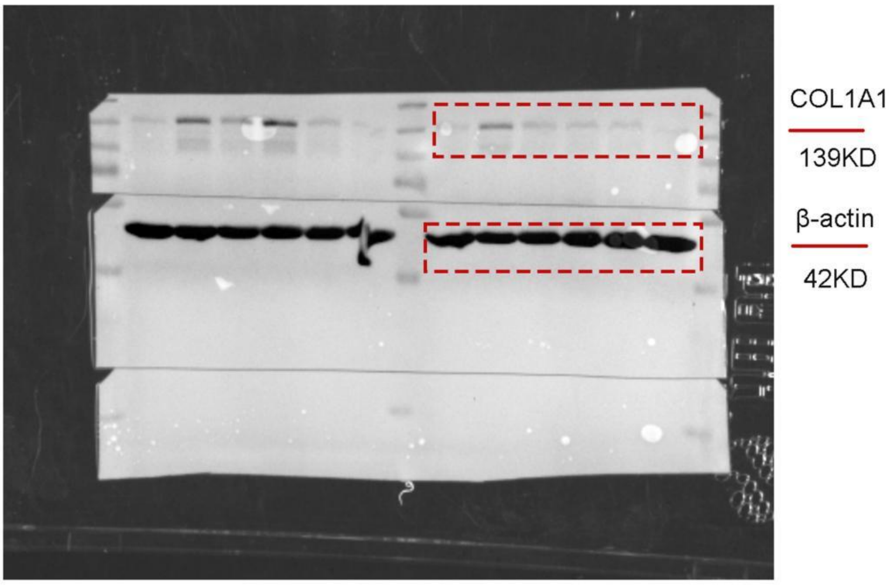

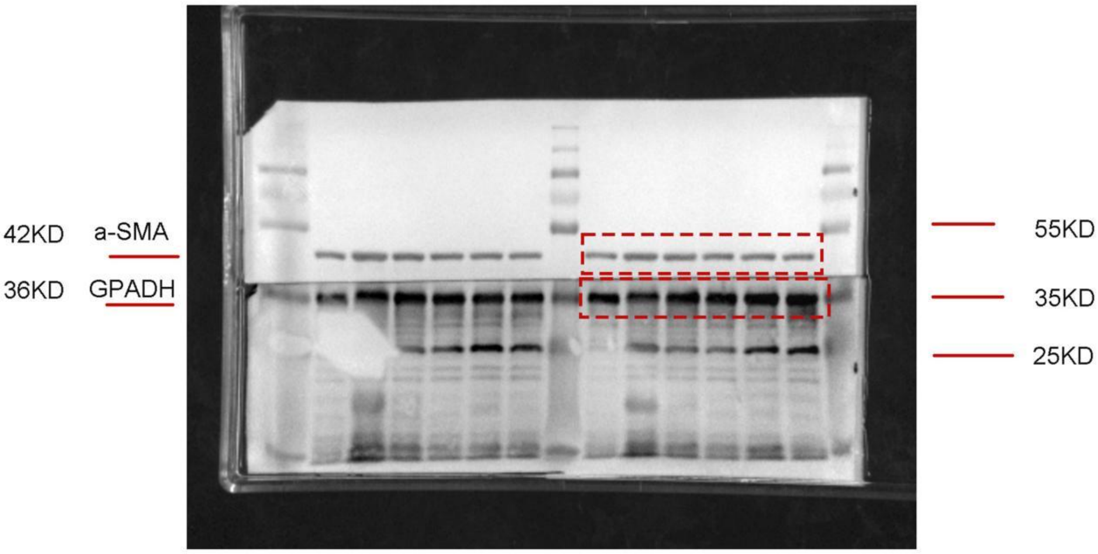

Fig. 3H (mouse)

The sample addition sequence is Con, DSS, L-PAM, M-PAM, H-PAM.

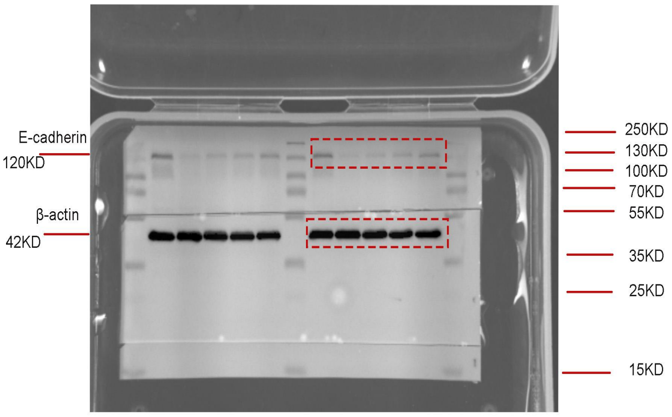

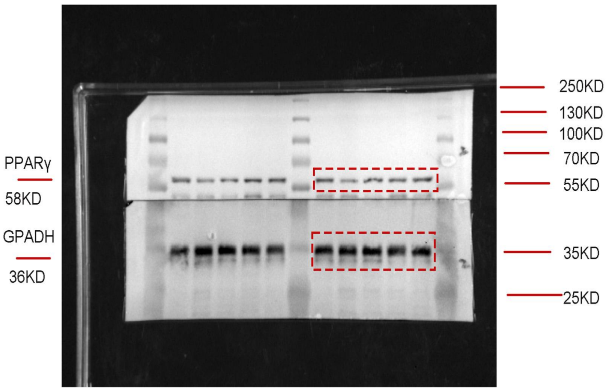

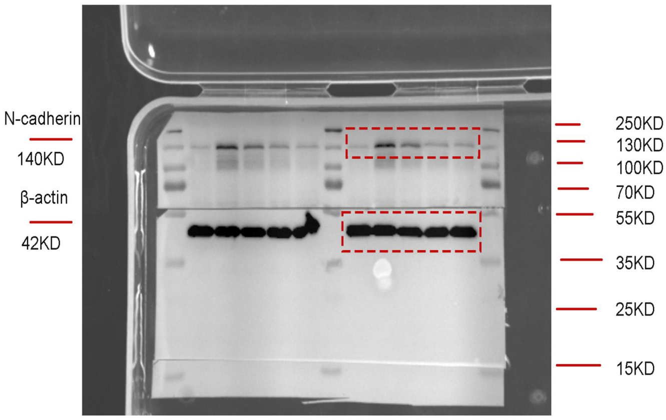

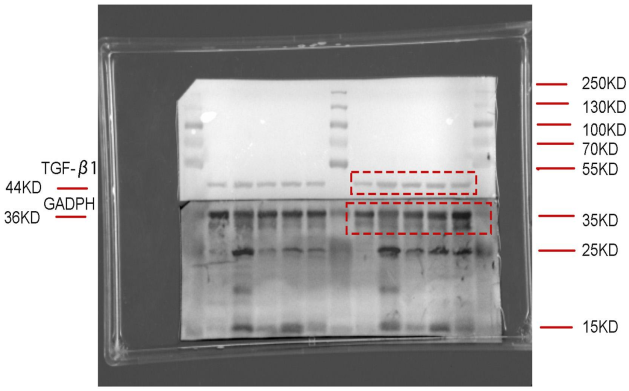

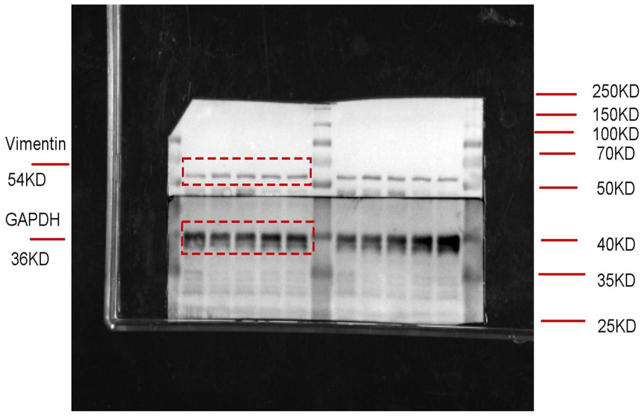

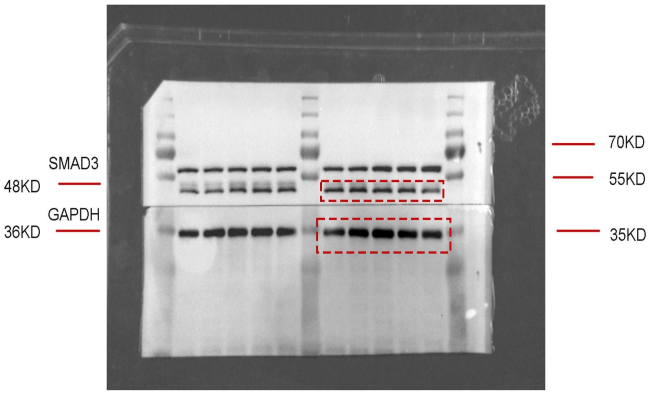

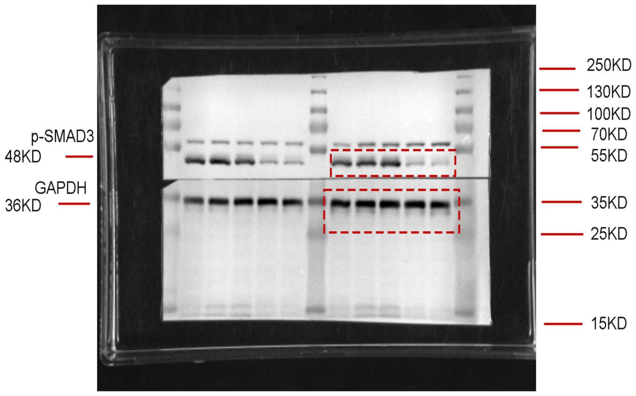

Fig. 5F (rat)

The sample addition sequence is Con, TGF-β1, 2%PAM, 4%PAM, 6%PAM.

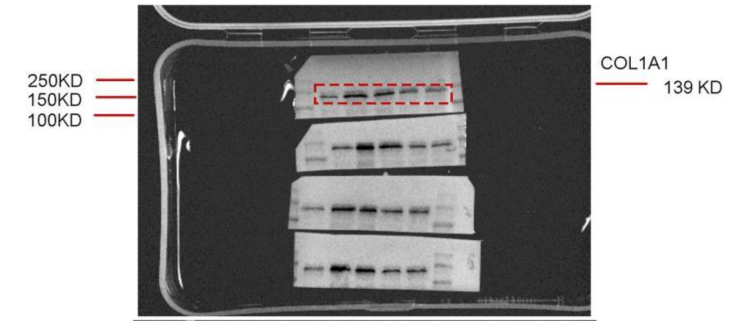

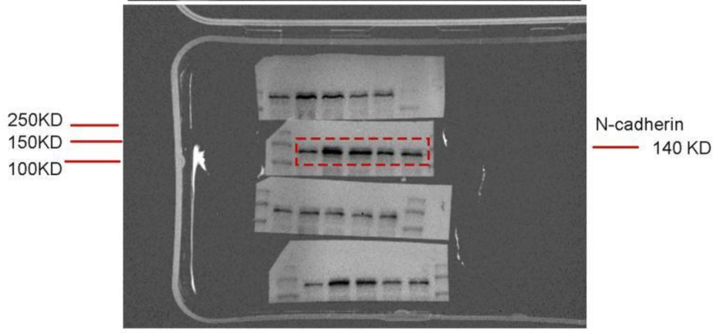

Fig. 5H

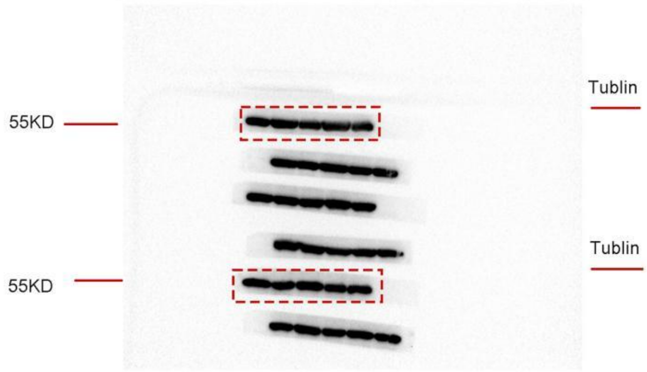

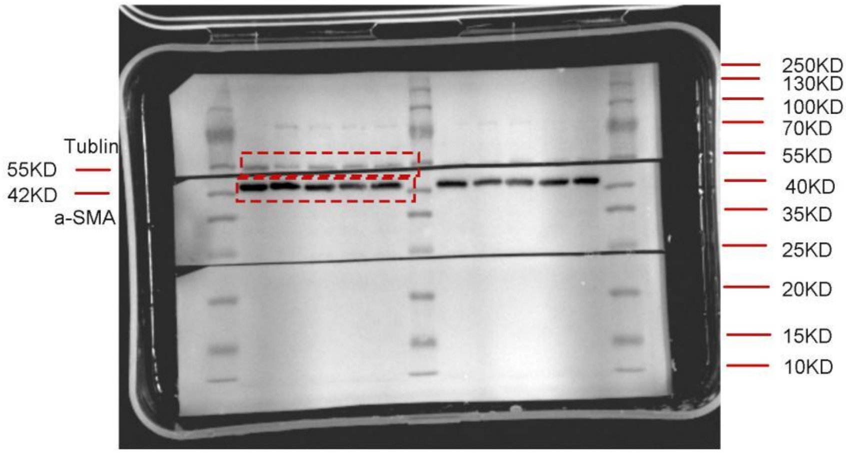

Fig. 5H (rat)

The sample addition sequence is Con, TGF-β1, 2%PAM, 4%PAM, 6%PAM.

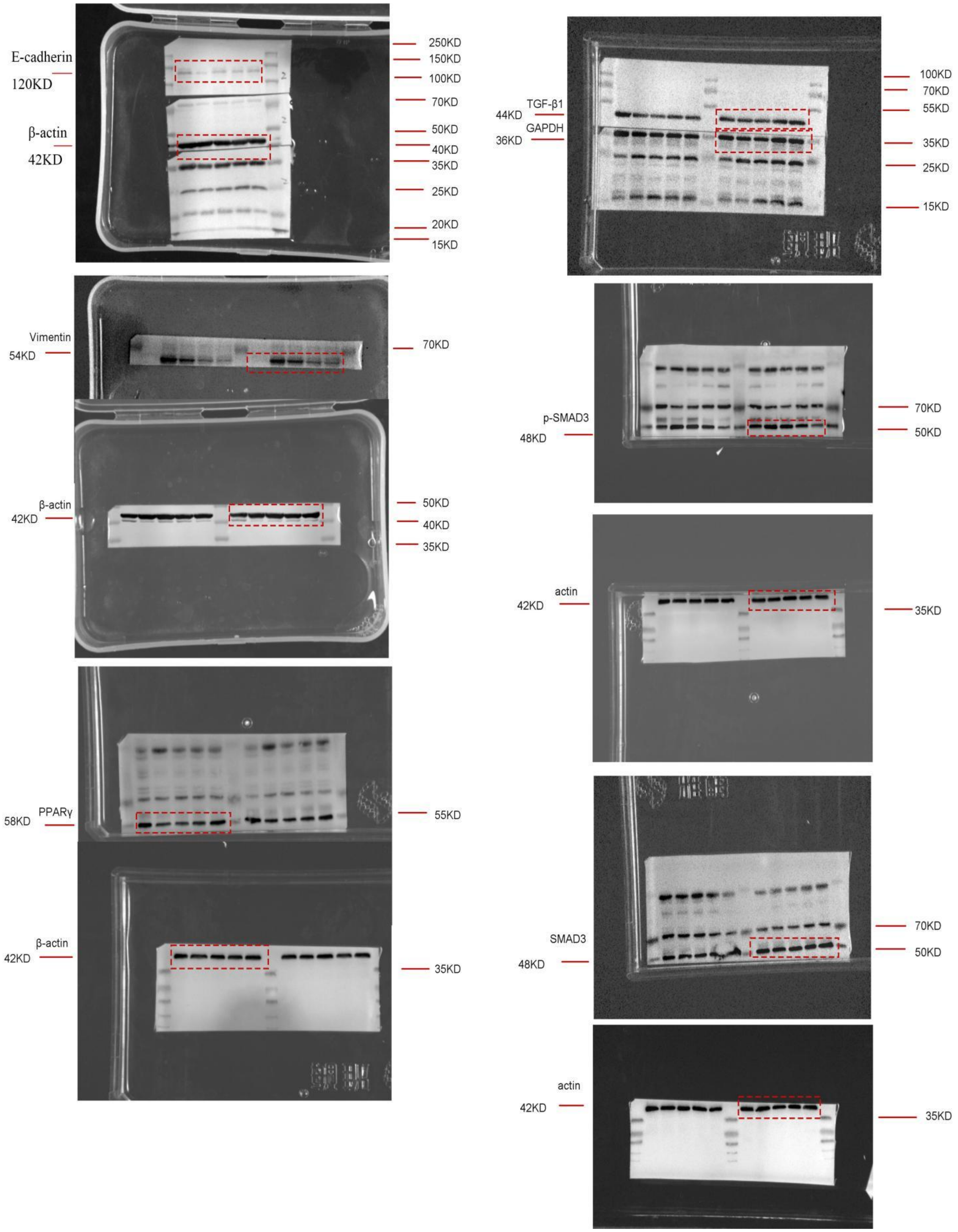

Fig. 7G (rat)

The sample addition sequence is Con, TGF-β1, 6%PAM, GW9662.

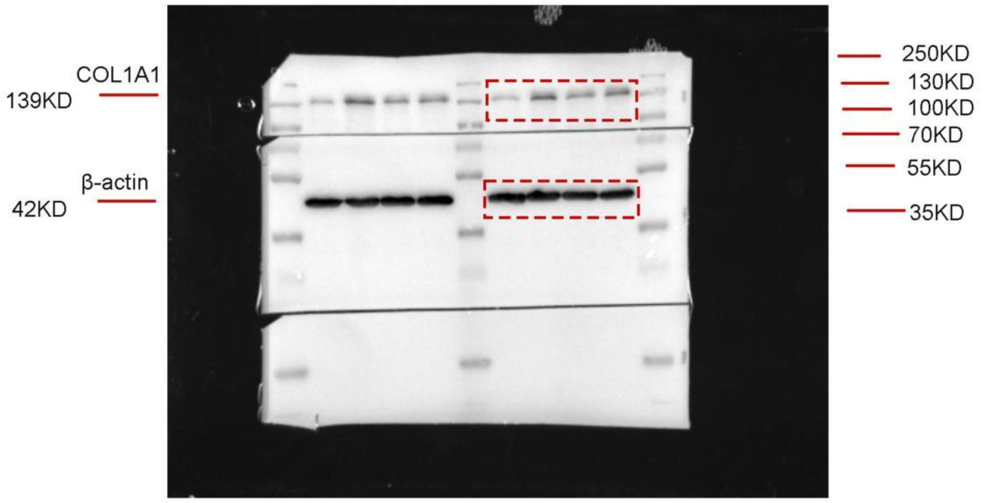

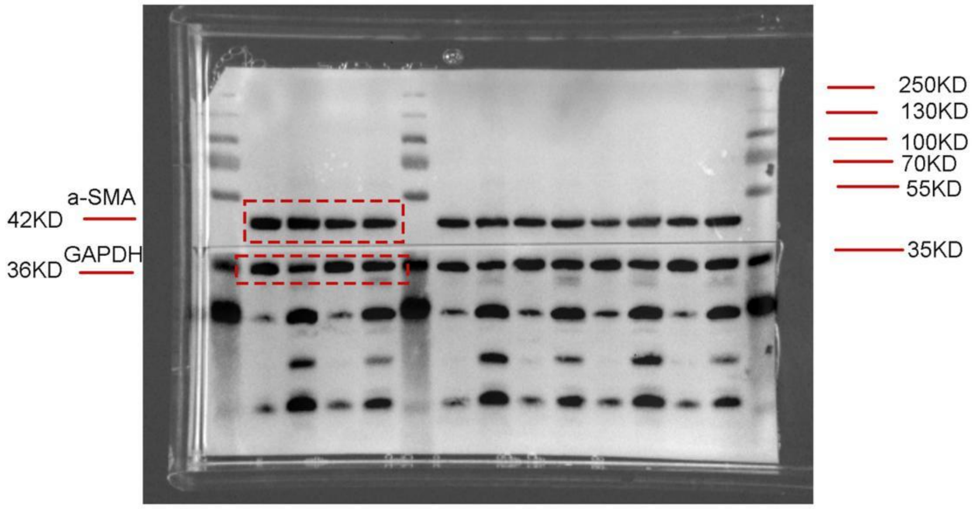

Fig. 7J (rat)

The sample addition sequence is Con, TGF-β1, 6%PAM. GW9662.

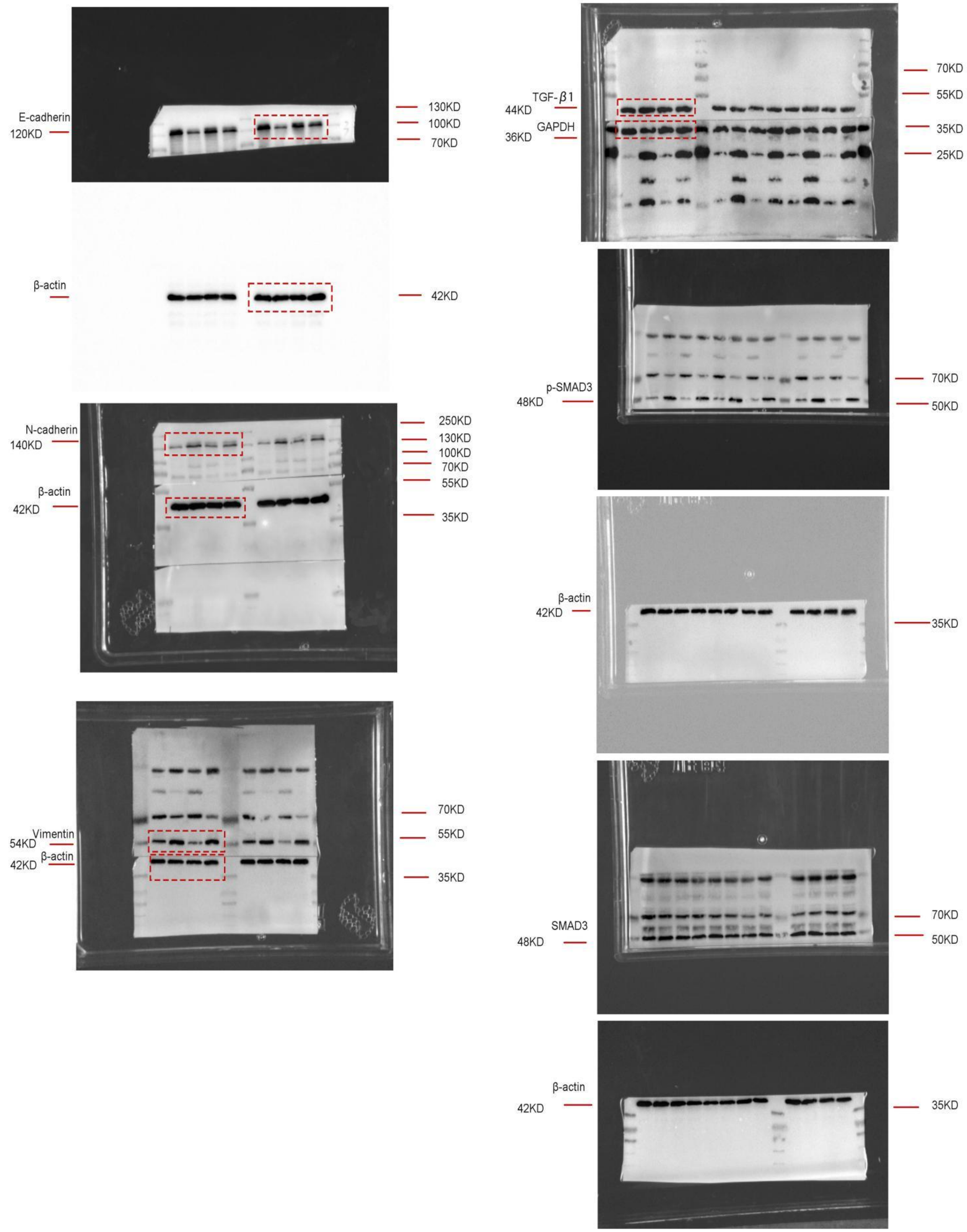

Supplement: S6 File — (PDF) [file pone.0335225.s011.pdf]
